# Smoking and alcohol consumption patterns among elderly Canadians with mobility disabilities

**DOI:** 10.1186/1756-0500-6-218

**Published:** 2013-06-04

**Authors:** Fang Liu, Jennifer Woodrow, Angela Loucks-Atkinson, Sharon Buehler, Roy West, Peizhong Peter Wang

**Affiliations:** 1 Division of Community Health and Humanities, Faculty of Medicine, Memorial University of Newfoundland, St. John’s, Newfoundland & Labrador, Canada; 2 School of Human Kinetics and Recreation, Memorial University of Newfoundland, St. John’s, Newfoundland & Labrador, Canada

**Keywords:** Aging, Alcohol drinking, Health behavior, Life style, Mobility limitation, Smoking

## Abstract

**Background:**

Mobility disability is a major adverse health outcome associated with aging and an impediment to older adults’ well-being and behaviors in social and leisure activities. It has been shown that lifestyle factors, including smoking and alcohol consumption, have been used as coping strategies to deal with the negative impact of disability. The aim of this study was to determine the prevalence of smoking and alcohol consumption among older Canadians with different levels of mobility disabilities and to examine factors associated with these two lifestyle patterns among those with disabilities.

**Methods:**

Secondary data analysis was performed using individuals (n = 6,038) aged 65 years and older from both the 2001 Participation and Activity Limitation Survey and the 2003 Canadian Community Health Survey. Multivariate logistic regressions examined the relationship between disability severity and smoking as well as alcohol consumption while controlling for potential confounding socioeconomic factors.

**Results:**

The proportion of current smokers among seniors with less-severe and more-severe mobility disabilities and those in the general population was comparable with 12.55%, 11.57% and 11.93%, respectively. Forty-eight percent of seniors in the general population consumed alcohol regularly, compared to only 12.85% with more-severe mobility disabilities. No significant association was shown between the severity level of mobility disabilities and smoking (odds ratio = 0.90, 95% confidence interval: 0.75, 1.08). However, seniors having more-severe disability were less likely to consume alcohol regularly (odds ratio = 0.76, 95% confidence interval: 0.65, 0.89). Other variables including age, gender, income, living status, and social participation also impacted these lifestyle patterns among the study population.

**Conclusions:**

Smoking and alcohol patterns present different associations with the severity level of mobility disabilities. Compared with the general population, elderly Canadians with mobility disabilities had similar smoking prevalence but differ significantly in terms of alcohol consumption. Results from this research will be relevant to decision makers involved in program planning, health education, and policy development as it pertains to the prevention and management of age-related disability.

## Background

The rapidly increasing aging population is becoming a worldwide health issue in the first half of the 21st century. According to population projections from 2005 to 2031, Canada’s population is aging rapidly and it is expected that senior citizens will outnumber children in the next ten years [1]. Canada is ranked as the 30th largest country in the world in terms of percentage of population aged 60 or over according to the United Nations’ 2009 report on World Population Ageing [2]. The International Classification of Functioning, Disability and Health (ICF) defines disability as an umbrella term for impairments, activity limitations and participation restrictions that is the result of the interaction between contextual factors (personal and environmental) and health conditions [3]. Disability is a particularly useful concept in assessing the health of seniors [4]. In 2006, there were over 1.76 million Canadians aged 65 and over who had a disability. The disability rate is even higher among older seniors: 33.0% of seniors aged 65 to 74 have a disability whereas 56.3% of seniors aged 75 and over have a disability. Furthermore, older seniors are more likely to have severe or very severe disabilities: 33.6% of seniors aged 65 to 74 with a disability indicate that their level of disability is severe or very severe, compared to 44.4% of seniors aged 75 or over [5].

The most common disability experienced by seniors in Canada is mobility disability defined as limitations in performing various daily activities such as walking up and down a flight of stairs [6]. More than 1.3 million (or one-third) of all Canadian seniors have mobility disabilities. Senior women are more likely to have a mobility disability than senior men (37.2% versus 28.1%) [5]. Existing studies have identified mobility disabilities as a major adverse health outcome associated with aging and an impediment to older adults’ well-being and behaviors in social and leisure activities [7].

Lifestyle factors describe the way people live their lives and include behaviour and social issues that impact health and well-being; examples include smoking, diet, nutrition, sedentary lifestyle, and alcohol or substance misuse. Research has shown that lifestyle is affected by mobility impairment among a significant number of people worldwide [8]. Several studies have also shown that unhealthy lifestyle factors are associated with physical decline as one ages [9]. Having two or more unhealthy lifestyle factors was found to be a strong predictor of mobility limitation among non-obese older adults [10]. Current research also indicates that lifestyle factors, including smoking and alcohol consumption, may be used as coping strategies to deal with the negative impact of disability including stress experienced by individuals with mobility-limited conditions such as fibromyalgia syndrome [11,12]. Smoking and alcohol consumption were found to be directly correlated with the socioeconomic resources of individuals [13], however individuals with mobility disabilities had different socioeconomic patterns compared to the general population [14,15]. Smoking is less prevalent among Canadian seniors than the younger population, with an estimated 9% of seniors aged 65 years and older being current smokers (either daily or occasional) [16]. The rate of alcohol use among seniors is also lower than that of younger age groups [16]. Data on alcohol consumption patterns among older Canadians are not available. However, research indicates that approximately 48% of adults aged 65–74 years and 38% of adults 75 years of age and older in the United States consume alcohol regularly [17].

Given that lifestyle modifications may serve as potential interventions to reduce mobility limitations [18], determining patterns of certain behaviors among individuals with varying degrees of mobility disabilities may provide further insight into the negative impact of mobility limitations on lifestyle and the development of coping strategies. Providing insight into the various dimensions of lifestyle behaviors is essential for setting up public health programs that deal with mobility issues. This study hypothesized that older adults were more likely to report unhealthy lifestyle patterns as the severity level of disability increased considering that smoking and alcohol consumption have been used as coping strategies. It was also hypothesized that several factors may be associated with selected lifestyle behaviors including income, gender, age, living status, self-perceived health, social participation and education. Given these hypotheses, the objectives of this study were two-fold: 1) to describe the prevalences of smoking and alcohol consumption behaviors among a sample of Canadians aged 65 and over with mobility disabilities and compare them to those of the general elderly population; and 2) to measure factors and potential determinants associated with these two lifestyle patterns among the older Canadian population with mobility disabilities.

## Methods

### Study population and design

This study is a secondary analysis of data from the 2001 Participation and Activity Limitation Survey (PALS) and the 2003 Canadian Community Health Survey (CCHS) conducted by Statistics Canada. CCHS 2003 is a cross-sectional survey that gathered information related to health status, health care utilization and health determinants of the Canadian population 12 years of age and over living in the ten provinces and the three territories. The survey excluded persons living on reserves and other Aboriginal settlements in the provinces; full-time members of the Canadian Forces; the institutionalized population; and persons living in the Quebec health regions of Région du Nunavik and Région des Terres-Cries-de-la-Baie-James.

PALS 2001 was a post censual survey designed to gather information on socio-demographic factors (e.g., education, employment, economic characteristics) and disabilities experienced by Canadians and the impact of these disabilities on daily living [see Additional file 1]. The target population of this survey consisted of all persons with a physical, sensory, or psychological disability who were living in Canada at the time of the 2001 Census, including permanent residents of most collective dwellings and health care institutions. Aboriginal reserves and settlements were not included in the 2001 PALS. Individuals also excluded for operational reasons were residents of penal institutions, correctional facilities, military camps, campgrounds and parks, soup kitchens, merchant and coastguard ships. A filter question in the 2001 Canadian Census (long form) was designed to identify individuals for more in-depth disability questions during the post-censual disability survey. The filter question was as follows: “Is this person limited in the kind or amount of activity that he/she can do because of a long-term physical, mental or health problem?” All PALS participants were asked to indicate whether they had one or more of five physical activity limitations. These were difficulties in walking half a kilometer or up and down a flight of stairs (about 12 steps) without resting; moving from one room to another; carrying an object of 5 kg for 10 meters; or standing for long periods. Those who answered “yes” to any one of these activities were defined as having mobility disability in this study.

Probability proportional-to-size sampling method was used in both the CCHS and PALS data collections. Smoking and alcohol consumption patterns in the general elderly Canadian population were calculated using CCHS 2003 with a sub-sample of 21,170 individuals aged 65 and over. The two lifestyle patterns among older adults with mobility disabilities were calculated using PALS 2001; from the total sample of 20,710 records, a sub-sample of 6,038 individuals aged 65 years and older with mobility disabilities was selected for the study.

### Variables and measurements

Self-reported smoking and alcohol consumption patterns were used as outcome variables separately. In both PALS and CCHS, these variables were dichotomously collapsed. For smoking patterns, two groups were defined: current smokers and non-current smokers at the time when participants were completing the survey. In CCHS, current and non-current smoking data was obtained directly from the derived variables by Statistics Canada. In PALS, participants were asked to report their current smoking patterns. In the current study, individuals who answered “not at all” regarding current smoking frequency were considered non-current smokers; those who responded “regularly” and “occasionally” were categorized as current smokers; other answers including “don’t know”, “refusal” and “not stated” were treated as missing values.

The following question was used to collect information on drinking behaviour: “In the past twelve months, how often have you had a drink?” Study participants could choose one of the provided answers: never; every day; 4–6 times a week; 2–3 times a week; once a week; once or twice a month; less than once a month; don’t know and refusal. Definitions for frequent/regular versus infrequent/non-regular alcohol consumption vary significantly in the literature and largely depend on the questions and response options/categories in a particular survey [19-21]. Given the alcohol consumption categories used in this study, those who consumed alcohol at least once per week were classified as regular alcohol drinkers and those who reported consuming alcohol less than once per week as non-regular alcohol drinkers; other responses were treated as missing values. There was no information on types of alcohol in the PALS questionnaire. However, in Canada, a drink is commonly understood as one beer, one small glass of wine, or 1.5 ounces of liquor [22].

With respect to independent variables, annual income was categorized as “low” and “high” with the cutting point of $30,000 Canadian dollars (CAD) (i.e., an income of more than $30,000 was considered “high”); this included the total income received during the calendar year of 2000 from all resources including wages, all kinds of benefits, income from government sources, interests and investment income and other money income. Considering the importance of having other individuals residing with those with mobility disabilities, the variable “living status” was considered rather than “marital status” in the study. The corresponding question in PALS is “Number of persons in household”; those who answered “one person” were considered as living alone, while other answers were considered as living with others. Social participation, defined as people's social involvement and interaction with others, was also taken into account and was self-derived from 8 categories: visiting family or friends; walking or playing sports; doing hobbies; shopping; attending sports or culture events; taking courses; visiting museums/libraries/parks; and travelling. Participants were asked to indicate how often they participated in these activities in a typical week on an ordinal scale (everyday; at least once a week; at least once a month; less than once a month; never; refusal; don’t know). Individuals who reported engaging in the aforementioned activities at least once per week were considered as active in social participation; those engaged less than once per week were considered as non-active.

Other independent variables included age (65–69; 70–74; 75–79; 80+), sex (male; female), self-perceived health (excellent; very good; good; fair; poor), and the severity level of mobility disability. The severity of disabilities used in this study was provided by Statistics Canada. It was based on complex and weighted scoring methods taking into account both the frequency and intensity of a set of disability questions. A final single numerical score was used to reflect the overall severity level of disability for an individual. In the 2001 public PALS database, the disability severity variable has four levels: mild, moderate, severe, and very severe. In our study, this variable was dichotomized into less severe (mild and moderate) and more severe (severe and very severe). More details regarding the scaling system used by Statistics Canada can be found elsewhere [23] and have also been summarized in Additional file 2. For all variables, values of non-response, refusal or blank were defined as missing values.

### Statistical analysis

The study performed both descriptive analyses and multivariate logistic regressions using the statistical software SAS version 9.1 (SAS Institute Inc., Cary, NC, USA). Odds ratios (OR) and corresponding 95% confidence intervals (CI), as the results from logistic regressions, were used to estimate the effects; alpha levels of 0.95 were used to examine the statistical significance. Potential interactions between the study variables were tested. Each record containing more than two missing values was deleted. Estimation weights were adjusted to bring data from public use microdata files into line with the census-based population in order to account for the unequal distribution for the strata and groups based on province, age and sex. The rescaled weight, achieved by dividing the original weight by the mean weight among selected sub-samples, was used to produce descriptive estimates.

### Ethical issues

The micro data used in the current study were secondary data collected by Statistics Canada for research and public use. Therefore, there were no ethical considerations directly related to data collection in this study. No individual or personal identifiable information was released from Statistics Canada.

## Results

Table 1 describes the prevalence of smoking and alcohol consumption among elderly Canadians with respect to the severity level of mobility disabilities. As previously discussed, data for the general older adult population (aged 65 and older) came from CCHS 2003 while the data on older adults with mobility disabilities came from PALS 2001. Consequently, comparisons are not from the same data set and are provided for descriptive purposes rather than to make statistical inferences. The prevalence of current smokers among individuals having less-severe and more-severe mobility disabilities was 12.55% and 11.57%, respectively. Similar prevalence was found among older adults in the general population with a

**Table 1:** Prevalence of Smoking and Alcohol Consumption Among Elderly Canadians, PALS 2001, CCHS 2003

	**Prevalence of Smoking**	**Prevalence of Alcohol Consumption**
	**%**	**%**
General elderly population^a^	11.93	48.08
(n = 21,170)		
Elderly population with less-severe mobility disabilities^b^	12.55	19.37
(n = 6,038)		
Elderly population with more-severe mobility disabilities^b^	11.57	12.85
(n = 6,038)		

Abbreviations: *CCHS*, Canadian Community Health Survey; *PALS*, Participation and Activity Limitation Survey.

^a^ Data came from CCHS 2003.

^b^ Data came from PALS 2001.

 proportion of 11.93%. Therefore, smoking status was relatively the same for seniors with mobility impairments and the general older adult population, and regardless of the severity of the mobility disability. However, the patterns of alcohol consumption were significantly different. The proportion of alcohol consumption significantly decreased with the increase of severity level of mobility disabilities. Approximately 50% of elderly Canadians in the general population consumed alcohol at least once per week, which is consistent with current literature. However, only 12.85% of older adults with more-severe mobility disabilities consumed alcohol. As severity of disability increases, weekly alcohol consumption decreases with the older adult population.

Table 2 summarizes the results of the univariate and multiple logistic regressions which analyzed how the independent variables affected the odds of smoking among elderly Canadians with mobility disabilities. Using less-severe mobility disability as the baseline, unadjusted results showed increased levels of disability were negatively associated with smoking (OR = 0.84, 95% CI: 0.72, 0.99). However, after adjusting for potential confounders, no statistically significant association was found between disability levels and smoking. As anticipated, being female and increased age were negatively associated with smoking status in both the univariate analysis and after adjusting for potential confounding variables (multivariate analysis). Having a higher income, living with someone else, and being active in social participation also decreased the likelihood of smoking among the study population; these odds were significant for the multivariate but not the univariate analysis. For the consideration of self-perceived health, the poorer people rated their health, the more likely they smoked with every decreased level in self-perceived health ratings (OR = 1.14, 95% CI: 1.05, 1.23). Effect modifications for living alone,

**Table 2:** Summary Statistics on Variables and Weighted ORs for Smoking Status Among Seniors With Mobility Disabilities

**Variable and level**	**Total**	**Univariate**	**Multivariate**
	**(n = 6,038)%**	**OR (95% CI)**	**OR (95% CI)**
**Severity of disabilities**			
Less-severe	58.97	1.00	1.00
More-severe	41.03	0.84 (0.72, 0.99)	0.90 (0.75, 1.08)
**Gender**			
Male	37.40	1.00	1.00
Female	62.60	0.61 (0.52,0.71)	0.58 (0.49, 0.69)
**Age**			
65-69	20.20	1.00	1.00
70-74	22.75	1.41 (1.18, 1.68)	0.65 (0.52, 0.79)
75-79	23.83	0.85 (0.71, 1.03)	0.42 (0.33, 0.52)
80 and over	33.22	0.30 (0.24, 0.37)	0.19 (0.14, 0.24)
**Annual Income**			
<=$30,000 (CAD)	84.42	1.00	1.00
>$30,000 (CAD)	15.58	0.81 (0.64, 1.01)	0.67(0.52, 0.85)
**Living status**			
Living with others	32.75	1.00	1.00
Living alone	67.25	1.13 (0.96, 1.34)	1.67 (1.40, 2.00)
**Self-perceived health**			
Every decreased scale		1.14 (1.05, 1.23)	1.10 (1.01, 1.19)
**Social Participation**			
No	21.48	1.00	1.00
Yes	78.52	0.93 (0.77, 1.13)	0.70 (0.56, 0.86)

Abbreviations: *CAD*, Canadian dollar; *CI*, confidence interval; *OR*, odds ratio; *PALS*, Participation and Activity Limitation Survey.

 the severity level of disabilities, and social participation were tested, but none of the interactions were significant at a level of 0.05.

Table 3 summarizes the results of the univariate and multiple logistic regressions which analyzed how the independent variables affected alcohol consumption among elderly Canadians with mobility disabilities. Using less-severe mobility disability as the baseline, both adjusted and unadjusted odds ratios indicated that increased level of disability significantly impeded regular alcohol consumption. Unlike smoking patterns, living alone had statistically significant association with alcohol consumption; however this was not significant after adjusting for confounding variables. Individuals who were active in social participation and those with a higher income had a significant higher likelihood of consuming alcohol, a reverse effect compared to that of smoking status. The poorer people rated their health, the less likelihood that they consumed alcohol regularly. Being female and increased age were negatively associated with drinking alcohol. Effect modifications for living alone, the severity level of disabilities and social participation were also tested, but none of the interactions were significant at a level of 0.05.

## Discussion

The primary objective of this study was to investigate the association of mobility disability with smoking status and alcohol consumption. After comparing the prevalence of three groups (general elderly Canadians, elderly Canadians with less-severe mobility disabilities and elderly Canadians with more-severe mobility disabilities), there were no significant differences in the prevalence of smoking. Conversely, the prevalence of alcohol consumption was negatively associated with the severity level of mobility disabilities; the prevalence of alcohol consumption among older adults with a more-severe level of mobility disability is approximately one quarter that of the general elderly population. After adjusting for potential confounders, odds ratios were consistent with the results from descriptive statistics and unadjusted results. This study found that mobility disabilities impact alcohol consumption more significantly than smoking among elderly Canadians.

**Table 3:** Summary Statistics on Variables and Weighted ORs for Alcohol Consumption Among Seniors With Mobility Disabilities

**Variable and level**	**Total**	**Univariate**	**Multivariate**
	**(n = 6,038)%**	**OR (95% CI)**	**OR (95% CI)**
**Severity of disabilities**			
Less-severe	58.97	1.00	1.00
More-severe	41.03	0.57 (0.50, 0.66)	0.76 (0.65, 0.89)
**Gender**			
Male	37.40	1.00	1.00
Female	62.60	0.33 (0.29,0.37)	0.35 (0.31, 0.41)
**Age**			
65-69	20.20	1.00	1.00
70-74	22.75	1.23 (1.06, 1.43)	0.86 (0.71, 1.05)
75-79	23.83	1.01 (0.87, 1.18)	0.76 (0.63, 0.93)
80 and over	33.22	0.56 (0.48, 0.65)	0.50 (0.41, 0.60)
**Annual Income**			
<=$30,000 (CAD)	84.42	1.00	1.00
>$30,000 (CAD)	15.58	1.87 (1.59, 2.19)	1.40 (1.18, 1.67)
**Living status**			
Living with others	32.75	1.00	1.00
Living alone	67.25	0.83 (0.72, 0.95)	1.13 (0.97, 1.32)
**Self-perceived health**			
Every decreased scale		0.83 (0.78, 0.89)	0.85 (0.79, 0.91)
**Social Participation**			
No	21.48	1.00	1.00
Yes	78.52	1.79 (1.49, 2.15)	1.31 (1.07, 1.61)

Abbreviations: *CAD*, Canadian dollar; *CI*, confidence interval; *OR*, odds ratio; *PALS*, Participation and Activity Limitation Survey.

Lifestyle factors including smoking and alcohol consumption have been found to be used as coping strategies to deal with the negative impact of disability [11]. This is not surprising since depression has been shown to be positively associated with mobility disabilities [24], and smoking and alcohol consumption are often used to deal with emotional problems or stressful situations [16,25,26]. One might also expect that an increase in smoking and/or alcohol use as the severity level of mobility disability increases may be indicative of using these lifestyle behaviours as coping strategies. However, this study did not show such an association and thus there was no evidence indicating the two lifestyle patterns may be used as coping strategies in the study population. Furthermore, additional data, such as mood status, feelings of depression, personal versus social alcohol consumption, and behaviour changes related to smoking and alcohol consumption, would be required to substantiate the use of these behaviours as coping mechanisms in the study population. Increased alcohol consumption among the study population with higher income is consistent with the general population [27,28].

The potential effects of alcohol consumption on certain health issues may partially explain a decrease in alcohol use with increased severity level of disability. Seniors' bodies process alcohol more slowly and are therefore more vulnerable to the effects of alcohol than are younger adults [25]. Alcohol reduces muscle control, which increases the risk of falling for seniors and can also exacerbate certain health issues, including confusion and memory loss, liver damage, diabetes, heart or blood pressure problems, and stomach problems [25]. Furthermore, over 150 medications commonly prescribed to seniors may result in problems if consumed with alcohol; some may not work as they are meant to, while others may have an increased or dangerous effect [25]. Individuals with increased severity of mobility disability may require more medication and may be more inclined to avoid situations that enhance risk of further impairment. This may lead to reduced alcohol intake for this sub-population.

Social behaviour patterns may also influence a person’s involvement in smoking and alcohol consumption in this sub-population. In the current study, greater social participation was associated with a decreased likelihood of smoking but an increased likelihood of consuming alcohol. As generally believed, social behaviors are affected and impeded by mobility disability due to the limitations and restrictions in daily life [29]. Long-term disability was found to be accompanied by a substantial effect on social isolation, which limited social behaviour [30]. In addition, lower socio-economic status, such as low income and low education, also account for the lack of involvement in social behaviors within this specific population compared with the general population [31,32]. These results are consistent with the ICF model which contends that restrictions in participation are correlated with functioning/structure impairments, environmental factors and personal factors [3]. Compared to smoking, alcohol consumption is more likely to involve a social context and is more likely to occur among a group of people [33]. Thus, severity of mobility disability is related to reduced social participation and thus reduced alcohol consumption. In contrast, social participation may be a protective factor against smoking due to its decreased social acceptance in current Canadian culture [34,35] and the prohibitions placed concerning smoking in public places. Thus, the lack of social behaviour of individuals with mobility disabilities could be used to partially explain why mobility limitations have a more pronounced role in affecting alcohol consumption patterns than smoking.

The strength of this research is that it is a national population-based study with a relatively large sample size (n = 6,038), weighted to take into account the unequal distribution for the strata and groups based on province, age and sex. It also provides further insight for examining interventions or strategies to improve the population health of older adults with mobility disabilities. Study limitations must also be acknowledged. First, the study relied on self-reported data, which might lead to over-estimation or under-estimation due to inaccurate recall. While alcohol consumption was measured for a period of 12-months, smoking was measured only at the point of conducting the survey. This could possibly cause information bias in the misclassification of the outcome. Second, although alcohol consumption was found to be negatively associated with the severity of mobility limitations, the nature of the cross-sectional study design could not provide us with a temporal relationship, and thus the causal effect of this relation could not be determined. Third, the public use microdata file of PALS 2001 failed to provide some important information. For example, education level and depression have been found to be associated with mobility disabilities [24,36,37]; however, this information was not available for the study population. Lack of information on depression, and failure in classifying personal alcohol consumption and social alcohol consumption, made it impossible to evaluate the impact of alcohol consumption as coping strategies to deal with loneliness and depression that may result from having a mobility disability. Geographical information and the duration of engaging in social participation were also not provided, which causes more residual confounding [38].

## Conclusions

In summary, the results indicate that smoking and alcohol patterns present different associations with the severity level of mobility disabilities. Compared with the general population, elderly Canadians with mobility disabilities had similar smoking prevalence but differ significantly in terms of alcohol consumption. Being female, higher income, increased age, living with someone else and being active in social participation were negatively associated with smoking after adjusting for potential confounders. For alcohol consumption, individuals who were active in social participation and those with higher income tended to have a higher likelihood of consuming alcohol, which showed a reverse effect compared to that of smoking status.

Healthy aging programs and policies aim to ensure people have the best possible physical, emotional, social, and mental health and well-being as they age. Certain individual behaviours may significantly impact health and well-being for older Canadians such as physical activity, healthy eating and nutrition, smoking, alcohol and other substance use, and use of medications [16]. Results from this research may be used to inform intervention programs or policies that address unhealthy lifestyle behaviours among the aging population, especially those programs and services that support independence, mobility and safety of older persons. It is important to identify factors associated with smoking and alcohol consumption as these may further contribute to public health issues involving those with mobility disabilities. Moreover, it is essential to continue to explore the subject of mobility disability, its association with various lifestyle factors and the potential impact on the aging population.

### Availability of supporting data

The CCHS and PALS public use microdata files (PUMF) used in this study were made available through Statistics Canada’s Data Liberation Initiative (DLI) License agreement with Memorial University. Under this agreement, use of data files is restricted to faculty, staff and students of Memorial University for teaching and non-commercial research; copies of data resources may not be distributed in whole or in part outside Memorial University. While the data files cannot be provided by the authors, they may be obtained directly through Statistics Canada:

PALS data: http://www5.statcan.gc.ca/bsolc/olc-cel/olc-cel?lang=eng&catno=82M0023X

CCHS data: http://www23.statcan.gc.ca/imdb/p2SV.pl?Function=getDatafileData&Item_Id=46547&lang=en&db=imdb&adm=8&dis=2

## Competing interests

There are no competing interests, financial or non-financial, to declare.

## Authors’ contributions

An early version of this work was part of FL’s MSc thesis. PPW, ALA, SB, and RW contributed to the conceptualization of this paper. With input from other co-authors, FL and JW drafted and finalized this manuscript. All authors read and approved the final manuscript.

## Supplementary Material

Additional file 1 Provides information to facilitate the consultation and manipulation of the public use microdata file (PUMF) for the Participation and Activity Limitation Survey (PALS) conducted by Statistics Canada in 2001. It contains information on the survey objectives, methodology and estimation methods and on the rules for disseminating estimates based on the survey data. It also describes how to use the PUMF correctly. Click here for file

Additional file 2 Provides a summary of Participation and Activity Limitation Survey (PALS) 2001 disability scale. Click here for file
