# Supplementary material for: Smoking and alcohol consumption patterns among elderly Canadians with mobility disabilities
Source: BMC Res Notes. 2013 Jun 4;6:218. doi: 10.1186/1756-0500-6-218 (PMC3680044; doi:10.1186/1756-0500-6-218)
Supplement: Additional file 1 — Provides information to facilitate the consultation and manipulation of the public use microdata file (PUMF) for the Participation and Activity Limitation Survey (PALS) conducted by Statistics Canada in 2001. It contains information on the survey objectives, methodology and estimation methods and on the rules for disseminating estimates based on the survey data. It also describes how to use the PUMF correctly. [file 1756-0500-6-218-S1.pdf]

Participation and Activity Limitation Survey (PALS) 2001:

User's Guide to the Public Use Microdata File

Statistics Canada Statistique Canada

Canada

How to obtain more information

Specific inquiries about this product and related statistics or services should be directed to: Housing, Family and Social Statistics Division, Statistics Canada, Ottawa, Ontario, K1A 0T6 (telephone: (613) 951-5979).

For information on the wide range of data available from Statistics Canada, you can contact us by calling one of our toll-free numbers. You can also contact us by e-mail or by visiting our Web site.

**National inquiries line**	**1 800 263-1136**
**National telecommunications device for the hearing impaired**	**1 800 363-7629**
**Depository Services Program inquiries**	**1 800 700-1033**
**Fax line for Depository Services Program**	**1 800 889-9734**
**E-mail inquiries**	**infostats@statcan.ca**
**Web site**	**www.statcan.ca**

Standards of service to the public

Statistics Canada is committed to serving its clients in a prompt, reliable and courteous manner and in the official language of their choice. To this end, the Agency has developed standards of service which its employees observe in serving its clients. To obtain a copy of these service standards, please contact Statistics Canada toll free at 1 800 263-1136.

Statistics Canada
Housing, Family and Social Statistics Division

Participation and Activity Limitation Survey (PALS), 2001 :

User's Guide to the Public Use Microdata File

Published by authority of the Minister responsible for Statistics Canada

© Minister of Industry, 2004

All rights reserved. No part of this publication may be reproduced, stored in a retrieval system or transmitted in any form or by any means, electronic, mechanical, photocopying, recording or otherwise, without prior written permission from Licence Services, Marketing Division, Statistics Canada, Ottawa, Ontario, Canada K1A 0T6.

October 2004

Catalogue no. 82M0023GPE

Frequency: Occasional

ISBN: 0-660-19356-6

Ottawa

Cette publication est disponible en français (n° 82M0023GPFau catalogue).

Note of appreciation

Canada owes the success of its statistical system to a long-standing partnership between Statistics Canada, the citizens of Canada, its businesses, governments and other institutions. Accurate and timely statistical information could not be produced without their continued cooperation and goodwill.

Symbols

The following standard symbols are used in Statistics Canada publications:

.	not available for any reference period
..	not available for a specific reference period
...	not applicable
0	true zero or a value rounded to zero
0^s	value rounded to 0 (zero) where there is a meaningful distinction between true zero and the value that was rounded
^{p}	preliminary
^{r}	revised
x	suppressed to meet the confidentiality requirements of the *Statistics Act*
^{E}	use with caution
F	too unreliable to be published

Table of Contents

1. INTRODUCTION AND SUMMARY ____ *3*

2. OBJECTIVES OF PALS ____ *6*

3. CONTENT AND SPECIAL CHARACTERISTICS OF PALS ____ *7*

4. TARGET POPULATION AND SAMPLING DESIGN ____ *8*

5. DATA COLLECTION ____ *10*

6. DATA PROCESSING ____ *11*

7. ESTIMATION ____ *13*

8. GUIDELINES ON DATA DISSEMINATION AND RELIABILITY ____ *17*

9. ADDITIONAL INFORMATION ____ *21*

Appendix A. Rules for Obtaining the Approximate Variance

Appendix B. Survey Questionnaire

Appendix C. Dictionary of Data File

- *by section*
- *by alphabetical order of variables in the file*

Appendix D. File Record Layout

Appendix E. Document Discussing Changes between the 1991 HALS and the 2001 PALS

Appendix F. Disability Severity Scale

Appendix G. Type of Disability among Adults

Appendix H. Weighted and Unweighted Frequencies for the Population without Disabilities

1. INTRODUCTION AND SUMMARY

1.0 Introduction

This document is intended to facilitate the consultation and manipulation of the public use microdata file (PUMF) for the Participation and Activity Limitation Survey (PALS) conducted by Statistics Canada in 2001. It contains information on the survey objectives, methodology and estimation methods and on the rules for disseminating estimates based on the survey data. It also describes how to use the PUMF correctly. Appendix A concerns the rules for obtaining the approximate variance. Appendix B contains the questionnaire. Appendix C contains the data dictionary. Appendix D presents the PUMF record layout. Appendix E is a document describing the changes between the 1991 HALS and the 2001 PALS. The disability severity scale and a typology of disabilities are presented in appendixes F and G respectively. Weighted and unweighted frequencies for population without disabilities are presented in Appendix H. This document is also available in print format on request.

1.1 Participation and Activity Limitation Survey 2001 — Summary of Concepts, Methodology and Data Quality

It's recommended to read this section to ensure that you have a good grasp of the basic concepts that define the PUMF data from PALS 2001, along with the underlying survey methodology and the main aspects of data quality. With this information, you will better understand the strengths and limitations of the data and how to analyse them properly. This information could be especially useful when making comparisons with data from other sources, or drawing conclusions. You will find additional details in the sections that follow.

1.1.1 Data Sources and Methodology

PALS is a post-censal survey designed to collect information on adults and children with disabilities, that is, those whose everyday activities are limited because of a condition or health problem.

Data collected by the survey will be used to plan the services and programs required by persons with disabilities to participate fully in our society. 2001 PALS was funded by Human Resources Development Canada (HDRC).

The most recent data collected on persons with disabilities before PALS is from the 1991 Health and Activity Limitation Survey (HALS). **In order to better identify the PALS target population, major changes were made to the structure of the sample and the filter questions identifying persons with disabilities. Therefore, comparisons between the 1986 and 1991 HALS and 2001 PALS surveys are not possible.** On the other hand, these filter questions can now serve as a standard for identifying persons with disabilities in other Statistics Canada surveys. For additional information on the new survey approach, see the appended document entitled *A New Approach to Disability Data: Changes between the 1991 Health and Activity Limitation Survey (HALS) and the 2001 Participation and Activity Limitation Survey (PALS).*

1.2.1 Overall Methodology

The PALS survey population consisted of all persons who reported disabilities in response to the Census questions on activity limitations and who were living in Canada at the time of the Census.

This is a voluntary survey. Interviews were conducted over the telephone by interviewers completing a paper-and-pencil questionnaire. Interviews by proxy were allowed. In some special cases, face-to-face interviews were conducted

1.2.1.i Reference Period

Data collection began in September 2001 and continued until January 2002. The reference period varied depending on the question asked, e.g., the past week, the past six months, the past year or the past five years.

1.2.1.ii Estimation

Estimation weights were adjusted by post-stratification to bring them into line with census-based population estimates for the strata and groups based on province, age and sex.

1.2.2 Concepts and Variables Measured

To collect information on adults whose everyday activities are limited because of a condition or health problem, the PALS questionnaire identifies ten types of disabilities. However, for reasons of confidentiality related to the PUMF, four types of disabilities were grouped as "Other." As a result, the typology provided to PUMF users contains seven types of disabilities, namely hearing, seeing, speech, mobility, agility, pain and "other" disabilities. Appendix G contains a description of this typology.

For the degree of disability severity, PALS constructed a scale measuring the overall severity according to the intensity and frequency of the activity limitations reported by respondents. The disability severity scale for adults is divided into four levels: mild, moderate, severe and very severe. To increase the analytical usefulness of the PUMF, a second severity index was also developed for each of the seven types of disability identified in the PUMF. These indexes were developed using the same model as for the overall index, except that they contain only two levels of severity: least severe and most severe. The list of variables identifying each index is as follows:

DEG_HEARP
DEG_SEEP
DEG_SPCHP
DEG_MOBP
DEG_AGILP
DEG_PAINP
DEG_OTHEP

For further information on the severity scale, see Appendix F.

The PUMF documentation includes an annotated list of all data elements included in the file, as well as the complete questionnaire used. Section 3 of this documentation gives a detailed description of the content, outlined below:

i. Filter questions
ii. Activity limitations

iii. Help with everyday activities
iv. Education
v. Employment status
vi. Social participation
vii. Economic characteristics

1.2.3 Data Accuracy

The survey methodology was designed to control errors and reduce their potential effects. However, the results are still subject to sampling error and non-sampling error (that is, the difference between an estimate and the real value that is not due to sampling).

1.2.3.i Sampling Error

Since the data are collected from a sample of individuals, they are subject to sampling error. In other words, estimates based on a sample vary from one sample to another, and usually they differ from the results that would have been obtained from a complete census. The probable extent of this difference was estimated for important data. From this information, a table was produced that can be used to estimate the sampling variability of many estimates. For this table, along with instructions for its use and examples, see Appendix A or Section 8 of the User's Guide documentation.

1.2.3.ii Non-sampling Error

Even estimates based on a census of the target population may contain errors. These are non-sampling errors, and estimates based on sample data may also contain them. Common sources of these errors are incomplete coverage, non-response, response errors and data processing errors.

2. OBJECTIVES OF PALS

PALS is a post-censal national survey designed to collect information on adults and children with disabilities, that is, those whose everyday activities are limited because of a condition or health problem.

PALS provides essential information on:

- the prevalence of various disabilities,
- support for persons with disabilities,
- their employment profile,
- their income and their participation in society.

The data collected by PALS, combined with those from the Census, are an important source of information about specific characteristic traits of persons with disabilities. This is therefore a database that can be used by all levels of government, associations, researchers and non-governmental organizations to support the planning of services that persons with activity limitations require in order to participate fully in society.

3. CONTENT AND SPECIAL CHARACTERISTICS OF PALS

As noted above, even though PALS 2001 is the successor to the 1986 and 1991 HALS, data from the two surveys cannot be compared owing to major differences in their sampling methods, the operational definition of their target population and the content of their questionnaires.

The adult questionnaire for PALS was developed based on a review of the 1991 Health and Activity Limitation Survey (HALS) questionnaires and input consultation with the client (HRDC), the federal and provincial governments and community associations. It includes seven sections focusing on the different aspects of activity limitations, broken down as follows:

***Section A**:* the filter questions asked in the 2001 Census, which identify the PALS target population, are repeated at the start of the PALS interview;

***Section B**:* types of activity limitations; their severity; the use of, need for and costs of medications, aids and specialized equipment; and underlying health problems and their causes;

***Section* C**: the use of, need for and costs of help with everyday activities and disability-related health needs;

***Section D**:* education, the use of and need for supportive measures, and the impact of the disability on the educational profile and experience;

Section E: the employment profile, the use of and need for supportive measures, and the impact on employment status and experience;

Section F: impact on social participation in terms of adaptability and accessibility of leisure and recreation, transportation and housing;

Section G: income-related characteristics, such as insurance plans, tax credits and income sources.

A pilot test was conducted in the fall of 2000 to test the content of the questionnaires, in both official languages.

4. TARGET POPULATION AND SAMPLING DESIGN

PALS, a national survey, is termed post-censal because it uses the census as a sampling frame for identifying its target population.

4.1 Target Population

The target population of PALS consists of individuals living in private households and some non-institutional collective households who answered "yes" to either question 7 or 8 in the 2001 Census (form 2B), which identify persons with disabilities. However, for operational reasons, persons living in Yukon, Nunavut or the Northwest Territories, on an Indian reserve or in an institutional collective dwelling were excluded from the sampling frame. The population targeted by PALS represents 18.6% of Canada's adult population and 5% of the child population.

4.2 PALS Sampling Design

The PALS sampling plan can be considered as a two stage stratified design which uses the 2001 Census long form sample. This long form contains two general filter questions on activity limitations and long-term disabilities. The 2001 PALS selected a sample of individuals from respondents on the Census long form who reported a positive response to at least one of these two filter questions. These respondents are said to be "disabled individuals" according to the Census. PALS uses, however, its own definition of disability and the PALS disabled population is a subset of the Census disabled population.

The sampling frame used for PALS consists of projections of the 2001 Census disabled population by age group and severity of disability within each Enumeration Area (EA). The strata are defined by the cross-classification of the ten provinces, four age groups and the Census severity of disability (defined by the response categories "Often" and "Sometimes"). Each Primary Sampling Unit (PSU) is made up geographically of one or more Census EAs and is defined within a severity and age group stratum. Although a given EA can be selected for more than one severity and age group combination, a PSU is defined in only one stratum. The PSU size comes from the projection of the Census disabled population for the combination of EA(s), age group and severity of the PSU.

At the first stage, PSUs are sampled using probability proportional-to-size (PPS) sampling. In the second stage of the sample design, all Census long-form respondents in a selected PSU are included in the 2001 PALS sample. The second stage sampling design is therefore the one of the 2001 Census long form, which is in most EAs, a systematic sample of one in five households.

The original size of the PALS sample totalled 43,276 individuals, including 35,424 adults and 7,853 children, living in private households and collective dwellings. An overall response rate of 82.5% was obtained. For purposes of the PUMF, analysts based themselves on the 28,908 persons aged 15 and over who responded to the survey. This included 22,134 adults who reported having at least one limitation in their activities at the time of the survey and 6,774 adults who reported being limited in their activities at the time of the census but indicated no limitation in PALS.

It should be noted that for analytical purposes, a stratified random sample of 130,000 individuals (101,874 adults and 28,126 children) was selected from the census frame, consisting of

individuals who reported no disability or activity limitation in the census. The sample was allocated proportionally to the size of the population included in each stratum, thus producing a self-weighted sampling design. The strata used in the selection of this sample were formed by cross-classifying a geographic variable, an age-related variable and the sex variable. A portion of the adults thus selected was also included in the PUMF, partly to enable users to calculate disability rates and compare the population with and without disabilities for particular characteristics. Thus, a sample of nearly 50,000 individuals without disabilities was selected using the same sampling design and added to the PUMF.

5. DATA COLLECTION

As noted above, PALS data were collected directly from respondents, from September 2001 to January 2002. Interviews were conducted by telephone, with interviewers completing a paper-and-pencil questionnaire. Interviews by proxy were allowed. In some special cases, face-to-face interviews were conducted. Respondents were interviewed in the official language of their choice.

The interviews were conducted from centralized telephone facilities set up in four regional offices of Statistics Canada, in Halifax, Sturgeon Falls, Vancouver and some cases were handled in Edmonton. Calls were made between 9 a.m. and 9 p.m., from Monday to Saturday inclusively. Statistics Canada employees gave the interviewers training in survey concepts and procedures and interview methods.

Additional information is available from Statistics Canada (see Section 9). The following manuals were used during the survey:

PALS 2001 Training Guide
PALS 2001 Interviewer Manual

6. DATA PROCESSING

6.1 Data Capture

Completed questionnaires were sent from the regional offices to the head office in Ottawa, where responses to the questions were captured either manually or using scanners with intelligent character recognition. Answers to open-ended questions were all captured manually.

6.2 Coding

For questions for which it was possible to develop a written answer, responses were assigned either a code representing a new category or the code for a category listed on the questionnaire if the information fell into an existing category. Where applicable, responses were coded according to appropriate classification systems, namely ICD-9, NAICS or SOC-96. Also, codes from NOC-S 2001 were derived using SOC-96 concordance tables.

6.3 Edit and Imputation

For PALS, the only type of imputation done was deterministic imputation. Once inconsistencies were identified between responses, a corrective action had to be used on at least one of the responses. The approach used to determine the appropriate action was generally the "bottom-up" approach. With this strategy, questions related to each other were edited simultaneously. If the answer to Question A determined that Question B was to be asked, then Question B was edited first and the edited responses to B were then used to determine if the response to Question A was correct. If both responses were inconsistent, the response to Question A was modified deterministically if possible. Conversely, for a small number of questions, a "top-down" approach was used. With this approach, responses to previous questions determined whether a subsequent question was to be asked. Although the corrections were generally done in an automated way, analysts reviewed some problem situations.

During this review, a valid response was deterministically imputed for the missing responses if sufficient information was available in the related questions. Otherwise, it was coded to "Not stated". In addition, the questions that were not to be asked were coded to "Not applicable". If a question with a missing answer (coded to "Not stated") should have been used to determine if subsequent questions were to be asked, these subsequent questions were set to "Path not known", because it was not possible to determine whether or not they should have been asked.

However, non-response was not permitted for the demographic information required for weighting, namely the age and sex of the respondent. This information was asked at the beginning of the interview to make sure the selected person had been reached. These two variables were imputed from the census if they were missing or invalid. In particular, an age was considered invalid if it was not consistent with the questionnaire used.

6.4 Creation of Combined and Derived Variables

Some variables in the file were derived from information collected on the questionnaire. In some cases, derived variables are simple ones formed by grouping several categories. In other cases, two or more variables were combined to create a new one. The data dictionary specifies which variables are derived and how this was done.

6.5 Level of Detail of the Microdata File

In order to ensure the non-disclosure of confidential information, the level of detail of the PUMF is not as fine as that of the master file kept by Statistics Canada. Actions were also taken to make the microdata file more secure from disclosure of confidential information. These actions concern the geography included in the file, survey weights, overlaps with other PUMFs yet to be published, exclusion of variables, grouping of categories for some variables, capping of some extreme numerical values, as well as identification of unique records at risk and rare occurrences.

As a result, the PUMF on adults with disabilities contains 20,710 records. Each record represents one respondent and provides data for most sections of the questionnaire. The records each contain 756 variables, including 622 original variables from the PALS questionnaire, 103 variables derived the original variables in the survey, and 31 variables from the census.

The PUMF on adults without disabilities—needed in part to enable users to calculate disability rates and compare the population with and without disabilities for particular characteristics—contains 55,550 records. For these, 33 variables are available, all drawn from the 2001 Census.

A complete list of these variables is provided in the data dictionary (appendix C).

7. ESTIMATION

In a probabilistic sample such as the PALS sample, estimation is based on the principle that each person included in the sample represents not only him/herself but also a number of other persons who were not included in the sample. For example, in a simple random sample of 2% of the population, each person represents 50 members of the population (him/herself and 49 others). The number of persons represented by a given respondent is what is known as the respondent's weight or weighting factor.

A weighting factor is included in the PALS microdata file:

WEIGHT_P : This is the weight for analysis with respect to persons, that is, for calculating estimates of the number of persons (included in the target population) with one or more of specified characteristics. WEIGHT_P should be used to calculate all estimates. For example, to estimate the number of persons who are married or living in common-law unions, it is necessary to sum the WEIGHT_P values for all records that include this characteristic (MARSTHP=2).

7.1 Weighting

As noted above, PALS 2001 is a survey of individuals, and the microdata file contains responses to the questionnaire and related information provided by 20,710 respondents.

Calculating the weight for the PUMF is a four-stage process:

1) Calculating the initial weight

The first stage is the assignment of an initial weight based on the sampling design. The initial weight is the inverse of the probability of inclusion in the sample. For the 2001 PALS, the initial weight was the product of three components: the inverse of the sampling ratio of the Primary Sampling Unit (called the PSU weight), the census weight and the subsampling weight. Following this calculation, individuals selected by mistake and those missed during sample selection were taken into consideration and the appropriate weight adjustments were applied to the initial weight.

2) Correction for non-response

The second stage of the weighting process is adjustment for non-response. More specifically, two adjustments were done at this stage to reflect the fact that non-respondents can be classified into two main categories with very different characteristics: the persons not contacted and the persons who were contacted but did not respond.

Weights were adjusted first for non-contact and then for non-response. Since the adjustment method was the same for the two types of non-respondents, it is described here for non-response only. The non-response was done by forming non-response adjustment classes in such a way that the records in each class had similar response probabilities. Estimated response probabilities were obtained by developing a logistic regression model to predict the response probability using explanatory variables.

Many explanatory variables can be used, since all census long form information is available for each respondent and non-respondent. Separate models were used for children and for adults.

Approximately ten classes of roughly the same size were obtained for each logistic regression model. The inverse of the weighted response rate in a class was used as the weighting adjustment factor for that class, and the initial weights of the respondents within the class were adjusted accordingly.

3) Post-stratification

The third stage of the weighting adjustment is post-stratification. This adjustment ensures that the sum of the final weights for the respondents is equal to the population counts obtained from the census. This adjustment is done for groups (called post-strata) defined by the combinations of province, sex, age group (generally five-year age groups) and severity of the activity limitation(s) reported in the census. The weights corrected for non-response were then adjusted using the ratio of the census count to the sample count for each post-stratum. These final weights were used to produce all estimates for the different releases of PALS data.

4) Additional adjustments of weights for the PUMF

The PUMF is a subsample of the PALS adult sample. Thus, additional adjustments had to be made to the weights of units in the PUMF to take account of the units removed. To do this, the weight obtained in stage 3 was first multiplied by the subsampling weight. Then a new post-stratification was needed in order to adjust the census counts. Finally, some random noise was added to the resulting weight as an additional measure to ensure confidentiality.

7.2 Weighting Guidelines

Users should not disseminate any unweighted total or perform analyses based on unweighted survey results. As explained in Section 7.1, several weight adjustments were made based on province, stratum, age and sex. Sampling rates and non-response rates vary considerably from one stratum to another, and non-response rates also vary according to demographic characteristics. Clearly, then, unweighted sample counts cannot be considered as representative of the population targeted by the survey.

7.3 Types of Estimation

As noted above, a basic weight was assigned to each person sampled, and as described in Section 7.1, these weights were adjusted to reflect the age and gender composition of the Canadian population as estimated by Statistics Canada.

$$\sum_{i=1}^{20,710} \text{WEIGHT\_P} = 3,420,340$$

= an estimate (rounded to ten) of the number of persons aged 15 and over in the Canadian population who reported having at least one activity limitation in the survey.

Using PALS data, two types of "simple" estimates can be calculated: qualitative estimates (estimates of numbers or proportions of persons with certain attributes or characteristics) and quantitative estimates (estimates of quantities or averages). Section 7.4 deals with more complex estimates and analyses.

7.3.1 Qualitative Estimates

Qualitative estimates are estimates of the number or percentage of persons in the population targeted by the survey who have a certain characteristic or fall into a defined category. The values of these variables represent a quality rather than a quantity. An example of a qualitative estimate is the number or proportion of persons who reported "High school graduation certificate" as the highest level of schooling completed.

Qualitative estimates can be obtained by summing the final weights of all records that contain the characteristic(s) of interest. Proportions and ratios of the form $\hat{Y}/\hat{W}$ are obtained by following the steps below:

(i) sum the final weights of records containing the characteristic of interest $\hat{Y}$;

(ii) sum the final weights of records containing the characteristic of interest $\hat{W}$;

(iii) divide the result obtained in (i) by the result obtained in (ii), namely $\hat{Y}/\hat{W}$.

7.3.2 Quantitative Estimates

Quantitative estimates are estimates of totals or means, medians or other measures of central tendency representing quantities. The number of weeks or hours worked is an example of a quantitative estimate.

This type of estimate can be obtained by multiplying the value of the variable of interest by the final weight of the corresponding record and summing this amount for all records selected.

To obtain a weighted average of the form $\hat{Y}/\hat{W}$, the numerator ($\hat{Y}$) is calculated in the same way as a quantitative estimate and the denominator ($\hat{W}$) in the same way as a qualitative estimate. For example, to estimate the average number of hours worked by respondents, proceed as follows:

(i) estimate the total number of hours worked by respondents ($\hat{Y}$) by multiplying the number of hours worked by each respondent by its corresponding final weight, then sum this value for all respondents;

(ii) estimate the number of respondents ($\hat{W}$) by summing the final weights for all records corresponding to a respondent;

(iii) divide (i) by (ii), namely $\hat{Y}/\hat{W}$.

7.4 Guidelines for Analysis

As explained in detail in Section 4, PALS respondents do not constitute a simple random sample of the target population. The survey is based on a complex sampling design that calls for multi-stage stratification and sampling. Consequently, the selection of respondents was done according to unequal probabilities.

Survey weights must therefore be used in making estimates and analyses so that insofar as possible, the over- or under-representation of age-sex groups in the unweighted file can be taken into consideration. The use of data from such a complex survey can pose problems for analysts,

since the choice of methods of estimation and variance calculation depends on the sampling design and selection probabilities. A number of analysis methods integrated into statistical packages allow the use of weights, but the meaning and definition of these weights often differ from those that apply in the context of a sample survey. Therefore, while the estimates made using these packages are often accurate, the variances calculated are practically meaningless.

In many methods of analysis (such as linear regression, logistic regression, estimation of rates or proportions and analysis of variance), the application of current software packages can be made more meaningful by standardizing the weights that appear in the records so that the average weight is equal to 1. The results produced by traditional packages are thus more reasonable, because even though they do not always reflect the stratification and clustering in the sampling design, they take account of selection with unequal probabilities. This standardizing can be done by dividing each weight by the overall average weight before proceeding to the analysis.

For an analysis of all respondents who are married or living in a common-law union, the procedure to follow is as follows:
- from the file, select all respondents who were living common-law (MARSTHP=2);
- calculate the average value of WEIGHT_P for all these records;
- for each of these respondents, calculate a "working" weight equal to WEIGHT_P /average weight;
- carry out the analysis for these respondents using the "working" weight.

Section 8 gives a more detailed description of sampling variability and data reliability, and Appendix A contains the rules for obtaining the approximate variance for estimating the sampling variability of a large number of qualitative estimates of proportions.

8. GUIDELINES ON DATA DISSEMINATION AND RELIABILITY

It is important for the user to become familiar with the content of this section before publishing or otherwise disseminating any estimate calculated using PALS microdata files.

This section of the document gives guidelines that users of the microdata file must follow. Users will thus be able to obtain figures which are consistent with those produced by Statistics Canada and which conform to established guidelines on rounding and dissemination. The guidelines fall into four major categories: minimum sample size for producing estimates; sampling variability; estimation of variance; and rounding.

8.1 Minimum Sample Size for Producing Estimates

The user must determine the number of records in the microdata file that provided the data entering into the calculation of a particular estimate. If the number is less than or equal to 10, the weighted estimate must generally not be disseminated, regardless of its approximate coefficient of variation. If the estimate is nevertheless disseminated, this must be done with considerable caution, and the user should clearly indicate that the estimate is based on an insufficient number of records.

8.2 Sampling Variability

Estimates drawn from the survey are based on a sample of individuals. Different figures might have been obtained if a complete census had been conducted using the same questionnaire, interviewers, supervisors, data processing methods, etc. The difference between an estimate produced from a sample and one produced from a complete enumeration conducted in similar conditions is called the sampling error of the estimate.

Errors unrelated to sampling can occur at almost any stage of a survey. Interviewers may misunderstand the instructions, respondents may make mistakes when answering questions, responses may be miskeyed into the computer or errors may occur when the data are processed or totalled. These are all examples of non-sampling errors.

If there are a large number of observations, random errors have little effect on estimates based on data from the survey. However, errors that occur systematically bias estimates. Much time and effort has been devoted to reducing non-sampling errors. At each stage of the data collection and processing cycle, quality assurance measures were applied in order to control data quality. These measures included using highly skilled interviewers, giving them intensive training in survey methods and the questionnaire, observing interviewers in order to detect problems caused by the questionnaire design or a failure to understand the instructions, adopting procedures to minimize data capture errors, and implementing coding controls and edits to confirm the processing logic.

8.2.1 Non-sampling Errors

The effect of non-response on survey results is a major source of non-sampling error. Non-response may be either partial (not answering one or more questions) or total. There is total non-response when the interviewer is unable to locate the respondent or the respondent cannot provide the desired information (perhaps because of a language problem) or refuses to participate in the survey. Cases of total non-response are treated by correcting the weight applied to those persons who responded to the survey to compensate for those who did not.

In most cases, there was partial non-response to the survey where the respondent misunderstood or misinterpreted a question or was unable to remember the information requested. In PALS, no responses were imputed to compensate for partial non-response, and the question was assigned the response code "Not stated."

8.2.2 Sampling Errors

Since estimates based on a sample survey inevitably contain sampling errors, good statistical methods require researchers to inform users of the magnitude of this type of error.

Although it is not possible to obtain an exact measure of the sampling error of an estimate as defined above using the sample data alone, it is possible to estimate a statistical measure of this error, namely the standard error, using these data. Based on the standard error, confidence intervals can be obtained for estimates (not taking the effects of non-sampling errors into account) on the assumption that the distribution of the estimates around the true value of the population is normal. In these conditions, the chances that the deviation between an estimate based on the sample and the true value for the population is less than one standard deviation are 68 in 100, while the chances that it is less than two standard deviations are approximately 95 in 100, and it is virtually certain that it is less than three standard deviations.

Since the absolute size of the sampling error of an estimate is often less important than its relative size (compared to the estimate itself), the standard error is not always the best measure of sampling error. For example, a standard error of 10 for an estimate of 20 would generally indicate that the quality of the estimate is poor, while the same standard error for an estimate of 1,000 would generally indicate that the estimate is good. Consequently, the size of the sampling error is often expressed in relation to the size of the estimate, in the form of a coefficient of variation (CV). The coefficient of variation of an estimate is obtained by dividing the standard error of the estimate by the estimate itself and expressing the resulting fraction as a percentage. In the above example, the CV of the first estimate is 50% (10/20), while that of the second is 1% (10/1,000).

Guidelines for Dissemination of Estimates

Before disseminating and/or publishing estimates based on the microdata file, the user should consult the table below and follow the guidelines corresponding to the value of the coefficient of variation of the estimate.

Type of estimate	Coefficient of variation	Guidelines for dissemination
1. Moderate sampling variability	0.0 % to 16.5%	You can consider general, unrestricted dissemination of the estimates. No particular annotation is required.
2. High sampling variability	16.6% to 33.3% (values in yellow in the variance table)	You can consider general, unrestricted dissemination of the estimates with a cautionary note concerning the high sampling variability associated with them.
3. Very high sampling variability	33.4% and over (values in red in the variance table)	The estimates should generally not be disseminated, but if they are, this should be done with great caution, with a clearly visible accompanying note stating that the sampling variability associated with them is very high.

Note: The sampling variability guidelines should be applied to rounded estimates.

8.3 Variance Estimates

In the following description of estimation of variance, qualitative estimates and quantitative estimates are treated separately.

8.3.1 Sampling Variability of Qualitative Estimates

Data variance for PALS was calculated using the bootstrap method. Bootstrap weights were calculated for each survey respondent. With a variance calculation software such as WesVar, variance estimates can be obtained in the form of a coefficient of variation (CV) for various types of estimates in nearly any field. Thus, to obtain an accurate estimate of variance, the bootstrap method should be used. However, bootstrap weights cannot be provided to PUMF users for reasons of confidentiality.

For most users, it would be extremely costly and pointless to calculate the sampling variability of each qualitative estimate that can be drawn from the survey. Therefore, Statistics Canada has produced approximate measures of sampling variability in the form of a table of approximate CVs, so that users can have an idea of the quality of the results they produce with the PUMF.

First, a number of variables in the file were identified that would probably be used most often in analytical tables. Approximate CVs were produced for the domains obtained by cross-tabulating these variables. For each of these domains, a total of ten were simulated several times and approximate average CVs were obtained for each proportion within each domain. CVs were calculated using bootstrap weights that had been adjusted for undersampling.

In the form of an interactive EXCEL application, Statistics Canada provides a table of the approximate variance of estimates produced using WEIGHT_P for Canada. This informatic tool is provided free of charge to all PUMF users.

It should be noted that all coefficients of variation that appear in this table are approximate, and therefore they may not be considered official values. On a cost-recovery basis, Statistics Canada may produce estimates of the real variance for some variables. A decision to use the variance calculated for a specific variable instead of the approximate variance drawn from the tables could give the user more confidence in the quality of the estimates, especially those for which the coefficient of variation estimated according to the tables falls within the "very high sampling variability" range (see above for guidelines on the dissemination of estimates drawn from the survey).

8.3.2 Sampling Variability of Quantitative Estimates

The use of approximate variances for quantitative variables cannot be summarized as easily. However, in general, the coefficient of variation of a quantitative total will be larger than that of the corresponding qualitative estimate (for example, the number of persons whose responses are used in producing the qualitative estimate). If the sampling variability of the qualitative estimate is high, that of the estimate of the quantitative total too will generally be high.

8.4 Rounding

To ensure that estimates produced from PALS microdata files will correspond to those produced by Statistics Canada, the user is strongly advised to follow the rounding guidelines. Disseminating unrounded estimates could be misleading, since such estimates might appear to be more precise than they actually are.

8.4.1 Rounding Guidelines

1) Estimates of totals that appear in the body of a statistical table should be rounded to the nearest ten by the traditional rounding method (see definition in Section 8.4.2).

2) Partial and grand totals in statistical tables should be calculated from their unrounded components, then rounded to the nearest ten by the traditional rounding method.

3) Averages, proportions, rates and percentages should be calculated from unrounded components, then rounded to one decimal by the traditional rounding method.

4) Sums and differences of aggregates or ratios should be calculated from their corresponding unrounded components, then rounded to the nearest ten or the nearest decimal using the traditional rounding method.

5) Because of technical or other constraints, a rounding method other than traditional rounding may be used. In this case, the estimates obtained may differ from the corresponding estimates produced by Statistics Canada. If so, the user is strongly advised to state the reason for these differences in the document disseminated.

8.4.2 Traditional Rounding Method

According to the traditional rounding method, if the first or only figure to be suppressed falls between 0 and 4, the last figure retained does not change. If the first or only figure to be suppressed falls between 5 and 9, the value of the last figure retained is increased by one unit (1). For example, the figure 8,499 rounded to the nearest thousand would be 8,000, while the figure 8,500 rounded to the nearest thousand would be 9,000.

9. ADDITIONAL INFORMATION

Additional information on the survey may be obtained from the persons listed below. Data from the survey are presented in the form of published reports, custom tabulations and microdata files. The file may be obtained from the Housing, Family and Social Statistics Division of Statistics Canada for $3,000 . Custom tabulations are available at a price that reflects the resources required to produce them.

Sampling methods, weighting and estimation
Éric Langlet
Social Survey Methods Division
(613) 951-6967
Eric.Langlet@statcan.ca

Content, collection and data processing
Martine Grenier
Housing, Family and Social Statistics Division
(613) 951-6008
Martine.Grenier@statcan.ca

Client services and Dissemination
Housing, Family and Social Statistics Division
(613) 951-5979
(fax) 951-0387
hfsslf@statcan.ca

APPENDIX A

RULES FOR CALCULATING APPROXIMATE VARIANCE FOR THE 2000 PALS PUBLIC USE MICRO-DATA FILE[1]

The rules that we are about to discuss should enable users to calculate approximate coefficients of variation using the Excel file "Tableaux de CV PALS (FMGD) - CV Tables PALS (PUMF).xls" for estimating the proportion or the percentage of observed population units that possess particular characteristics, as well as for ratios and differences between estimates.

Quality rules

The same quality rules that were applied for different PALS data releases have been applied here. Accordingly, any cell containing 10 or fewer (unweighted) individuals is deleted. Zeroes thus replace the results in all of the table's "results" columns. We have also adopted a colour code for estimated CVs:

Yellow	For CVs ranging from 16.5% to 33.3%
Red	For CVs over 33.3%

It is important to note that certain simulated proportions are relatively far from the target proportion. In most cases, this situation is due to the small number of observations in the cell in question. For that reason, it is quite likely that all simulated proportions for this particular domain are far from the target value and their corresponding CVs appear in red.

The table's different columns appear in tables 1 and 2.

[1]. This document accompanies the file Tableaux de CV EPLA (FMGD) - CV Tables PALS (PUMF).xls

Table 1. Parameters to be specified in defining estimation domains

PARAMETERS TO BE SPECIFIED	POSSIBLE VALUES		MARGINAL
Age groups	15-24	15-19	TOTAL (15 years and older)
		20-24	
	25-44	25-29	
		30-34	
		35-39	
		40-44	
	45-64	45-49	
		50-54	
		55-59	
		60-64	
	65-74	65-69	
		70-74	
	75 and older	75-79	
		80-84	
		85 and over	
Sex	Male or female		TOTAL (Male and female)
Severity of disability	Slight, Moderate, Severe or Very Severe		TOTAL (All degrees of severity)
Type of disability	Hearing, Sight, Speech, Mobility, Agility, Pain or Other		Don't know (No type of disability specified. Applies to all individuals in the file)
Flag	Presence (1) or absence (0) of the disability specified under "type of disability."		Don't know (If "Don't know" is specified under "type of disability," the Flag is automatically set to "Don't know")
Target P	Proportion targeted during simulation (1%, 5%, 10%, 15%, 20%, 25%, 30%, 35%, 40% or 50%). Only used as benchmark. Use Simulated P instead, for analyses.		

Table 2. Estimates provided by the table

RESULTS	MEANING
Simulated P	Actual proportion obtained during simulation. Should be fairly close to Target P. Use this proportion, rather than Target P, in analyses.
n	Number of individuals in the sample (unweighted)
N	Number of individuals in the population (weighted)
CV	Approximate estimated coefficient of variation using the bootstrap
INF	Lower limit of the 95% confidence interval for the simulated proportion
SUP	Upper limit of the 95% confidence interval for the simulated population

Use

The next section provides explanations and examples which respond to the questions that are most commonly answered during data analysis, which are:

1. How is the CV of an estimate determined (as a proportion or as a percentage)?
2. Is the observed difference between the two estimates statistically significant?
3. How can a CV be obtained if the estimate is greater than 50%?
4. How can a CV be obtained if only one subgroup of the population responds to a certain question?

The following screen appears when the Excel file is opened Tableaux de CV PALS (FMGD) - CV Tables PALS (PUMF).xls

A	B	C	D	E	F	G	H	I	J	K	L
Groupes d'âge Age groups	Sexe Sex	Gravité de l'incap. Severity of disab.	Type d'incapacité Type of disability	Flag	P cible Target P	P simulée Simulated P	n	N	CV	INF	SUP
TOTAL	TOTAL	TOTAL	NSP	NSP	1%	1.009652331	20710	3420340	12.968	0.751787	1.267517
TOTAL	TOTAL	TOTAL	NSP	NSP	5%	5.044980465	20710	3420340	5.691	4.48162	5.608341
TOTAL	TOTAL	TOTAL	NSP	NSP	10%	9.944165607	20710	3420340	3.873	9.189167	10.69916
TOTAL	TOTAL	TOTAL	NSP	NSP	15%	14.89764421	20710	3420340	3.056	14.00472	15.79057
TOTAL	TOTAL	TOTAL	NSP	NSP	20%	19.93561802	20710	3420340	2.6	18.91972	20.95151
TOTAL	TOTAL	TOTAL	NSP	NSP	25%	24.88143281	20710	3420340	2.262	23.7784	25.98447

1. How can the CV of an estimate be determined (proportion or percentage)?

a) Click on the drop-down menu button 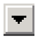 in the "Age group" column and select the age group for which you want estimates. This action filters your data so that only those lines of the table that contain estimates for the specified age group are considered. If you are not seeking information on a particular age group, select "(all)," to list all age groups or select "TOTAL" to only retain overall estimates for ages 15 and above.

b) Perform the same actions as above with the "Sex", "Severity of Disability," Type of Disability" and "Flag" columns.

c) Use the "Target P" button to select the proportion you want. If, for example, you want to obtain a CV for a proportion of 23% that does not appear on the list, select "(all)" from the menu to retain all proportions. In this manner, by using those CVs that correspond to proportions of 20% and 25% for the same domain, we know that the desired CV is situated within these two limits.

d) Results are presented in the last six columns of the table.

2. Is the observed difference between the two estimates statistically significant?

As appears in the table, the lower (INF) and upper (SUP) limits of the 95% confidence interval correspond with each simulated proportion. Once these limits have been identified, it is relatively simple to determine if the observed difference between two estimates is statistically significant. This method involves determining if the confidence intervals of each simulated proportion do or do not overlap. If they do, we cannot be certain that both estimates are statistically different. Conversely, if no overlap is present, we can state that there is a statistically significant difference between both estimates.

3. How is a CV obtained if the estimate is greater than 50%?

A brief reminder is warranted. We use the following formula for calculating a coefficient of variation:

$$CV = \frac{Standard\ error}{Estimation} \times 100$$

The table gives us the CV and the estimate (the proportion). The standard error was estimated using the bootstrap, and is the square root of the variance.

Let us assume that we are interested in a proportion greater than 50% in a particular domain. It appears that no CV has been calculated for proportions greater than 50%. But

the desired CV can be easily calculated using the supplementary proportion. Here is how to do it:

- We want the CV of proportion B, which is greater than 50%.
- We use the CV of the complementary proportion A, for which A=100-B
- We must work **in the same domain** for proportions A and B.
- We thus have:

$$CV_A = \frac{Standard\ error_A}{Estimation_A} \times 100$$

- We must isolate the standard error from the formula and calculate the standard error based on the CV and the estimate in the table.

$$Standard\ error_A = \frac{CV_A \times Estimation_A}{100}$$

- As the **standard error for A is the same as it is for its complement B**, we can find the CV of B simply by using the original formula:

$$CV_B = \frac{Standard\ error_A}{Estimation_B} \times 100$$

Example

A = 40% (proportion of people **with** a certain characteristic)	CV_A =15,5%
B = 60% (proportion of people **without** this characteristic)	CV_B = ?

$$Standard\ error_A = \frac{15{,}5 \times 40}{100} = 6{,}2$$

$$CV_B = \frac{6{,}2}{60} \times 100 = 10{,}3\%$$

4. How can a CV be calculated if only one subgroup of the population responds to a question?

This scenario differs from its predecessors to the extent that respondents have been previously distinguished from the rest of the population on the basis of a particular characteristic. For example, let us assume we are interested in the CV for that percentage of respondents who reported that they have "much difficulty speaking" (B31). To calculate the CV, we must use the proportion that these respondents represent with

respect to the set of respondents rather than what they represent within the subgroup which reported that it had "trouble speaking due to a health situation or problem" (B29). Naturally, we must distinguish between these two different estimation domains if we want to obtain the CV for a subgroup of the population. Ultimately, we must ensure that the denominator of our proportion clearly corresponds with the N value appearing in the table.

APPENDIX B

Survey Questionnaire

Participation and Activity Limitation Survey – 2001 (Adults – 15 and over)

FORM 02

Collected under the authority of the *Statistics Act*. Statutes of Canada, 1985, Chapter S19.

INTRODUCTION

Statistics Canada is conducting a survey on Canadians whose day-to-day activities may be limited because of a condition or health problem. Survey results will help to identify difficulties and barriers these Canadians may face. To reduce the number of questions we need to ask, the Census information collected last May will be added to the information provided in this interview. All information will be kept confidential and used for statistical purposes only. While your participation is voluntary, your assistance is very important to ensure that the results are accurate.

CONFIDENTIAL WHEN COMPLETED

First name(s) ______ Initial(s) ______ Family name ______

Sex: Male ☐ Female ☐

Date of Birth: Year ☐☐☐☐ Month ☐☐ Day ☐☐

Telephone: Area code ☐☐☐ Telephone No. ☐☐☐ — ☐☐☐☐

Address:

Number and Street or lot and concession or exact location ______ Apt. No. ☐☐☐☐

City, Town, Village or Municipality ______ Province or Territory ______ Postal Code ☐☐☐ ☐☐☐

Number of residents in the household: Adults (15 and over) ☐☐ Children (0-14) ☐☐

INFORMATION SOURCE

Source:

(1) Respondent .... ○

(2) Respondent (via interpreter) ... ○

(3) Proxy .... ○

Proxy Information

Relationship to respondent:

(1) Parent .... ○

(2) Guardian .... ○

(3) Child .... ○

(4) Other household member ... ○

(5) Other, specify .... ○ ______

Reason for proxy:

(1) Does not speak English or French .... ○

(2) Unable to respond .... ○

(3) Absent – duration of survey.... ○

(4) Parent wishes to respond for child (15 or older)......... ○

Proxy name:

First name(s) ______

Family name ______

Statistics Canada Statistique Canada

Canada

SECTION A — FILTER QUESTIONS

A1. Do you (Does . . . . .) have any DIFFICULTY hearing, seeing, communicating, walking, climbing stairs, bending, learning or doing any similar activities?

Interviewer: Read list.

(1) Yes, sometimes . . . . ◯
(2) Yes, often . . . . ◯ ▶ Check box General – Limitation on Profile Sheet
(3) No . . . . ◯
(x) Don't know . . . . ◯
(r) Refusal . . . . ◯

A2A. Does a physical condition OR mental condition OR health problem REDUCE THE AMOUNT OR THE KIND OF ACTIVITY you (. . . . .) can do at home?

Interviewer: Read list.

(1) Yes, sometimes . . . . ◯
(2) Yes, often . . . . ◯ ▶ Check box General – Limitation on Profile Sheet
(3) No . . . . ◯
(x) Don't know . . . . ◯
(r) Refusal . . . . ◯

A2B. Does a physical condition OR mental condition OR health problem REDUCE THE AMOUNT OR THE KIND OF ACTIVITY you (. . . . .) can do at work or at school?

Interviewer: Read list.

(1) Yes, sometimes . . . . ◯
(2) Yes, often . . . . ◯ ▶ Check box General – Limitation on Profile Sheet
(3) No . . . . ◯
(5) Not applicable . . . . . ◯
(x) Don't know . . . . ◯
(r) Refusal . . . . ◯

A2C. Does a physical condition OR mental condition OR health problem REDUCE THE AMOUNT OR THE KIND OF ACTIVITY you (. . . . .) can do in other activities, for example, transportation or leisure?

Interviewer: Read list.

(1) Yes, sometimes . . . . ◯
(2) Yes, often . . . . ◯ ▶ Check box General – Limitation on Profile Sheet
(3) No . . . . ◯
(x) Don't know . . . . ◯
(r) Refusal . . . . ◯

SECTION B — ACTIVITY LIMITATIONS

B1. I am going to ask you a series of questions about your (. . . . . 's) ability to do certain activities. Please tell me only about those difficulties that have lasted, or are expected to last, six months or more.

Do you (Does . . . . .) use a hearing aid or hearing aids?

(1) Yes . . . . ◯
(3) No . . . . ◯
(x) Don't know . . . . ◯ ▶ **Go to B5**
(r) Refusal . . . . ◯

B2. **WITH your (his/her) hearing aid(s), how much difficulty do you (does . . . . .) have hearing what is said in a conversation with ONE other person?**

Interviewer: Read list. Mark one only.

(1) No difficulty . . . . ◯
(2) Some difficulty . . . . ◯
(3) A lot of difficulty . . . . ◯
(4) You (. . . . .) cannot hear . . . . ◯
▶ Check box Hearing – Limitation on Profile Sheet (for 2–4)

(x) Don't know . . . . ◯
(r) Refusal . . . . ◯

B3. **WITH your (his/her) hearing aid(s), how much difficulty do you (does . . . . .) have hearing what is said in a conversation with at least THREE other persons?**

Interviewer: Read list. Mark one only.

(1) No difficulty . . . . ◯
(2) Some difficulty . . . . ◯
(3) A lot of difficulty . . . . ◯
(4) You (. . . . .) cannot hear . . . . ◯
▶ Check box Hearing – Limitation on Profile Sheet (for 2–4)

(x) Don't know . . . . ◯
(r) Refusal . . . . ◯

B4. **WITH your (his/her) hearing aid(s), how much difficulty do you (does . . . . .) have hearing what is said in a TELEPHONE conversation?**

Interviewer: Read list. Mark one only.

(1) No difficulty . . . . ◯
(2) Some difficulty . . . . ◯
(3) A lot of difficulty . . . . ◯
(4) You (. . . . .) cannot hear . . . . ◯
▶ Check box Hearing – Limitation on Profile Sheet (for 2–4)

(x) Don't know . . . . ◯
(r) Refusal . . . . ◯

B4. edit

Interviewer: If box Hearing – Limitation is checked on Profile Sheet, go to B9. Otherwise, go to B14.

B5. **Which of the following best describes your (. . . . .'s) ability to hear?**

Interviewer: Read list. Mark one only.

(1) You (. . . . .) cannot hear . . . . ◯ → Check box Hearing – Limitation on Profile Sheet **Go to B9**
(2) You have (. . . . . has) difficulty hearing . . . . ◯
(3) You have (. . . . . has) no problem hearing . . . . ◯ → **Go to B14**

(x) Don't know . . . . ◯
(r) Refusal . . . . ◯ → **Go to B14**

B6. **How much difficulty do you (does . . . . .) have hearing what is said in a conversation with ONE other person?**

Interviewer: Read list. Mark one only.

(1) No difficulty . . . . ◯
(2) Some difficulty . . . . ◯
(3) A lot of difficulty . . . . ◯
(4) You (. . . . .) cannot hear . . . . ◯
▶ Check box Hearing – Limitation on Profile Sheet (for 2–4)

(x) Don't know . . . . ◯
(r) Refusal . . . . ◯

B7. How much difficulty do you (does . . . . .) have hearing what is said in a conversation with at least THREE other persons?

Interviewer: Read list. Mark one only.

(1) No difficulty . . . . ○
(2) Some difficulty . . . . ○
(3) A lot of difficulty . . . . ○
(4) You (. . . . .) cannot hear . . . . ○
▶ Check box Hearing – Limitation on Profile Sheet

(x) Don't know . . . . ○
(r) Refusal . . . . ○

B8. How much difficulty do you (does . . . . .) have hearing what is said in a TELEPHONE conversation?

Interviewer: Read list. Mark one only.

(1) No difficulty . . . . ○
(2) Some difficulty . . . . ○
(3) A lot of difficulty . . . . ○
(4) You (. . . . .) cannot hear . . . . ○
▶ Check box Hearing – Limitation on Profile Sheet

(x) Don't know . . . . ○
(r) Refusal . . . . ○

B9. edit

Interviewer: If box Hearing – Limitation is checked on Profile Sheet, continue. Otherwise, go to B14.

B9. Besides hearing aids, do you (does . . . . .) USE any other aids, specialized equipment or services for persons who are deaf or hard of hearing, for example, a volume control telephone or TV decoder?

(1) Yes . . . . ○ → Check box Hearing – USE aid on Profile Sheet
(3) No . . . . ○
(x) Don't know . . . . ○
(r) Refusal . . . . ○
▶ **Go to B11**

B10. Do you (Does . . . . .) now USE:

Interviewer: Read list.

	(1) Yes	(3) No	(x) DK	(r) Ref
(a) a computer to communicate, e.g., e-mail or chat service?	○	○	○	○
(b) a volume control telephone?	○	○	○	○
(c) a TTY or TDD?	○	○	○	○
(d) a message relay service?	○	○	○	○
(e) other phone related devices, e.g., flashers?	○	○	○	○
(f) a closed caption T.V. or decoder?	○	○	○	○
(g) amplifiers, e.g., FM, acoustic, infrared?	○	○	○	○
(h) visual or vibrating alarms?	○	○	○	○
(i) a Sign language interpreter?	○	○	○	○
(j) a hearing ear dog?	○	○	○	○
(k) another aid? — specify	○ ↓	○	○	○

[ ]

B11. Are there any aids, specialized equipment or services for persons who are deaf or hard of hearing that you think you NEED (. . . . . thinks he/she NEEDS) but do (does) not have?

(1) Yes . . . . ○ → Check box Hearing – NEED aid on Profile Sheet
(3) No . . . . ○
(x) Don't know . . . . ○
(r) Refusal . . . . ○
▶ **Go to B13**

B12. **Which aids do you (does . . . . .) NEED but do (does) not have?**

Interviewer: Do not read list. Mark all that apply.

- (a) Computer to communicate (e.g., e-mail or chat service) . . . . ☐
- (b) Volume control telephone . . . . ☐
- (c) TTY or TDD . . . . ☐
- (d) Message relay service . . . . ☐
- (e) Other phone related devices (e.g., flashers) . . . . ☐
- (f) Closed caption T.V. or decoder . . . . ☐
- (g) Amplifiers (e.g., FM, acoustic, infrared) . . . . ☐
- (h) Visual or vibrating alarms . . . . ☐
- (i) Sign language interpreter . . . . ☐
- (j) Hearing ear dog . . . . ☐
- (k) Other, specify . . . . ☐ ↓

[ ]

- (x) Don't know . . . . ☐
- (r) Refusal . . . . ☐

B13. **This question deals with certain communication skills. I will read you a list. Please answer Yes or No to each.**

**Do you (Does . . . . .) . . .**	(1) Yes	(3) No	(x) DK	(r) Ref
(a) use Sign language such as ASL or LSQ?	◯	◯	◯	◯
(b) speech read or lip read?	◯	◯	◯	◯

B14. **Do you (Does . . . . .) wear glasses or contact lenses to see up close?**

- (1) Yes . . . . ◯
- (3) No . . . . ◯ ▶ **Go to B17**
- (x) Don't know . . . . ◯ ▶ **Go to B17**
- (r) Refusal . . . . ◯ → **Go to B29**

B15. **WITH your (his/her) glasses or contact lenses, do you (does . . . . .) have any difficulty seeing ordinary newsprint?**

- (1) Yes . . . . ◯ → Check box Seeing – Limitation on Profile Sheet
- (3) No . . . . ◯
- (x) Don't know . . . . ◯
- (r) Refusal . . . . ◯

▶ (3), (x), (r): **Go to B19**

B16. **How much difficulty?**

Interviewer: Read list. Mark one only.

- (1) Some difficulty . . . . ◯
- (2) A lot of difficulty . . . . ◯
- (3) You (. . . . .) cannot see . . . . ◯
- (x) Don't know . . . . ◯
- (r) Refusal . . . . ◯

▶ **Go to B19**

B17. **Do you (Does . . . . .) have any difficulty seeing ordinary newsprint?**

- (1) Yes . . . . ◯ → Check box Seeing – Limitation on Profile Sheet
- (3) No . . . . ◯
- (x) Don't know . . . . ◯
- (r) Refusal . . . . ◯

▶ (3), (x), (r): **Go to B19**

B18. How much difficulty?

Interviewer: Read list. Mark one only.

(1) Some difficulty .... ◯
(2) A lot of difficulty .... ◯
(3) You (.....) cannot see .... ◯

(x) Don't know .... ◯
(r) Refusal .... ◯

B19. Do you (Does .....) wear glasses or contact lenses to see at a distance?

(1) Yes .... ◯
(3) No .... ◯ } **Go to B22**
(x) Don't know .... ◯ } **Go to B22**
(r) Refusal .... ◯ → **Go to B24edit**

B20. WITH your (his/her) glasses or contact lenses, do you (does .....) have any difficulty clearly seeing the face of someone across a room, that is, from 4 metres or 12 feet?

(1) Yes .... ◯ → Check box Seeing – Limitation on Profile Sheet
(3) No .... ◯ } **Go to B24edit**
(x) Don't know .... ◯ } **Go to B24edit**
(r) Refusal .... ◯ } **Go to B24edit**

B21. How much difficulty?

Interviewer: Read list. Mark one only.

(1) Some difficulty .... ◯ } **Go to B24 edit**
(2) A lot of difficulty .... ◯ } **Go to B24 edit**
(3) You (.....) cannot see .... ◯ } **Go to B24 edit**
(x) Don't know .... ◯ } **Go to B24 edit**
(r) Refusal .... ◯ } **Go to B24 edit**

B22. Do you (Does .....) have any difficulty clearly seeing the face of someone across a room, that is, from 4 metres or 12 feet?

(1) Yes .... ◯ → Check box Seeing – Limitation on Profile Sheet
(3) No .... ◯ } **Go to B24edit**
(x) Don't know .... ◯ } **Go to B24edit**
(r) Refusal .... ◯ } **Go to B24edit**

B23. How much difficulty?

Interviewer: Read list. Mark one only.

(1) Some difficulty .... ◯
(2) A lot of difficulty .... ◯
(3) You (.....) cannot see .... ◯

(x) Don't know .... ◯
(r) Refusal .... ◯

B24. edit

Interviewer: If box Seeing – Limitation is checked on Profile Sheet, continue. Otherwise, go to B29.

B24. Have you (Has . . . . .) been diagnosed by an eye specialist as being legally blind?

(1) Yes . . . . ○
(3) No . . . . ○
(x) Don't know or not sure . . . . ○
(r) Refusal . . . . ○

B25. Besides glasses or contact lenses, do you (does . . . . .) USE any other aids or specialized equipment for persons who are blind or visually impaired, for example, magnifiers or Braille reading materials?

(1) Yes . . . . ○ → Check box Seeing – USE aid on Profile Sheet
(3) No . . . . ○
(x) Don't know . . . . ○
(r) Refusal . . . . ○

(No, Don't know, Refusal) **Go to B27**

B26. Do you (Does . . . . .) now USE . . .

	(1) Yes	(3) No	(x) DK	(r) Ref
(a) magnifiers?	○	○	○	○
(b) Braille reading materials?	○	○	○	○
(c) large print reading materials?	○	○	○	○
(d) talking books?	○	○	○	○
(e) recording equipment or portable note-takers?	○	○	○	○
(f) closed circuit devices, e.g., CCTV's?	○	○	○	○
(g) a computer with Braille, large print or speech access?	○	○	○	○
(h) a white cane?	○	○	○	○
(i) a guide dog?	○	○	○	○
(j) another aid? — specify	○	○	○	○

[ ]

B27. Are there any aids or specialized equipment for persons who are blind or visually impaired that you think you NEED (. . . . . thinks he/she NEEDS) but do (does) not have?

(1) Yes . . . . ○ → Check box Seeing – NEED aid on Profile Sheet
(3) No . . . . ○
(x) Don't know . . . . ○
(r) Refusal . . . . ○

(No, Don't know, Refusal) **Go to B29**

B28. Which aids do you (does . . . . .) NEED but do (does) not have?

Interviewer: Do not read list. Mark all that apply.

(a) Glasses, contact lenses, or a new prescription . . . . ☐
(b) Magnifiers . . . . ☐
(c) Braille reading materials . . . . ☐
(d) Large print reading materials . . . . ☐
(e) Talking books . . . . ☐
(f) Recording equipment or portable note-takers . . . . ☐
(g) Closed circuit devices (CCTV's) . . . . ☐
(h) Computer with Braille, large print or speech access . . . . . . ☐
(i) White cane . . . . ☐
(j) Guide dog . . . . ☐
(k) Other, specify . . . . ☐

[ ]

(x) Don't know . . . . ☐
(r) Refusal . . . . ☐

B29. Because of a condition or health problem, do you (does .....) have any difficulty speaking?

(1) Yes ○ → Check box Communicating – Limitation on Profile Sheet **Go to B31**
(3) No ○
(x) Don't know ○
(r) Refusal ○

B30. Because of a condition or health problem, do you (does .....) have any difficulty making yourself (himself/herself) understood when speaking?

(1) Yes ○ → Check box Communicating – Limitation on Profile Sheet **Go to B33**
(3) No ○
(x) Don't know ○
(r) Refusal ○
(3), (x), (r): **Go to B40**

B31. How much difficulty do you (does .....) have speaking?

Interviewer: Read list. Mark one only.

(1) Some difficulty ○
(2) A lot of difficulty ○
(3) You (.....) cannot speak ○ → **Go to B34**
(x) Don't know ○
(r) Refusal ○

B32. Because of a condition or health problem, do you (does .....) have any difficulty making yourself (himself/herself) understood when speaking?

(1) Yes ○
(3) No ○
(x) Don't know ○
(r) Refusal ○
Go to B34

B33. How well are you (is .....) able to make yourself (himself/herself) understood when speaking with ...

Interviewer: Read categories. Mark one only.

Interviewer: Read list.	(1) Completely	(2) Partially	(3) Not at all	(x) DK	(r) Ref
(a) members of your (his/her) own family?	○	○	○	○	○
(b) your (his/her) friends?	○	○	○	○	○
(c) professionals and service providers, e.g., doctors, home care providers?	○	○	○	○	○
(d) other people?	○	○	○	○	○

B34. Do you (Does .....) use:

Interviewer: Read list. Mark one only.

	(1) Yes	(3) No	(x) DK	(r) Ref
(a) Sign language such as ASL or LSQ?	○	○	○	○
(b) another form of communication?	○	○	○	○

B34. edit

Interviewer: If at least one Yes is checked in B34, then continue. Otherwise, go to B36.

B35. How well are you (is . . . . .) able to make yourself (himself/herself) understood when communicating in this manner with . . .

Interviewer: Read categories. Mark one only.

Interviewer: Read list.

	(1) Completely	(2) Partially	(3) Not at all	(x) DK	(r) Ref
(a) members of your (his/her) own family?	◯	◯	◯	◯	◯
(b) your (his/her) friends?	◯	◯	◯	◯	◯
(c) professionals and service providers, e.g., doctors, home care providers?	◯	◯	◯	◯	◯
(d) other people?	◯	◯	◯	◯	◯

B36. Do you (Does . . . . .) USE any aids or specialized equipment for persons who have difficulty speaking or making themselves understood, for example, a keyboard device to communicate?

(1) Yes . . . . ◯ → Check box Communicating – USE aid on Profile Sheet
(3) No . . . . ◯
(x) Don't know . . . . ◯
(r) Refusal . . . . ◯
Go to B38

B37. Do you (Does . . . . .) now USE:

Interviewer: Read list.

	(1) Yes	(3) No	(x) DK	(r) Ref
(a) a voice amplifier?	◯	◯	◯	◯
(b) a computer or keyboard device to communicate?	◯	◯	◯	◯
(c) a communications board such as Bliss?	◯	◯	◯	◯
(d) another aid? — specify	◯	◯	◯	◯

B38. Are there any aids or specialized equipment for persons who have difficulty speaking or making themselves understood that you think you NEED (. . . . . thinks he/she NEEDS) but do (does) not have?

(1) Yes . . . . ◯ → Check box Communicating – NEED aid on Profile Sheet
(3) No . . . . ◯
(x) Don't know . . . . ◯
(r) Refusal . . . . ◯
Go to B40

B39. Which aids do you (does . . . . .) NEED but do (does) not have?

Interviewer: Do not read list. Mark all that apply.

(a) Voice amplifier . . . . ☐
(b) Computer or keyboard device to communicate . . . . . . ☐
(c) Communications board such as Bliss . . . . ☐
(d) Other, specify . . . . ☐

(x) Don't know . . . . ☐
(r) Refusal . . . . ☐

B40. The next few questions are about your (. . . . . 's) ability to move around, even when using an aid or specialized equipment such as a cane or crutches. Remember, I am asking about difficulties that have lasted or are expected to last 6 months or more.

Are you (Is . . . . .) able to walk?

(1) Yes . . . . ◯
(3) No . . . . ◯ → Check box Mobility – Limitation on Profile Sheet **Go to B47**
(x) Don't know . . . . ◯
(r) Refusal . . . . ◯ → **Go to B55**

B41. Do you (Does . . . . .) have any difficulty walking half a kilometre or a quarter mile, that is, about three city blocks, without resting?

Interviewer: Read list. Mark one only.

(1) Yes, sometimes . . . . ◯
(2) Yes, often or always . . . . ◯
▶ Check box Mobility – Limitation on Profile Sheet

(3) No . . . . ◯
(x) Don't know . . . . ◯
(r) Refusal . . . . ◯
▶ **Go to B43**

B42. How much difficulty?

Interviewer: Read list. Mark one only.

(1) Some difficulty . . . . ◯
(2) A lot of difficulty . . . . ◯
(3) Completely unable . . . . . ◯
(x) Don't know . . . . ◯
(r) Refusal . . . . ◯

B43. Do you (Does . . . . .) have any difficulty walking up and down a flight of stairs, about 12 steps, without resting?

Interviewer: Read list. Mark one only.

(1) Yes, sometimes . . . . ◯
(2) Yes, often or always . . . . ◯
▶ Check box Mobility – Limitation on Profile Sheet

(3) No . . . . ◯
(x) Don't know . . . . ◯
(r) Refusal . . . . ◯
▶ **Go to B45**

B44. How much difficulty?

Interviewer: Read list. Mark one only.

(1) Some difficulty . . . . ◯
(2) A lot of difficulty . . . . ◯
(3) Completely unable . . . . . ◯
(x) Don't know . . . . ◯
(r) Refusal . . . . ◯

B45. Do you (Does . . . . .) have any difficulty carrying an object of 5 kg or 10 pounds, like a bag of groceries, for 10 metres or 30 feet?

Interviewer: Read list. Mark one only.

(1) Yes, sometimes . . . . ◯
(2) Yes, often or always . . . . ◯
▶ Check box Mobility – Limitation on Profile Sheet

(3) No . . . . ◯
(x) Don't know . . . . ◯
(r) Refusal . . . . ◯
▶ **Go to B47**

B46. How much difficulty?

Interviewer: Read list. Mark one only.

(1) Some difficulty .... ○
(2) A lot of difficulty .... ○
(3) Completely unable ..... ○
(x) Don't know .... ○
(r) Refusal .... ○

B47. Do you (Does .....) have any difficulty standing in line for more than 20 minutes?

Interviewer: Read list. Mark one only.

(1) Yes, sometimes .... ○
(2) Yes, often or always .... ○
▶ Check box Mobility – Limitation on Profile Sheet

(3) No .... ○
(x) Don't know .... ○
(r) Refusal .... ○
▶ **Go to B49**

B48. How much difficulty?

Interviewer: Read list. Mark one only.

(1) Some difficulty .... ○
(2) A lot of difficulty .... ○
(3) Completely unable ..... ○
(x) Don't know .... ○
(r) Refusal .... ○

B49. Do you (Does .....) have any difficulty moving from one room to another?

Interviewer: Read list. Mark one only.

(1) Yes, sometimes .... ○
(2) Yes, often or always .... ○
▶ Check box Mobility – Limitation on Profile Sheet

(3) No .... ○
(x) Don't know .... ○
(r) Refusal .... ○
▶ **Go to B51edit**

B50. How much difficulty?

Interviewer: Read list. Mark one only.

(1) Some difficulty .... ○
(2) A lot of difficulty .... ○
(3) Completely unable ..... ○
(x) Don't know .... ○
(r) Refusal .... ○

B51. edit

Interviewer: If box Mobility – Limitation is checked on Profile Sheet, continue. Otherwise, go to B55.

B51. Do you (Does .....) USE any aids or specialized equipment for persons who have difficulty moving around?

(1) Yes .... ○ → Check box Mobility – USE aid on Profile Sheet
(3) No .... ○
(x) Don't know .... ○
(r) Refusal .... ○
▶ **Go to B53**

B52. Do you (Does . . . . .) now USE . . .

Interviewer: Read list.

	(1) Yes	(3) No	(x) DK	(r) Ref
(a) orthopaedic footwear?	◯	◯	◯	◯
(b) a cane or walking stick?	◯	◯	◯	◯
(c) crutches?	◯	◯	◯	◯
(d) a manual wheelchair?	◯	◯	◯	◯
(e) an electric wheelchair?	◯	◯	◯	◯
(f) a walker?	◯	◯	◯	◯
(g) a scooter?	◯	◯	◯	◯
(h) braces or supportive devices?	◯	◯	◯	◯
(i) lifts or lift type devices?	◯	◯	◯	◯
(j) grab bars or bathroom aids?	◯	◯	◯	◯
(k) another aid? — specify	◯ →	◯	◯	◯

[ ]

B53. Are there any aids or specialized equipment for persons who have difficulty moving around that you think you NEED (. . . . . thinks he/she NEEDS) but do (does) not have?

(1) Yes ◯ → Check box Mobility – NEED aid on Profile Sheet
(3) No ◯
(x) Don't know ◯
(r) Refusal ◯
→ **Go to B55** (for No, Don't know, Refusal)

B54. Which aids do you (does . . . . .) NEED but do (does) not have?

Interviewer: Do not read list. Mark all that apply.

(a) Orthopaedic footwear ☐
(b) Cane or walking stick ☐
(c) Crutches ☐
(d) Manual wheelchair ☐
(e) Electric wheelchair ☐
(f) Walker ☐
(g) Scooter ☐
(h) Braces or supportive devices ☐
(i) Lifts or lift type devices ☐
(j) Grab bars or bathroom aids ☐
(k) Other, specify ☐ →

[ ]

(x) Don't know ☐
(r) Refusal ☐

B55. The next questions deal with flexibility and agility. Remember, I am asking about difficulties that have lasted or are expected to last 6 months or more.

Do you (Does . . . . .) have any difficulty bending down and picking up an object from the floor (for example, a shoe)?

Interviewer: Read list. Mark one only.

(1) Yes, sometimes ◯
(2) Yes, often or always ◯
→ Check box Agility – Limitation on Profile Sheet
(3) No ◯
(x) Don't know ◯
(r) Refusal ◯
→ **Go to B57**

B56. How much difficulty?

Interviewer: Read list. Mark one only.

(1) Some difficulty . . . . ◯
(2) A lot of difficulty . . . . ◯
(3) Completely unable . . . . . ◯
(x) Don't know . . . . ◯
(r) Refusal . . . . ◯

B57. Do you (Does . . . . .) have any difficulty dressing and undressing yourself (himself/herself)?

Interviewer: Read list. Mark one only.

(1) Yes, sometimes . . . . ◯
(2) Yes, often or always . . . . ◯ ▶ Check box Agility – Limitation on Profile Sheet
(3) No . . . . ◯
(x) Don't know . . . . ◯
(r) Refusal . . . . ◯ ▶ **Go to B59**

B58. How much difficulty?

Interviewer: Read list. Mark one only.

(1) Some difficulty . . . . ◯
(2) A lot of difficulty . . . . ◯
(3) Completely unable . . . . . ◯
(x) Don't know . . . . ◯
(r) Refusal . . . . ◯

B59. Do you (Does . . . . .) have any difficulty getting into and out of bed?

Interviewer: Read list. Mark one only.

(1) Yes, sometimes . . . . ◯
(2) Yes, often or always . . . . ◯ ▶ Check box Agility – Limitation on Profile Sheet
(3) No . . . . ◯
(x) Don't know . . . . ◯
(r) Refusal . . . . ◯ ▶ **Go to B61**

B60. How much difficulty?

Interviewer: Read list. Mark one only.

(1) Some difficulty . . . . ◯
(2) A lot of difficulty . . . . ◯
(3) Completely unable . . . . . ◯
(x) Don't know . . . . ◯
(r) Refusal . . . . ◯

B61. Is it physically difficult for you (. . . . .) to cut your (his/her) own toenails?

Interviewer: Read list. Mark one only.

(1) Yes, sometimes . . . . ◯
(2) Yes, often or always . . . . ◯ ▶ Check box Agility – Limitation on Profile Sheet
(3) No . . . . ◯
(x) Don't know . . . . ◯
(r) Refusal . . . . ◯ ▶ **Go to B63**

B62. How much difficulty do you (does . . . . .) have doing this?

Interviewer: Read list. Mark one only.

(1) Some difficulty . . . . ◯
(2) A lot of difficulty . . . . ◯
(3) Completely unable . . . . . ◯
(x) Don't know . . . . ◯
(r) Refusal . . . . ◯

B63. Do you (Does . . . . .) have any difficulty using your (his/her) fingers to grasp or to handle an object, such as pliers or scissors?

Interviewer: Read list. Mark one only.

(1) Yes, sometimes . . . . ◯
(2) Yes, often or always . . . . ◯
▶ Check box Agility – Limitation on Profile Sheet

(3) No . . . . ◯
(x) Don't know . . . . ◯
(r) Refusal . . . . ◯
▶ **Go to B65**

B64. How much difficulty?

Interviewer: Read list. Mark one only.

(1) Some difficulty . . . . ◯
(2) A lot of difficulty . . . . ◯
(3) Completely unable . . . . . ◯
(x) Don't know . . . . ◯
(r) Refusal . . . . ◯

B65. Do you (Does . . . . .) have any difficulty reaching in any direction (for example, above your (his/her) head)?

Interviewer: Read list. Mark one only.

(1) Yes, sometimes . . . . ◯
(2) Yes, often or always . . . . ◯
▶ Check box Agility – Limitation on Profile Sheet

(3) No . . . . ◯
(x) Don't know . . . . ◯
(r) Refusal . . . . ◯
▶ **Go to B67**

B66. How much difficulty?

Interviewer: Read list. Mark one only.

(1) Some difficulty . . . . ◯
(2) A lot of difficulty . . . . ◯
(3) Completely unable . . . . . ◯
(x) Don't know . . . . ◯
(r) Refusal . . . . ◯

B67. Do you (Does . . . . .) have any difficulty cutting your (his/her) own food?

Interviewer: Read list. Mark one only.

(1) Yes, sometimes . . . . ◯
(2) Yes, often or always . . . . ◯
▶ Check box Agility – Limitation on Profile Sheet

(3) No . . . . ◯
(x) Don't know . . . . ◯
(r) Refusal . . . . ◯
▶ **Go to B69edit**

B68. How much difficulty?

Interviewer: Read list. Mark one only.

(1) Some difficulty ○
(2) A lot of difficulty ○
(3) Completely unable ○

(x) Don't know ○
(r) Refusal ○

B69. edit

Interviewer: If box Agility – Limitation is checked on Profile Sheet, then continue. Otherwise, go to B73.

B69. Do you (Does .....) USE any aids or specialized equipment designed to support, replace or assist in the use of hands or arms?

(1) Yes ○ → Check box Agility – USE aid on Profile Sheet
(3) No ○
(x) Don't know ○
(r) Refusal ○
(3), (x), (r): **Go to B71**

B70. Do you (Does .....) now USE:

Interviewer: Read list.

	(1) Yes	(3) No	(x) DK	(r) Ref
(a) a hand or arm brace?	○	○	○	○
(b) grasping tools or reach extenders?	○	○	○	○
(c) another aid? — specify	○	○	○	○

(c) Yes → specify: ______________________

B71. Are there any aids or specialized equipment designed to support, replace or assist in the use of hands or arms that you think you NEED (..... thinks he/she NEEDS) but do (does) not have?

(1) Yes ○ → Check box Agility – NEED aid on Profile Sheet
(3) No ○
(x) Don't know ○
(r) Refusal ○
(3), (x), (r): **Go to B73**

B72. Which aids do you (does .....) NEED but do (does) not have?

Interviewer: Do not read list. Mark all that apply.

(a) Hand or arm brace ☐
(b) Grasping tools or reach extenders ☐
(c) Other, specify ☐ → ______________________

(x) Don't know ☐
(r) Refusal ☐

B73. The next few questions deal with long-term pain and discomfort.

Do you (Does .....) have any pain or discomfort that is ALWAYS present?

(1) Yes ○ → **Go to B75**
(3) No ○

(x) Don't know ○
(r) Refusal ○

B74. Do you (Does . . . . .) have PERIODS of pain or discomfort that REOCCUR from time to time?

(1) Yes . . . . ◯
(3) No . . . . ◯ ▶ **Go to B77**
(x) Don't know . . . . ◯ ▶ **Go to B77**
(r) Refusal . . . . ◯ ▶ **Go to B77**

B75. Does this pain or discomfort reduce the amount or the kind of activities you (. . . . .) can do?

Interviewer: Read list. Mark one only.

(1) Yes, sometimes . . . . ◯ ▶ Check box Pain – Limitation on Profile Sheet
(2) Yes, often or always . . . . ◯ ▶ Check box Pain – Limitation on Profile Sheet
(3) No . . . . ◯ ▶ **Go to B77**
(x) Don't know . . . . ◯ ▶ **Go to B77**
(r) Refusal . . . . ◯ ▶ **Go to B77**

B76. How many activities does your (. . . . . 's) pain or discomfort usually prevent you (him/her) from doing . . .

Interviewer: Read categories. Mark one only.

Interviewer: Read list.	(1) None	(2) A few	(3) Many	(4) Most	(5) N/A	(x) DK	(r) Ref
(a) at home?	◯	◯	◯	◯		◯	◯
(b) at work?	◯	◯	◯	◯	◯	◯	◯
(c) at school?	◯	◯	◯	◯	◯	◯	◯
(d) in other areas, such as transportation or leisure?	◯	◯	◯	◯	◯	◯	◯

B77. Do you think you have (Does . . . . . think he/she has) a condition that makes it difficult in general for you (him/her) to learn? Such conditions include attention problems, hyperactivity, dyslexia and others.

(1) Yes . . . . ◯ → Check box Learning – Limitation on Profile Sheet
(3) No . . . . ◯
(x) Don't know . . . . ◯
(r) Refusal . . . . ◯

B78. Has a teacher, doctor or other health professional ever said that you (. . . . .) had a learning disability?

(1) Yes . . . . ◯ → Check box Learning – Limitation on Profile Sheet
(3 No . . . . ◯
(x) Don't know . . . . ◯
(r) Refusal . . . . ◯

B78. edit

Interviewer: If B77 is Yes OR B78 is Yes, then continue. Otherwise, go to B85.

B79. Does this condition reduce the amount or the kind of activities you (. . . . .) can do?

Interviewer: Read list. Mark one only.

(1) Yes, sometimes . . . . ◯
(2) Yes, often or always . . . . ◯
(3) No . . . . ◯ ▶ **Go to B81**
(x) Don't know . . . . ◯ ▶ **Go to B81**
(r) Refusal . . . . ◯ ▶ **Go to B81**

B80. How many activities does this condition usually prevent you (. . . . .) from doing . . .

Interviewer: Read categories. Mark one only.

Interviewer: Read list.

	(1) None	(2) A few	(3) Many	(4) Most	(5) N/A	(x) DK	(r) Ref
(a) at home?	◯	◯	◯	◯		◯	◯
(b) at work?	◯	◯	◯	◯	◯	◯	◯
(c) at school?	◯	◯	◯	◯	◯	◯	◯
(d) in other areas, such as transportation or leisure?	◯	◯	◯	◯	◯	◯	◯

B81. Do you (Does . . . . .) USE any aids or specialized equipment to help you (him/her) with your (his/her) learning difficulty?

(1) Yes . . . . ◯ → Check box Learning – USE aid on Profile Sheet
(3) No . . . . ◯
(x) Don't know . . . . ◯
(r) Refusal . . . . ◯
Go to B83

B82. Do you (Does . . . . .) now USE . . .

Interviewer: Read list.

	(1) Yes	(3) No	(x) DK	(r) Ref
(a) portable spell checkers?	◯	◯	◯	◯
(b) recording equipment?	◯	◯	◯	◯
(c) talking books?	◯	◯	◯	◯
(d) pocket organizers?	◯	◯	◯	◯
(e) a home computer?	◯	◯	◯	◯

Interviewer: If (e) is Yes, ask (f) – (i).

	(1) Yes	(3) No	(x) DK	(r) Ref
(f) a scanner or printer?	◯	◯	◯	◯
(g) spell/grammar checking software?	◯	◯	◯	◯
(h) voice recognition software?	◯	◯	◯	◯
(i) software organizational tools?	◯	◯	◯	◯
(j) another aid? — specify	◯	◯	◯	◯

B83. Are there any learning aids that you think you NEED (. . . . . thinks he/she NEEDS) but do (does) not have?

(1) Yes . . . . ◯ → Check box Learning – NEED aid on Profile Sheet
(3) No . . . . ◯
(x) Don't know . . . . ◯
(r) Refusal . . . . ◯
Go to B85

B84. Which aids do you (does . . . . .) NEED but do (does) not have?

Interviewer: Do not read list. Mark all that apply.

(a) Portable spell checkers . . . . ☐
(b) Recording equipment . . . . ☐
(c) Talking books . . . . ☐
(d) Pocket organizers . . . . ☐
(e) Home computer . . . . ☐
(f) Scanner or printer . . . . ☐
(g) Spell/grammar checking software . . . . . ☐
(h) Voice recognition software . . . . ☐
(i) Software organizational tools . . . . ☐
(j) Other, specify . . . . ☐ →

(x) Don't know . . . . ☐
(r) Refusal . . . . ☐

B85. Do you (Does . . . . .) FREQUENTLY have periods of confusion or difficulty remembering things? These difficulties are often associated with diseases such as Alzheimer's or may be the result of a brain injury.

(1) Yes . . . . ○
(3) No . . . . ○ ▶ **Go to B88**
(x) Don't know . . . . ○ ▶ **Go to B88**
(r) Refusal . . . . ○ ▶ **Go to B88**

B86. Does this condition reduce the amount or the kind of activities you (. . . . .) can do?

Interviewer: Read list. Mark one only.

(1) Yes, sometimes . . . . ○ ▶ Check box Confusion/Memory – Limitation on Profile Sheet
(2) Yes, often or always . . . . ○ ▶ Check box Confusion/Memory – Limitation on Profile Sheet
(3) No . . . . ○ ▶ **Go to B88**
(x) Don't know . . . . ○ ▶ **Go to B88**
(r) Refusal . . . . ○ ▶ **Go to B88**

B87. How many activities does this condition usually prevent you (. . . . .) from doing . . .

Interviewer: Read categories. Mark one only.

Interviewer: Read list.	(1) None	(2) A few	(3) Many	(4) Most	(5) N/A	(x) DK	(r) Ref
(a) at home?	○	○	○	○		○	○
(b) at work?	○	○	○	○	○	○	○
(c) at school?	○	○	○	○	○	○	○
(d) in other areas, such as transportation or leisure?	○	○	○	○	○	○	○

B88. Has a doctor, psychologist or other health professional ever said that you (. . . . .) had a developmental disability or disorder? These include, for example, Down syndrome, autism, Asperger syndrome, mental impairment due to a lack of oxygen at birth, etc..

(1) Yes . . . . ○ → Check box Developmental – Limitation on Profile Sheet
(3) No . . . . ○ ▶ **Go to B91**
(x) Don't know . . . . ○ ▶ **Go to B91**
(r) Refusal . . . . ○ ▶ **Go to B91**

B89. Does this condition reduce the amount or the kind of activities you (. . . . .) can do?

Interviewer: Read list. Mark one only.

(1) Yes, sometimes . . . . ○
(2) Yes, often or always . . . . ○
(3) No . . . . ○
(x) Don't know . . . . ○
(r) Refusal . . . . ○

(3), (x), (r) ► **Go to B91**

B90. How many activities does this condition usually prevent you (. . . . .) from doing . . .

Interviewer: Read categories. Mark one only.

Interviewer: Read list.	(1) None	(2) A few	(3) Many	(4) Most	(5) N/A	(x) DK	(r) Ref
(a) at home?	○	○	○	○		○	○
(b) at work?	○	○	○	○	○	○	○
(c) at school?	○	○	○	○	○	○	○
(d) in other areas, such as transportation or leisure?	○	○	○	○	○	○	○

B91. Do you (Does . . . . .) have any emotional, psychological or psychiatric conditions that have lasted, or are expected to last, 6 months or more? These include phobias, depression, schizophrenia, drinking or drug problems, and others.

(1) Yes . . . . ○
(3) No . . . . ○
(x) Don't know . . . . ○
(r) Refusal . . . . ○

(3), (x), (r) ► **Go to B94edit**

B92. Does this condition reduce the amount or the kind of activities you (. . . . .) can do?

Interviewer: Read list. Mark one only.

(1) Yes, sometimes . . . . ○
(2) Yes, often or always . . . . ○

(1), (2) ► Check box Emotional / Psychological – Limitation on Profile Sheet

(3) No . . . . ○
(x) Don't know . . . . ○
(r) Refusal . . . . ○

(3), (x), (r) ► **Go to B94edit**

B93. How many activities does this condition usually prevent you (. . . . .) from doing . . .

Interviewer: Read categories. Mark one only.

Interviewer: Read list.	(1) None	(2) A few	(3) Many	(4) Most	(5) N/A	(x) DK	(r) Ref
(a) at home?	○	○	○	○		○	○
(b) at work?	○	○	○	○	○	○	○
(c) at school?	○	○	○	○	○	○	○
(d) in other areas, such as transportation or leisure?	○	○	○	○	○	○	○

B94. edit

Interviewer: If any box is checked in the Limitation column on the Profile Sheet, then continue; otherwise, go to the Follow-up question on page 81.

B94. **You reported that because of a physical condition, mental condition or health problem, you have (. . . . . has) difficulties or limitations in doing certain activities. Do you (Does . . . . .) think that these difficulties or activity limitations create a disadvantage for you (him/her) at home?**

Interviewer: Read list. Mark one only.

(1) Yes, sometimes . . . . ◯
(2) Yes, often or always . . . . ◯
(3) No . . . . ◯
(x) Don't know . . . . ◯
(r) Refusal . . . . ◯

(3), (x), (r): **Go to B96**

B95. **How much of a disadvantage?**

Interviewer: Read list. Mark one only.

(1) Mild . . . . ◯
(2) Moderate . . . . ◯
(3) Severe . . . . ◯
(x) Don't know . . . . ◯
(r) Refusal . . . . ◯

B96. **Do you (Does . . . . .) think that these difficulties or activity limitations create a disadvantage for you (him/her) at work?**

Interviewer: Read list. Mark one only.

(1) Yes, sometimes . . . . ◯
(2) Yes, often or always . . . . ◯
(3) No . . . . ◯
(5) Not applicable . . . . ◯
(x) Don't know . . . . ◯
(r) Refusal . . . . ◯

(3), (5), (x), (r): **Go to B98**

B97. **How much of a disadvantage?**

Interviewer: Read list. Mark one only.

(1) Mild . . . . ◯
(2) Moderate . . . . ◯
(3) Severe . . . . ◯
(x) Don't know . . . . ◯
(r) Refusal . . . . ◯

B98. **Do you (Does . . . . .) think that these difficulties or activity limitations create a disadvantage for you (him/her) at school?**

Interviewer: Read list. Mark one only.

(1) Yes, sometimes . . . . ◯
(2) Yes, often or always . . . . ◯
(3) No . . . . ◯
(5) Not applicable . . . . ◯
(x) Don't know . . . . ◯
(r) Refusal . . . . ◯

(3), (5), (x), (r): **Go to B100**

B99. How much of a disadvantage?

Interviewer: Read list. Mark one only.

(1) Mild .... ◯
(2) Moderate .... ◯
(3) Severe .... ◯
(x) Don't know .... ◯
(r) Refusal .... ◯

B100. Do you (Does .....) think that these difficulties or activity limitations create a disadvantage for you (him/her) in other areas, such as transportation or leisure?

Interviewer: Read list. Mark one only.

(1) Yes, sometimes .... ◯
(2) Yes, often or always .... ◯
(3) No .... ◯ ▶ **Go to B102**
(5) Not applicable .... ◯ ▶ **Go to B102**
(x) Don't know .... ◯ ▶ **Go to B102**
(r) Refusal .... ◯ ▶ **Go to B102**

B101. How much of a disadvantage?

Interviewer: Read list. Mark one only.

(1) Mild .... ◯
(2) Moderate .... ◯
(3) Severe .... ◯
(x) Don't know .... ◯
(r) Refusal .... ◯

B102. At what age did you (.....) first start having any difficulty or activity limitation?

☐☐☐ (0-120) Years (if age less than 1 year, enter 00)

(x) Don't know .... ◯
(r) Refusal .... ◯

B103. What is the MAIN condition which causes you (.....) difficulty or limits your (his/her) activities, e.g., cataracts, arthritis, multiple sclerosis, etc.?

Specify

☐

(x) Don't know .... ◯ ▶ **Go to B112**
(r) Refusal .... ◯ ▶ **Go to B112**

B104. Did this condition exist at birth?

(1) Yes .... ◯ → **Go to B107**
(3) No .... ◯
(x) Don't know .... ◯
(r) Refusal .... ◯

B105. Which one of the following best describes the CAUSE of this condition?

Interviewer: Read list. Mark one only.

(1) A disease or illness .... ◯ } Go to B107
(2) Ageing .... ◯ } Go to B107
(3) Work conditions .... ◯ } Go to B107
(4) Stress .... ◯ } Go to B107
(5) An accident .... ◯
(6) Another cause, specify ..... ◯ → **Go to B107**

[ ]

(x) Don't know .... ◯
(r) Refusal .... ◯

B106. What type of accident?

Interviewer: Read list. Mark one only.

(1) An accident at home .... ◯
(2) A motor vehicle accident .... ◯
(3) An accident at work .... ◯
(4) A sports related accident .... ◯
(5) Another type of accident .... ◯

(x) Don't know .... ◯
(r) Refusal .... ◯

B107. Is there a SECOND condition which causes you (. . . . .) difficulty or limits your (his/her) activities?

(1) Yes .... ◯
(3) No .... ◯ } Go to B112
(x) Don't know .... ◯ } Go to B112
(r) Refusal .... ◯ } Go to B112

B108. What is this second condition?

Specify

[ ]

(x) Don't know .... ◯ } Go to B112
(r) Refusal .... ◯ } Go to B112

B109. Did this condition exist at birth?

(1) Yes .... ◯ → **Go to B112**
(3) No .... ◯
(x) Don't know .... ◯
(r) Refusal .... ◯

B110. Which one of the following best describes the CAUSE of this condition?

Interviewer: Read list. Mark one only.

(1) A disease or illness .... ◯
(2) Ageing .... ◯
(3) Work conditions .... ◯
(4) Stress .... ◯
(5) An accident .... ◯
→ **Go to B112**

(6) Another cause, specify ..... ◯ → **Go to B112**

[ ]

(x) Don't know .... ◯
(r) Refusal .... ◯

B111. What type of accident?

Interviewer: Read list. Mark one only.

(1) An accident at home .... ◯
(2) A motor vehicle accident .... ◯
(3) An accident at work .... ◯
(4) A sports related accident .... ◯
(5) Another type of accident .... ◯
(x) Don't know .... ◯
(r) Refusal .... ◯

B112. The next few questions are about your (. . . . . 's) use of medication or drugs.

Do you (Does . . . . .) use any medication or drugs, prescription or non-prescription, on a regular basis, that is AT LEAST ONCE A WEEK?

(1) Yes .... ◯
(3) No .... ◯
(x) Don't know .... ◯
(r) Refusal .... ◯
→ **Go to B118**

B113. How many kinds of PRESCRIPTION drugs do you (does . . . . .) take EACH DAY?

[|] (0-99)

B113. edit ***Interviewer: If more than 10 kinds of drugs, confirm response.***

B114. How many kinds of NON-PRESCRIPTION medication do you (does . . . . .) take EACH DAY?

[|] (0-99)

B114. edit ***Interviewer: If more than 10 kinds of medication, confirm response.***

B115. Are there any other kinds of medication or drugs you take (. . . . . takes) regularly BUT NOT DAILY?

(1) Yes .... ◯
(3) No .... ◯
(x) Don't know .... ◯
(r) Refusal .... ◯
→ **Go to B118**

B116. How many kinds of PRESCRIPTION drugs do you (does . . . . .) take regularly BUT NOT DAILY?

☐☐ (0-99)

B116. edit ***Interviewer: If more than 10 kinds of drugs, confirm response.***

B117. How many kinds of NON-PRESCRIPTION medication do you (does . . . . .) take regularly BUT NOT DAILY?

☐☐ (0-99)

B117. edit ***Interviewer: If more than 10 kinds of medication, confirm response.***

B118. In the past 12 months, did you (. . . . .) have any OUT-OF-POCKET or DIRECT EXPENSES for prescription and non-prescription drugs?

INCLUDE amounts not covered by insurance such as exclusions, deductibles and expenses over limits. EXCLUDE payments for which you have (. . . . . has) been or will be reimbursed by any insurance or government program.

(1) Yes . . . . ◯
(3) No . . . . ◯
(x) Don't know . . . . ◯
(r) Refusal . . . . ◯

No, Don't know, Refusal: **Go to B121**

B119. What is your (. . . . . 's) best estimate of the OUT-OF-POCKET or DIRECT COSTS to you (him/her) in the past 12 months, for prescription and non-prescription drugs?

(INCLUDE amounts not covered by insurance such as exclusions, deductibles and expenses over limits. EXCLUDE payments for which you have (. . . . . has) been or will be reimbursed by any insurance or government program.)

$ ☐☐☐☐☐☐ .00 Range: 1-999999

(x) Don't know . . . . ◯
(r) Refusal . . . . ◯

Don't know, Refusal: **Go to B120**

B119. edit ***Interviewer: If costs are over $5,000., confirm response. Go to B121.***

B120. Which of the following expense groups is the best estimate of the DIRECT COSTS to you (. . . . .) in the past 12 months, for prescription and non-prescription drugs?

Interviewer: Read list. Mark one only.

(1) less than $100 . . . . ◯
(2) $100 to less than $200 . . . . ◯
(3) $200 to less than $500 . . . . ◯
(4) $500 to less than $1,000 . . . . ◯
(5) $1,000 to less than $2,000 . . . . . ◯
(6) $2,000 to less than $5,000 . . . . . ◯
(7) $5,000 or more . . . . ◯
(x) Don't know . . . . ◯
(r) Refusal . . . . ◯

B121. In the past 12 months, were you (was . . . . .) ever UNABLE TO GET the medication or drugs you were (he/she was) supposed to use, because of the COST?

(1) Yes . . . . ◯
(3) No . . . . ◯
(x) Don't know . . . . ◯
(r) Refusal . . . . ◯

B122. In the past 12 months, did you (. . . . .) ever use your (his/her) medication or drugs LESS OFTEN than you were (he/she was) supposed to, because of the COST?

(1) Yes . . . . ◯
(3) No . . . . ◯
(x) Don't know . . . . ◯
(r) Refusal . . . . ◯

B123. In the past 12 months, did you (. . . . .) ever NOT USE the medication or drugs you were (he/she was) supposed to use, because of the SIDE EFFECTS?

(1) Yes . . . . ◯
(3) No . . . . ◯
(x) Don't know . . . . ◯
(r) Refusal . . . . ◯

B124. Because of your (his/her) condition, do you (does . . . . .) USE any other aids or specialized equipment that have not already been mentioned?

(1) Yes . . . . ◯ → Check box Other – USE aid on Profile Sheet
(3) No . . . . ◯
(x) Don't know . . . . ◯
(r) Refusal . . . . ◯
▶ **Go to B126edit**

B125. Do you (Does . . . . .) now USE:

Interviewer: Read list.

	(1) Yes	(3) No	(x) DK	(r) Ref
(a) respiratory aids, e.g., inhalers, puffers, oxygen?	◯	◯	◯	◯
(b) pain management aids, e.g., a TENS machine?	◯	◯	◯	◯
(c) another aid or other specialized equipment? — specify	◯ ↓	◯	◯	◯

[ ]

B126. edit

Interviewer: If any box is checked in the USE aid column on the Profile Sheet, then continue; otherwise, go to B129.

B126. I would now like you (. . . . .) to think of all the aids and specialized equipment that you USE (he/she USES).

In the past 12 months, did you (. . . . .) have any OUT-OF-POCKET or DIRECT EXPENSES for the purchase and maintenance of aids and specialized equipment?

(INCLUDE amounts not covered by insurance such as exclusions, deductibles and expenses over limits. EXCLUDE payments for which you have (. . . . . has) been or will be reimbursed by any insurance or government program.)

(1) Yes . . . . ◯
(3) No . . . . ◯
(x) Don't know . . . . ◯
(r) Refusal . . . . ◯
▶ **Go to B129**

B127. What is your (. . . . . 's) best estimate of the OUT-OF-POCKET or DIRECT COSTS to you (him/her) in the past 12 months, for the purchase and maintenance of aids and specialized equipment?

(INCLUDE amounts not covered by insurance such as exclusions, deductibles and expenses over limits. EXCLUDE payments for which you have (. . . . . has) been or will be reimbursed by any insurance or government program.)

$ ☐☐☐☐☐☐ .00 Range: 1-999999

(x) Don't know . . . . ◯
(r) Refusal . . . . ◯
→ **Go to B128**

B127. edit ***Interviewer:*** ***If costs are over $5,000., confirm response. Go to B129.***

B128. Which of the following expense groups is the best estimate of the DIRECT COSTS to you (. . . . .) in the past 12 months, for the purchase and maintenance of aids and specialized equipment?

Interviewer: Read list. Mark one only.

(1) less than $200 . . . . ◯
(2) $200 to less than $500 . . . . ◯
(3) $500 to less than $1,000 . . . . ◯
(4) $1,000 to less than $2,000 . . . . . ◯
(5) $2,000 to less than $5,000 . . . . . ◯
(6) $5,000 or more . . . . ◯

(x) Don't know . . . . ◯
(r) Refusal . . . . ◯

B129. Do you (Does . . . . .) NEED any other aids or specialized equipment that have not already been mentioned?

(1) Yes . . . . ◯ → Check box Other – NEED aid on Profile Sheet
(3) No . . . . ◯
(x) Don't know . . . . ◯
(r) Refusal . . . . ◯
→ **Go to B131edit**

B130. Which aids do you (does . . . . .) NEED but do (does) not have?

Interviewer: Do not read list. Mark all that apply.

(a) Respiratory aids, e.g., inhalers, puffers, oxygen . . . . ☐
(b) Pain management aids, e.g., a TENS machine . . . . ☐
(c) Other, specify . . . . ☐ ↓

☐

(x) Don't know . . . . ☐
(r) Refusal . . . . ☐

B131. edit ***Interviewer:*** ***If any box is checked in the NEED aid column on the Profile Sheet, then continue; otherwise, go to C1.***

B131. Now, I would like you (. . . . .) to think about all the aids and specialized equipment you NEED (he/she NEEDS) but do (does) not have.

Why do you (does . . . . .) not have these aids? I will read you a list of possible reasons. Please answer yes or no to each.

Interviewer: Read list.

	(1) Yes	(3) No	(x) DK	(r) Ref
(a) It is not covered by insurance	◯	◯	◯	◯
(b) It is too expensive	◯	◯	◯	◯
(c) Your (. . . . . 's) condition is not serious enough	◯	◯	◯	◯
(d) You do (. . . . . does) not know where or how to obtain it	◯	◯	◯	◯
(e) It is not available	◯	◯	◯	◯
(f) Another reason, specify	◯ →	◯	◯	◯

SECTION C — HELP WITH EVERYDAY ACTIVITIES

C1. The next questions are about the help you receive (. . . . . receives) with everyday activities BECAUSE OF YOUR (HIS/HER) CONDITION. Include help received from your (his/her) spouse or partner, from family members, friends or neighbours, and from organizations, whether paid or unpaid.

Because of your (his/her) condition, do you (does . . . . .) usually RECEIVE help with preparing meals?

(1) Yes ◯ → Check box Meals – RECEIVE help on Profile Sheet **Go to C3**

(3) No ◯

(x) Don't know ◯
(r) Refusal ◯ ▶ **Go to C5**

C2. Do you think you NEED (Does . . . . . think he/she NEEDS) help with preparing meals?

(1) Yes ◯ → Check box Meals – NEED help on Profile Sheet **Go to C5**

(3) No ◯
(x) Don't know ◯
(r) Refusal ◯ ▶ **Go to C5**

C3. WHO usually helps you (. . . . .) with preparing meals? I will read you a list. Please answer yes or no to each.

Interviewer: Read list.
For each "Yes" response, ask: C3A.

C3A. HOW OFTEN do they help with preparing meals?

Interviewer: Read categories. Mark one only.

	(r) Ref	(x) DK	(3) No	(1) Yes		C3A (1) Every-day	(2) At least once a week	(3) Less than once a week	(x) DK	(r) Ref
(a) Family living with you (him/her)	◯	◯	◯	◯	→	◯	◯	◯	◯	◯
(b) Family not living with you (him/her)	◯	◯	◯	◯	→	◯	◯	◯	◯	◯
(c) Friends or neighbours	◯	◯	◯	◯	→	◯	◯	◯	◯	◯
(d) Organization or agency (Include voluntary, private and government agencies)	◯	◯	◯	◯	→	◯	◯	◯	◯	◯
(e) Other	◯	◯	◯	◯	→	◯	◯	◯	◯	◯

C4. Do you (Does . . . . .) need ADDITIONAL help with preparing meals?

(1) Yes . . . . ◯ → Check box Meals – NEED help on Profile Sheet
(3) No . . . . ◯
(x) Don't know . . . . ◯
(r) Refusal . . . . ◯

C5. Because of your (his/her) condition, do you (does . . . . .) usually RECEIVE help with everyday housework, such as dusting and tidying up?

(1) Yes . . . . ◯ → Check box Housework – RECEIVE help on Profile Sheet **Go to C7**
(3) No . . . . ◯
(x) Don't know . . . . ◯ ▶ **Go to C9**
(r) Refusal . . . . ◯

C6. Do you think you NEED (Does . . . . . think he/she NEEDS) help with everyday housework?

(1) Yes . . . . ◯ → Check box Housework – NEED help on Profile Sheet **Go to C9**
(3) No . . . . ◯ ▶ **Go to C9**
(x) Don't know . . . . ◯
(r) Refusal . . . . ◯

C7. WHO usually helps you (. . . . .) with everyday housework? I will read you a list. Please answer yes or no to each.

Interviewer: Read list.
For each "Yes" response, ask: C7A.

C7A. HOW OFTEN do they help with everyday housework?

Interviewer: Read categories.
Mark one only.

	(r) Ref	(x) DK	(3) No	(1) Yes		(1) Every-day	(2) At least once a week	(3) Less than once a week	(x) DK	(r) Ref
(a) Family living with you (him/her)	◯	◯	◯	◯	→	◯	◯	◯	◯	◯
(b) Family not living with you (him/her)	◯	◯	◯	◯	→	◯	◯	◯	◯	◯
(c) Friends or neighbours	◯	◯	◯	◯	→	◯	◯	◯	◯	◯
(d) Organization or agency (Include voluntary, private and government agencies) . . .	◯	◯	◯	◯	→	◯	◯	◯	◯	◯
(e) Other . . . . Specify	◯	◯	◯	◯	→	◯	◯	◯	◯	◯

C8. Do you (Does . . . . .) need ADDITIONAL help with everyday housework?

(1) Yes . . . . ◯ → Check box Housework – NEED help on Profile Sheet
(3) No . . . . ◯
(x) Don't know . . . . ◯
(r) Refusal . . . . ◯

C9. Because of your (his/her) condition, do you (does . . . . .) usually RECEIVE help with heavy household chores, such as spring cleaning or yard work?

(1) Yes . . . . ◯ → Check box Chores – RECEIVE help on Profile Sheet **Go to C11**
(3) No . . . . ◯
(x) Don't know . . . . ◯ ▶ **Go to C13**
(r) Refusal . . . . ◯

C10. Do you think you NEED (Does . . . . . think he/she NEEDS) help with heavy household chores?

(1) Yes . . . . ◯ → Check box Chores – NEED help on Profile Sheet **Go to C13**

(3) No . . . . ◯
(x) Don't know . . . . ◯
(r) Refusal . . . . ◯
▶ **Go to C13**

C11. WHO usually helps you (. . . . .) with heavy household chores? I will read you a list. Please answer yes or no to each.

Interviewer: Read list.
For each "Yes" response, ask: C11A.

C11A. HOW OFTEN do they help with heavy household chores?

Interviewer: Read categories. Mark one only.

	(r) Ref	(x) DK	(3) No	(1) Yes		(1) At least once a week	(2) At least once a month	(3) Less than once a month	(x) DK	(r) Ref
(a) Family living with you (him/her)	◯	◯	◯	◯	→	◯	◯	◯	◯	◯
(b) Family not living with you (him/her)	◯	◯	◯	◯	→	◯	◯	◯	◯	◯
(c) Friends or neighbours	◯	◯	◯	◯	→	◯	◯	◯	◯	◯
(d) Organization or agency (Include voluntary, private and government agencies) . . .	◯	◯	◯	◯	→	◯	◯	◯	◯	◯
(e) Other	◯	◯	◯	◯	→	◯	◯	◯	◯	◯

Specify

C12. Do you (Does . . . . .) need ADDITIONAL help with heavy household chores?

(1) Yes . . . . ◯ → Check box Chores – NEED help on Profile Sheet
(3) No . . . . ◯
(x) Don't know . . . . ◯
(r) Refusal . . . . ◯

C13. Because of your (his/her) condition, do you (does . . . . .) usually RECEIVE help with getting to appointments and running errands, such as shopping for groceries?

(1) Yes . . . . ◯ → Check box Appointments – RECEIVE help on Profile Sheet **Go to C15**

(3) No . . . . ◯

(x) Don't know . . . . ◯
(r) Refusal . . . . ◯
▶ **Go to C17**

C14. Do you think you NEED (Does . . . . . think he/she NEEDS) help with getting to appointments and running errands, such as shopping for groceries?

(1) Yes . . . . ◯ → Check box Appointments – NEED help on Profile Sheet **Go to C17**

(3) No . . . . ◯
(x) Don't know . . . . ◯
(r) Refusal . . . . ◯
▶ **Go to C17**

C15. WHO usually helps you (. . . . .) with getting to appointments and running errands? I will read you a list. Please answer yes or no to each.

Interviewer: Read list.
For each "Yes" response, ask: C15A.

C15A. HOW OFTEN do they help with getting to appointments and running errands?

Interviewer: Read categories. Mark one only.

	(r) Ref	(x) DK	(3) No	(1) Yes		(1) Every-day	(2) At least once a week	(3) Less than once a week	(x) DK	(r) Ref
(a) Family living with you (him/her)	◯	◯	◯	◯	→	◯	◯	◯	◯	◯
(b) Family not living with you (him/her)	◯	◯	◯	◯	→	◯	◯	◯	◯	◯
(c) Friends or neighbours	◯	◯	◯	◯	→	◯	◯	◯	◯	◯
(d) Organization or agency (Include voluntary, private and government agencies) . . .	◯	◯	◯	◯	→	◯	◯	◯	◯	◯
(e) Other	◯	◯	◯	◯	→	◯	◯	◯	◯	◯

C16. Do you (Does . . . . .) need ADDITIONAL help with getting to appointments and running errands?

(1) Yes . . . . ◯ → Check box Appointments – NEED help on Profile Sheet
(3) No . . . . ◯
(x) Don't know . . . . ◯
(r) Refusal . . . . ◯

C17. Because of your (his/her) condition, do you (does . . . . .) usually RECEIVE help with looking after your (his/her) personal finances, such as making bank transactions or paying bills?

(1) Yes . . . . ◯ → Check box Finances – RECEIVE help on Profile Sheet **Go to C19**
(3) No . . . . ◯
(x) Don't know . . . . ◯
(r) Refusal . . . . ◯ ▶ **Go to C21** (Don't know, Refusal)

C18. Do you think you NEED (Does . . . . . think he/she NEEDS) help with looking after your (his/her) personal finances?

(1) Yes . . . . ◯ → Check box Finances – NEED help on Profile Sheet **Go to C21**
(3) No . . . . ◯
(x) Don't know . . . . ◯
(r) Refusal . . . . ◯ ▶ **Go to C21** (No, Don't know, Refusal)

C19. WHO usually helps you (. . . . .) with looking after your (his/her) personal finances? I will read you a list. Please answer yes or no to each.

Interviewer: Read list.
For each "Yes" response, ask: C19A.

C19A. HOW OFTEN do they help with looking after your (his/her) personal finances?

Interviewer: Read categories. Mark one only.

	(r) Ref	(x) DK	(3) No	(1) Yes		(1) At least once a week	(2) At least once a month	(3) Less than once a month	(x) DK	(r) Ref
(a) Family living with you (him/her)	◯	◯	◯	◯	→	◯	◯	◯	◯	◯
(b) Family not living with you (him/her)	◯	◯	◯	◯	→	◯	◯	◯	◯	◯
(c) Friends or neighbours	◯	◯	◯	◯	→	◯	◯	◯	◯	◯
(d) Organization or agency (Include voluntary, private and government agencies) . . .	◯	◯	◯	◯	→	◯	◯	◯	◯	◯
(e) Other	◯	◯	◯	◯	→	◯	◯	◯	◯	◯

C20. Do you (Does . . . . .) need ADDITIONAL help with looking after your (his/her) personal finances?

(1) Yes . . . . ◯ → Check box Finances – NEED help on Profile Sheet
(3) No . . . . ◯
(x) Don't know . . . . ◯
(r) Refusal . . . . ◯

C21. Are there any children less than 15 years of age living with you (. . . . .)?

(1) Yes . . . . ◯
(3) No . . . . ◯
(x) Don't know . . . . ◯
(r) Refusal . . . . ◯
▶ **Go to C26** (No, Don't know, Refusal)

C22. Because of your (his/her) condition, do you (does . . . . .) usually RECEIVE help with child care?

(1) Yes . . . . ◯ → Check box Childcare – RECEIVE help on Profile Sheet **Go to C24**
(3) No . . . . ◯
(x) Don't know . . . . ◯
(r) Refusal . . . . ◯
▶ **Go to C26** (Don't know, Refusal)

C23. Do you think you NEED (Does . . . . . think he/she NEEDS) help with child care?

(1) Yes . . . . ◯ → Check box Childcare – NEED help on Profile Sheet **Go to C26**
(3) No . . . . ◯
(x) Don't know . . . . ◯
(r) Refusal . . . . ◯
▶ **Go to C26** (No, Don't know, Refusal)

C24. WHO usually helps you (. . . . .) with child care? I will read you a list. Please answer yes or no to each.

Interviewer: Read list.
For each "Yes" response, ask: C24A.

C24A. HOW OFTEN do they help with child care?

Interviewer: Read categories. Mark one only.

	(r) Ref	(x) DK	(3) No	(1) Yes		(1) Every-day	(2) At least once a week	(3) Less than once a week	(x) DK	(r) Ref
(a) Family living with you (him/her)	◯	◯	◯	◯	→	◯	◯	◯	◯	◯
(b) Family not living with you (him/her)	◯	◯	◯	◯	→	◯	◯	◯	◯	◯
(c) Friends or neighbours	◯	◯	◯	◯	→	◯	◯	◯	◯	◯
(d) Organization or agency (Include voluntary, private and government agencies) . . .	◯	◯	◯	◯	→	◯	◯	◯	◯	◯
(e) Other	◯	◯	◯	◯	→	◯	◯	◯	◯	◯

C25. Do you (Does . . . . .) need ADDITIONAL help with child care?

(1) Yes . . . . ◯ → Check box Childcare – NEED help on Profile Sheet
(3) No . . . . ◯
(x) Don't know . . . . ◯
(r) Refusal . . . . ◯

C26. Because of your (his/her) condition, do you (does . . . . .) usually RECEIVE help with personal care, such as washing, dressing or taking medication?

(1) Yes . . . . ◯ → Check box Personal Care – RECEIVE help on Profile Sheet **Go to C28**
(3) No . . . . ◯
(x) Don't know . . . . ◯ ▶ **Go to C30**
(r) Refusal . . . . ◯

C27. Do you think you NEED (Does . . . . . think he/she NEEDS) help with personal care?

(1) Yes . . . . ◯ → Check box Personal Care – NEED help on Profile Sheet **Go to C30**
(3) No . . . . ◯ ▶ **Go to C30**
(x) Don't know . . . . ◯
(r) Refusal . . . . ◯

C28. WHO usually helps you (. . . . .) with personal care? I will read you a list. Please answer yes or no to each.

Interviewer: Read list.
For each "Yes" response, ask: C28A and C28B.

C28A AND C28B

	(r) Ref	(x) DK	(3) No	(1) Yes		**C28A On average, how many days a week do they help with personal care?** (1-7)	**C28B On average, how many hours a day?** (0.5-24)
(a) Family living with you (him/her)	◯	◯	◯	◯	→	☐	☐☐.☐
(b) Family not living with you (him/her)	◯	◯	◯	◯	→	☐	☐☐.☐
(c) Friends or neighbours	◯	◯	◯	◯	→	☐	☐☐.☐
(d) Organization or agency (Include voluntary, private and government agencies) . . .	◯	◯	◯	◯	→	☐	☐☐.☐
(e) Other	◯	◯	◯	◯	→	☐	☐☐.☐

C29. Do you (Does . . . . .) need ADDITIONAL help with personal care?

(1) Yes . . . . ◯ → Check box Personal Care – NEED help on Profile Sheet
(3) No . . . . ◯
(x) Don't know . . . . ◯
(r) Refusal . . . . ◯

C30. Because of your (his/her) condition, do you (does . . . . .) usually RECEIVE specialized nursing care or medical treatment at home such as injections, therapy, blood, urine testing or catheter care?

(1) Yes . . . . ◯ → Check box Nursing/Treatment – RECEIVE help on Profile Sheet **Go to C32**
(3) No . . . . ◯
(x) Don't know . . . . ◯
(r) Refusal . . . . ◯ ▶ **Go to C34**

C31. Do you think you NEED (Does . . . . . think he/she NEEDS) specialized nursing care or medical treatment at home?

(1) Yes . . . . ◯ → Check box Nursing/Treatment – NEED help on Profile Sheet **Go to C34**
(3) No . . . . ◯
(x) Don't know . . . . ◯
(r) Refusal . . . . ◯ ▶ **Go to C34**

C32. WHO usually provides you (. . . . .) specialized nursing care or medical treatment at home? I will read you a list. Please answer yes or no to each.

Interviewer: Read list.
For each "Yes" response, ask: C32A.

C32A. HOW OFTEN do they provide specialized nursing care or medical treatment at home?

Interviewer: Read categories. Mark one only.

	(r) Ref	(x) DK	(3) No	(1) Yes		(1) Every-day	(2) At least once a week	(3) Less than once a week	(x) DK	(r) Ref
(a) Family living with you (him/her)	◯	◯	◯	◯	→	◯	◯	◯	◯	◯
(b) Family not living with you (him/her)	◯	◯	◯	◯	→	◯	◯	◯	◯	◯
(c) Friends or neighbours	◯	◯	◯	◯	→	◯	◯	◯	◯	◯
(d) Organization or agency (Include voluntary, private and government agencies) . . .	◯	◯	◯	◯	→	◯	◯	◯	◯	◯
(e) Other	◯	◯	◯	◯	→	◯	◯	◯	◯	◯

C33. Do you (Does . . . . .) need ADDITIONAL specialized nursing care or medical treatment at home?

(1) Yes . . . . ◯ → Check box Nursing/Treatment – NEED help on Profile Sheet
(3) No . . . . ◯
(x) Don't know . . . . ◯
(r) Refusal . . . . ◯

C34. Because of your (his/her) condition, do you (does . . . . .) usually RECEIVE help with moving about inside your (his/her) residence?

(1) Yes . . . . ◯ → Check box Moving About – RECEIVE help on Profile Sheet **Go to C36**
(3) No . . . . ◯
(x) Don't know . . . . ◯
(r) Refusal . . . . ◯ ▶ **Go to C38edit**

C35. Do you think you NEED (Does . . . . . think he/she NEEDS) help with moving about inside your (his/her) residence?

(1) Yes . . . . ◯ → Check box Moving About – NEED help on Profile Sheet **Go to C38edit**

(3) No . . . . ◯
(x) Don't know . . . . ◯
(r) Refusal . . . . ◯
→ **Go to C38edit**

C36. WHO usually helps you (. . . . .) with moving about inside your (his/her) residence? I will read you a list. Please answer yes or no to each.

Interviewer: Read list.

	(1) Yes	(3) No	(x) DK	(r) Ref
(a) Family living with you (him/her)	◯	◯	◯	◯
(b) Family not living with you (him/her)	◯	◯	◯	◯
(c) Friends or neighbours	◯	◯	◯	◯
(d) Organization or agency (Include voluntary, private and government agencies)	◯	◯	◯	◯
(e) Other	◯	◯	◯	◯

C37. Do you (Does . . . . .) need ADDITIONAL help with moving about inside your (his/her) residence?

(1) Yes . . . . ◯ → Check box Moving About – NEED help on Profile Sheet
(3) No . . . . ◯
(x) Don't know . . . . ◯
(r) Refusal . . . . ◯

C38. edit ***Interviewer: If any box is checked in the RECEIVE help column on the Profile Sheet, then continue; otherwise, go to C45edit.***

C38. Now, I would like you (. . . . .) to think of all the help you RECEIVE (he/she RECEIVES) with everyday activities.

Was it difficult to make the arrangements for the help you receive (. . . . . receives)?

(1) Yes . . . . ◯
(3) No . . . . ◯
(x) Don't know . . . . ◯
(r) Refusal . . . . ◯
→ **Go to C40**

C39. What were the difficulties? I will read you a list. Please answer yes or no to each.

Interviewer: Read list.

	(1) Yes	(3) No	(x) DK	(r) Ref
(a) Finding qualified help	◯	◯	◯	◯
(b) Delay in obtaining assistance	◯	◯	◯	◯
(c) Did not know where to look for help	◯	◯	◯	◯
(d) Too expensive	◯	◯	◯	◯
(e) Other, specify	◯	◯	◯	◯

C40. The next few questions are about the cost of the help you receive (. . . . . receives) with everyday activities.

Who pays for the help you receive (. . . . . receives)?

Interviewer: Do not read list. Mark all that apply.

(a) No one, it's free . . . . ☐
(b) Yourself (. . . . .) or family living with you (him/her) . . . . ☐
(c) Family not living with you (him/her) . . . . ☐
(d) Private health insurance, e.g., employer insurance plan . . . . ☐
(e) Home care program . . . . ☐
(f) Voluntary organization . . . . ☐
(g) Other private source . . . . ☐
(h) Other public source, e.g., government health insurance plan . . . . ☐

(x) Don't know . . . . ☐
(r) Refusal . . . . ☐

C40. edit

Interviewer: If C40(b) is checked, then continue. Otherwise, go to C45edit.

C41. Is the cost to you (. . . . .) or your (his/her) family living with you (him/her) . . .

Interviewer: Read list. Mark one only.

(1) fully reimbursed . . . . ◯
(2) partially reimbursed . . . . ◯
(3) not reimbursed . . . . ◯ ▶ **Go to C43**
(x) Don't know . . . . ◯ ▶ **Go to C43**
(r) Refusal . . . . ◯ ▶ **Go to C43**

C42. From what source does the reimbursement come? I will read you a list. Please answer yes or no to each.

Interviewer: Read list.

	(1) Yes	(3) No	(x) DK	(r) Ref
(a) Government tax credit	◯	◯	◯	◯
(b) Direct government financial support	◯	◯	◯	◯
(c) Private health insurance	◯	◯	◯	◯
(d) Other source	◯	◯	◯	◯

C42. edit

Interviewer: If C41 is (2) "partially reimbursed", then continue. Otherwise, go to C45edit.

C43. What is your (. . . . . 's) best estimate of the OUT-OF-POCKET or DIRECT COSTS to you (him/her), or your (his/her) family living with you (him/her), for the help you (he/she) received in the past 12 months?

INCLUDE amounts not covered by insurance such as exclusions, deductibles and expenses over limits. EXCLUDE payments for which you have (. . . . . has) been or will be reimbursed by any insurance or government program.

$ ☐☐☐☐☐☐ .00 Range: 1-999999

(x) Don't know . . . . ◯ ▶ **Go to C44**
(r) Refusal . . . . ◯ ▶ **Go to C44**

C43. edit

Interviewer: If costs are over $5,000., confirm response. Go to C45edit.

C44. Which of the following expense groups is the best estimate of the DIRECT COSTS to you (. . . . .), or your (his/her) family living with you (him/her), for the help you (he/she) received in the past 12 months?

Interviewer: Read list. Mark one only.

(1) Less than $200 . . . . ◯
(2) $200 to less than $500 . . . . ◯
(3) $500 to less than $1,000 . . . . ◯
(4) $1,000 to less than $2,000 . . . . . ◯
(5) $2,000 to less than $5,000 . . . . . ◯
(6) $5,000 or more . . . . ◯

(x) Don't know . . . . ◯
(r) Refusal . . . . ◯

C45. edit

Interviewer: If any box is checked in the NEED help column on the Profile Sheet, then continue; otherwise, go to C46.

C45. Now, I would like you (. . . . .) to think about all the help you NEED (. . . . . NEEDS) but do (does) not have.

Why do you (does . . . . .) not receive the help you NEED (he/she NEEDS)? I will read you a list of possible reasons. Please answer yes or no to each.

Interviewer: Read list.

	(1) Yes	(3) No	(x) DK	(r) Ref
(a) You (. . . . .) applied for home care and were (was) turned down	◯	◯	◯	◯
(b) You are (. . . . . is) presently on a waiting list	◯	◯	◯	◯
(c) It is not covered by insurance	◯	◯	◯	◯
(d) It is too expensive	◯	◯	◯	◯
(e) You do (. . . . . does) not know where to obtain it	◯	◯	◯	◯
(f) Help is not available in the area	◯	◯	◯	◯
(g) Informal help, e.g., from family or friends, is not available	◯	◯	◯	◯
(h) Another reason, specify	◯ ↓	◯	◯	◯

[ ]

C46. Which of the following best describes the control you have (. . . . . has) in making decisions:

Interviewer: Read list. Mark one only.

(1) I make (He/She makes) all decisions about my (his/her) everyday activities . . . . ◯
(2) I make (He/She makes) the majority of decisions about my (his/her) everyday activities . . . . ◯
(3) I make (He/She makes) some of the decisions about my (his/her) everyday activities . . . . ◯
(4) I don't (He/She does not) make any decisions about my (his/her) everyday activities . . . . ◯

(x) Don't know . . . . ◯
(r) Refusal . . . . ◯

C47. **The next few questions are about the contacts you have (. . . . . has) with health care and social service providers because of your (his/her) condition.**

EXCLUDE any contacts AT HOME with health professionals providing you (. . . . .) specialized nursing care or medical treatment.

In the PAST 12 MONTHS, how often have you (has . . . . .) seen or talked about your (his/her) physical, emotional or mental condition, with . . .

Interviewer: Read categories. Mark one only.

Interviewer: Read list.	(1) At least once a week	(2) At least once a month	(3) Less than once a month	(4) Never	(x) DK	(r) Ref
(a) a physician (including general practitioners and specialists)?	○	○	○	○	○	○
(b) a physiotherapist or occupational therapist?	○	○	○	○	○	○
(c) an audiologist or speech therapist?	○	○	○	○	○	○
(d) a chiropractor?	○	○	○	○	○	○
(e) a massage therapist?	○	○	○	○	○	○
(f) a psychologist, social worker or counsellor?	○	○	○	○	○	○
(g) another health care or social service provider?	○	○	○	○	○	○

Specify

C48. edit ***Interviewer: If there is at least one checkmark in columns (1), (2) OR (3) of C47, then continue. Otherwise, go to C51.***

C48. **In the past 12 months, did you (. . . . .) have any OUT-OF-POCKET or DIRECT EXPENSES for the health care and social services you (he/she) received?**

INCLUDE amounts not covered by insurance such as exclusions, deductibles and expenses over limits. EXCLUDE payments for which you have (. . . . . has) been or will be reimbursed by any insurance or government program.

(1) Yes . . . . ○
(3) No . . . . ○
(x) Don't know . . . . ○
(r) Refusal . . . . ○

(3), (x), (r): **Go to C51**

C49. **What is your (. . . . . 's) best estimate of the OUT-OF-POCKET or DIRECT COSTS to you (him/her) for the health care and social services you (he/she) received in the past 12 months?**

(INCLUDE amounts not covered by insurance such as exclusions, deductibles and expenses over limits. EXCLUDE payments for which you have (. . . . . has) been or will be reimbursed by any insurance or government program.)

$ ☐☐☐☐☐☐ .00 Range: 1-999999

(x) Don't know . . . . ○
(r) Refusal . . . . ○

Go to C50

C49. edit ***Interviewer: If costs are over $5,000., confirm response. Go to C51.***

C50. Which of the following expense groups is the best estimate of the DIRECT COSTS to you (. . . . .), for the health care and social services you (he / she) received in the past 12 months?

Interviewer: Read list. Mark one only.

(1) Less than $200 . . . . ◯
(2) $200 to less than $500 . . . . ◯
(3) $500 to less than $1,000 . . . . ◯
(4) $1,000 to less than $2,000 . . . . . ◯
(5) $2,000 to less than $5,000 . . . . . ◯
(6) $5,000 or more . . . . ◯

(x) Don't know . . . . ◯
(r) Refusal . . . . ◯

C51. In the past 12 months, was there ever a time when you felt you (. . . . . felt that he / she) NEEDED health care or social services because of your (his / her) condition, but you (he / she) did not receive them?

(1) Yes . . . . ◯
(3) No . . . . ◯ ▶ **Go to D1**
(x) Don't know . . . . ◯ ▶ **Go to D1**
(r) Refusal . . . . ◯ ▶ **Go to D1**

C52. Why did you (. . . . .) not get these services? I will read you a list of reasons. Please answer yes or no to each.

Interviewer: Read list.

	(1) Yes	(3) No	(x) DK	(r) Ref
(a) They are not covered by insurance	◯	◯	◯	◯
(b) They are too expensive	◯	◯	◯	◯
(c) Your (. . . . . 's) condition is not serious enough	◯	◯	◯	◯
(d) You do (. . . . . does) not know where or how to obtain them	◯	◯	◯	◯
(e) They are not available in the area	◯	◯	◯	◯
(f) Another reason, specify	◯ ↓	◯	◯	◯

[ ]

SECTION D — EDUCATION

Interviewer: If respondent was born AFTER May 15, 1936, continue. Otherwise, go to Section F (page 65).

D1. The next few questions are on education.

In April 2001, were you (was . . . . .) attending a school, college or university? (Include private schools, colleges or universities.)

(1) Yes . . . . ◯ → **Go to D3**
(3) No . . . . ◯
(x) Don't know . . . . ◯
(r) Refusal . . . . ◯

D2. Did you (. . . . .) attend school after April, 1996?

(1) Yes . . . . ◯ → **Go to D13**
(3) No . . . . ◯ ▶ **Go to D18**
(x) Don't know . . . . ◯ ▶ **Go to D18**
(r) Refusal . . . . ◯ ▶ **Go to D18**

D3. Were you (Was . . . . .) enrolled as a:

Interviewer: Read list. Mark one only.

(1) full-time student . . . . . ◯ → **Go to D5**
(2) part-time student . . . . ◯
(x) Don't know . . . . ◯ } **Go to D5**
(r) Refusal . . . . ◯ }

D4. Were you (Was . . . . .) studying part-time because of your (his/her) condition?

(1) Yes . . . . ◯
(3) No . . . . ◯
(x) Don't know . . . . ◯
(r) Refusal . . . . ◯

D5. Were you (Was . . . . .) taking any courses by correspondence or home study in April 2001? Consider only courses which can be used as credits towards a certificate, diploma or degree.

(1) Yes . . . . ◯
(3) No . . . . ◯
(x) Don't know . . . . ◯
(r) Refusal . . . . ◯

D6. In April 2001, in what kind of school were you (was . . . . .) enrolled? I will read you a list. Please specify one only.

Interviewer: Read list. Mark one only.

(1) Regular primary or secondary school . . . . ◯
(2) Special education school . . . . ◯
(3) Community college, CEGEP or technical institute . . . . ◯ } **Go to D8**
(4) Private training institutes, for example, business schools or trade or vocational schools . . . . ◯ }
(5) University . . . . ◯ }
(6) Other . . . . ◯ }
Specify

____

(x) Don't know . . . . ◯ } **Go to D9**
(r) Refusal . . . . ◯ }

D7. In what grade were you (was . . . . .) enrolled in April 2001?

[|] Grade (1-13) → **Go to D9**

(0) Non-graded . . . . ◯ } **Go to D9**
(x) Don't know . . . . ◯ }
(r) Refusal . . . . ◯ }

D8. What type of certificate, diploma or degree were you (was . . . . .) seeking? I will read you a list. Please indicate all that apply.

Interviewer: Read list. Mark all that apply.

- (a) Trades certificate or diploma . . . . ☐
- (b) Other non-university certificate or diploma (obtained at community college, CEGEP, technical institute, etc.) . . . . . ☐
- (c) University certificate or diploma **below** bachelor level . . . . . . ☐
- (d) Bachelor's degree(s) (e.g., B.A., B.Sc., LL.B.) . . . . ☐
- (e) University certificate or diploma **above** bachelor level . . . . . . ☐
- (f) Master's degree(s) (e.g., M.A., M.Sc., M.Ed.) . . . . ☐
- (g) Degree in medicine, dentistry, veterinary medicine or optometry (e.g., M.D., D.D.S., D.M.D., D.V.M., O.D.) . . . . ☐
- (h) Earned doctorate (e.g., Ph.D., D.Sc., D.Ed.) . . . . ☐
- (x) Don't know . . . . ☐
- (r) Refusal . . . . ☐

D9. Did you (. . . . .) require modified building features or services to attend school?

- (1) Yes . . . . ○
- (3) No . . . . ○ ⟩ **Go to D11**
- (x) Don't know . . . . ○ ⟩ **Go to D11**
- (r) Refusal . . . . ○ ⟩ **Go to D11**

D10. Did you (. . . . .) require . . .

Interviewer: Read list.
For each "Yes" response, ask: D10A.

D10A. Was this available to you (. . . . .)?

	(r) Ref	(x) DK	(3) No	(1) Yes		D10A (1) Yes	D10A (3) No	D10A (x) DK	D10A (r) Ref
(a) accessible classrooms?	○	○	○	○	→	○	○	○	○
(b) accessible washrooms?	○	○	○	○	→	○	○	○	○
(c) accessible residences?	○	○	○	○	→	○	○	○	○
(d) accessible buildings, excluding residences?	○	○	○	○	→	○	○	○	○
(e) accessible transportation?	○	○	○	○	→	○	○	○	○
(f) other feature or service? . . . . Specify	○	○	○	○	→	○	○	○	○

[   ]

D11. Did you (. . . . .) need any assistive devices or services to follow your (his/her) courses?

- (1) Yes . . . . ○
- (3) No . . . . ○ ⟩ **Go to D20**
- (x) Don't know . . . . ○ ⟩ **Go to D20**
- (r) Refusal . . . . ○ ⟩ **Go to D20**

D12. **Did you (. . . . .) need . . .**

Interviewer: Read list.
For each "Yes" response, ask: D12A.

D12A. Was it made available to you (. . . . .)?

	(r) Ref	(x) DK	(3) No	(1) Yes		D12A: (1) Yes	(3) No	(x) DK	(r) Ref
(a) note takers or readers?	○	○	○	○	→	○	○	○	○
(b) a tutor or teacher's aide?	○	○	○	○	→	○	○	○	○
(c) a computer with Braille, large print or speech access?	○	○	○	○	→	○	○	○	○
(d) talking books?	○	○	○	○	→	○	○	○	○
(e) magnifiers or CCTV's (Closed circuit television readers)?	○	○	○	○	→	○	○	○	○
(f) Braille or large print reading materials?	○	○	○	○	→	○	○	○	○
(g) a Sign language interpreter?	○	○	○	○	→	○	○	○	○
(h) recording equipment or portable note-takers?	○	○	○	○	→	○	○	○	○
(i) attendant care services?	○	○	○	○	→	○	○	○	○
(j) other aid or service?	○	○	○	○	→	○	○	○	○

Specify ↓

[ ]

D12. edit ***Interviewer: Go to D20.***

D13. **Did you (. . . . .) have your (his/her) condition when you were (he/she was) attending school (after April, 1996)?**

(1) Yes . . . . ○
(3) No . . . . ○
(x) Don't know . . . . ○
(r) Refusal . . . . ○

(No, Don't know, Refusal) **Go to D18**

D14. **Did you (. . . . .) require modified building features or services to attend school?**

(1) Yes . . . . ○
(3) No . . . . ○
(x) Don't know . . . . ○
(r) Refusal . . . . ○

(No, Don't know, Refusal) **Go to D16**

D15. **Did you (. . . . .) require . . .**

Interviewer: Read list.
For each "Yes" response, ask: D15A.

D15A. Was this available to you (. . . . .)?

	(r) Ref	(x) DK	(3) No	(1) Yes		D15A: (1) Yes	(3) No	(x) DK	(r) Ref
(a) accessible classrooms?	○	○	○	○	→	○	○	○	○
(b) accessible washrooms?	○	○	○	○	→	○	○	○	○
(c) accessible residences?	○	○	○	○	→	○	○	○	○
(d) accessible buildings, excluding residences?	○	○	○	○	→	○	○	○	○
(e) accessible transportation?	○	○	○	○	→	○	○	○	○
(f) other feature or service?	○	○	○	○	→	○	○	○	○

Specify ↓

[ ]

D16. Did you (. . . . .) need any assistive devices or services to follow your (his/her) courses?

(1) Yes . . . . ◯
(3) No . . . . ◯
(x) Don't know . . . . ◯
(r) Refusal . . . . ◯

(3), (x), (r): **Go to D18**

D17. Did you (. . . . .) need . . .

Interviewer: Read list.
For each "Yes" response, ask: D17A.

D17A. Was it made available to you (. . . . .)?

	(r) Ref	(x) DK	(3) No	(1) Yes	D17A: (1) Yes	(3) No	(x) DK	(r) Ref
(a) note takers or readers?	◯	◯	◯	◯ →	◯	◯	◯	◯
(b) a tutor or teacher's aide?	◯	◯	◯	◯ →	◯	◯	◯	◯
(c) a computer with Braille, large print or speech access?	◯	◯	◯	◯ →	◯	◯	◯	◯
(d) talking books?	◯	◯	◯	◯ →	◯	◯	◯	◯
(e) magnifiers or CCTV's (Closed circuit television readers)?	◯	◯	◯	◯ →	◯	◯	◯	◯
(f) Braille or large print reading materials?	◯	◯	◯	◯ →	◯	◯	◯	◯
(g) a Sign language interpreter?	◯	◯	◯	◯ →	◯	◯	◯	◯
(h) recording equipment or portable note-takers?	◯	◯	◯	◯ →	◯	◯	◯	◯
(i) attendant care services?	◯	◯	◯	◯ →	◯	◯	◯	◯
(j) other aid or service?	◯	◯	◯	◯ →	◯	◯	◯	◯

Specify ↓

[ ]

D18. Did you (. . . . .) have your (his/her) condition before completing all your (his/her) formal education or training?

(1) Yes . . . . ◯
(3) No . . . . ◯
(x) Don't know . . . . ◯
(r) Refusal . . . . ◯

(3), (x), (r): **Go to D20**

D19. Did you (. . . . .) discontinue your (his/her) formal education or training because of your (his/her) condition?

(1) Yes . . . . ◯
(3) No . . . . ◯
(x) Don't know . . . . ◯
(r) Refusal . . . . ◯

D20. What is the highest level of schooling you have (. . . . . has) completed?

Interviewer: Read list. Mark one only.

(1) No schooling ○
(2) Elementary school ○
(3) Some high (secondary) school ○
(4) High (secondary) school graduation certificate or equivalent ○
(5) Some trade, technical or vocational school; or business college ○
(6) Trades certificate or diploma ○
(7) Some community college, CEGEP or nursing school ○
(8) Diploma or certificate from a community college, CEGEP or nursing school ○
(9) Some university ○
(10) University certificate or diploma **below** bachelor level ○
(11) Bachelor's degree(s) (e.g., B.A., B.Sc., LL.B.) ○
(12) University certificate or diploma **above** bachelor level ○
(13) Master's degree(s) (e.g., M.A., M.Sc., M.Ed.) ○
(14) Degree in medicine, dentistry, veterinary medicine or optometry (e.g., M.D., D.D.S., D.M.D., D.V.M., O.D.) ○
(15) Earned doctorate (e.g., Ph.D., D.Sc., D.Ed.) ○
(x) Don't know ○
(r) Refusal ○

D21. Because of your (. . . . . 's) condition . . .

Interviewer: Read list.

	(1) Yes	(3) No	(x) DK	(r) Ref
(a) did you (he/she) begin school later than most other people your (his/her) age?	○	○	○	○
(b) was your (his/her) education interrupted for long periods of time?	○	○	○	○
(c) did you (he/she) ever attend a special education school or special education classes in a regular school?	○	○	○	○
(d) did you (he/she) take fewer courses or subjects than you (he/she) otherwise would have?	○	○	○	○
(e) did you (he/she) take any courses by correspondence or home study?	○	○	○	○
(f) did you (he/she) have to leave your (his/her) community to attend school?	○	○	○	○
(g) did it take you (him/her) longer to achieve your (his/her) present level of education?	○	○	○	○

D21. edit

Interviewer: If D21(g) is Yes, go to D22. Otherwise, go to Section E (page 44).

D22. How much longer?

Interviewer: Round year to nearest whole number.

☐☐ (1-10) Years

(x) Don't know ○
(r) Refusal ○

SECTION E — EMPLOYMENT STATUS

***Interviewer:* If respondent was born AFTER May 15, 1936, continue. Otherwise, go to Section F (page 65).**

E1. The next few questions will help us establish your (. . . . . 's) employment status.

LAST WEEK, how many hours did you (he / she) spend working for pay OR in self-employment?

Interviewer: Include
- *working for wages, salary, tips or commission;*
- *working in your (his / her) own business, farm or professional practice, alone or in partnership;*
- *working directly towards the operation of a family farm or business without formal pay arrangements (e.g., assisting in seeding, doing accounts).*

(0) None . . . . ◯ → **Go to E2**

Interviewer: Round to the nearest hour.

☐☐☐ (1-168) Hours → **Go to E1edit**

(x) Don't know . . . . ◯
(r) Refusal . . . . ◯
→ **Go to E2**

E1. edit ***Interviewer:* If hours are over 80, confirm response. Otherwise, go to E7.**

E2. LAST WEEK, were you (was . . . . .) on temporary layoff or absent from your (his / her) job or business?

Interviewer: Read list. Mark one only.

(1) Yes, on temporary layoff from a job or business to which you (he / she) expect(s) to return . . . . ◯ → **Go to E4**
(2) Yes, on vacation, ill, on strike or locked out . . . . ◯ → **Go to E7**
(3) Yes, absent for other reasons . . . . ◯
(4) No . . . . ◯
(x) Don't know . . . . ◯
(r) Refusal . . . . ◯
→ **Go to E4**

E3. Why were you (was . . . . .) absent?

Interviewer: Do not read list. Mark one only.

(1) Caring for own children . . . . ◯
(2) Caring for elder relative (60 years of age or older) . . . . ◯
(3) Maternity or parental leave . . . . ◯
(4) Other personal or family responsibilities . . . . ◯
(x) Don't know . . . . ◯
(r) Refusal . . . . ◯
→ **Go to E7**

E4. LAST WEEK, did you (. . . . .) have definite arrangements to start a new job within the next four weeks?

(1) Yes . . . . ◯
(3) No . . . . ◯
(x) Don't know . . . . ◯
(r) Refusal . . . . ◯

E5. **Did you (. . . . .) look for paid work DURING THE PAST FOUR WEEKS? For example, did you (he/she) contact an employment centre, check with employers, place or answer newspaper ads, etc.?**

Interviewer: Read list. Mark one only.

(1) Yes, looked for full-time work . . . . . ◯
(2) Yes, looked for part-time work (less than 30 hours per week) . . . . . ◯
(3) No . . . . . ◯
→ **Go to E6**

(x) Don't know . . . . . ◯
(r) Refusal . . . . . ◯
→ **Go to E5edit**

E5. edit ***Interviewer: If E2 is (1) and E5 is (x) or (r) OR if E4 is Yes and E5 is (x) or (r), go to E49 (page 53). Otherwise, continue.***

E6. **Could you (. . . . .) have started a job last week had one been available?**

Interviewer: Read list. Mark one only.

(1) Yes, could have started a job . . . . . ◯
(2) No, already had a job . . . . . ◯
(3) No, because of temporary illness or disability . . . . . ◯
(4) No, because of personal or family responsibilities . . . . . ◯
→ **Go to E49 (page 53)**

(5) No, going to school . . . . . ◯
(6) No, other reasons (includes retired) . . . . . ◯
(x) Don't know . . . . . ◯
(r) Refusal . . . . . ◯
→ **Go to E73 (page 58)**

Employed

E7. **How many hours do you (does . . . . .) usually work per week?**

Interviewer: Round to the nearest hour.

☐☐☐ (1-168) Hours → **Go to E7edit**

(x) Don't know . . . . ◯
(r) Refusal . . . . ◯
→ **Go to E9**

E7. edit ***Interviewer: If E7 is less than 30 hours, go to E8. If E7 is over 80, confirm hours entered in E7. Otherwise, go to E9.***

E8. **What is the main reason you (. . . . .) usually work(s) less than 30 hours per week?**

Interviewer: Do not read list. Mark one only.

(1) Own illness, condition or disability . . . . . ◯
(2) Caring for own children . . . . . ◯
(3) Caring for elder relative (60 years of age or older) . . . . . ◯
(4) Other personal or family responsibilities . . . . . ◯
(5) Going to school . . . . . ◯
(6) Business conditions . . . . . ◯
(7) Could not find work with 30 or more hours per week . . . . . ◯
(8) Other, specify . . . . . ◯ ↓

[ ]

(x) Don't know . . . . . ◯
(r) Refusal . . . . . ◯

E9. On what date did you (. . . . .) start this job?

☐☐ Day (1-31)

☐☐ Month (1-12)

☐☐☐☐ Year

(x) Don't know . . . . ○
(r) Refusal . . . . ○

E10. In what kind of business, industry or service is this job? For example, a wheat farm, department store, fish plant.

(x) Don't know . . . . ○
(r) Refusal . . . . ○

E11. What is your (. . . . . 's) work or occupation? For example, accountant, secondary school teacher, sales clerk.

(x) Don't know . . . . ○
(r) Refusal . . . . ○

E12. In this work, what are your (his/her) main activities? For example, administering accounts, teaching mathematics, selling men's clothing.

(x) Don't know . . . . ○
(r) Refusal . . . . ○

E13. In this job are you (is . . . . .) mainly . . .

Interviewer: Read list. Mark one only.

(1) working for wages, salary, tips or commission? . . . . ○
(2) working without pay for your (his/her) spouse or another relative in a family farm or business? . . . . ○
(3) self-employed alone or in partnership? . . . . ○
(x) Don't know . . . . ○
(r) Refusal . . . . ○

(2), (3), (x), (r) → **Go to E26**

E14. In this job, are you (is . . . . .) a union member?

(1) Yes . . . . ○ → **Go to E16**
(3) No . . . . ○
(x) Don't know . . . . ○
(r) Refusal . . . . ○

E15. Are you (is . . . . .) covered by a union contract or collective agreement?

(1) Yes . . . . ○
(3) No . . . . ○
(x) Don't know . . . . ○
(r) Refusal . . . . ○

E16. About how many persons are employed at the location where you (. . . . .) now work(s)?

Interviewer: Do not read list. Mark one only.

(1) Less than 20 . . . . ◯
(2) 20 to 99 . . . . ◯
(3) 100 to 500 . . . . ◯
(4) Over 500 . . . . ◯

(x) Don't know . . . . ◯
(r) Refusal . . . . ◯

E17. Does your (. . . . . 's) employer operate at more than one location?

(1) Yes . . . . ◯
(3) No . . . . ◯
(x) Don't know . . . . ◯
(r) Refusal . . . . ◯

(3), (x), (r) ▶ **Go to E19**

E18. In total, about how many persons are employed at all locations?

Interviewer: Do not read list. Mark one only.

(1) Less than 20 . . . . ◯
(2) 20 to 99 . . . . ◯
(3) 100 to 500 . . . . ◯
(4) Over 500 . . . . ◯

(x) Don't know . . . . ◯
(r) Refusal . . . . ◯

E19. At your (. . . . . 's) job, what is your (his/her) rate of pay or salary before deductions (not counting overtime, tips, commissions or bonuses)?

(1) Commissions only . . . . . ◯ → **Go to E22**
(2) Minimum wage . . . . ◯ → **Go to E21**

$ ☐☐☐☐☐☐.☐☐

(x) Don't know . . . . ◯
(r) Refusal . . . . ◯

(x), (r) ▶ **Go to E21**

E20. Is that . . .

Interviewer: Read list. Mark one only.

(1) per hour? . . . . ◯
(2) per day? . . . . ◯
(3) per week? . . . . ◯
(4) bi-weekly? . . . . ◯
(5) twice per month? . . . . . . ◯
(6) per month? . . . . ◯
(7) per year? . . . . ◯

(x) Don't know . . . . ◯
(r) Refusal . . . . ◯

E21. Do you (Does . . . . .) usually receive tips or commissions?

(1) Yes . . . . ◯
(3) No . . . . ◯
(x) Don't know . . . . ◯
(r) Refusal . . . . ◯

(3), (x), (r) ▶ **Go to E24**

E22. What are your (. . . . . 's) usual tips or commissions in that job?

Interviewer: Obtain the estimate for a period of time that is easy to calculate for the respondent.

$ [ ][ ][ ] , [ ][ ][ ] . [ ][ ]

(x) Don't know . . . . ◯
(r) Refusal . . . . ◯
Go to E24

E23. Is that . . .

Interviewer: Read list. Mark one only.

(1) per hour? . . . . ◯
(2) per day? . . . . ◯
(3) per week? . . . . ◯
(4) bi-weekly? . . . . ◯
(5) twice per month? . . . . ◯
(6) per month? . . . . ◯
(7) per year? . . . . ◯
(x) Don't know . . . . ◯
(r) Refusal . . . . ◯

E24. Is your (. . . . . 's) job a permanent job?

Interviewer: It is a permanent job if the employer did not hire the employee on the understanding that the job would last only for a fixed duration, or until a given date or until the end of the project.

(1) Yes . . . . ◯ → **Go to E26**
(3) No . . . . ◯
(x) Don't know . . . . ◯
(r) Refusal . . . . ◯
Go to E26

E25. In what way is your (. . . . . 's) job not permanent?

Interviewer: Do not read list. Mark one only.

(1) It is seasonal . . . . ◯
(2) Temporary, term or contract (non-seasonal) . . . . . . ◯
(3) Casual job . . . . ◯
(4) Work done through a temporary help agency . . . . . ◯
(5) Other, specify . . . . ◯

[ ]

(x) Don't know . . . . ◯
(r) Refusal . . . . ◯

E26. Have you (Has . . . . .) had any periods of unemployment in the last twelve months, that is to say, periods when you were (he/she was) unemployed or did not have a job?

(1) Yes . . . . ◯
(3) No . . . . ◯
(x) Don't know . . . . ◯
(r) Refusal . . . . ◯
Go to E29

E27. How many different periods of unemployment did you (. . . . .) have?

Interviewer: Do not read list. Mark one only.

(1) One ○
(2) Two ○
(3) Three or more ○
(x) Don't know ○ ▶ **Go to E29**
(r) Refusal ○ ▶ **Go to E29**

E28. What was the length of the longest period of unemployment?

Interviewer: Do not read list. Mark one only.

(1) Under three months ○
(2) Three to five months ○
(3) Six months or more ○
(x) Don't know ○
(r) Refusal ○

E29. Because of your (. . . . . 's) condition, have you (has . . . . .) ever:

Interviewer: Read list.

	(1) Yes	(3) No	(x) DK	(r) Ref
(a) changed the kind of work you do (he/she does)?	○	○	○	○
(b) changed the amount of work you do (he/she does)?	○	○	○	○
(c) changed your (his/her) job?	○	○	○	○

E30. Does your (. . . . . 's) condition limit the amount or kind of work you (he/she) can do at your (his/her) present job or business?

(1) Yes ○
(3) No ○ ▶ **Go to E35edit**
(x) Don't know ○ ▶ **Go to E35edit**
(r) Refusal ○ ▶ **Go to E35edit**

E31. Where were you (was . . . . .) employed when you (he/she) first experienced work limitations?

Interviewer: Read list. Mark one only.

(1) Present employer ○
(2) Elsewhere ○
(3) Not working ○ ▶ **Go to E35edit**
(x) Don't know ○ ▶ **Go to E35edit**
(r) Refusal ○ ▶ **Go to E35edit**

E32. Are you (Is . . . . .) now doing the same kind of work as you were (he/she was) doing at the time you (he/she) first experienced work limitations?

(1) Yes ○ → **Go to E34**
(3) No ○
(x) Don't know ○ ▶ **Go to E34**
(r) Refusal ○ ▶ **Go to E34**

E33. Is your (. . . . . 's) condition the reason you are (he/she is) now doing a different kind of work?

(1) Yes ○
(3) No ○
(x) Don't know ○
(r) Refusal ○

E34. Because of your (. . . . . 's) condition, would you say that you are (he/she is) now doing:

Interviewer: Read list. Mark one only.

(1) about the same amount of work? .... ◯
(2) more work now? .... ◯ ▶ **Go to E35edit**
(3) less work now? .... ◯

(x) Don't know .... ◯
(r) Refusal .... ◯ ▶ **Go to E35edit**

E35. Is your (. . . . . 's) condition the reason you are (he/she is) doing less?

(1) Yes .... ◯
(3) No .... ◯

(x) Don't know .... ◯
(r) Refusal .... ◯

E35. edit ***Interviewer: If E13 is (1) (on page 46), go to E36. Otherwise, go to E40.***

E36. Do you (Does . . . . .) believe that your (his/her) condition makes it difficult for you (him/her) to change jobs or to advance at your (his/her) present job?

Interviewer: Read list. Mark one only.

(1) Yes, very dfficult .... ◯
(2) Yes, difficult .... ◯
(3) No, not difficult .... ◯

(x) Don't know .... ◯
(r) Refusal .... ◯

E37. Does your (. . . . . 's) job give you (him/her) the opportunity to use all your (his/her) education, skills or work experience?

(1) Yes .... ◯
(3) No .... ◯

(x) Don't know .... ◯
(r) Refusal .... ◯

E38. Does your (. . . . . 's) job require the level of education you have (he/she has)?

(1) Yes .... ◯
(3) No .... ◯

(x) Don't know .... ◯
(r) Refusal .... ◯

E39. Because of your (his/her) condition, do you (does . . . . .) require any of the following to be able to work?

Interviewer: Read list.
For each "Yes" response, ask: E39A.

E39A. Has this been made available to you (him/her)?

	(r) Ref	(x) DK	(3) No	(1) Yes		E39A (1) Yes	E39A (3) No	E39A (x) DK	E39A (r) Ref
(a) Job redesign (modified or different duties)	○	○	○	○	→	○	○	○	○
(b) Modified hours or days or reduced work hours	○	○	○	○	→	○	○	○	○
(c) Human support, such as a reader, Sign language interpreter, job coach or personal assistant	○	○	○	○	→	○	○	○	○
(d) Technical aids, such as a voice synthesizer, a TTY or TDD, an infrared system or portable note-takers	○	○	○	○	→	○	○	○	○
(e) A computer with Braille, large print or speech access, or a scanner	○	○	○	○	→	○	○	○	○
(f) Communication aids, such as Braille or large print reading material or recording equipment	○	○	○	○	→	○	○	○	○
(g) Other equipment, help or work arrangement	○	○	○	○	→	○	○	○	○

Specify ↓

____

E40. Because of your (his/her) condition, do you (does . . . . .) require any of the following to be able to work?

Interviewer: Read list.
For each "Yes" response, ask: E40A.

E40A. Has this been made available to you (. . . . .)?

	(r) Ref	(x) DK	(3) No	(1) Yes		E40A (1) Yes	E40A (3) No	E40A (x) DK	E40A (r) Ref
(a) Handrails, ramps	○	○	○	○	→	○	○	○	○
(b) Appropriate parking	○	○	○	○	→	○	○	○	○
(c) Accessible elevator	○	○	○	○	→	○	○	○	○
(d) Modified workstation	○	○	○	○	→	○	○	○	○
(e) Accessible washrooms	○	○	○	○	→	○	○	○	○
(f) Accessible transportation	○	○	○	○	→	○	○	○	○
(g) Other	○	○	○	○	→	○	○	○	○

Specify ↓

____

E41. In the past five years, have you (has . . . . .) taken any work-related training courses to either improve your (his/her) skills or to learn new skills?

- (1) Yes . . . . ○
- (3) No . . . . ○ ▶ **Go to E44**
- (x) Don't know . . . . ○ ▶ **Go to E44**
- (r) Refusal . . . . ○ ▶ **Go to E44**

E42. What was the main reason you (. . . . .) took this course? Was it . . .

Interviewer: Read list. Mark one only.

- (1) for your (his/her) current or a future job? ○
- (2) because of your (his/her) condition? ○
- (3) for personal interest? ○
- (4) for another reason? ○
- (x) Don't know ○
- (r) Refusal ○

E43. At work, to what extent are you (is he/she) using the skills or knowledge acquired in this course?

Interviewer: Read list. Mark one only.

- (1) To a great extent ○
- (2) Somewhat ○
- (3) Very little ○
- (4) Not at all ○
- (x) Don't know ○
- (r) Refusal ○

Go to E46

E44. Did you (. . . . .) want to take some work-related training courses?

- (1) Yes ○
- (3) No ○
- (x) Don't know ○
- (r) Refusal ○

(3), (x), (r): **Go to E46**

E45. Did any of the following prevent you (. . . . .) from taking work-related training courses? I will read you a list. Please answer yes or no to each.

Interviewer: Read list.

	(1) Yes	(3) No	(x) DK	(r) Ref
(a) Location was not physically accessible to you (him/her)	○	○	○	○
(b) Courses were not adapted to your (his/her) needs	○	○	○	○
(c) You (He/She) requested courses, but were denied them (by employer)	○	○	○	○
(d) Your (His/Her) condition	○	○	○	○
(e) Inadequate transportation	○	○	○	○
(f) Too costly	○	○	○	○
(g) Other reason, specify	○	○	○	○

E46. In the past five years, do you (does .....) believe that because of your (his/her) condition, you have (he/she has) been refused ...

Interviewer: Read list.
If answer categories are not applicable, enter "No".
For each "Yes" response, ask: E46A.

E46A. How many times?

Interviewer: Do not read list.
Mark one only.

	(r) Ref	(x) DK	(3) No	(1) Yes		(1) Once	(2) 2 to 4 times	(3) More than 4 times	(x) DK	(r) Ref
(a) employment?	◯	◯	◯	◯	→	◯	◯	◯	◯	◯
(b) a promotion?	◯	◯	◯	◯	→	◯	◯	◯	◯	◯
(c) access to training programs?	◯	◯	◯	◯	→	◯	◯	◯	◯	◯
(d) or, has your (his/her) employment been terminated?	◯	◯	◯	◯	→	◯	◯	◯	◯	◯

E47. Do you (Does .....) consider yourself (himself/herself) to be disadvantaged in employment because of your (his/her) condition?

(1) Yes .... ◯
(3) No .... ◯
(x) Don't know ...... ◯
(r) Refusal .... ◯

E48. Do you (Does .....) believe that your (his/her) current employer or any potential employer would be likely to consider you (him/her) disadvantaged in employment because of your (his/her) condition?

(1) Yes .... ◯
(3) No .... ◯
(x) Don't know ...... ◯
(r) Refusal .... ◯

▶ **Go to Section F (page 65)**

Unemployed

E49. When did you (.....) last work, even for a few days? Include as work, working without pay at a family farm or business. Do not include volunteer work, housework, maintenance or repairs for your (his/her) own home.

(1) Never worked .... ◯ → **Go to E58**

Year ☐☐☐☐ → **Go to E49edit**

(x) Don't know ...... ◯
(r) Refusal .... ◯

▶ **Go to E50**

E49. edit

Interviewer: If E49 is before 1996, go to E58. Otherwise, continue.

E50. When you (.....) last worked, how many hours did you (he/she) usually work per week?

Interviewer: Round to the nearest hour.

☐☐☐ (1-168) Hours → **Go to E50edit**

(x) Don't know ...... ◯
(r) Refusal .... ◯

▶ **Go to E51**

E50. edit ***Interviewer: If hours are over 80, confirm response. Otherwise, continue.***

E51. What kind of business, industry or service was this? For example, a wheat farm, department store, fish plant.

(x) Don't know ...... ○
(r) Refusal .... ○

E52. What was your (. . . . . 's) work or occupation? For example, accountant, secondary school teacher, sales clerk.

(x) Don't know ...... ○
(r) Refusal .... ○

E53. In this work, what were your (. . . . . 's) main activities? For example, administering accounts, teaching mathematics, selling men's clothing.

(x) Don't know ...... ○
(r) Refusal .... ○

E54. In that job, were you (was . . . . .) mainly . . .

Interviewer: Read list. Mark one only.

(1) working for wages, salary, tips or commission? .... ○
(2) working without pay for spouse or another relative in a family farm or business? .... ○
(3) self-employed alone or in partnership? .... ○
(x) Don't know .... ○
(r) Refusal .... ○

E54. edit ***Interviewer: If E49 is after 1999, go to E55. Otherwise, go to E58.***

E55. Have you (Has . . . . .) had any periods of employment in the last twelve months; that is to say, periods when you (he / she) had a job?

(1) Yes .... ○
(3) No .... ○
(x) Don't know ...... ○
(r) Refusal .... ○

(3), (x), (r): **Go to E58**

E56. How many different periods of employment did you (. . . . .) have?

Interviewer: Do not read list. Mark one only.

(1) One .... ○
(2) Two .... ○
(3) Three or more .... ○
(x) Don't know ...... ○
(r) Refusal .... ○

(x), (r): **Go to E58**

E57. What was the length of the longest period of employment?

Interviewer: Do not read list. Mark one only.

(1) Under three months . . . . ◯
(2) Three to five months . . . . ◯
(3) Six months or more . . . . ◯
(x) Don't know . . . . ◯
(r) Refusal . . . . ◯

E58. Does your (. . . . . 's) condition limit the amount or kind of work you (he/she) can do at a job or business?

(1) Yes . . . . ◯ → **Go to E58edit**
(3) No . . . . ◯ **Go to E60**
(x) Don't know . . . . ◯ **Go to E60**
(r) Refusal . . . . ◯ **Go to E60**

E58. edit ***Interviewer: If E49 is (1), go to E60. Otherwise, continue.***

E59. Were you (Was . . . . .) working at a job or business at the time you (he/she) became limited in the kind or amount of work you (he/she) can do?

(1) Yes . . . . ◯
(3) No . . . . ◯
(x) Don't know . . . . ◯
(r) Refusal . . . . ◯

E60. Does your (. . . . . 's) condition affect your (his/her) ability to look for work?

(1) Yes . . . . ◯
(3) No . . . . ◯
(x) Don't know . . . . ◯
(r) Refusal . . . . ◯

E61. Would you (. . . . .) prefer to work . . .

Interviewer: Read list. Mark one only.

(1) either full-time or part-time? . . . . . ◯ **Go to E63**
(2) full-time only? . . . . ◯ **Go to E63**
(3) part-time only? . . . . ◯
(x) Don't know . . . . ◯ **Go to E63**
(r) Refusal . . . . ◯ **Go to E63**

E62. Because of your (. . . . . 's) condition, are you (is he/she) limited in your (his/her) ability to:

Interviewer: Read list.

	(1) Yes	(3) No	(x) DK	(r) Ref
(a) work at a full-time job?	◯	◯	◯	◯
(b) work at a part-time job?	◯	◯	◯	◯

E63. Because of your (his/her) condition, do you (does .....) require any of the following to be able to work? I will read you a list. Please answer yes or no to each.

Interviewer: Read list.

	(1) Yes	(3) No	(x) DK	(r) Ref
(a) Job redesign (modified or different duties)	○	○	○	○
(b) Modified hours or days or reduced work hours	○	○	○	○
(c) Human support such as a reader, Sign language interpreter, job coach or personal assistant	○	○	○	○
(d) Technical aids, such as a voice synthesizer, a TTY or TDD, an infrared system or portable note-takers	○	○	○	○
(e) A computer with Braille, large print or speech access, or a scanner	○	○	○	○
(f) Communication aids, such as Braille or large print reading material or recording equipment	○	○	○	○
(g) Other equipment, help or work arrangement, specify	○	○	○	○

E64. Do you (Does .....) require modified features or arrangements in the workplace, such as:

Interviewer: Read list.

	(1) Yes	(3) No	(x) DK	(r) Ref
(a) handrails, ramps?	○	○	○	○
(b) appropriate parking?	○	○	○	○
(c) accessible elevator?	○	○	○	○
(d) modified workstation?	○	○	○	○
(e) accessible washrooms?	○	○	○	○
(f) accessible transportation?	○	○	○	○
(g) Other, specify	○	○	○	○

E65. In the past five years, have you (has .....) taken any work-related training courses to either improve your (his/her) skills or to learn new skills?

(1) Yes .... ○
(3) No .... ○
(x) Don't know .... ○
(r) Refusal .... ○

Go to E68 (for No, Don't know, Refusal)

E66. What was the main reason you (.....) took the course? Was it ...

Interviewer: Read list. Mark one only.

(1) for your (his/her) job or a future job? .... ○
(2) because of your (his/her) condition? .... ○
(3) for personal interest? .... ○
(4) for another reason? .... ○
(x) Don't know .... ○
(r) Refusal .... ○

E67. At work, to what extent were you (was he/she) using the skills or knowledge acquired in the course?

Interviewer: Read list. Mark one only.

- (1) To a great extent .... ○
- (2) Somewhat .... ○
- (3) Very little .... ○
- (4) Not at all .... ○
- (x) Don't know .... ○
- (r) Refusal .... ○

Go to E70

E68. Did you (.....) want to take some work-related training courses?

- (1) Yes .... ○
- (3) No .... ○ — **Go to E70**
- (x) Don't know .... ○ — **Go to E70**
- (r) Refusal .... ○ — **Go to E70**

E69. Did any of the following prevent you (.....) from taking those courses?

Interviewer: Read list.

	(1) Yes	(3) No	(x) DK	(r) Ref
(a) Location was not physically accessible to you (him/her)	○	○	○	○
(b) Courses were not adapted to your (his/her) needs	○	○	○	○
(c) You (He/She) requested courses, but were denied them (by employer)	○	○	○	○
(d) Your (His/Her) condition	○	○	○	○
(e) Inadequate transportation	○	○	○	○
(f) Too costly	○	○	○	○
(g) Other reason, specify	○ ↓	○	○	○

[ ]

E70. In the past five years, do you (does .....) believe that because of your (his/her) condition, you have (he/she has) been refused . . .

Interviewer: Read list.
If answer categories are not applicable, enter "No".
For each "Yes" response, ask: E70A.

E70A. How many times?

Interviewer: Do not read list. Mark one only.

	(r) Ref	(x) DK	(3) No	(1) Yes		E70A (1) Once	(2) 2 to 4 times	(3) More than 4 times	(x) DK	(r) Ref
(a) employment?	○	○	○	○	→	○	○	○	○	○
(b) a promotion?	○	○	○	○	→	○	○	○	○	○
(c) access to training programs?	○	○	○	○	→	○	○	○	○	○
(d) or, has your (his/her) employment been terminated?	○	○	○	○	→	○	○	○	○	○

E71. Would you (.....) consider yourself (himself/herself) to be disadvantaged in employment because of your (his/her) condition, if you (he/she) were employed?

- (1) Yes .... ○
- (3) No .... ○
- (x) Don't know .... ○
- (r) Refusal .... ○

E72. Do you (Does . . . . .) believe that any potential employer would be likely to consider you (him/her) disadvantaged in employment because of your (his/her) condition?

(1) Yes . . . . ◯
(3) No . . . . ◯
(x) Don't know . . . . . . ◯
(r) Refusal . . . . ◯

Go to Section F (page 65)

Not in the Labour Force

E73. When did you (. . . . .) last work, even for a few days? Include as work, working without pay at a family farm or business. Do not include volunteer work, housework, maintenance or repairs for your (his/her) own home.

☐☐☐☐ Year

(1) Never worked . . . . ◯ → **Go to E81**
(x) Don't know . . . . . . ◯
(r) Refusal . . . . ◯

E74. Are you (Is . . . . .) permanently retired?

Interviewer: If the respondent was retired in September, 2001 and did not work at any time between January 1, 2000 and September, 2001, mark "Yes". Some retired persons may have returned to the workforce following official retirement. If the respondent returned to the work force between January 1, 2000 and September, 2001, mark "No".

(1) Yes . . . . ◯ → **Go to E75**
(3) No . . . . ◯
(x) Don't know . . . . . . ◯
(r) Refusal . . . . ◯

E74. edit

Interviewer: If E73 is before 1996, go to E81. Otherwise, go to E76.

E75. Is that because of your (his/her) condition?

(1) Yes . . . . ◯
(3) No . . . . ◯
(x) Don't know . . . . . . ◯
(r) Refusal . . . . ◯

Go to E101 (page 63)

E76. When you (. . . . .) last worked, how many hours did you (he/she) usually work per week?

Interviewer: Round to the nearest hour.

☐☐☐ (1-168) Hours → **Go to E76edit**

(x) Don't know . . . . . . ◯
(r) Refusal . . . . ◯

E76. edit

Interviewer: If hours are over 80, confirm response. Otherwise, continue.

E77. **What kind of business, industry or service was this? For example, a wheat farm, department store, fish plant.**

(x) Don't know . . . . ○
(r) Refusal . . . . ○

E78. **What was your (. . . . . 's) work or occupation? For example, accountant, secondary school teacher, sales clerk.**

(x) Don't know . . . . ○
(r) Refusal . . . . ○

E79. **In this work, what were your (. . . . . 's) main activities? For example, administering accounts, teaching mathematics, selling men's clothing.**

(x) Don't know . . . . ○
(r) Refusal . . . . ○

E80. **In that job, were you (was . . . . .) mainly** . . .

Interviewer: Read list. Mark one only.

(1) working for wages, salary, tips or commission? . . . . ○
(2) working without pay for spouse or another relative in a family farm or business? . . . . ○
(3) self-employed alone or in partnership? . . . . ○
(x) Don't know . . . . ○
(r) Refusal . . . . ○

E81. **Does your (. . . . . 's) condition completely prevent you (him/her) from working at a job or business?**

(1) Yes . . . . ○ → **Go to E86**
(3) No . . . . ○
(x) Don't know . . . . ○
(r) Refusal . . . . ○

E82. **Does your (. . . . . 's) condition limit the amount or kind of work you (he/she) could do at a job or business?**

(1) Yes . . . . ○
(3) No . . . . ○
(x) Don't know . . . . ○
(r) Refusal . . . . ○

E82. edit ***Interviewer: If E73 is (1), go to E84. Otherwise, continue.***

E83. **Were you (Was . . . . .) working at a job or business at the time you (he/she) became limited in the amount or kind of work you (he/she) can do?**

(1) Yes . . . . ○
(3) No . . . . ○
(x) Don't know . . . . ○
(r) Refusal . . . . ○

E84. Does your (. . . . . 's) condition affect your (his/her) ability to look for work?

(1) Yes .... ◯
(3) No .... ◯
(x) Don't know .... ◯
(r) Refusal .... ◯

E85. Have you (Has . . . . .) looked for work in the past two years?

(1) Yes .... ◯
(3) No .... ◯
(x) Don't know .... ◯
(r) Refusal .... ◯

E86. Some people have encountered barriers which have discouraged them from looking for work. Could you (. . . . .) think about your (his/her) own situation and indicate which of the following situations might apply to you (him/her)? Please answer yes or no to each of the statements.

Interviewer: Read list.

	(1) Yes	(3) No	(x) DK	(r) Ref
(a) You (He/She) would lose some or all of your (his/her) current income if you (he/she) went to work	◯	◯	◯	◯
(b) You (He/She) would lose some or all of your (his/her) current additional supports such as your (his/her) drug plan or housing if you (he/she) went to work	◯	◯	◯	◯
(c) Your (His/Her) family or friends have discouraged your (him/her) going to work	◯	◯	◯	◯
(d) Family responsibilities prevent you (him/her)	◯	◯	◯	◯
(e) Information about jobs is not accessible to you (him/her)	◯	◯	◯	◯
(f) You (He/She) worry(ies) about being isolated by other workers on the job	◯	◯	◯	◯
(g) You have (He/She has) been a victim of discrimination	◯	◯	◯	◯
(h) You feel (He/She feels) your (his/her) training is not adequate	◯	◯	◯	◯
(i) Lack of accessible transportation	◯	◯	◯	◯
(j) No jobs available	◯	◯	◯	◯
(k) Other reason, specify	◯ ↓	◯	◯	◯

[ ]

E87. If you were (. . . . . was) to find employment, would you (he/she) prefer to work:

Interviewer: Read list. Mark one only.

(1) either full-time or part-time? ..... ◯ ▶ **Go to E89**
(2) full-time only? .... ◯ ▶ **Go to E89**
(3) part-time only? .... ◯
(x) Don't know .... ◯ ▶ **Go to E89**
(r) Refusal .... ◯ ▶ **Go to E89**

E88. Is this because of your (his/her) condition?

(1) Yes .... ◯
(3) No .... ◯
(x) Don't know .... ◯
(r) Refusal .... ◯

E89. Do you (Does . . . . .) think you (he/she) will look for work at any time in the next twelve months?

(1) Yes . . . . ○

(3) No . . . . ○ } **Go to E91**

(x) Don't know . . . . ○ } **Go to E91**

(r) Refusal . . . . ○ } **Go to E91**

E90. Is this:

Interviewer: Read list.

	(1) Yes	(3) No	(x) DK	(r) Ref
(a) because you (. . . . .) expect(s) your (his/her) condition to improve?	○	○	○	○
(b) because there will be changes or improvements in the workplace?	○	○	○	○
(c) because you (. . . . .) will be taking training?	○	○	○	○
(d) because of another reason? — Specify	○	○	○	○

E91. Because of your (. . . . . 's) condition, would you (he/she) require any of the following to be able to work? I will read you a list. Please answer yes or no to each.

Interviewer: Read list.

	(1) Yes	(3) No	(x) DK	(r) Ref
(a) Job redesign (modified or different duties)	○	○	○	○
(b) Modified hours or days or reduced work hours	○	○	○	○
(c) Human support such as a reader, Sign language interpreter, job coach or personal assistant	○	○	○	○
(d) Technical aids, such as a voice synthesizer, a TTY or TDD, an infrared system or portable note-takers	○	○	○	○
(e) A computer with Braille, large print or speech access, or a scanner	○	○	○	○
(f) Communication aids, such as Braille or large print reading material or recording equipment	○	○	○	○
(g) Other equipment, help or work arrangement, specify	○	○	○	○

E92. Because of your (. . . . . 's) condition, would you (he/she) require modified features or arrangements in the workplace, such as:

Interviewer: Read list.

	(1) Yes	(3) No	(x) DK	(r) Ref
(a) handrails, ramps?	○	○	○	○
(b) appropriate parking?	○	○	○	○
(c) accessible elevator?	○	○	○	○
(d) modified workstation?	○	○	○	○
(e) accessible washrooms?	○	○	○	○
(f) accessible transportation?	○	○	○	○
(g) Other, specify	○	○	○	○

E93. In the past five years, have you (has . . . . .) taken any work-related training courses to either improve your (his/her) skills or to learn new skills?

(1) Yes . . . . ◯

(3) No . . . . ◯

(x) Don't know . . . . ◯

(r) Refusal . . . . ◯

Go to E96 (No, Don't know, Refusal)

E94. What was the main reason you (. . . . .) took the course? Was it . . .

Interviewer: Read list. Mark one only.

(1) for your (his/her) job or a future job? . . . . ◯

(2) because of your (his/her) condition? . . . . ◯

(3) for personal interest? . . . . ◯

(4) for another reason? . . . . ◯

(x) Don't know . . . . ◯

(r) Refusal . . . . ◯

E95. To what extent were you (was . . . . .) using the skills or knowledge acquired in the course at work?

Interviewer: Read list.

(1) To a great extent . . . . ◯

(2) Somewhat . . . . ◯

(3) Very little . . . . ◯

(4) Not at all . . . . ◯

(x) Don't know . . . . ◯

(r) Refusal . . . . ◯

Go to E97

E96. Did you (. . . . .) want to take some work-related training courses?

(1) Yes . . . . ◯

(3) No . . . . ◯

(x) Don't know . . . . ◯

(r) Refusal . . . . ◯

Go to E98 (No, Don't know, Refusal)

E97. Did any of the following prevent you (. . . . .) from taking those courses?

Interviewer: Read list.

	(1) Yes	(3) No	(x) DK	(r) Ref
(a) Location was not physically accessible to you (him/her)	◯	◯	◯	◯
(b) Courses were not adapted to your (his/her) needs	◯	◯	◯	◯
(c) You (He/She) requested courses, but were denied them (by employer)	◯	◯	◯	◯
(d) Your (His/Her) condition	◯	◯	◯	◯
(e) Inadequate transportation	◯	◯	◯	◯
(f) Too costly	◯	◯	◯	◯
(g) Other reason, specify	◯	◯	◯	◯

[ ]

E98. In the past five years, do you (does . . . . .) believe that because of your (his/her) condition or health problem, you have (he/she has) been refused . . .

Interviewer: Read list.
If answer categories are not applicable, enter "No".
For each "Yes" response, ask: E98A.

E98A. How many times?

Interviewer: Do not read list.
Mark one only.

	(r) Ref	(x) DK	(3) No	(1) Yes		(1) Once	(2) 2 to 4 times	(3) More than 4 times	(x) DK	(r) Ref
(a) employment?	○	○	○	○	→	○	○	○	○	○
(b) a promotion?	○	○	○	○	→	○	○	○	○	○
(c) access to training programs?	○	○	○	○	→	○	○	○	○	○
(d) or, has your (his/her) employment been terminated?	○	○	○	○	→	○	○	○	○	○

E99. Would you (. . . . .) consider yourself (himself/herself) to be disadvantaged in employment because of your (his/her) condition, if you (he/she) were employed?

(1) Yes . . . . ○
(3) No . . . . ○
(x) Don't know . . . . ○
(r) Refusal . . . . ○

E100. Do you (Does . . . . .) believe that any potential employer would be likely to consider you (him/her) disadvantaged in employment because of your (his/her) condition?

(1) Yes . . . . ○
(3) No . . . . ○
(x) Don't know . . . . ○
(r) Refusal . . . . ○

Go to Section F (page 65)

Retired

E101. Does your (. . . . . 's) condition completely prevent you (him/her) from working at a job or business?

(1) Yes . . . . ○ → **Go to E103**
(3) No . . . . ○
(x) Don't know . . . . ○
(r) Refusal . . . . ○

E102. Does your (. . . . . 's) condition limit the amount or kind of work you (he/she) could do at a job or business?

(1) Yes . . . . ○
(3) No . . . . ○
(x) Don't know . . . . ○
(r) Refusal . . . . ○

E103. Some people have encountered barriers which have discouraged them from looking for work. Could you (. . . . .) think about your (his/her) own situation and indicate which of the following situations might apply to you (him/her)? Please answer yes or no to each of the statements.

Interviewer: Read list.

	(1) Yes	(3) No	(x) DK	(r) Ref
(a) You (He/She) would lose some or all of your (his/her) current income if you (he/she) went to work	○	○	○	○
(b) You (He/She) would lose some or all of your (his/her) current additional supports such as your (his/her) drug plan or housing if you (he/she) went to work	○	○	○	○
(c) Your (His/Her) family or friends have discouraged your (him/her) going to work	○	○	○	○
(d) Family responsibilities prevent you (him/her)	○	○	○	○
(e) Information about jobs is not accessible to you (him/her)	○	○	○	○
(f) You (He/She) worry(ies) about being isolated by other workers on the job	○	○	○	○
(g) You have (He/She has) been a victim of discrimination	○	○	○	○
(h) You (He/She) feel(s) your (his/her) training is not adequate	○	○	○	○
(i) Lack of accessible transportation	○	○	○	○
(j) No jobs available	○	○	○	○
(k) Other reason, specify	○	○	○	○

[ ]

E104. Because of your (. . . . . 's) condition, would you (he/she) require any of the following to be able to work? I will read you a list. Please answer yes or no to each.

Interviewer: Read list.

	(1) Yes	(3) No	(x) DK	(r) Ref
(a) Job redesign (modified or different duties)	○	○	○	○
(b) Modified hours or days or reduced work hours	○	○	○	○
(c) Human support such as a reader, Sign language interpreter, job coach or personal assistant	○	○	○	○
(d) Technical aids, such as a voice synthesizer, a TTY or TDD, an infrared system or portable note-takers	○	○	○	○
(e) A computer with Braille, large print or speech access, or a scanner	○	○	○	○
(f) Communication aids, such as Braille or large print reading material or recording equipment	○	○	○	○
(g) Other equipment, help or work arrangement, specify	○	○	○	○

[ ]

E105. Because of your (. . . . . 's) condition, would you (he/she) require modified features or arrangements in the workplace, such as:

Interviewer: Read list.

	(1) Yes	(3) No	(x) DK	(r) Ref
(a) handrails, ramps?	○	○	○	○
(b) appropriate parking?	○	○	○	○
(c) accessible elevator?	○	○	○	○
(d) modified workstation?	○	○	○	○
(e) accessible washrooms?	○	○	○	○
(f) accessible transportation?	○	○	○	○
(g) Other, specify	○	○	○	○

[ ]

E106. In the past five years, do you (does . . . . .) believe that because of your (his/her) condition, you have (he/she has) been refused . . .

Interviewer: Read list.
If answer categories are not applicable, enter "No".
For each "Yes" response, ask: E106A.

E106A. How many times?

Interviewer: Do not read list.
Mark one only.

	(r) Ref	(x) DK	(3) No	(1) Yes		(1) Once	(2) 2 to 4 times	(3) More than 4 times	(x) DK	(r) Ref
(a) employment?	◯	◯	◯	◯	→	◯	◯	◯	◯	◯
(b) a promotion?	◯	◯	◯	◯	→	◯	◯	◯	◯	◯
(c) access to training programs?	◯	◯	◯	◯	→	◯	◯	◯	◯	◯
(d) or, has your (his/her) employment been terminated?	◯	◯	◯	◯	→	◯	◯	◯	◯	◯

E107. Would you (. . . . .) consider yourself (himself/herself) to be disadvantaged in employment because of your (his/her) condition, if you (he/she) were employed?

(1) Yes . . . . ◯
(3) No . . . . ◯
(x) Don't know . . . . ◯
(r) Refusal . . . . ◯

E108. Do you (Does . . . . .) believe that any potential employer would be likely to consider you (him/her) disadvantaged in employment because of your (his/her) condition?

(1) Yes . . . . ◯
(3) No . . . . ◯
(x) Don't know . . . . ◯
(r) Refusal . . . . ◯

SECTION F — SOCIAL PARTICIPATION

F1. This section will collect information on your (. . . . . 's) day to day activities such as leisure and recreation, unpaid voluntary activities, local and long distance transportation and housing facilities.

I'll start with a few questions concerning your (. . . . . 's) health in general.

In general, would you say your (. . . . . 's) health is:

Interviewer: Read list. Mark one only.

(1) excellent? . . . . . ◯
(2) very good? . . . . ◯
(3) good? . . . . ◯
(4) fair? . . . . ◯
(5) poor? . . . . ◯
(x) Don't know . . . . ◯
(r) Refusal . . . . ◯

F2. Do you (Does . . . . .) smoke cigarettes?

Interviewer: Read list. Mark one only.

(1) Not at all . . . . ◯
(2) Regularly, that is usually every day . . . . ◯
(3) Occasionally, not every day? . . . . ◯
(x) Don't know . . . . ◯
(r) Refusal . . . . ◯

F3. Now I would like to ask you (. . . . .) a question about alcohol consumption. When I use the word drink, it means one beer, one small glass of wine or 1½ ounces of liquor.

In the past twelve months, how often have you (has . . . . .) had a drink?

Interviewer: Read list.

- (1) Never ◯
- (2) Every day ◯
- (3) 4 to 6 times a week ◯
- (4) 2 to 3 times a week ◯
- (5) Once a week ◯
- (6) Once or twice a month ◯
- (7) Less than once a month ◯
- (x) Don't know ◯
- (r) Refusal ◯

Leisure and Recreation

F4. Now I will ask you (. . . . .) some questions about activities you do (he/she does) in your (his/her) spare time.

In the past 12 months did you (. . . . .) do any of the following activities WITHIN YOUR (HIS/HER) HOME?

Interviewer: Read categories. Mark one only.

Interviewer: Read list.	(1) Every-day	(2) At least once a week	(3) At least once a month	(4) Less than once a month	(5) Never	(x) DK	(r) Ref
(a) exercise	◯	◯	◯	◯	◯	◯	◯
(b) stay in touch by email with family or friends	◯	◯	◯	◯	◯	◯	◯
(c) participate in electronic news groups or chat groups	◯	◯	◯	◯	◯	◯	◯
(d) surf the internet for information or e-commerce	◯	◯	◯	◯	◯	◯	◯
(e) do arts, crafts or hobbies within the home	◯	◯	◯	◯	◯	◯	◯

F5. How often do you (does he/she):

Interviewer: Read categories. Mark one only.

Interviewer: Read list.	(1) Every-day	(2) At least once a week	(3) At least once a month	(4) Less than once a month	(5) Never	(x) DK	(r) Ref
(a) watch TV or videos, listen to radio or CD's?	◯	◯	◯	◯	◯	◯	◯

(1) → **How many hours a day?** ☐☐

Interviewer: Round to the nearest full hour.

(b) read?	◯	◯	◯	◯	◯	◯	◯

(1) → **How many hours a day?** ☐☐

Interviewer: Round to the nearest full hour.

(c) talk on the telephone with family or friends?	◯	◯	◯	◯	◯	◯	◯

(1) → **How many hours a day?** ☐☐

Interviewer: Round to the nearest full hour.

F6. In the past 12 months, how often did you (. . . . .) participate in any of the following activities OUTSIDE YOUR (HIS/HER) HOME?

Interviewer: Read categories. Mark one only.

Interviewer: Read list.

	(1) Every-day	(2) At least once a week	(3) At least once a month	(4) Less than once a month	(5) Never	(x) DK	(r) Ref
(a) visit family or friends	○	○	○	○	○	○	○
(b) do physical activities such as exercise, walk or play sports	○	○	○	○	○	○	○
(c) do hobbies outside the home such as playing cards, bridge or bingo	○	○	○	○	○	○	○
(d) shop	○	○	○	○	○	○	○
(e) attend sporting or cultural events, such as plays or movies		○	○	○	○	○	○
(f) take personal interest courses	○	○	○	○	○	○	○
(g) visit museums, libraries or national or provincial parks		○	○	○	○	○	○
(h) travel for business or personal reasons		○	○	○	○	○	○

F7. Would you (. . . . .) like to do more activities during your (his/her) spare time?

(1) Yes . . . . ○
(3) No . . . . ○
(x) Don't know . . . . ○
(r) Refusal . . . . ○

Go to F9 (No, Don't know, Refusal)

F8. What PREVENTS you (. . . . .) from doing more leisure activities?

Interviewer: Read list.

	(1) Yes	(3) No	(x) DK	(r) Ref
(a) Your (His/Her) condition prevents you (him/her) from doing more	○	○	○	○
(b) You need (He/She needs) specialized aid(s) or equipment that you don't (he/she doesn't) have	○	○	○	○
(c) You need (He/She needs) someone's assistance	○	○	○	○
(d) Your (His/Her) transportation services are inadequate or not accessible	○	○	○	○
(e) Your (His/Her) community has no facilities or programs available	○	○	○	○
(f) The facilities, equipment or programs are not accessible	○	○	○	○
(g) It is too expensive	○	○	○	○
(h) Other, specify	○	○	○	○

[ ]

F9. Does the design and layout of buildings and places in your (. . . . . 's) community make it DIFFICULT for you (him/her) to participate in leisure activities?

(1) Yes . . . . ○
(3) No . . . . ○
(x) Don't know . . . . ○
(r) Refusal . . . . ○

Go to F12 (No, Don't know, Refusal)

F10. In the past 12 months, how often has the design and layout of buildings and places made it DIFFICULT for you (. . . . .) to participate in leisure activities?

Interviewer: Read list. Mark one only.

(1) Daily . . . . ◯
(2) Weekly . . . . ◯
(3) Monthly . . . . ◯
(4) Less than once a month . . . . ◯

(x) Don't know . . . . ◯
(r) Refusal . . . . ◯

F11. When this problem occurred, was it a big problem or a little problem?

Interviewer: Read list. Mark one only.

(1) Big problem . . . . ◯
(2) Little problem . . . . ◯

(x) Don't know . . . . ◯
(r) Refusal . . . . ◯

Unpaid Volunteer Activities

F12. The next question is about UNPAID VOLUNTEER activities which you (. . . . .) may have participated in.

In the past 12 months, did you (. . . . .):

Interviewer: Read list. Mark all that apply.

	(1) Yes	(3) No	(x) DK	(r) Ref
(a) help to organize or supervise activities or events for an organization?	◯	◯	◯	◯
(b) canvass, campaign or fund raise as an unpaid volunteer?	◯	◯	◯	◯
(c) sit as an unpaid member of a board or committee?	◯	◯	◯	◯
(d) do any consulting, executive, office or administrative work as a volunteer?	◯	◯	◯	◯
(e) provide information, help to educate, lobby or influence public opinion on behalf of an organization?	◯	◯	◯	◯
(f) teach, coach, provide care or friendly visits through an organization?	◯	◯	◯	◯
(g) collect, serve or deliver food or other goods as a volunteer through an organization?	◯	◯	◯	◯
(h) do any other unpaid volunteer activities (including help given to schools, religious organizations and community organizations)?	◯	◯	◯	◯

Local Transportation for Personal or Business Reasons

F13. I am now going to ask you (. . . . .) some questions about local travel for personal or business reasons, by which I mean trips of less than 80 kms or 50 miles.

In the past 12 months, did you (. . . . .) travel locally by CAR for personal or business reasons?

(1) Yes . . . . ◯ → **Go to F16**
(3) No . . . . ◯

(x) Don't know . . . . ◯
(r) Refusal . . . . ◯

F14. Were you (Was . . . . .) PREVENTED from travelling locally by car?

(1) Yes . . . . ◯
(3) No . . . . ◯ ▶ **Go to F25**

(x) Don't know . . . . ◯ ▶ **Go to F25**
(r) Refusal . . . . ◯ ▶ **Go to F25**

F15. What PREVENTED you (. . . . .) from travelling locally by car?

Interviewer: Read list. Mark all that apply.

	(1) Yes	(3) No	(x) DK	(r) Ref
(a) The lack of proper equipment on your (his/her) car (for example, hand or brake controls, power steering, etc.)	◯	◯	◯	◯
(b) You need (. . . . . needs) an attendant to help you (him/her)	◯	◯	◯	◯
(c) The lack of space for wheelchairs or other specialized equipment	◯	◯	◯	◯
(d) Other reason, specify	◯ ↓	◯	◯	◯

Interviewer: ***Go to F25.***

F16. In the past 12 months, did you (. . . . .) have DIFFICULTY travelling locally by car due to your (his/her) condition?

(1) Yes ◯
(3) No ◯
(x) Don't know ◯
(r) Refusal ◯
▶ **Go to F25** (No, Don't know, Refusal)

F17. Does this DIFFICULTY occur when you are (he/she is) the driver?

(1) Yes ◯
(3) No ◯
(x) Don't know ◯
(r) Refusal ◯
▶ **Go to F21** (No, Don't know, Refusal)

F18. Is this DIFFICULTY . . .

Interviewer: Read list. Mark all that apply.

	(1) Yes	(3) No	(x) DK	(r) Ref
(a) because you (he/she) lack(s) the proper equipment on your (his/her) car? (e.g., hand or brake controls, power steering)	◯	◯	◯	◯
(b) because you need (he/she needs) an attendant to help you (him/her)?	◯	◯	◯	◯
(c) due to the lack of space for wheelchairs or other specialized equipment?	◯	◯	◯	◯
(d) due to another reason? — specify	◯ ↓	◯	◯	◯

F19. How often was this a problem for you (. . . . .)?

Interviewer: Read list. Mark one only.

(1) Daily ◯
(2) Weekly ◯
(3) Monthly or less often ◯
(4) Never ◯ → **Go to F21**
(x) Don't know ◯
(r) Refusal ◯

F20. When this problem occurred, was it a big problem or a little problem?

Interviewer: Read list. Mark one only.

(1) Big problem . . . . ◯
(2) Little problem . . . ◯
(x) Don't know . . . . ◯
(r) Refusal . . . . ◯

F21. Does this DIFFICULTY occur when you are (. . . . . is) a passenger?

(1) Yes . . . . ◯
(3) No . . . . ◯ **Go to F25**
(x) Don't know . . . . ◯ **Go to F25**
(r) Refusal . . . . ◯ **Go to F25**

F22. Is this DIFFICULTY . . .

Interviewer: Read list. Mark all that apply.

	(1) Yes	(3) No	(x) DK	(r) Ref
(a) because you need (. . . . . needs) an attendant to help you (him/her)?	◯	◯	◯	◯
(b) because there is no space for wheelchairs or other specialized equipment?	◯	◯	◯	◯
(c) due to another reason? — specify	◯	◯	◯	◯

[ ]

F23. How often was this a problem for you (. . . . .)?

Interviewer: Read list. Mark one only.

(1) Daily . . . . ◯
(2) Weekly . . . . ◯
(3) Monthly or less often . . . . ◯
(4) Never . . . . ◯ → **Go to F25**
(x) Don't know . . . . ◯
(r) Refusal . . . . ◯

F24. When this problem occurred, was it a big problem or a little problem?

Interviewer: Read list. Mark one only.

(1) Big problem . . . . ◯
(2) Little problem . . . ◯
(x) Don't know . . . . ◯
(r) Refusal . . . . ◯

F25. In the past 12 months, did you (. . . . .) travel locally by specialized bus services, or local public transportation, including buses, subways and taxis?

(1) Yes . . . . ◯ → **Go to F28**
(3) No . . . . ◯
(x) Don't know . . . . ◯ **Go to F32**
(r) Refusal . . . . ◯ **Go to F32**

F26. Were you (Was . . . . .) PREVENTED from travelling locally by specialized bus services, or local public transportation, including buses, subways and taxis?

(1) Yes . . . . ◯
(3) No . . . . ◯
(x) Don't know . . . . ◯
(r) Refusal . . . . ◯

(3), (x), (r): **Go to F32**

F27. What PREVENTS you (. . . . .) from travelling locally by specialized bus services, or local public transportation (including buses, subways and taxis)?

Interviewer: Read list.

	(1) Yes	(3) No	(x) DK	(r) Ref
(a) service not available on a 24 hour, 7 day a week basis	◯	◯	◯	◯
(b) booking rules don't allow for last minute arrangements	◯	◯	◯	◯
(c) getting to or locating bus stops	◯	◯	◯	◯
(d) getting on or off vehicles	◯	◯	◯	◯
(e) seeing signs or notices	◯	◯	◯	◯
(f) other, specify	◯	◯	◯	◯

Interviewer: ***Go to F32.***

F28. In the past 12 months, did you (. . . . .) have any DIFFICULTY travelling locally by specialized bus or van services, or local public transportation, because of your (his/her) condition?

(1) Yes . . . . ◯
(3) No . . . . ◯
(x) Don't know . . . . ◯
(r) Refusal . . . . ◯

(3), (x), (r): **Go to F32**

F29. What type of difficulty did you (. . . . .) have?

Interviewer: Read list. Mark all that apply.

	(1) Yes	(3) No	(x) DK	(r) Ref
(a) service not available on a 24 hour, 7 day a week basis	◯	◯	◯	◯
(b) booking rules don't allow for last minute arrangements	◯	◯	◯	◯
(c) getting to or locating bus stops	◯	◯	◯	◯
(d) getting on or off vehicles	◯	◯	◯	◯
(e) seeing signs or notices	◯	◯	◯	◯
(f) other, specify	◯	◯	◯	◯

F30. How often was this a problem for you (. . . . .)?

Interviewer: Read list. Mark one only.

(1) Daily . . . . ◯
(2) Weekly . . . . ◯
(3) Monthly or less often . . . . ◯
(4) Never . . . . ◯ → **Go to F32**
(x) Don't know . . . . ◯
(r) Refusal . . . . ◯

F31. When this problem occurred, was it a big problem or a little problem?

Interviewer: Read list. Mark one only.

(1) Big problem . . . . ◯
(2) Little problem . . . ◯
(x) Don't know . . . . ◯
(r) Refusal . . . . ◯

Long Distance Travel for Personal or Business Reasons

F32. I am now going to ask you (. . . . .) some questions about long distance travel for personal or business reasons, by which I mean trips of 80 kms or 50 miles or more.

In the past 12 months, did you (. . . . .) take any long distance trips for personal or business reasons?

(1) Yes . . . . ◯ → **Go to F35**
(3) No . . . . ◯
(x) Don't know . . . . ◯
(r) Refusal . . . . ◯

F33. Are you (Is . . . . .) PREVENTED from travelling long distance?

(1) Yes . . . . ◯
(3) No . . . . ◯ → **Go to F41**
(x) Don't know . . . . ◯
(r) Refusal . . . . ◯

F34. What PREVENTS you (. . . . .) from travelling long distance?

Interviewer: Read list. Mark all that apply.

	(1) Yes	(3) No	(x) DK	(r) Ref
(a) Flight or ride aggravates your (his/her) condition	◯	◯	◯	◯
(b) Lack of appropriate transportation to and from terminal or station	◯	◯	◯	◯
(c) Moving around terminal or station	◯	◯	◯	◯
(d) Boarding or disembarking	◯	◯	◯	◯
(e) Seating on board	◯	◯	◯	◯
(f) Seeing signs or notices	◯	◯	◯	◯
(g) Hearing announcements	◯	◯	◯	◯
(h) Washroom facilities	◯	◯	◯	◯
(i) Unsupportive staff	◯	◯	◯	◯
(j) Transporting wheelchair or other specialized aids	◯	◯	◯	◯
(k) Too costly	◯	◯	◯	◯
(l) Other, specify	◯	◯	◯	◯

Interviewer: ***Go to F38.***

F35. In the past 12 months, did you (. . . . .) travel long distance for personal or business reasons by:

Interviewer: Read list. Mark all that apply.

	(1) Yes	(3) No	(x) DK	(r) Ref
(a) Car?	◯	◯	◯	◯
(b) Inter-city bus?	◯	◯	◯	◯
(c) Train?	◯	◯	◯	◯
(d) Airplane?	◯	◯	◯	◯

F35. edit

Interviewer: If F35(b) or F35(c) or F35(d) is Yes, then continue. Otherwise, go to F38.

F36. In the past 12 months, did you (. . . . .) have any DIFFICULTY travelling by bus, train or airplane?

(1) Yes ◯
(3) No ◯
(x) Don't know ◯
(r) Refusal ◯

Go to F38

F37. What kind of DIFFICULTY did you (. . . . .) have travelling by bus, train or airplane? I will read you a list. Please answer Yes or No to each.

Interviewer: Read list.

	(1) Yes	(3) No	(x) DK	(r) Ref
(a) Flight or ride aggravates your (his/her) condition	◯	◯	◯	◯
(b) Lack of appropriate transportation to and from terminal or station	◯	◯	◯	◯
(c) Moving around terminal or station	◯	◯	◯	◯
(d) Boarding or disembarking	◯	◯	◯	◯
(e) Seating on board	◯	◯	◯	◯
(f) Seeing signs or notices	◯	◯	◯	◯
(g) Hearing announcements	◯	◯	◯	◯
(h) Washroom facilities	◯	◯	◯	◯
(i) Unsupportive staff	◯	◯	◯	◯
(j) Transporting wheelchair or other specialized aids	◯	◯	◯	◯
(k) Too costly	◯	◯	◯	◯
(l) Other, specify	◯	◯	◯	◯

F38. In the past 12 months, was long distance travel a problem for you (. . . . .) because of your (his/her) condition?

(1) Yes ◯
(3) No ◯
(x) Don't know ◯
(r) Refusal ◯

Go to F41

F39. How often was this a problem for you (. . . . .)?

Interviewer: Read list. Mark one only.

(1) Daily . . . . ◯
(2) Weekly . . . . ◯
(3) Monthly or less often . . . . ◯
(4) Never . . . . ◯ → **Go to F41**

(x) Don't know . . . . ◯
(r) Refusal . . . . ◯

F40. When this problem occurred, was it a big problem or a little problem?

Interviewer: Read list. Mark one only.

(1) Big problem . . . . ◯
(2) Little problem . . . ◯

(x) Don't know . . . . ◯
(r) Refusal . . . . ◯

Interviewer: The last few questions in this section are asked about BOTH local transportation and long distance travel.

F41. In the past 12 months, did you (. . . . .) have any OUT-OF-POCKET or DIRECT EXPENSES for transportation, for example, travel to and from treatment, therapy or other medical or rehabilitation services; or extra expenses due to the need for more expensive transportation?

INCLUDE amounts not covered by insurance such as exclusions, deductibles and expenses over limits. EXCLUDE payments for which you have (he / she has) been or will be reimbursed by any insurance or government program.

(1) Yes . . . . ◯
(3) No . . . . ◯ ▶ **Go to F44**
(x) Don't know . . . . ◯ ▶ **Go to F44**
(r) Refusal . . . . ◯ ▶ **Go to F44**

F42. What is your (. . . . . 's) best estimate of the DIRECT cost to you (. . . . .), for these extra expenses?

(INCLUDE amounts not covered by insurance such as exclusions, deductibles and expenses over limits. EXCLUDE payments for which you have (he / she has) been or will be reimbursed by any insurance or government program.)

$ ☐☐☐☐☐☐ .00 → **Go to F44**

(x) Don't know . . . . ◯
(r) Refusal . . . . ◯

F43. Which one of the following groups is the best estimate of the DIRECT costs to you (. . . . .), for these expenses?

Interviewer: Read list. Mark one only.

(1) less than $100 . . . . ◯
(2) $100 to less than $200 . . . . ◯
(3) $200 to less than $500 . . . . ◯
(4) $500 to less than $1,000 . . . . . . ◯
(5) $1,000 to less than $2,000 . . . . ◯
(6) $2,000 to less than $5,000 . . . . ◯
(7) $5,000 or more . . . . ◯

(x) Don't know . . . . ◯
(r) Refusal . . . . ◯

Housing

F44. I am now going to ask you (. . . . .) some questions about your (his/her) residence and any specialized features you (he/she) may have.

Because of your (. . . . . 's) condition, do you (does . . . . .) use any specialized features to enter or leave your (his/her) residence, or inside your (his/her) residence?

(1) Yes . . . . ◯
(3) No . . . . ◯
(x) Don't know . . . . ◯
(r) Refusal . . . . ◯

(3), (x), (r): **Go to F47**

F45. Do you (Does . . . . .) now use:

Interviewer: Read list. Mark all that apply.

	(1) Yes	(3) No	(x) DK	(r) Ref
(a) ramps or street level entrances?	◯	◯	◯	◯
(b) automatic or easy to open doors (includes lever handles)?	◯	◯	◯	◯
(c) widened doorways or hallways?	◯	◯	◯	◯
(d) elevator or lift device?	◯	◯	◯	◯
(e) visual alarms or audio warning devices?	◯	◯	◯	◯
(f) grab bars or a bath lift (in the bathroom)?	◯	◯	◯	◯
(g) lowered counters in the kitchen?	◯	◯	◯	◯
(h) other, specify	◯	◯	◯	◯

[ ]

F46. Do you (Does . . . . .) need any other specialized features, which you do (he/she does) not already have?

(1) Yes . . . . ◯ → **Go to F48**
(3) No . . . . ◯
(x) Don't know . . . . ◯
(r) Refusal . . . . ◯

(3), (x), (r): **Go to F50**

F47. Are there any specialized features that you NEED (. . . . . NEEDS) but do not have (does not have)?

(1) Yes . . . . ◯
(3) No . . . . ◯
(x) Don't know . . . . ◯
(r) Refusal . . . . ◯

(3), (x), (r): **Go to F50**

F48. Which specialized features do you (does .....) need, but do(es) not have?

Interviewer: Read list. Mark all that apply.

	(1) Yes	(3) No	(x) DK	(r) Ref
(a) Ramps or street level entrances	◯	◯	◯	◯
(b) Automatic or easy to open doors (includes lever handles)	◯	◯	◯	◯
(c) Widened doorways or hallways	◯	◯	◯	◯
(d) Elevator or lift device	◯	◯	◯	◯
(e) Visual alarms or audio warning devices	◯	◯	◯	◯
(f) Grab bars or a bath lift (in the bathroom)	◯	◯	◯	◯
(g) Lowered counters in the kitchen	◯	◯	◯	◯
(h) Other, specify	◯	◯	◯	◯

F49. Why don't you (doesn't .....) have this (these) feature(s)?

Interviewer: Read list. Mark all that apply.

	(1) Yes	(3) No	(x) DK	(r) Ref
(a) Not covered by insurance	◯	◯	◯	◯
(b) Too expensive	◯	◯	◯	◯
(c) Specialized features not approved or recommended by health professional	◯	◯	◯	◯
(d) Currently on a waiting list for aids (features)	◯	◯	◯	◯
(e) Other reason, specify	◯	◯	◯	◯

F50. Has the design and layout of your (..... 's) home, including entrance and exits, made it difficult to participate in the activities you (he/she) want(s) or need(s) to do? (**INCLUDE ALL** activities of daily living, not just leisure or recreational activities.)

(1) Yes .... ◯
(3) No .... ◯
(x) Don't know .... ◯
(r) Refusal .... ◯

Go to F53

F51. In the past 12 months, how often has the design and layout of your (..... 's) home, including entrance and exits, made it difficult to participate in the activities you (he/she) want(s) or need(s) to do? (**INCLUDE ALL** activities of daily living, not just leisure or recreational activities.)

Interviewer: Read list. Mark one only.

(1) Daily .... ◯
(2) Weekly .... ◯
(3) Monthly or less often .... ◯
(x) Don't know .... ◯
(r) Refusal .... ◯

F52. When this problem occurred, was it a big problem or a little problem?

Interviewer: Read list. Mark one only.

(1) Big problem . . . . ◯
(2) Little problem . . . ◯
(x) Don't know . . . . ◯
(r) Refusal . . . . ◯

F53. In the past 12 months, did you (. . . . .) or your (his/her) family living with you (him/her), have any OUT-OF-POCKET or DIRECT EXPENSES for modifications to your (his/her) residence because of your (his/her) condition?

INCLUDE amounts not covered by insurance such as exclusions, deductibles and expenses over limits. EXCLUDE payments for which you have (he/she has) been or will be reimbursed by any insurance or government program.

(1) Yes . . . . ◯
(3) No . . . . ◯
(x) Don't know . . . . ◯
(r) Refusal . . . . ◯

(3), (x), (r): **Go to Section G**

F54. What is your (. . . . . 's) best estimate of the DIRECT costs to you (him/her), or your (his/her) family living with you (him/her), for these extra expenses?

(INCLUDE amounts not covered by insurance such as exclusions, deductibles and expenses over limits. EXCLUDE payments for which you have (he/she has) been or will be reimbursed by any insurance or government program.)

$ ☐☐☐☐☐☐ .00 → **Go to Section G**

(x) Don't know . . . . ◯
(r) Refusal . . . . ◯

(x), (r): **Go to F55**

F55. Which one of the following groups is your best estimate of the DIRECT costs to you (. . . . .), or your (his/her) family living with you (him/her), for these expenses? I will read you a list.

Interviewer: Read list. Mark one only.

(1) less than $100 . . . . ◯
(2) $100 to less than $200 . . . . ◯
(3) $200 to less than $500 . . . . ◯
(4) $500 to less than $1,000 . . . . ◯
(5) $1,000 to less than $2,000 . . . . . ◯
(6) $2,000 to less than $5,000 . . . . . ◯
(7) $5,000 or more . . . . ◯
(x) Don't know . . . . ◯
(r) Refusal . . . . ◯

SECTION G — ECONOMIC CHARACTERISTICS

G1. The next question is about insurance coverage. Please include any private, government or employer-paid plans.

Do you (Does . . . . .) have insurance that covers all or part of:

Interviewer: Read list.

	(1) Yes	(3) No	(x) DK	(r) Ref
(a) the cost of your (his/her) prescription medication?	◯	◯	◯	◯
(b) the cost of eye glasses or contact lenses?	◯	◯	◯	◯
(c) hospital charges for a private or semi-private room?	◯	◯	◯	◯

G2. Did you (. . . . .) claim a Medical Expense Tax Credit with your (his/her) 2000 income tax return?

(1) Yes ○
(3) No ○ ▶ **Go to G4**
(x) Don't know ○ ▶ **Go to G4**
(r) Refusal ○ ▶ **Go to G4**

G3. Did you (. . . . .) receive it?

(1) Yes ○
(3) No ○
(x) Don't know ○
(r) Refusal ○

G4. Did you (. . . . .), or someone else on your (his/her) behalf, claim the Disability Tax Credit on an income tax return for the year 2000?

(1) Yes ○
(3) No ○ → **Go to G6**
(x) Don't know ○ ▶ **Go to G7**
(r) Refusal ○ ▶ **Go to G7**

G5. Did you (. . . . .) receive it?

(1) Yes ○ → **Go to G7**
(3) No ○
(x) Don't know ○
(r) Refusal ○

G6. Why did you (. . . . .) not claim the Disability Tax Credit? I will read you a list. Please answer yes or no to each.

Interviewer: Read list.

	(1) Yes		(3) No	(x) DK	(r) Ref
(a) You (. . . . .) did not know it existed	○	→ **Go to G7**	○	○	○
(b) You (. . . . .) did not think that you (he/she) would meet the eligibility requirements	○		○	○	○
(c) You were (. . . . . was) not able to obtain the disability certificate (Form T2201) from your (his/her) doctor	○		○	○	○
(d) Other reason, specify	○ ↓		○	○	○

[ ]

G7. Did you (. . . . .), or someone else on your (his/her) behalf, claim the following on an income tax return for the year 2000? I will read you a list. Please answer yes or no to each.

Interviewer: Read list.

	(1) Yes	(3) No	(x) DK	(r) Ref
(a) Attendant care expenses	○	○	○	○
(b) Caregiver amount	○	○	○	○
(c) Tuition and education amounts	○	○	○	○
(d) Child care expenses	○	○	○	○
(e) Other, specify	○ ↓	○	○	○

[ ]

G8. **The next few questions are about income. Although many health expenses are covered by health insurance, there is still a relationship between health and income. Please be assured that, like all the other information which you have provided, these answers will be kept strictly confidential.**

In the year 2000, did you (. . . . .) personally receive income from the following sources? I will read you a list. Please answer yes or no to each source.

Interviewer: Read list.

	(1) Yes	(3) No	(x) DK	(r) Ref
(a) Wages and salaries	◯	◯	◯	◯
(b) Income from self-employment	◯	◯	◯	◯
(c) Employment Insurance	◯	◯	◯	◯
(d) Worker's Compensation	◯	◯	◯	◯
(e) Benefits from Canada or Quebec Pension Plan	◯	◯	◯	◯
(f) Old Age Security Pension	◯	◯	◯	◯
(g) Guaranteed Income Supplement or Spouse's Allowance	◯	◯	◯	◯
(h) Disability pension from the Canada or Quebec Pension Plan	◯	◯	◯	◯
(i) Insurance plans, such as private or employer disability insurance plan or motor vehicle accident insurance	◯	◯	◯	◯
(j) Child Tax Benefit	◯	◯	◯	◯
(k) Provincial or municipal social assistance or welfare	◯	◯	◯	◯
(l) Other income, such as retirement pensions, dividends and interest on bonds, deposits and savings; alimony, child support, scholarships, etc.	◯	◯	◯	◯
(m) Other (for example, Veteran's Disability Pension or Allowance, federal or provincial assistance not mentioned above, etc.)	◯ ↓	◯	◯	◯

[ ]

G9. **For the year ending December 31, 2000, what is your (. . . . . 's) best estimate of your (his/her) total PERSONAL income, before taxes and deductions, from all sources?**

$ [| | | | |] .00

(a) No income or loss . . . . ◯

▶ **Go to G11**

(x) Don't know . . . . ◯

(r) Refusal . . . . ◯

▶ **Go to G10**

G10. **Can you (. . . . .) estimate in which of the following groups your (his/her) personal income fell? I will read you a list.**

Interviewer: Read list. Mark one only.

(1) $1 to less than $5,000 . . . . ◯

(2) $5,000 to less than $10,000 . . . . . . ◯

(3) $10,000 to less than $15,000 . . . . . ◯

(4) $15,000 to less than $20,000 . . . . . ◯

(5) $20,000 to less than $30,000 . . . . . ◯

(6) $30,000 to less than $40,000 . . . . . ◯

(7) $40,000 to less than $50,000 . . . . . ◯

(8) $50,000 to less than $60,000 . . . . . ◯

(9) $60,000 to less than $80,000 . . . . . ◯

(10) $80,000 or more . . . . ◯

(x) Don't know . . . . ◯

(r) Refusal . . . . ◯

G11. Is this a one person household?

(1) Yes .... ○ → **Go to Follow-up question**
(3) No .... ○

(x) Don't know .... ○
(r) Refusal .... ○
▶ **Go to Follow-up question**

G12. For the year ending December 31, 2000, what is your (..... 's) best estimate of the total income, before taxes and deductions, of all household members, including yourself (himself/herself), from all sources?

$ |_|_|_|_|_|_| .00

(a) No income or loss ..... ○
▶ **Go to Follow-up question**

(x) Don't know .... ○
(r) Refusal .... ○
▶ **Go to G13**

G13. Can you (.....) estimate in which of the following groups your (his/her) household income fell? I will read you a list.

Interviewer: Read list. Mark one only.

(1) $1 to less than $5,000 .... ○
(2) $5,000 to less than $10,000 ...... ○
(3) $10,000 to less than $15,000 ..... ○
(4) $15,000 to less than $20,000 ..... ○
(5) $20,000 to less than $30,000 ..... ○
(6) $30,000 to less than $40,000 ..... ○
(7) $40,000 to less than $50,000 ..... ○
(8) $50,000 to less than $60,000 ..... ○
(9) $60,000 to less than $80,000 ..... ○
(10) $80,000 or more .... ○

(x) Don't know .... ○
(r) Refusal .... ○

Interviewer: Go to Follow-up question.

FOLLOW-UP

That's the end of our questions. Someone from Statistics Canada may contact you (. . . . .) in a year or two to find out more about your (his/her) day-to-day activities. In case there are difficulties reaching you (them), could you please give me the name, address and telephone number of a family member or friend we could contact? We will only call this person if you move (. . . . . moves) and then only to obtain the new address or telephone number.

(1) Yes . . . . ◯ → **Go to Name and Address of Other Contact**
(3) No . . . . ◯
(r) Refusal . . . . . ◯ ▶ **Go to End of Interview**

Name and Address of Other Contact:

First name(s) ______ Initial(s) ☐☐ Family name ______

Address:
Number and Street or lot and concession or exact location ______ Apt. No. ☐☐☐☐

City, Town, Village or Municipality ______ Province or Territory ______ Postal Code ☐☐☐ ☐☐☐

Telephone:
Area code ☐☐☐ Telephone No. ☐☐☐ — ☐☐☐☐

END OF INTERVIEW
THANK RESPONDENT

COMMENTS

Question No.	

RECORD OF CALLS AND VISITS

Visit/call number	Date	Time	Observations

RECORD OF INTERVIEWS

Interview number	Date	Month	Began	Ended
1			:	:
2			:	:
3			:	:
4			:	:

FIELD STATUS CODE

FINAL STATUS CODE

00 Complete
01 Partial
20 Duplicate (already interviewed)
28 Death
50 Refusal
52 Unable to respond – Proxy not available
53 Does not speak English or French – Proxy not available
54 Impossible to contact/recontact
55 Impossible to trace
56 Absent for duration of survey – Proxy not available
57 No longer living in Canada

Interviewer's Name (Please print)	Interviewer's Telephone Number	Interviewer's Identification Number

PROFILE SHEET

Case Identification Number: ______________________

ACTIVITY LIMITATIONS

SECTION	ACTIVITY/CONDITION	LIMITATION	USE aid	NEED aid
A	General	☐		
B	Hearing	☐	☐	☐
	Seeing	☐	☐	☐
	Communicating	☐	☐	☐
	Mobility	☐	☐	☐
	Agility	☐	☐	☐
	Pain	☐		
	Learning	☐	☐	☐
	Confusion / Memory	☐		
	Developmental	☐		
	Emotional / Psychological	☐		
	Other		☐	☐

HELP WITH EVERYDAY ACTIVITIES

SECTION	ACTIVITY	RECEIVE help	NEED help
C	Meals	☐	☐
	Housework	☐	☐
	Chores	☐	☐
	Appointments	☐	☐
	Finances	☐	☐
	Childcare	☐	☐
	Personal Care	☐	☐
	Nursing / Treatment	☐	☐
	Moving About	☐	☐

Appendix C

Dictionary of Data File

TABLE OF CONTENTS

CONTROL VARIABLES 1

DEMOGRAPHIC VARIABLES 2

ACTIVITY LIMITATIONS 3

HELP WITH DAILY ACTIVITY 48

EDUCATION 122

EMPLOYMENT 154

EMPLOYMENT-EMPLOYED 155

EMPLOYMENT-UNEMPLOYED 191

EMPLOYMENT-NOT IN LABOUR FORCE 212

SOCIAL PARTICIPATION 223

ECONOMIC CHARACTERISTICS 288

DERIVED/CENSUS VARIABLES 302

Section: **CONTROL VARIABLES**

Variable Name:	**IDNUM**	*Position:*	1	*Length:*	8
Collection Name:	IDNUM				

Respondent's CaseID on label from sample file

Variable Name:	**DISAB**	*Position:*	9	*Length:*	1
Collection Name:	DISAB				

Is the respondent disabled or not?

		FREQ	WTD
0	Non-Disabled	0	0
1	Disabled	20,710	3,420,338
		======	=========
		20,710	3,420,338

Coverage: All respondents
Source: Participation and Activity Limitation Survey, 2001
Note: This variable contains the flag to indicate whether or not the respondent is disabled. Users are advised to use this variable whenever they are tabulating characteristics from the PALS questionnaire.

Variable Name:	**WEIGHT_P**	*Position:*	10	*Length:*	8
Collection Name:	WEIGHT_P				

Record weight

Mean	307.44
Minimum	16.92
Maximum	4739.92
Range	4722.99

Note: As the PALS is a survey based on a probability sampling plan, each person selected for the survey represents himself/herself as well as a certain number of other persons in the target population who are not part of the sample. The variable WEIGHT_P, called weight, gives the number of persons represented by each record. The weights of the individuals have been calculated based on the probability of selection and have been adjusted so that the PALS sample is representative of the population of interest. Because of those adjustments and because certain individuals had unequal probabilities of selection, the weights might vary significantly from one person to another. The weight must therefore be used for all estimates and analyses that are based on the PUMF otherwise the results will be biased.

Section: **DEMOGRAPHIC VARIABLES**

Variable Name: **SEX** *Position:* 18 *Length:* 1
Collection Name: SEX

Respondent's Sex on label from sample file

		FREQ	WTD
1	Male	9,270	1,526,827
2	Female	11,440	1,893,511
		======	=========
		20,710	3,420,338

Variable Name: **AGEGRP5** *Position:* 19 *Length:* 2
Collection Name: AGEGRP5

Derived variable: Five year age group

		FREQ	WTD
01	15-19	1,485	69,151
02	20-24	1,560	81,878
03	25-29	695	84,047
04	30-34	956	116,187
05	35-39	1,495	180,933
06	40-44	1,923	245,445
07	45-49	1,096	267,798
08	50-54	1,263	312,247
09	55-59	1,270	312,875
10	60-64	1,171	297,931
11	65-69	1,636	302,407
12	70-74	1,931	346,769
13	75-79	1,778	351,828
14	80-84	1,371	248,695
15	85+	1,080	202,145
		======	=========
		20,710	3,420,336

Coverage: All respondents
Note: AGEGRP5 is derived from the PALS age variable for persons with disabilities. For persons without disabilities, AGEGRP5 is derived from the AGE variable on the 2001 Census of Population.

Section: **ACTIVITY LIMITATIONS**

Variable Name: **B1** *Position:* 21 *Length:* 2
Collection Name: AB1

I am going to ask you a series of questions about your (.....'s) ability to do certain activities. Please tell me only about those difficulties that have lasted, or are expected to last, six months or more. Do you (Does.....) use a hearing aid or hearing aids?

		FREQ	WTD
1	Yes	2,316	398,056
3	No	18,227	2,983,226
93	Not applicable	0	0
98	Not stated	4	2,211
R	Refusal	0	0
X	Don't know	163	36,845
		======	=========
		20,710	3,420,338

Coverage: All respondents
Source: Participation and Activity Limitation Survey, 2001

Variable Name: **B9** *Position:* 23 *Length:* 2
Collection Name: AB9

Besides hearing aids, do you (does....) Use any other aids, specialized equipment or services for persons who are deaf or hard of hearing, for example, a volume control telephone or TV decoder?

		FREQ	WTD
1	Yes	1,240	201,636
3	No	4,439	799,404
93	Not applicable	14,490	2,330,160
98	Not stated	535	88,333
R	Refusal	0	0
X	Don't know	6	805
		======	=========
		20,710	3,420,338

Coverage: Respondents who reported a "hearing" limitation (HEARLIM=1)
Source: Participation and Activity Limitation Survey, 2001

Variable Name: **B10A** *Position:* 25 *Length:* 2
Collection Name: AB10A

Do you (Does.....) now USE: a computer to communicate, e.g., e-mail or chat service?

		FREQ	WTD
1	Yes	235	35,987
3	No	967	160,094
93	Not applicable	18,935	3,130,369
98	Not stated	573	93,888
R	Refusal	0	0
X	Don't know	0	0
		======	=========
		20,710	3,420,338

Coverage: Respondents who answered B9=1
Source: Participation and Activity Limitation Survey, 2001

Variable Name: **B10B** *Position:* 27 *Length:* 2
Collection Name: AB10B

Do you (Does.....) now USE: a volume control telephone?

		FREQ	WTD
1	Yes	1,015	164,358
3	No	199	33,469
93	Not applicable	18,935	3,130,369
98	Not stated	561	92,143
R	Refusal	0	0
X	Don't know	0	0
		======	=========
		20,710	3,420,339

Coverage: Respondents who answered B9=1
Source: Participation and Activity Limitation Survey, 2001

Variable Name: **B10C** *Position:* 29 *Length:* 2
Collection Name: AB10C

Do you (Does.....) now USE: a TTY or TDD?

		FREQ	WTD
1	Yes	99	13,997
3	No	1,076	177,242
93	Not applicable	18,935	3,130,369
98	Not stated	582	95,744
R	Refusal	0	0
X	Don't know	18	2,986
		======	=========
		20,710	3,420,338

Coverage: Respondents who answered B9=1
Source: Participation and Activity Limitation Survey, 2001

Variable Name: **B10D** *Position:* 31 *Length:* 2
Collection Name: AB10D

Do you (Does.....) now USE: a message relay service?

		FREQ	WTD
1	Yes	104	15,511
3	No	1,081	178,246
93	Not applicable	18,935	3,130,369
98	Not stated	583	95,205
R	Refusal	0	0
X	Don't know	7	1,008
		======	=========
		20,710	3,420,339

Coverage: Respondents who answered B9=1
Source: Participation and Activity Limitation Survey, 2001

Variable Name: **B10E** *Position:* 33 *Length:* 2
Collection Name: AB10E

Do you (Does.....) now USE: other phone related devices, e.g., flashers?

		FREQ	WTD
1	Yes	201	38,260
3	No	993	156,835
93	Not applicable	18,935	3,130,369
98	Not stated	578	94,461
R	Refusal	0	0
X	Don't know	3	413
		======	=========
		20,710	3,420,338

Coverage: Respondents who answered B9=1
Source: Participation and Activity Limitation Survey, 2001

Variable Name: **B10F** *Position:* 35 *Length:* 2
Collection Name: AB10F

Do you (Does.....) now USE: a closed caption T.V. or decoder?

		FREQ	WTD
1	Yes	305	46,148
3	No	892	148,702
93	Not applicable	18,935	3,130,369
98	Not stated	575	94,398
R	Refusal	0	0
X	Don't know	3	721
		======	=========
		20,710	3,420,338

Coverage: Respondents who answered B9=1
Source: Participation and Activity Limitation Survey, 2001

Variable Name: **B10G** *Position:* 37 *Length:* 2
Collection Name: AB10G

Do you (Does.....) now USE: amplifiers, e.g., FM, acoustic, infrared?

		FREQ	WTD
1	Yes	169	29,247
3	No	1,025	166,735
93	Not applicable	18,935	3,130,369
98	Not stated	577	93,396
R	Refusal	0	0
X	Don't know	4	591
		======	=========
		20,710	3,420,338

Coverage: Respondents who answered B9=1
Source: Participation and Activity Limitation Survey, 2001

Variable Name: **B10H** *Position:* 39 *Length:* 2
Collection Name: AB10H

Do you (Does.....) now USE: visual or vibrating alarms?

		FREQ	WTD
1	Yes	131	16,991
3	No	1,058	176,625
93	Not applicable	18,935	3,130,369
98	Not stated	579	95,709
R	Refusal	0	0
X	Don't know	7	645
		======	=========
		20,710	3,420,339

Coverage: Respondents who answered B9=1
Source: Participation and Activity Limitation Survey, 2001

Variable Name: **B10I** *Position:* 41 *Length:* 2
Collection Name: AB10I

Do you (Does.....) now USE: a Sign language interpreter?

		FREQ	WTD
1	Yes	49	5,957
3	No	1,145	189,113
93	Not applicable	18,935	3,130,369
98	Not stated	578	94,559
R	Refusal	0	0
X	Don't know	3	339
		======	=========
		20,710	3,420,337

Coverage: Respondents who answered B9=1
Source: Participation and Activity Limitation Survey, 2001

Variable Name:	**B10_OTH**	*Position:*	43	*Length:*	2
Collection Name:	AB10_OTH				

Derived variable: Using hearing aid dog/other aid?

		FREQ	WTD
1	Yes	88	13,782
3	No	1,100	180,101
93	Not applicable	19,470	3,218,702
98	Not stated	49	7,476
R	Refusal	0	0
X	Don't know	3	277
		======	=========
		20,710	3,420,338

Coverage: Respondents who answered B9=1
Source: Participation and Activity Limitation Survey, 2001
Note: B10_OTH is derived from variables B10J and B10K.

Variable Name:	**B11**	*Position:*	45	*Length:*	2
Collection Name:	AB11				

Are there any aids, specialized equipment or services for persons who are deaf or hard of hearing that you think you NEED (..... thinks he / she NEEDS) but do (does) not have?

		FREQ	WTD
1	Yes	1,149	199,604
3	No	4,273	759,204
93	Not applicable	14,490	2,330,160
98	Not stated	540	85,635
R	Refusal	0	0
X	Don't know	258	45,735
		======	=========
		20,710	3,420,338

Coverage: Respondents who reported a "hearing" limitation (HEARLIM=1)
Source: Participation and Activity Limitation Survey, 2001

Variable Name:	**B12**	*Position:*	47	*Length:*	2
Collection Name:	AB12				

Which aids do you (does.....) NEED but do (does) not have?

		FREQ	WTD
0	Valid data	1,084	185,339
93	Not applicable	19,021	3,135,099
98	Not stated	567	89,908
R	Refusal	1	42
X	Don't know	37	9,950
		======	=========
		20,710	3,420,338

Coverage: Respondents who answered B11=1
Source: Participation and Activity Limitation Survey, 2001

Variable Name:	**B12A**	*Position:*	49	*Length:*	2
Collection Name:	AB12A				

Which aids do you (does.....) NEED but do (does) not have: Computer to communicate (e.g., e-mail or chat service)?

		FREQ	WTD
0	Not selected	20,685	3,416,592
1	Yes	25	3,746
		======	=========
		20,710	3,420,338

Coverage: Respondents who answered B11=1
Source: Participation and Activity Limitation Survey, 2001

Variable Name:	**B12B**	*Position:*	51	*Length:*	2
Collection Name:	AB12B				

Which aids do you (does.....) NEED but do (does) not have: Volume control telephone?

		FREQ	WTD
0	Not selected	20,499	3,385,213
1	Yes	211	35,125
		======	=========
		20,710	3,420,338

Coverage: Respondents who answered B11=1
Source: Participation and Activity Limitation Survey, 2001

Variable Name: **B12C** *Position:* 53 *Length:* 2
Collection Name: AB12C

Which aids do you (does.....) NEED but do (does) not have, TTY or TDD?

		FREQ	WTD
0	Not selected	20,671	3,415,188
1	Yes	39	5,149
		======	=========
		20,710	3,420,337

Coverage: Respondents who answered B11=1
Source: Participation and Activity Limitation Survey, 2001

Variable Name: **B12D** *Position:* 55 *Length:* 2
Collection Name: AB12D

Which aids do you (does.....) NEED but do (does) not have: Message relay service?

		FREQ	WTD
0	Not selected	20,693	3,417,449
1	Yes	17	2,888
		======	=========
		20,710	3,420,337

Coverage: Respondents who answered B11=1
Source: Participation and Activity Limitation Survey, 2001

Variable Name: **B12E** *Position:* 57 *Length:* 2
Collection Name: AB12E

Which aids do you (does.....) NEED but do (does) not have: Other phone related devices (e.g., flashers)?

		FREQ	WTD
0	Not selected	20,651	3,411,333
1	Yes	59	9,004
		======	=========
		20,710	3,420,337

Coverage: Respondents who answered B11=1
Source: Participation and Activity Limitation Survey, 2001

Variable Name:	**B12F**	*Position:*	59	*Length:*	2
Collection Name:	AB12F				

Which aids do you (does.....) NEED but do (does) not have: closed caption T.V. or decoder?

		FREQ	WTD
0	Not selected	20,642	3,406,729
1	Yes	68	13,609
		======	=========
		20,710	3,420,338

Coverage: Respondents who answered B11=1
Source: Participation and Activity Limitation Survey, 2001

Variable Name:	**B12G**	*Position:*	61	*Length:*	2
Collection Name:	AB12G				

Which aids do you (does.....) NEED but do (does) not have: Amplifiers (e.g., FM, acoustic, infrared)?

		FREQ	WTD
0	Not selected	20,666	3,412,073
1	Yes	44	8,265
		======	=========
		20,710	3,420,338

Coverage: Respondents who answered B11=1
Source: Participation and Activity Limitation Survey, 2001

Variable Name:	**B12H**	*Position:*	63	*Length:*	2
Collection Name:	AB12H				

Which aids do you (does.....) NEED but do (does) not have: visual or vibrating alarms?

		FREQ	WTD
0	Not selected	20,666	3,412,348
1	Yes	44	7,990
		======	=========
		20,710	3,420,338

Coverage: Respondents who answered B11=1
Source: Participation and Activity Limitation Survey, 2001

Variable Name:	**B12I**	*Position:*	65	*Length:*	2
Collection Name:	AB12I				

Which aids do you (does.....) NEED but do (does) not have: sign language interpreter?

		FREQ	WTD
0	Not selected	20,704	3,419,266
1	Yes	6	1,072
		======	=========
		20,710	3,420,338

Coverage: Respondents who answered B11=1
Source: Participation and Activity Limitation Survey, 2001

Variable Name:	**B12_OTH**	*Position:*	67	*Length:*	2
Collection Name:	AB12_OTH				

Derived variable: Need, but do not have hearing aid dog/other aid?

		FREQ	WTD
0	Not selected	20,567	3,393,832
1	Yes	143	26,506
		======	=========
		20,710	3,420,338

Coverage: Respondents who answered B11=1
Source: Participation and Activity Limitation Survey, 2001
Note: B12_OTH is derived from variables B12J and B12K.

Variable Name:	**B12L**	*Position:*	69	*Length:*	2
Collection Name:	AB12L				

Derived variable: Need but do not have: Hearing aid(s)?

		FREQ	WTD
0	Not selected	20,017	3,299,889
1	Yes	693	120,449
		======	=========
		20,710	3,420,338

Coverage: Respondents who answered B11=1 and B12K=1
Source: Participation and Activity Limitation Survey, 2001
Note: B12L is derived from write-in resposes in B12K.

Variable Name: **B14**	*Position:* 71	*Length:* 2	
Collection Name: AB14			

Do you (Does.....) wear glasses or contact lenses to see up close?

		FREQ	WTD
1	Yes	14,036	2,588,598
3	No	6,599	822,827
93	Not applicable	0	0
98	Not stated	10	873
R	Refusal	0	0
X	Don't know	65	8,039
		======	=========
		20,710	3,420,337

Coverage: All respondents
Source: Participation and Activity Limitation Survey, 2001

Variable Name: **B19**	*Position:* 73	*Length:* 2	
Collection Name: AB19			

Do you (Does.....) wear glasses or contact lenses to see at a distance?

		FREQ	WTD
1	Yes	11,960	2,023,333
3	No	8,612	1,371,973
93	Not applicable	0	0
98	Not stated	24	5,350
R	Refusal	0	0
X	Don't know	114	19,682
		======	=========
		20,710	3,420,338

Coverage: All respondents
Source: Participation and Activity Limitation Survey, 2001

Variable Name:	**B25**	*Position:*	75	*Length:*	2
Collection Name:	AB25				

Besides glasses or contact lenses, do you (does.....) USE any other aids or specialized equipment for persons who are blind or visually impaired, for example, magnifiers or Braille reading materials?

		FREQ	WTD
1	Yes	1,050	175,802
3	No	2,199	362,582
93	Not applicable	16,958	2,792,665
98	Not stated	503	89,288
R	Refusal	0	0
X	Don't know	0	0
		======	=========
		20,710	3,420,337

Coverage: Respondents who reported a "seeing" limitation (SEELIM=1)
Source: Participation and Activity Limitation Survey, 2001

Variable Name:	**B26A**	*Position:*	77	*Length:*	2
Collection Name:	AB26A				

Do you (Does.....) now USE magnifiers?

		FREQ	WTD
1	Yes	922	159,778
3	No	117	14,251
93	Not applicable	19,157	3,155,247
98	Not stated	514	91,061
R	Refusal	0	0
X	Don't know	0	0
		======	=========
		20,710	3,420,337

Coverage: Respondents who answered B25=1
Source: Participation and Activity Limitation Survey, 2001

Variable Name: **B26B** *Position:* 79 *Length:* 2
Collection Name: AB26B

Do you (Does.....) now USE Braille reading materials?

		FREQ	WTD
1	Yes	37	5,132
3	No	982	165,287
93	Not applicable	19,157	3,155,247
98	Not stated	534	94,671
R	Refusal	0	0
X	Don't know	0	0
		======	=========
		20,710	3,420,337

Coverage: Respondents who answered B25=1
Source: Participation and Activity Limitation Survey, 2001

Variable Name: **B26C** *Position:* 81 *Length:* 2
Collection Name: AB26C

Do you (Does.....) now USE large print reading materials?

		FREQ	WTD
1	Yes	403	62,140
3	No	625	107,543
93	Not applicable	19,157	3,155,247
98	Not stated	524	95,021
R	Refusal	0	0
X	Don't know	1	386
		======	=========
		20,710	3,420,337

Coverage: Respondents who answered B25=1
Source: Participation and Activity Limitation Survey, 2001

Variable Name: **B26D** *Position:* 83 *Length:* 2
Collection Name: AB26D

Do you (Does.....) now USE talking books?

		FREQ	WTD
1	Yes	177	24,178
3	No	848	147,035
93	Not applicable	19,157	3,155,247
98	Not stated	527	93,826
R	Refusal	0	0
X	Don't know	1	52
		======	=========
		20,710	3,420,338

Coverage: Respondents who answered B25=1
Source: Participation and Activity Limitation Survey, 2001

Variable Name: **B26E** *Position:* 85 *Length:* 2
Collection Name: AB26E

Do you (Does.....) now USE recording equipment or portable note-takers?

		FREQ	WTD
1	Yes	97	11,479
3	No	927	159,747
93	Not applicable	19,157	3,155,247
98	Not stated	528	93,813
R	Refusal	0	0
X	Don't know	1	52
		======	=========
		20,710	3,420,338

Coverage: Respondents who answered B25=1
Source: Participation and Activity Limitation Survey, 2001

Variable Name: **B26F** *Position:* 87 *Length:* 2
Collection Name: AB26F

Do you (Does.....) now USE closed circuit devices, e.g., CCTV's?

		FREQ	WTD
1	Yes	60	8,410
3	No	959	161,962
93	Not applicable	19,157	3,155,247
98	Not stated	529	94,292
R	Refusal	0	0
X	Don't know	5	427
		======	=========
		20,710	3,420,338

Coverage: Respondents who answered B25=1
Source: Participation and Activity Limitation Survey, 2001

Variable Name: **B26G** *Position:* 89 *Length:* 2
Collection Name: AB26G

Do you (Does.....) now USE a computer with Braille, large print or speech access?

		FREQ	WTD
1	Yes	88	10,932
3	No	934	159,799
93	Not applicable	19,157	3,155,247
98	Not stated	528	94,078
R	Refusal	0	0
X	Don't know	3	281
		======	=========
		20,710	3,420,337

Coverage: Respondents who answered B25=1
Source: Participation and Activity Limitation Survey, 2001

Variable Name:	**B26H**	*Position:*	91	*Length:*	2
Collection Name:	AB26H				

Do you (Does.....) now USE a white cane?

		FREQ	WTD
1	Yes	160	21,594
3	No	868	150,118
93	Not applicable	19,157	3,155,247
98	Not stated	525	93,378
R	Refusal	0	0
X	Don't know	0	0
		======	=========
		20,710	3,420,337

Coverage: Respondents who answered B25=1
Source: Participation and Activity Limitation Survey, 2001

Variable Name:	**B26_OTH**	*Position:*	93	*Length:*	2
Collection Name:	AB26_OTH				

Derived variable: Using guide dog/other aid?

		FREQ	WTD
1	Yes	115	14,613
3	No	907	154,845
93	Not applicable	19,660	3,244,535
98	Not stated	28	6,345
R	Refusal	0	0
X	Don't know	0	0
		======	=========
		20,710	3,420,338

Coverage: Respondents who answered B25=1
Source: Participation and Activity Limitation Survey, 2001
Note: B26_OTH is derived from variables B26I and B26J.

Variable Name: **B27** *Position:* 95 *Length:* 2
Collection Name: AB27

Are there any aids or specialized equipment for persons who are blind or visually impaired that you think you NEED (..... thinks he / she NEEDS) but do (does) not have?

		FREQ	WTD
1	Yes	441	70,857
3	No	2,707	450,662
93	Not applicable	16,958	2,792,665
98	Not stated	510	88,611
R	Refusal	0	0
X	Don't know	94	17,542
		======	=========
		20,710	3,420,337

Coverage: Respondents who reported a "seeing" limitation (SEELIM=1)
Source: Participation and Activity Limitation Survey, 2001

Variable Name: **B28** *Position:* 97 *Length:* 2
Collection Name: AB28

Which aids do you (does.....) NEED but do (does) not have?

		FREQ	WTD
0	Valid data	431	69,077
93	Not applicable	19,759	3,260,869
98	Not stated	511	88,650
R	Refusal	0	0
X	Don't know	9	1,742
		======	=========
		20,710	3,420,338

Coverage: Respondents who answered B27=1
Source: Participation and Activity Limitation Survey, 2001

Variable Name: **B28A** *Position:* 99 *Length:* 2
Collection Name: AB28A

Which aids do you (does.....) NEED but do (does) not have: Glasses, contact lenses, or a new prescription?

		FREQ	WTD
0	Not selected	20,465	3,376,175
1	Yes	245	44,163
		======	=========
		20,710	3,420,338

Coverage: Respondents who answered B27=1
Source: Participation and Activity Limitation Survey, 2001

Variable Name: **B28B** *Position:* 101 *Length:* 2
Collection Name: AB28B

Which aids do you (does.....) NEED but do (does) not have: Magnifiers?

		FREQ	WTD
0	Not selected	20,642	3,409,050
1	Yes	68	11,288
		======	=========
		20,710	3,420,338

Coverage: Respondents who answered B27=1
Source: Participation and Activity Limitation Survey, 2001

Variable Name: **B28C** *Position:* 103 *Length:* 2
Collection Name: AB28C

Which aids do you (does.....) NEED but do (does) not have: Braille reading materials?

		FREQ	WTD
0	Not selected	20,708	3,420,150
1	Yes	2	187
		======	=========
		20,710	3,420,337

Coverage: Respondents who answered B27=1
Source: Participation and Activity Limitation Survey, 2001

Variable Name: **B28D** *Position:* 105 *Length:* 2
Collection Name: AB28D

Which aids do you (does.....) NEED but do (does) not have: large print reading materials?

		FREQ	WTD
0	Not selected	20,667	3,412,511
1	Yes	43	7,827
		======	=========
		20,710	3,420,338

Coverage: Respondents who answered B27=1
Source: Participation and Activity Limitation Survey, 2001

Variable Name:	**B28E**	*Position:*	107	*Length:*	2
Collection Name:	AB28E				

Which aids do you (does.....) NEED but do (does) not have: Talking books?

		FREQ	WTD
0	Not selected	20,692	3,418,131
1	Yes	18	2,206
		======	=========
		20,710	3,420,337

Coverage: Respondents who answered B27=1
Source: Participation and Activity Limitation Survey, 2001

Variable Name:	**B28F**	*Position:*	109	*Length:*	2
Collection Name:	AB28F				

Which aids do you (does.....) NEED but do (does) not have: Recording equipment or portable note-takers?

		FREQ	WTD
0	Not selected	20,700	3,419,394
1	Yes	10	944
		======	=========
		20,710	3,420,338

Coverage: Respondents who answered B27=1
Source: Participation and Activity Limitation Survey, 2001

Variable Name:	**B28G**	*Position:*	111	*Length:*	2
Collection Name:	AB28G				

Which aids do you (does.....) NEED but do (does) not have: Closed circuit devices (CCTV's)?

		FREQ	WTD
0	Not selected	20,689	3,417,500
1	Yes	21	2,837
		======	=========
		20,710	3,420,337

Coverage: Respondents who answered B27=1
Source: Participation and Activity Limitation Survey, 2001

Variable Name:	**B28H**	*Position:*	113	*Length:*	2
Collection Name:	AB28H				

Which aids do you (does.....) NEED but do (does) not have: Computer with Braille, large print or speech access?

		FREQ	WTD
0	Not selected	20,681	3,417,483
1	Yes	29	2,854
		======	=========
		20,710	3,420,337

Coverage: Respondents who answered B27=1
Source: Participation and Activity Limitation Survey, 2001

Variable Name:	**B28I**	*Position:*	115	*Length:*	2
Collection Name:	AB28I				

Which aids do you (does.....) NEED but do (does) not have: white cane?

		FREQ	WTD
0	Not selected	20,702	3,419,127
1	Yes	8	1,211
		======	=========
		20,710	3,420,338

Coverage: Respondents who answered B27=1
Source: Participation and Activity Limitation Survey, 2001

Variable Name:	**B28_OTH**	*Position:*	117	*Length:*	2
Collection Name:	AB28_OTH				

Derived variable: Need, but do not have guide dog/other aid?

		FREQ	WTD
0	Not selected	20,626	3,411,209
1	Yes	84	9,128
		======	=========
		20,710	3,420,337

Coverage: Respondents who answered B27=1
Source: Participation and Activity Limitation Survey, 2001
Note: B28_OTH is derived from variables B28J and B28K.

Variable Name: **B36** *Position:* 119 *Length:* 2
Collection Name: AB36

Do you (Does.....) USE any aids or specialized equipment for persons who have difficulty speaking or making themselves understood, for example, a keyboard device to communicate?

		FREQ	WTD
1	Yes	109	11,684
3	No	2,318	326,878
93	Not applicable	17,946	3,019,006
98	Not stated	334	62,685
R	Refusal	0	0
X	Don't know	3	84
		======	=========
		20,710	3,420,337

Coverage: Respondents who reported a "speech" limitation (SPCHLIM=1)
Source: Participation and Activity Limitation Survey, 2001

Variable Name: **B38** *Position:* 121 *Length:* 2
Collection Name: AB38

Are there any aids or specialized equipment for persons who have difficulty speaking or making themselves understood that you think you NEED (.....thinks he / she NEEDS) but do (does) not have?

		FREQ	WTD
1	Yes	119	11,586
3	No	2,197	313,287
93	Not applicable	17,946	3,019,006
98	Not stated	339	63,088
R	Refusal	0	0
X	Don't know	109	13,371
		======	=========
		20,710	3,420,338

Coverage: Respondents who reported a "speech" limitation (SPCHLIM=1)
Source: Participation and Activity Limitation Survey, 2001

Variable Name:	**B51**	*Position:*	123	*Length:*	2
Collection Name:	AB51				

Do you (Does.....) USE any aids or specialized equipment for persons who have difficulty moving around?

		FREQ	WTD
1	Yes	5,233	970,495
3	No	8,193	1,403,806
93	Not applicable	6,769	955,030
98	Not stated	511	90,805
R	Refusal	0	0
X	Don't know	4	201
		======	=========
		20,710	3,420,337

Coverage: Respondents who reported a "mobility" limitation (MOBLIM=1)
Source: Participation and Activity Limitation Survey, 2001

Variable Name:	**B52A**	*Position:*	125	*Length:*	2
Collection Name:	AB52A				

Do you (Does.....) now USE orthopaedic footwear?

		FREQ	WTD
1	Yes	1,044	199,121
3	No	3,865	711,314
93	Not applicable	14,966	2,359,038
98	Not stated	828	149,904
R	Refusal	0	0
X	Don't know	7	961
		======	=========
		20,710	3,420,338

Coverage: Respondents who answered B51=1
Source: Participation and Activity Limitation Survey, 2001

Variable Name: **B52B** *Position:* 127 *Length:* 2
Collection Name: AB52B

Do you (Does.....) now USE a cane or walking stick?

		FREQ	WTD
1	Yes	3,163	637,208
3	No	1,784	281,822
93	Not applicable	14,966	2,359,038
98	Not stated	797	142,269
R	Refusal	0	0
X	Don't know	0	0
		======	=========
		20,710	3,420,337

Coverage: Respondents who answered B51=1
Source: Participation and Activity Limitation Survey, 2001

Variable Name: **B52C** *Position:* 129 *Length:* 2
Collection Name: AB52C

Do you (Does.....) now USE crutches?

		FREQ	WTD
1	Yes	313	59,454
3	No	4,559	839,626
93	Not applicable	14,966	2,359,038
98	Not stated	872	162,221
R	Refusal	0	0
X	Don't know	0	0
		======	=========
		20,710	3,420,339

Coverage: Respondents who answered B51=1
Source: Participation and Activity Limitation Survey, 2001

Variable Name: **B52F** *Position:* 131 *Length:* 2
Collection Name: AB52F

Do you (Does.....) now USE a walker?

		FREQ	WTD
1	Yes	1,354	266,641
3	No	3,542	639,385
93	Not applicable	14,966	2,359,038
98	Not stated	847	155,232
R	Refusal	0	0
X	Don't know	1	43
		======	=========
		20,710	3,420,339

Coverage: Respondents who answered B51=1
Source: Participation and Activity Limitation Survey, 2001

Variable Name: **B52G** *Position:* 133 *Length:* 2
Collection Name: AB52G

Do you (Does.....) now USE a scooter?

		FREQ	WTD
1	Yes	210	43,614
3	No	4,670	859,788
93	Not applicable	14,966	2,359,038
98	Not stated	863	157,856
R	Refusal	0	0
X	Don't know	1	43
		======	=========
		20,710	3,420,339

Coverage: Respondents who answered B51=1
Source: Participation and Activity Limitation Survey, 2001

Variable Name: **B52H** *Position:* 135 *Length:* 2
Collection Name: AB52H

Do you (Does.....) now USE braces or supportive devices?

		FREQ	WTD
1	Yes	1,133	188,306
3	No	3,867	740,319
93	Not applicable	14,966	2,359,038
98	Not stated	740	132,116
R	Refusal	0	0
X	Don't know	4	560
		======	=========
		20,710	3,420,339

Coverage: Respondents who answered B51=1
Source: Participation and Activity Limitation Survey, 2001

Variable Name: **B52I** *Position:* 137 *Length:* 2
Collection Name: AB52I

Do you (Does.....) now USE lifts or lift type devices?

		FREQ	WTD
1	Yes	443	64,630
3	No	4,432	836,381
93	Not applicable	14,966	2,359,038
98	Not stated	867	160,161
R	Refusal	0	0
X	Don't know	2	128
		======	=========
		20,710	3,420,338

Coverage: Respondents who answered B51=1
Source: Participation and Activity Limitation Survey, 2001

Variable Name: **B52J** *Position:* 139 *Length:* 2
Collection Name: AB52J

Do you (Does.....) now USE grab bars or bathroom aids?

		FREQ	WTD
1	Yes	2,427	429,831
3	No	2,497	476,880
93	Not applicable	14,966	2,359,038
98	Not stated	816	154,333
R	Refusal	0	0
X	Don't know	4	256
		======	=========
		20,710	3,420,338

Coverage: Respondents who answered B51=1
Source: Participation and Activity Limitation Survey, 2001

Variable Name: **B52K** *Position:* 141 *Length:* 2
Collection Name: AB52K

Do you (Does.....) now USE other aid?

		FREQ	WTD
1	Yes	590	93,640
3	No	4,231	796,908
93	Not applicable	14,966	2,359,038
98	Not stated	919	170,223
R	Refusal	0	0
X	Don't know	4	529
		======	=========
		20,710	3,420,338

Coverage: Respondents who answered B51=1
Source: Participation and Activity Limitation Survey, 2001

Variable Name:	**B52DE**	*Position:*	143	*Length:*	2
Collection Name:	AB52DE				

Derived variable: Using Manual/Electric wheelchair?

		FREQ	WTD
1	Yes	1,076	175,249
3	No	3,806	728,527
93	Not applicable	15,477	2,449,843
98	Not stated	351	66,719
R	Refusal	0	0
X	Don't know	0	0
		======	=========
		20,710	3,420,338

Coverage: Respondents who answered B51=1
Source: Participation and Activity Limitation Survey, 2001
Note: B52DE is derived from variables B52D, B52E.

Variable Name:	**B53**	*Position:*	145	*Length:*	2
Collection Name:	AB53				

Are there any aids or specialized equipment for persons who have difficulty moving around that you think you NEED (..... thinks he / she NEEDS) but do (does) not have?

		FREQ	WTD
1	Yes	1,556	297,404
3	No	11,616	2,033,937
93	Not applicable	6,769	955,030
98	Not stated	551	95,026
R	Refusal	0	0
X	Don't know	218	38,941
		======	=========
		20,710	3,420,338

Coverage: Respondents who reported a "mobility" limitation (MOBLIM=1)
Source: Participation and Activity Limitation Survey, 2001

Variable Name: **B54** *Position:* 147 *Length:* 2
Collection Name: AB54

Which aids do you (does.....) NEED but do (does) not have?

		FREQ	WTD
0	Valid data	1,524	291,748
93	Not applicable	18,603	3,027,907
98	Not stated	566	99,059
R	Refusal	2	189
X	Don't know	15	1,433
		======	=========
		20,710	3,420,336

Coverage: Respondents who answered B53=1
Source: Participation and Activity Limitation Survey, 2001

Variable Name: **B54A** *Position:* 149 *Length:* 2
Collection Name: AB54A

Which aids do you (does.....) NEED but do (does) not have: orthopaedic footwear?

		FREQ	WTD
0	Not selected	20,536	3,390,336
1	Yes	174	30,002
		======	=========
		20,710	3,420,338

Coverage: Respondents who answered B53=1
Source: Participation and Activity Limitation Survey, 2001

Variable Name: **B54B** *Position:* 151 *Length:* 2
Collection Name: AB54B

Which aids do you (does.....) NEED but do (does) not have: Cane or walking stick?

		FREQ	WTD
0	Not selected	20,472	3,376,626
1	Yes	238	43,712
		======	=========
		20,710	3,420,338

Coverage: Respondents who answered B53=1
Source: Participation and Activity Limitation Survey, 2001

Variable Name:	**B54C**	*Position:*	153	*Length:*	2
Collection Name:	AB54C				

Which aids do you (does.....) NEED but do (does) not have: crutches?

		FREQ	WTD
0	Not selected	20,694	3,417,198
1	Yes	16	3,140
		======	=========
		20,710	3,420,338

Coverage: Respondents who answered B53=1
Source: Participation and Activity Limitation Survey, 2001

Variable Name:	**B54F**	*Position:*	155	*Length:*	2
Collection Name:	AB54F				

Which aids do you (does.....) NEED but do (does) not have: Walker?

		FREQ	WTD
0	Not selected	20,540	3,390,818
1	Yes	170	29,520
		======	=========
		20,710	3,420,338

Coverage: Respondents who answered B53=1
Source: Participation and Activity Limitation Survey, 2001

Variable Name:	**B54G**	*Position:*	157	*Length:*	2
Collection Name:	AB54G				

Which aids do you (does.....) NEED but do (does) not have: scooter?

		FREQ	WTD
0	Not selected	20,528	3,372,666
1	Yes	182	47,672
		======	=========
		20,710	3,420,338

Coverage: Respondents who answered B53=1
Source: Participation and Activity Limitation Survey, 2001

Variable Name: **B54H** *Position:* 159 *Length:* 2
Collection Name: AB54H

Which aids do you (does.....) NEED but do (does) not have: Braces or supportive devices?

		FREQ	WTD
0	Not selected	20,595	3,401,868
1	Yes	115	18,470
		======	=========
		20,710	3,420,338

Coverage: Respondents who answered B53=1
Source: Participation and Activity Limitation Survey, 2001

Variable Name: **B54I** *Position:* 161 *Length:* 2
Collection Name: AB54I

Which aids do you (does.....) NEED but do (does) not have: Lifts or lift type devices?

		FREQ	WTD
0	Not selected	20,534	3,383,894
1	Yes	176	36,444
		======	=========
		20,710	3,420,338

Coverage: Respondents who answered B53=1
Source: Participation and Activity Limitation Survey, 2001

Variable Name: **B54J** *Position:* 163 *Length:* 2
Collection Name: AB54J

Which aids do you (does.....) NEED but do (does) not have: Grab bars or bathroom aids?

		FREQ	WTD
0	Not selected	20,260	3,336,737
1	Yes	450	83,601
		======	=========
		20,710	3,420,338

Coverage: Respondents who answered B53=1
Source: Participation and Activity Limitation Survey, 2001

Variable Name: **B54K** *Position:* 165 *Length:* 2
Collection Name: AB54K

Which aids do you (does.....) NEED but do (does) not have: Other?

		FREQ	WTD
0	Not selected	20,393	3,369,390
1	Yes	317	50,948
		======	=========
		20,710	3,420,338

Coverage: Respondents who answered B53=1
Source: Participation and Activity Limitation Survey, 2001

Variable Name: **B54DE** *Position:* 167 *Length:* 2
Collection Name: AB54DE

Derived variable: Need, but do not have Manual/Electric wheelchair?

		FREQ	WTD
0	Not selected	20,519	3,384,541
1	Yes	191	35,797
		======	=========
		20,710	3,420,338

Coverage: Respondents who answered B53=1
Source: Participation and Activity Limitation Survey, 2001
Note: B54DE is derived from variables B54D and B54E.

Variable Name: **B69** *Position:* 169 *Length:* 2
Collection Name: AB69

Do you (Does.....) USE any aids or specialized equipment designed to support, replace or assist in the use of hands or arms?

		FREQ	WTD
1	Yes	1,088	201,970
3	No	11,180	1,964,886
93	Not applicable	7,697	1,123,698
98	Not stated	734	126,843
R	Refusal	0	0
X	Don't know	11	2,941
		======	=========
		20,710	3,420,338

Coverage: Respondents who reported an "agility" limitation (AGILIM=1)
Source: Participation and Activity Limitation Survey, 2001

Variable Name: **B70A** *Position:* 171 *Length:* 2
Collection Name: AB70A

Do you (Does.....) now USE a hand or arm brace?

		FREQ	WTD
1	Yes	422	74,666
3	No	600	114,911
93	Not applicable	18,888	3,091,525
98	Not stated	800	139,235
R	Refusal	0	0
X	Don't know	0	0
		======	=========
		20,710	3,420,337

Coverage: Respondents who answered B69=1
Source: Participation and Activity Limitation Survey, 2001

Variable Name: **B70B** *Position:* 173 *Length:* 2
Collection Name: AB70B

Do you (Does.....) now USE grasping tools or reach extenders?

		FREQ	WTD
1	Yes	525	101,185
3	No	497	88,140
93	Not applicable	18,888	3,091,525
98	Not stated	799	139,378
R	Refusal	0	0
X	Don't know	1	110
		======	=========
		20,710	3,420,338

Coverage: Respondents who answered B69=1
Source: Participation and Activity Limitation Survey, 2001

Variable Name: **B70C** *Position:* 175 *Length:* 2
Collection Name: AB70C

Do you (Does.....) now USE another aid?

		FREQ	WTD
1	Yes	353	62,084
3	No	684	132,159
93	Not applicable	18,888	3,091,525
98	Not stated	781	133,460
R	Refusal	0	0
X	Don't know	4	1,109
		======	=========
		20,710	3,420,337

Coverage: Respondents who answered B69=1
Source: Participation and Activity Limitation Survey, 2001

Variable Name:	**B71**	*Position:*	177	*Length:*	2
Collection Name:	AB71				

Are there any aids or specialized equipment designed to support, replace or assist in the use of hands or arms that you think you NEED (..... thinks he / she NEEDS) but do (does) not have?

		FREQ	WTD
1	Yes	453	77,745
3	No	11,490	2,037,718
93	Not applicable	7,697	1,123,698
98	Not stated	773	134,595
R	Refusal	0	0
X	Don't know	297	46,582
		======	=========
		20,710	3,420,338

Coverage: Respondents who reported an "agility" limitation (AGILIM=1)
Source: Participation and Activity Limitation Survey, 2001

Variable Name:	**B72**	*Position:*	179	*Length:*	2
Collection Name:	AB72				

Which aids do you (does.....) NEED but do (does) not have?

		FREQ	WTD
0	Valid data	403	69,128
93	Not applicable	19,484	3,207,997
98	Not stated	783	136,404
R	Refusal	0	0
X	Don't know	40	6,809
		======	=========
		20,710	3,420,338

Coverage: Respondents who answered B71=1
Source: Participation and Activity Limitation Survey, 2001

Variable Name:	**B72A**	*Position:*	181	*Length:*	2
Collection Name:	AB72A				

Which aids do you (does.....) NEED but do (does) not have: Hand or arm brace?

		FREQ	WTD
0	Not selected	20,622	3,407,571
1	Yes	88	12,766
		======	=========
		20,710	3,420,337

Coverage: Respondents who answered B71=1
Source: Participation and Activity Limitation Survey, 2001

Variable Name:	**B72B**	*Position:*	183	*Length:*	2
Collection Name:	AB72B				

Which aids do you (does.....) NEED but do (does) not have: Grasping tools or reach extenders?

		FREQ	WTD
0	Not selected	20,518	3,384,924
1	Yes	192	35,414
		======	=========
		20,710	3,420,338

Coverage: Respondents who answered B71=1
Source: Participation and Activity Limitation Survey, 2001

Variable Name:	**B72C**	*Position:*	185	*Length:*	2
Collection Name:	AB72C				

Which aids do you (does.....) NEED but do (does) not have: other aid?

		FREQ	WTD
0	Not selected	20,548	3,394,172
1	Yes	162	26,166
		======	=========
		20,710	3,420,338

Coverage: Respondents who answered B71=1
Source: Participation and Activity Limitation Survey, 2001

Variable Name:	**B94**	*Position:*	187	*Length:*	2
Collection Name:	AB94				

You reported that because of a physical condition, mental condition or health problem, you have (..... has) difficulties or limitations in doing certain activities. Do you (Does.....) think that these difficulties or activity limitations create a disadvantage for you (him/ her) at home?

		FREQ	WTD
1	Yes, sometimes	7,470	1,241,323
2	Yes, often or always	4,714	831,146
3	No	7,898	1,249,160
4	Yes, undetermined	62	10,211
93	Not applicable (Persons without disabilities)	0	0
98	Not stated	437	66,717
R	Refusal	3	515
X	Don't know	126	21,265
		======	=========
		20,710	3,420,337

Coverage: All respondents (DISAB=1)
Source: Participation and Activity Limitation Survey, 2001

Variable Name:	**B95**	*Position:*	189	*Length:*	2
Collection Name:	AB95				

How much of a disadvantage?

		FREQ	WTD
1	Mild	4,295	687,721
2	Moderate	5,461	968,700
3	Severe	2,332	397,617
93	Not applicable	8,027	1,270,940
98	Not stated	507	80,276
R	Refusal	1	17
X	Don't know	87	15,065
		======	=========
		20,710	3,420,336

Coverage: Respondents (DISAB=1) who answered B94=1
Source: Participation and Activity Limitation Survey, 2001

Variable Name:	**B96**	*Position:*	191	*Length:*	2
Collection Name:	AB96				

Do you (Does.....) think that these difficulties or activity limitations create a disadvantage for you (him/ her) at work?

		FREQ	WTD
1	Yes, sometimes	2,926	404,221
2	Yes, often or always	1,759	257,447
3	No	2,691	387,694
4	Yes, undetermined	27	3,878
5	Not Applicable (Work)	12,816	2,293,502
93	Not applicable (Persons without disabilities)	0	0
98	Not stated	441	67,568
R	Refusal	1	37
X	Don't know	49	5,991
		======	=========
		20,710	3,420,338

Coverage: All respondents (DISAB=1)
Source: Participation and Activity Limitation Survey, 2001

Variable Name:	**B97**	*Position:*	193	*Length:*	2
Collection Name:	B97				

How much of a disadvantage?

		FREQ	WTD
1	Mild	1,849	247,222
2	Moderate	1,849	264,210
3	Severe	943	144,685
93	Not applicable	15,557	2,687,224
98	Not stated	495	75,309
R	Refusal	0	0
X	Don't know	17	1,688
		======	=========
		20,710	3,420,338

Coverage: Respondents (DISAB=1) who answered B96=1
Source: Participation and Activity Limitation Survey, 2001

Variable Name:	**B98**	*Position:*	195	*Length:*	2
Collection Name:	AB98				

Do you (Does.....) think that these difficulties or activity limitations create a disadvantage for you (him/ her) at school?

		FREQ	WTD
1	Yes, sometimes	878	68,985
2	Yes, often or always	653	48,067
3	No	1,175	132,320
4	Yes, undetermined	16	519
5	Not Applicable (School)	17,474	3,090,131
93	Not applicable (Persons without disabilities)	0	0
98	Not stated	473	75,411
R	Refusal	1	37
X	Don't know	40	4,869
		======	=========
		20,710	3,420,339

Coverage: All respondents (DISAB=1)
Source: Participation and Activity Limitation Survey, 2001

Variable Name:	**B99**	*Position:*	197	*Length:*	2
Collection Name:	AB99				

How much of a disadvantage?

		FREQ	WTD
1	Mild	552	40,246
2	Moderate	617	44,927
3	Severe	333	29,610
93	Not applicable	18,690	3,227,356
98	Not stated	507	77,742
R	Refusal	0	0
X	Don't know	11	457
		======	=========
		20,710	3,420,338

Coverage: Respondents (DISAB=1) who answered B98=1
Source: Participation and Activity Limitation Survey, 2001

Variable Name:	**B100**	*Position:*	199	*Length:*	2
Collection Name:	AB100				

Do you (Does.....) think that these difficulties or activity limitations create a disadvantage for you (him/ her) in other areas, such as transportation or leisure?

		FREQ	WTD
1	Yes, sometimes	7,068	1,145,427
2	Yes, often or always	5,641	971,031
3	No	7,091	1,131,476
4	Yes, undetermined	52	11,986
5	Not Applicable (Transportation or leisure)	289	59,713
93	Not applicable (Persons without disabilities)	0	0
98	Not stated	455	74,605
R	Refusal	0	0
X	Don't know	114	26,099
		======	=========
		20,710	3,420,337

Coverage: All respondents (DISAB=1)
Source: Participation and Activity Limitation Survey, 2001

Variable Name: **B101** *Position:* 201 *Length:* 2
Collection Name: AB101

How much of a disadvantage?

		FREQ	WTD
1	Mild	4,194	670,732
2	Moderate	5,472	927,663
3	Severe	2,915	501,513
93	Not applicable	7,494	1,217,288
98	Not stated	563	88,872
R	Refusal	1	54
X	Don't know	71	14,216
		======	=========
		20,710	3,420,338

Coverage: Respondents (DISAB=1) who answered B100=1
Source: Participation and Activity Limitation Survey, 2001

Variable Name: **B104** *Position:* 203 *Length:* 2
Collection Name: AB104

Did this condition exist at birth?

		FREQ	WTD
1	Yes	2,229	238,102
3	No	16,230	2,817,019
93	Not applicable	318	59,522
98	Not stated	1,189	191,877
R	Refusal	0	0
X	Don't know	744	113,818
		======	=========
		20,710	3,420,338

Coverage: Respondents (DISAB=1) who reported a valid response in B103
Source: Participation and Activity Limitation Survey, 2001

Variable Name:	**B105**	*Position:*	205	*Length:*	2
Collection Name:	AB105				

Which one of the following best describes the CAUSE of this condition?

		FREQ	WTD
1	A disease or illness	4,377	770,436
2	Ageing	2,598	537,311
3	Work conditions	1,843	344,398
4	Stress	808	134,817
5	An accident	3,255	513,070
6	Another cause, specify	1,994	299,910
93	Not applicable	2,547	297,624
98	Not stated	1,475	245,623
R	Refusal	2	170
X	Don't know	1,811	276,978
		======	=========
		20,710	3,420,337

Coverage: Respondents (DISAB=1) who reported a valid response in B103 and DID NOT answer B104=1
Source: Participation and Activity Limitation Survey, 2001

Variable Name:	**B107**	*Position:*	207	*Length:*	2
Collection Name:	AB107				

Is there a SECOND condition which causes you (.....) difficulty or limits your (his / her) activities?

		FREQ	WTD
1	Yes	8,445	1,474,034
3	No	10,564	1,660,053
93	Not applicable	318	59,522
98	Not stated	1,289	208,244
R	Refusal	1	294
X	Don't know	93	18,190
		======	=========
		20,710	3,420,337

Coverage: Respondents (DISAB=1) who reported a valid response in B103
Source: Participation and Activity Limitation Survey, 2001

Variable Name:	**B109**	*Position:*	209	*Length:*	2
Collection Name:	AB109				

Did this condition exist at birth?

		FREQ	WTD
1	Yes	644	73,300
3	No	7,337	1,320,943
93	Not applicable	10,994	1,740,121
98	Not stated	1,452	238,767
R	Refusal	0	0
X	Don't know	283	47,207
		======	=========
		20,710	3,420,338

Coverage: Respondents (DISAB=1) who reported a valid response in B108
Source: Participation and Activity Limitation Survey, 2001

Variable Name:	**B110**	*Position:*	211	*Length:*	2
Collection Name:	AB110				

Which one of the following best describes the CAUSE of this condition?

		FREQ	WTD
1	A disease or illness	2,259	434,327
2	Ageing	1,236	235,765
3	Work conditions	624	109,411
4	Stress	495	88,355
5	An accident	1,009	161,873
6	Another cause, specify	1,011	160,458
93	Not applicable	11,638	1,813,421
98	Not stated	1,573	261,428
R	Refusal	1	288
X	Don't know	864	155,011
		======	=========
		20,710	3,420,337

Coverage: Respondents (DISAB=1) who reported a valid response in B108 and DID NOT answer B109=1
Source: Participation and Activity Limitation Survey, 2001

Variable Name: **B112** *Position:* 213 *Length:* 2
Collection Name: AB112

Do you (Does.....) use any medication or drugs, prescription or non-prescription, on a regular basis, that is AT LEAST ONCE A WEEK?

		FREQ	WTD
1	Yes	15,910	2,781,805
3	No	4,282	556,544
93	Not applicable	0	0
98	Not stated	464	72,973
R	Refusal	10	1,682
X	Don't know	44	7,333
		======	=========
		20,710	3,420,337

Coverage: All respondents (DISAB=1)
Source: Participation and Activity Limitation Survey, 2001

Variable Name: **B113_R** *Position:* 215 *Length:* 1
Collection Name: AB113_R

Derived variable: Number of prescription drugs taken daily

		FREQ	WTD
0	No medication	1,310	200,680
1	1 medication	3,331	504,771
2	2 medications	3,070	506,282
3	3 medications	2,441	443,650
4	4 medications	1,837	348,817
5	5+ medications	3,773	752,269
6	Not applicable	4,336	565,560
7	Not stated	612	98,310
		======	=========
		20,710	3,420,339

Coverage: Respondents (DISAB=1) who answered B112=1
Source: Participation and Activity Limitation Survey, 2001
Note: AB113_R is derived from variable B113.

Variable Name:	**B114_R**	*Position:*	216	*Length:*	1
Collection Name:	AB114_R				

Derived variable: Number of non-prescription drugs taken daily

		FREQ	WTD
0	No medication	8,975	1,599,875
1	1 medication	4,109	679,055
2	2 medications	1,376	242,813
3	3 medications	558	105,871
4	4 medications	286	50,675
5	5+ medications	403	71,440
6	Not applicable	4,336	565,560
7	Not stated	667	105,049
		======	=========
		20,710	3,420,338

Coverage: Respondents (DISAB=1) who answered B112=1
Source: Participation and Activity Limitation Survey, 2001
Note: AB114_R is derived from variable B114.

Variable Name:	**B115**	*Position:*	217	*Length:*	2
Collection Name:	AB115				

Are there any other kinds of medication or drugs you take (..... takes) regularly BUT NOT DAILY?

		FREQ	WTD
1	Yes	4,679	802,074
3	No	11,113	1,954,405
93	Not applicable	4,336	565,560
98	Not stated	533	85,267
R	Refusal	3	946
X	Don't know	46	12,087
		======	=========
		20,710	3,420,339

Coverage: Respondents (DISAB=1) who answered B112=1
Source: Participation and Activity Limitation Survey, 2001

Variable Name: **B116_R** *Position:* 219 *Length:* 1
Collection Name: AB116_R

Derived variable: Number of prescription drugs taken regularly

		FREQ	WTD
0	No medication	1,926	319,043
1	1 medication	1,885	320,610
2	2 medications	493	93,543
3	3 medications	139	27,195
4	4 medications	49	8,173
5	5+ medications	59	11,700
6	Not applicable	15,498	2,532,997
7	Not stated	661	107,077
		======	=========
		20,710	3,420,338

Coverage: Respondents (DISAB=1) who answered B115=1
Source: Participation and Activity Limitation Survey, 2001
Note: AB116_R is derived from variable B116.

Variable Name: **B117_R** *Position:* 220 *Length:* 1
Collection Name: AB117_R

Derived variable: Kind of non-prescription drugs taken regularly

		FREQ	WTD
0	No medication	1,577	289,233
1	1 medication	2,322	373,085
2	2 medications	494	77,751
3	3 medications	115	26,856
4	4 medications	30	10,193
5	5+ medications	50	7,325
6	Not applicable	15,498	2,532,997
7	Not stated	624	102,898
		======	=========
		20,710	3,420,338

Coverage: Respondents (DISAB=1) who answered B115=1
Source: Participation and Activity Limitation Survey, 2001
Note: AB117_R is derived from variable B117.

Variable Name: **B118** *Position:* 221 *Length:* 2
Collection Name: AB118

In the past 12 months, did you (.....) have any OUT-OF-POCKET or DIRECT EXPENSES for prescription and non-prescription drugs?

		FREQ	WTD
1	Yes	12,433	2,126,428
3	No	7,501	1,159,026
93	Not applicable	0	0
98	Not stated	521	88,075
R	Refusal	8	2,230
X	Don't know	247	44,578
		======	=========
		20,710	3,420,337

Coverage: All respondents (DISAB=1)
Source: Participation and Activity Limitation Survey, 2001

Variable Name: **B121** *Position:* 223 *Length:* 2
Collection Name: AB121

In the past 12 months, were you (was.....) ever UNABLE TO GET the medication or drugs you were (he / she was) supposed to use, because of the COST?

		FREQ	WTD
1	Yes	2,200	355,249
3	No	17,597	2,897,473
93	Not applicable	0	0
98	Not stated	855	157,503
R	Refusal	5	1,538
X	Don't know	53	8,576
		======	=========
		20,710	3,420,339

Coverage: All respondents (DISAB=1)
Source: Participation and Activity Limitation Survey, 2001

Variable Name: **B122** *Position:* 225 *Length:* 2
Collection Name: AB122

In the past 12 months, did you (.....) ever use your (his / her) medication or drugs LESS OFTEN than you were (he / she was) supposed to, because of the COST?

		FREQ	WTD
1	Yes	2,362	372,692
3	No	17,753	2,941,859
93	Not applicable	0	0
98	Not stated	533	96,036
R	Refusal	4	1,042
X	Don't know	58	8,709
		======	=========
		20,710	3,420,338

Coverage: All respondents (DISAB=1)
Source: Participation and Activity Limitation Survey, 2001

Variable Name: **B123** *Position:* 227 *Length:* 2
Collection Name: AB123

In the past 12 months, did you (.....) ever NOT USE the medication or drugs you were (he / she was) supposed to use, because of the SIDE EFFECTS?

		FREQ	WTD
1	Yes	4,640	773,072
3	No	15,398	2,524,357
93	Not applicable	0	0
98	Not stated	544	93,955
R	Refusal	6	1,583
X	Don't know	122	27,370
		======	=========
		20,710	3,420,337

Coverage: All respondents (DISAB=1)
Source: Participation and Activity Limitation Survey, 2001

Variable Name:	**B126**	*Position:*	229	*Length:*	2
Collection Name:	AB126				

In the past 12 months, did you (.....) have any OUT-OF-POCKET or DIRECT EXPENSES for the purchase and maintenance of aids and specialized equipment? (INCLUDE amounts not covered by insurance such as exclusions, deductibles and expenses over limits. EXCLUDE payments for which you have (.....has) been or will be reimbursed by any insurance or government program.)

		FREQ	WTD
1	Yes	2,705	491,181
3	No	4,828	810,565
93	Not applicable	9,774	1,534,964
98	Not stated	3,307	566,165
R	Refusal	21	4,393
X	Don't know	75	13,070
		======	=========
		20,710	3,420,338

Coverage: Respondents (DISAB=1) who reported using aids and specialized equipment (USEAID=1)
Source: Participation and Activity Limitation Survey, 2001

Variable Name:	**B131A**	*Position:*	231	*Length:*	2
Collection Name:	AB131A				

Why do you (does.....) not have these aids? I will read you a list of possible reasons. Please answer yes or no to each: not covered by insurance?

		FREQ	WTD
1	Yes	1,482	236,689
3	No	1,333	211,503
93	Not applicable	14,883	2,447,502
98	Not stated	2,854	495,407
R	Refusal	0	0
X	Don't know	158	29,237
		======	=========
		20,710	3,420,338

Coverage: Respondents (DISAB=1) who reported needing aids and specialized equipment (NEEDAID=1)
Source: Participation and Activity Limitation Survey, 2001

Variable Name:	**B131B**	*Position:*	233	*Length:*	2
Collection Name:	AB131B				

Why do you (does.....) not have these aids? I will read you a list of possible reasons. Please answer yes or no to each: too expensive?

		FREQ	WTD
1	Yes	1,969	315,191
3	No	926	153,327
93	Not applicable	14,883	2,447,502
98	Not stated	2,845	490,995
R	Refusal	0	0
X	Don't know	87	13,322
		======	=========
		20,710	3,420,337

Coverage: Respondents (DISAB=1) who reported needing aids and specialized equipment (NEEDAID=1)
Source: Participation and Activity Limitation Survey, 2001

Variable Name:	**B131C**	*Position:*	235	*Length:*	2
Collection Name:	AB131C				

Why do you (does.....) not have these aids? I will read you a list of possible reasons. Please answer yes or no to each: your (.....'s) condition is not serious enough?

		FREQ	WTD
1	Yes	463	69,449
3	No	2,415	390,591
93	Not applicable	14,883	2,447,502
98	Not stated	2,864	497,370
R	Refusal	0	0
X	Don't know	85	15,425
		======	=========
		20,710	3,420,337

Coverage: Respondents (DISAB=1) who reported needing aids and specialized equipment (NEEDAID=1)
Source: Participation and Activity Limitation Survey, 2001

Variable Name:	**B131D**	*Position:*	237	*Length:*	2
Collection Name:	AB131D				

Why do you (does.....) not have these aids? I will read you a list of possible reasons. Please answer yes or no to each: you do (.....does) not know where or how to obtain it?

		FREQ	WTD
1	Yes	542	82,625
3	No	2,387	386,303
93	Not applicable	14,883	2,447,502
98	Not stated	2,858	494,638
R	Refusal	0	0
X	Don't know	40	9,269
		======	=========
		20,710	3,420,337

Coverage: Respondents (DISAB=1) who reported needing aids and specialized equipment (NEEDAID=1)
Source: Participation and Activity Limitation Survey, 2001

Variable Name:	**B131E**	*Position:*	239	*Length:*	2
Collection Name:	AB131E				

Why do you (does.....) not have these aids? I will read you a list of possible reasons. Please answer yes or no to each: It is not available?

		FREQ	WTD
1	Yes	208	26,303
3	No	2,617	424,322
93	Not applicable	14,883	2,447,502
98	Not stated	2,864	495,837
R	Refusal	0	0
X	Don't know	138	26,373
		======	=========
		20,710	3,420,337

Coverage: Respondents (DISAB=1) who reported needing aids and specialized equipment (NEEDAID=1)
Source: Participation and Activity Limitation Survey, 2001

Variable Name: **B131F** *Position:* 241 *Length:* 2
Collection Name: AB131F

Why do you (does.....) not have these aids? I will read you a list of possible reasons. Please answer yes or no to each: Another reason?

		FREQ	WTD
1	Yes	784	123,458
3	No	2,126	347,062
93	Not applicable	14,883	2,447,502
98	Not stated	2,883	496,971
R	Refusal	0	0
X	Don't know	34	5,344
		======	=========
		20,710	3,420,337

Coverage: Respondents (DISAB=1) who reported needing aids and specialized equipment (NEEDAID=1)
Source: Participation and Activity Limitation Survey, 2001

Section: **HELP WITH DAILY ACTIVITY**

Variable Name: **C1** *Position:* 243 *Length:* 2
Collection Name: AC1

Because of your (his / her) condition, do you (does.....) usually RECEIVE help with preparing meals?

		FREQ	WTD
1	Yes	4,784	782,113
3	No	15,369	2,541,893
93	Not applicable	0	0
98	Not stated	548	94,508
R	Refusal	1	283
X	Don't know	8	1,541
		======	=========
		20,710	3,420,338

Coverage: All respondents (DISAB=1)
Source: Participation and Activity Limitation Survey, 2001

Variable Name:	**C2**	*Position:* 245	*Length:* 2
Collection Name:	AC2		

Do you think you NEED (Does..... think he / she NEEDS) help with preparing meals?

		FREQ	WTD
1	Yes	394	62,069
3	No	14,905	2,462,782
93	Not applicable	4,793	783,937
98	Not stated	606	109,504
R	Refusal	0	0
X	Don't know	12	2,047
		======	=========
		20,710	3,420,339

Coverage: Respondents (DISAB=1) who answered C1=3
Source: Participation and Activity Limitation Survey, 2001

Variable Name:	**C3A**	*Position:* 247	*Length:* 2
Collection Name:	AC3A		

WHO usually helps you (.....) with preparing meals? I will read you a list. Please answer yes or no to each. Family living with you (him / her)?

		FREQ	WTD
1	Yes	3,689	590,415
3	No	964	168,419
93	Not applicable	15,378	2,543,717
98	Not stated	678	117,753
R	Refusal	0	0
X	Don't know	1	34
		======	=========
		20,710	3,420,338

Coverage: Respondents (DISAB=1) who answered C1=1
Source: Participation and Activity Limitation Survey, 2001

Variable Name:	**C3AA**	*Position:*	249	*Length:*	2
Collection Name:	AC3AA				

HOW OFTEN family help with preparing meals?

		FREQ	WTD
1	Every day	3,004	493,763
2	At least once a week	569	79,922
3	Less than once a week	95	13,536
93	Not applicable	16,343	2,712,170
98	Not stated	696	120,053
R	Refusal	0	0
X	Don't know	3	893
		======	=========
		20,710	3,420,337

Coverage: Respondents (DISAB=1) who answered C3A=1
Source: Participation and Activity Limitation Survey, 2001

Variable Name:	**C3B**	*Position:*	251	*Length:*	2
Collection Name:	AC3B				

WHO usually helps you (.....) with preparing meals? I will read you a list. Please answer yes or no to each. Family not living with you (him / her)?

		FREQ	WTD
1	Yes	922	141,100
3	No	3,668	607,790
93	Not applicable	15,378	2,543,717
98	Not stated	741	127,697
R	Refusal	0	0
X	Don't know	1	34
		======	=========
		20,710	3,420,338

Coverage: Respondents (DISAB=1) who answered C1=1
Source: Participation and Activity Limitation Survey, 2001

Variable Name: **C3AB** *Position:* 253 *Length:* 2
Collection Name: AC3AB

HOW OFTEN do they help with preparing meals?

		FREQ	WTD
1	Every day	188	33,241
2	At least once a week	422	62,461
3	Less than once a week	295	42,913
93	Not applicable	19,047	3,151,541
98	Not stated	752	129,172
R	Refusal	0	0
X	Don't know	6	1,009
		======	=========
		20,710	3,420,337

Coverage: Respondents (DISAB=1) who answered C3B=1
Source: Participation and Activity Limitation Survey, 2001

Variable Name: **C3C** *Position:* 255 *Length:* 2
Collection Name: AC3C

WHO usually helps you (.....) with preparing meals? I will read you a list. Please answer yes or no to each. Friends or neighbours?

		FREQ	WTD
1	Yes	429	72,885
3	No	4,150	674,916
93	Not applicable	15,378	2,543,717
98	Not stated	749	128,569
R	Refusal	0	0
X	Don't know	4	250
		======	=========
		20,710	3,420,337

Coverage: Respondents (DISAB=1) who answered C1=1
Source: Participation and Activity Limitation Survey, 2001

Variable Name: **C3AC** *Position:* 257 *Length:* 2
Collection Name: AC3AC

HOW OFTEN do they help with preparing meals?

		FREQ	WTD
1	Every day	90	22,881
2	At least once a week	158	22,318
3	Less than once a week	172	26,680
93	Not applicable	19,532	3,218,884
98	Not stated	755	129,287
R	Refusal	0	0
X	Don't know	3	289
		======	=========
		20,710	3,420,339

Coverage: Respondents (DISAB=1) who answered C3C=1
Source: Participation and Activity Limitation Survey, 2001

Variable Name: **C3D** *Position:* 259 *Length:* 2
Collection Name: AC3D

WHO usually helps you (.....) with preparing meals? I will read you a list. Please answer yes or no to each: Organization or agency (Include voluntary, private and government agencies)?

		FREQ	WTD
1	Yes	851	126,925
3	No	3,751	623,051
93	Not applicable	15,378	2,543,717
98	Not stated	727	126,476
R	Refusal	0	0
X	Don't know	3	169
		======	=========
		20,710	3,420,338

Coverage: Respondents (DISAB=1) who answered C1=1
Source: Participation and Activity Limitation Survey, 2001

Variable Name:	**C3AD**	*Position:*	261	*Length:*	2
Collection Name:	AC3AD				

HOW OFTEN do they help with preparing meals?

		FREQ	WTD
1	Every day	517	73,643
2	At least once a week	276	43,751
3	Less than once a week	47	7,953
93	Not applicable	19,132	3,166,937
98	Not stated	738	128,054
R	Refusal	0	0
X	Don't know	0	0
		======	=========
		20,710	3,420,338

Coverage: Respondents (DISAB=1) who answered C3D=1
Source: Participation and Activity Limitation Survey, 2001

Variable Name:	**C3E**	*Position:*	263	*Length:*	2
Collection Name:	AC3E				

WHO usually helps you (.....) with preparing meals? I will read you a list. Please answer yes or no to each: Other help?

		FREQ	WTD
1	Yes	226	33,938
3	No	4,331	708,676
93	Not applicable	15,378	2,543,717
98	Not stated	772	133,776
R	Refusal	0	0
X	Don't know	3	231
		======	=========
		20,710	3,420,338

Coverage: Respondents (DISAB=1) who answered C1=1
Source: Participation and Activity Limitation Survey, 2001

Variable Name: **C3AE** *Position:* 265 *Length:* 2
Collection Name: AC3AE

HOW OFTEN do they help with preparing meals?

		FREQ	WTD
1	Every day	155	24,257
2	At least once a week	46	5,984
3	Less than once a week	13	1,658
93	Not applicable	19,712	3,252,624
98	Not stated	784	135,815
R	Refusal	0	0
X	Don't know	0	0
		======	=========
		20,710	3,420,338

Coverage: Respondents (DISAB=1) who answered C3E=1
Source: Participation and Activity Limitation Survey, 2001

Variable Name: **C4** *Position:* 267 *Length:* 2
Collection Name: AC4

Do you (Does.....) need ADDITIONAL help with preparing meals?

		FREQ	WTD
1	Yes	520	79,351
3	No	4,086	676,566
93	Not applicable	15,378	2,543,717
98	Not stated	711	118,250
R	Refusal	0	0
X	Don't know	15	2,455
		======	=========
		20,710	3,420,339

Coverage: Respondents (DISAB=1) who answered C1=1
Source: Participation and Activity Limitation Survey, 2001

Variable Name: **C5** *Position:* 269 *Length:* 2
Collection Name: AC5

Because of your (his / her) condition, do you (does.....) usually RECEIVE help with everyday housework, such as dusting and tidying up?

		FREQ	WTD
1	Yes	6,471	1,101,632
3	No	13,598	2,209,631
93	Not applicable	0	0
98	Not stated	616	105,969
R	Refusal	0	0
X	Don't know	25	3,106
		======	=========
		20,710	3,420,338

Coverage: All respondents (DISAB=1)
Source: Participation and Activity Limitation Survey, 2001

Variable Name: **C6** *Position:* 271 *Length:* 2
Collection Name: AC6

Do you think you NEED (Does..... think he / she NEEDS) help with everyday housework?

		FREQ	WTD
1	Yes	930	159,627
3	No	12,567	2,034,702
93	Not applicable	6,496	1,104,737
98	Not stated	696	117,984
R	Refusal	0	0
X	Don't know	21	3,287
		======	=========
		20,710	3,420,337

Coverage: Respondents (DISAB=1) who answered C5=3
Source: Participation and Activity Limitation Survey, 2001

Variable Name: **C7A** *Position:* 273 *Length:* 2
Collection Name: AC7A

WHO usually helps you (.....) with everyday housework? I will read you a list. Please answer yes or no to each. Family living with you (her / him)?

		FREQ	WTD
1	Yes	4,183	683,524
3	No	2,100	381,900
93	Not applicable	13,623	2,212,737
98	Not stated	801	141,361
R	Refusal	0	0
X	Don't know	3	816
		======	=========
		20,710	3,420,338

Coverage: Respondents (DISAB=1) who answered C5=1
Source: Participation and Activity Limitation Survey, 2001

Variable Name: **C7AA** *Position:* 275 *Length:* 2
Collection Name: AC7AA

HOW OFTEN do they help with everyday housework?

		FREQ	WTD
1	Every day	3,032	503,981
2	At least once a week	969	151,569
3	Less than once a week	117	17,208
93	Not applicable	15,726	2,595,453
98	Not stated	858	151,165
R	Refusal	1	59
X	Don't know	7	903
		======	=========
		20,710	3,420,338

Coverage: Respondents (DISAB=1) who answered C7A=1
Source: Participation and Activity Limitation Survey, 2001

Variable Name:	**C7B**	*Position:*	277	*Length:*	2
Collection Name:	AC7B				

WHO usually helps you (.....) with everyday housework? I will read you a list. Please answer yes or no to each. Family not living with you (her / him)?

		FREQ	WTD
1	Yes	1,162	195,858
3	No	5,035	859,924
93	Not applicable	13,623	2,212,737
98	Not stated	877	150,093
R	Refusal	0	0
X	Don't know	13	1,725
		======	=========
		20,710	3,420,337

Coverage: Respondents (DISAB=1) who answered C5=1
Source: Participation and Activity Limitation Survey, 2001

Variable Name:	**C7AB**	*Position:*	279	*Length:*	2
Collection Name:	AC7AB				

HOW OFTEN do they help with everyday housework?

		FREQ	WTD
1	Every day	117	21,274
2	At least once a week	646	116,414
3	Less than once a week	373	54,817
93	Not applicable	18,671	3,074,386
98	Not stated	896	152,585
R	Refusal	1	59
X	Don't know	6	803
		======	=========
		20,710	3,420,338

Coverage: Respondents (DISAB=1) who answered C7B=1
Source: Participation and Activity Limitation Survey, 2001

Variable Name: **C7C** *Position:* 281 *Length:* 2
Collection Name: AC7C

WHO usually helps you (.....) with everyday housework? I will read you a list. Please answer yes or no to each: Friends or neighbours?

		FREQ	WTD
1	Yes	586	100,455
3	No	5,583	945,538
93	Not applicable	13,623	2,212,737
98	Not stated	905	159,473
R	Refusal	0	0
X	Don't know	13	2,135
		======	=========
		20,710	3,420,338

Coverage: Respondents (DISAB=1) who answered C5=1
Source: Participation and Activity Limitation Survey, 2001

Variable Name: **C7AC** *Position:* 283 *Length:* 2
Collection Name: AC7AC

HOW OFTEN do they help with everyday housework?

		FREQ	WTD
1	Every day	80	15,735
2	At least once a week	269	42,406
3	Less than once a week	226	38,392
93	Not applicable	19,219	3,160,409
98	Not stated	914	162,821
R	Refusal	0	0
X	Don't know	2	574
		======	=========
		20,710	3,420,337

Coverage: Respondents (DISAB=1) who answered C7C=1
Source: Participation and Activity Limitation Survey, 2001

Variable Name: **C7D** *Position:* 285 *Length:* 2
Collection Name: AC7D

WHO usually helps you (.....) with everyday housework? I will read you a list. Please answer yes or no to each: Organization or agency (Include voluntary, private and government agencies)?

		FREQ	WTD
1	Yes	1,372	247,464
3	No	4,825	804,635
93	Not applicable	13,623	2,212,737
98	Not stated	876	154,285
R	Refusal	1	29
X	Don't know	13	1,188
		======	=========
		20,710	3,420,338

Coverage: Respondents (DISAB=1) who answered C5=1
Source: Participation and Activity Limitation Survey, 2001

Variable Name: **C7AD** *Position:* 287 *Length:* 2
Collection Name: AC7AD

HOW OFTEN do they help with everyday housework?

		FREQ	WTD
1	Every day	352	48,590
2	At least once a week	605	118,949
3	Less than once a week	385	73,349
93	Not applicable	18,462	3,018,589
98	Not stated	903	160,685
R	Refusal	0	0
X	Don't know	3	175
		======	=========
		20,710	3,420,337

Coverage: Respondents (DISAB=1) who answered C7D=1
Source: Participation and Activity Limitation Survey, 2001

Variable Name: **C7E** *Position:* 289 *Length:* 2
Collection Name: AC7E

WHO usually helps you (.....) with everyday housework? I will read you a list. Please answer yes or no to each. Other help?

		FREQ	WTD
1	Yes	694	131,231
3	No	5,476	916,522
93	Not applicable	13,623	2,212,737
98	Not stated	902	157,711
R	Refusal	0	0
X	Don't know	15	2,136
		======	=========
		20,710	3,420,337

Coverage: Respondents (DISAB=1) who answered C5=1
Source: Participation and Activity Limitation Survey, 2001

Variable Name: **C7AE** *Position:* 291 *Length:* 2
Collection Name: AC7AE

HOW OFTEN do they help with everyday housework?

		FREQ	WTD
1	Every day	121	19,402
2	At least once a week	307	60,204
3	Less than once a week	229	47,755
93	Not applicable	19,114	3,131,395
98	Not stated	938	161,477
R	Refusal	0	0
X	Don't know	1	104
		======	=========
		20,710	3,420,337

Coverage: Respondents (DISAB=1) who answered C7E=1
Source: Participation and Activity Limitation Survey, 2001

Variable Name:	**C8**	*Position:* 293	*Length:* 2
Collection Name:	AC8		

Do you (Does.....) need ADDITIONAL help with everyday housework?

		FREQ	WTD
1	Yes	1,041	180,529
3	No	5,262	895,208
93	Not applicable	13,623	2,212,737
98	Not stated	771	131,105
R	Refusal	0	0
X	Don't know	13	759
		======	=========
		20,710	3,420,338

Coverage: Respondents (DISAB=1) who answered C5=1
Source: Participation and Activity Limitation Survey, 2001

Variable Name:	**C9**	*Position:* 295	*Length:* 2
Collection Name:	AC9		

Because of your (his / her) condition, do you (does.....) usually RECEIVE help with heavy household chores, such as spring cleaning or yard work?

		FREQ	WTD
1	Yes	10,029	1,711,601
3	No	9,997	1,582,072
93	Not applicable	0	0
98	Not stated	589	105,008
R	Refusal	5	846
X	Don't know	90	20,810
		======	=========
		20,710	3,420,337

Coverage: All respondents (DISAB=1)
Source: Participation and Activity Limitation Survey, 2001

Variable Name: **C10** *Position:* 297 *Length:* 2
Collection Name: AC10

Do you think you NEED (Does..... think he / she NEEDS) help with heavy household chores?

		FREQ	WTD
1	Yes	1,073	200,611
3	No	8,774	1,346,212
93	Not applicable	10,124	1,733,258
98	Not stated	712	134,442
R	Refusal	1	312
X	Don't know	26	5,504
		======	=========
		20,710	3,420,339

Coverage: Respondents (DISAB=1) who answered C9=3
Source: Participation and Activity Limitation Survey, 2001

Variable Name: **C11A** *Position:* 299 *Length:* 2
Collection Name: AC11A

WHO usually helps you (.....) with heavy household chores? I will read you a list. Please answer yes or no to each: Family living with you (him / her)?

		FREQ	WTD
1	Yes	5,786	959,550
3	No	3,970	703,055
93	Not applicable	10,092	1,603,729
98	Not stated	856	152,366
R	Refusal	1	19
X	Don't know	5	1,619
		======	=========
		20,710	3,420,338

Coverage: Respondents (DISAB=1) who answered C9=1
Source: Participation and Activity Limitation Survey, 2001

Variable Name: **C11AA** *Position:* 301 *Length:* 2
Collection Name: AC11AA

HOW OFTEN do they help with heavy household chores?

		FREQ	WTD
1	At least once a week	3,753	622,379
2	At least once a month	1,292	218,797
3	Less than once a month	633	98,716
93	Not applicable	14,068	2,308,421
98	Not stated	922	163,454
R	Refusal	1	59
X	Don't know	41	8,511
		======	=========
		20,710	3,420,337

Coverage: Respondents (DISAB=1) who answered C11A=1
Source: Participation and Activity Limitation Survey, 2001

Variable Name: **C11B** *Position:* 303 *Length:* 2
Collection Name: AC11B

WHO usually helps you (.....) with heavy household chores? I will read you a list. Please answer yes or no to each: Family not living with you (him / her)

		FREQ	WTD
1	Yes	3,270	560,326
3	No	6,384	1,079,453
93	Not applicable	10,092	1,603,729
98	Not stated	956	174,610
R	Refusal	1	19
X	Don't know	7	2,201
		======	=========
		20,710	3,420,338

Coverage: Respondents (DISAB=1) who answered C9=1
Source: Participation and Activity Limitation Survey, 2001

Variable Name:	**C11AB**	*Position:*	305	*Length:*	2
Collection Name:	AC11AB				

HOW OFTEN do they help with heavy household chores?

		FREQ	WTD
1	At least once a week	830	140,551
2	At least once a month	1,205	196,554
3	Less than once a month	1,157	208,530
93	Not applicable	16,484	2,685,402
98	Not stated	1,003	183,725
R	Refusal	0	0
X	Don't know	31	5,576
		======	=========
		20,710	3,420,338

Coverage: Respondents (DISAB=1) who answered C11B=1
Source: Participation and Activity Limitation Survey, 2001

Variable Name:	**C11C**	*Position:*	307	*Length:*	2
Collection Name:	AC11C				

WHO usually helps you (.....) with heavy household chores? I will read you a list. Please answer yes or no to each: Friends or neighbours?

		FREQ	WTD
1	Yes	1,999	325,991
3	No	7,628	1,317,297
93	Not applicable	10,092	1,603,729
98	Not stated	982	172,073
R	Refusal	0	0
X	Don't know	9	1,249
		======	=========
		20,710	3,420,339

Coverage: Respondents (DISAB=1) who answered C9=1
Source: Participation and Activity Limitation Survey, 2001

Variable Name:	**C11AC**	*Position:*	309	*Length:*	2
Collection Name:	AC11AC				

HOW OFTEN do they help with heavy household chores?

		FREQ	WTD
1	At least once a week	464	71,022
2	At least once a month	686	98,823
3	Less than once a month	793	146,357
93	Not applicable	17,729	2,922,274
98	Not stated	1,019	179,306
R	Refusal	1	59
X	Don't know	18	2,498
		======	=========
		20,710	3,420,339

Coverage: Respondents (DISAB=1) who answered C11C=1
Source: Participation and Activity Limitation Survey, 2001

Variable Name:	**C11D**	*Position:*	311	*Length:*	2
Collection Name:	AC11D				

WHO usually helps you (.....) with heavy household chores? I will read you a list. Please answer yes or no to each: Organization or agency (Include voluntary, private and government agencies)?

		FREQ	WTD
1	Yes	1,320	238,047
3	No	8,312	1,403,090
93	Not applicable	10,092	1,603,729
98	Not stated	977	172,851
R	Refusal	0	0
X	Don't know	9	2,621
		======	=========
		20,710	3,420,338

Coverage: Respondents (DISAB=1) who answered C9=1
Source: Participation and Activity Limitation Survey, 2001

Variable Name: **C11AD** *Position:* 313 *Length:* 2
Collection Name: AC11AD

HOW OFTEN do they help with heavy household chores?

		FREQ	WTD
1	At least once a week	434	81,919
2	At least once a month	429	69,780
3	Less than once a month	396	72,768
93	Not applicable	18,413	3,009,439
98	Not stated	1,023	184,620
R	Refusal	0	0
X	Don't know	15	1,811
		======	=========
		20,710	3,420,337

Coverage: Respondents (DISAB=1) who answered C11D=1
Source: Participation and Activity Limitation Survey, 2001

Variable Name: **C11E** *Position:* 315 *Length:* 2
Collection Name: AC11E

WHO usually helps you (.....) with heavy household chores? I will read you a list. Please answer yes or no to each: Other help?

		FREQ	WTD
1	Yes	1,217	213,984
3	No	8,402	1,429,001
93	Not applicable	10,092	1,603,729
98	Not stated	990	171,774
R	Refusal	0	0
X	Don't know	9	1,850
		======	=========
		20,710	3,420,338

Coverage: Respondents (DISAB=1) who answered C9=1
Source: Participation and Activity Limitation Survey, 2001

Variable Name:	**C11AE**	*Position:*	317	*Length:*	2
Collection Name:	AC11AE				

HOW OFTEN do they help with heavy household chores?

		FREQ	WTD
1	At least once a week	368	68,258
2	At least once a month	378	65,626
3	Less than once a month	378	63,269
93	Not applicable	18,503	3,034,580
98	Not stated	1,068	185,780
R	Refusal	0	0
X	Don't know	15	2,824
		======	=========
		20,710	3,420,337

Coverage: Respondents (DISAB=1) who answered C11E=1
Source: Participation and Activity Limitation Survey, 2001

Variable Name:	**C12**	*Position:*	319	*Length:*	2
Collection Name:	AC12				

Do you (Does.....) need ADDITIONAL help with heavy household chores?

		FREQ	WTD
1	Yes	1,720	283,502
3	No	8,147	1,395,916
93	Not applicable	10,092	1,603,729
98	Not stated	719	128,934
R	Refusal	0	0
X	Don't know	32	8,257
		======	=========
		20,710	3,420,338

Coverage: Respondents (DISAB=1) who answered C9=1
Source: Participation and Activity Limitation Survey, 2001

Variable Name: **C13** *Position:* 321 *Length:* 2
Collection Name: AC13

Because of your (his / her) condition, do you (does.....) usually RECEIVE help with getting to appointments and running errands, such as shopping for groceries?

		FREQ	WTD
1	Yes	7,109	1,199,439
3	No	12,989	2,118,983
93	Not applicable	0	0
98	Not stated	588	97,688
R	Refusal	1	312
X	Don't know	23	3,916
		======	=========
		20,710	3,420,338

Coverage: All respondents (DISAB=1)
Source: Participation and Activity Limitation Survey, 2001

Variable Name: **C14** *Position:* 323 *Length:* 2
Collection Name: AC14

Do you think you NEED (Does..... think he / she NEEDS) help with getting to appointments and running errands, such as shopping for groceries?

		FREQ	WTD
1	Yes	418	64,460
3	No	12,504	2,042,575
93	Not applicable	7,133	1,203,666
98	Not stated	644	108,524
R	Refusal	0	0
X	Don't know	11	1,112
		======	=========
		20,710	3,420,337

Coverage: Respondents (DISAB=1) who answered C13=3
Source: Participation and Activity Limitation Survey, 2001

Variable Name:	**C15A**	*Position:*	325	*Length:*	2
Collection Name:	AC15A				

WHO usually helps you (.....) with getting to appointments and running errands; I will read you a list. Please answer yes or no to each. Family living with you (him / her)?

		FREQ	WTD
1	Yes	4,373	722,728
3	No	2,426	423,250
93	Not applicable	13,013	2,123,211
98	Not stated	890	149,492
R	Refusal	0	0
X	Don't know	8	1,658
		======	=========
		20,710	3,420,339

Coverage: Respondents (DISAB=1) who answered C13=1
Source: Participation and Activity Limitation Survey, 2001

Variable Name:	**C15AA**	*Position:*	327	*Length:*	2
Collection Name:	AC15AA				

HOW OFTEN do they help with getting to appointments and running errands?

		FREQ	WTD
1	Every day	1,084	166,658
2	At least once a week	2,292	400,503
3	Less than once a week	913	139,494
93	Not applicable	15,447	2,548,118
98	Not stated	959	161,526
R	Refusal	1	167
X	Don't know	14	3,872
		======	=========
		20,710	3,420,338

Coverage: Respondents (DISAB=1) who answered C15A=1
Source: Participation and Activity Limitation Survey, 2001

Variable Name:	**C15B**	*Position:*	329	*Length:*	2
Collection Name:	AC15B				

WHO usually helps you (.....) with getting to appointments and running errands; I will read you a list. Please answer yes or no to each. Family not living with you (him / her)?

		FREQ	WTD
1	Yes	2,701	465,209
3	No	4,040	670,328
93	Not applicable	13,013	2,123,211
98	Not stated	941	159,253
R	Refusal	0	0
X	Don't know	15	2,338
		======	=========
		20,710	3,420,339

Coverage: Respondents (DISAB=1) who answered C13=1
Source: Participation and Activity Limitation Survey, 2001

Variable Name:	**C15AB**	*Position:*	331	*Length:*	2
Collection Name:	AC15AB				

HOW OFTEN do they help with getting to appointments and running errands?

		FREQ	WTD
1	Every day	136	20,621
2	At least once a week	1,333	239,246
3	Less than once a week	1,173	195,888
93	Not applicable	17,068	2,795,876
98	Not stated	980	163,777
R	Refusal	0	0
X	Don't know	20	4,929
		======	=========
		20,710	3,420,337

Coverage: Respondents (DISAB=1) who answered C15B=1
Source: Participation and Activity Limitation Survey, 2001

Variable Name:	**C15C**	*Position:*	333	*Length:*	2
Collection Name:	AC15C				

WHO usually helps you (.....) with getting to appointments and running errands; I will read you a list. Please answer yes or no to each: Friends or neighbours?

		FREQ	WTD
1	Yes	1,364	218,656
3	No	5,336	909,147
93	Not applicable	13,013	2,123,211
98	Not stated	977	166,237
R	Refusal	0	0
X	Don't know	20	3,086
		======	=========
		20,710	3,420,337

Coverage: Respondents (DISAB=1) who answered C13=1
Source: Participation and Activity Limitation Survey, 2001

Variable Name:	**C15AC**	*Position:*	335	*Length:*	2
Collection Name:	AC15AC				

HOW OFTEN do they help with getting to appointments and running errands?

		FREQ	WTD
1	Every day	81	16,573
2	At least once a week	586	87,666
3	Less than once a week	664	108,808
93	Not applicable	18,369	3,035,444
98	Not stated	996	170,099
R	Refusal	0	0
X	Don't know	14	1,749
		======	=========
		20,710	3,420,339

Coverage: Respondents (DISAB=1) who answered C15C=1
Source: Participation and Activity Limitation Survey, 2001

Variable Name: **C15D** *Position:* 337 *Length:* 2
Collection Name: AC15D

WHO usually helps you (.....) with getting to appointments and running errands; I will read you a list. Please answer yes or no to each: Organization or agency (Include voluntary, private and government agencies)?

		FREQ	WTD
1	Yes	687	109,367
3	No	6,013	1,019,197
93	Not applicable	13,013	2,123,211
98	Not stated	971	164,611
R	Refusal	0	0
X	Don't know	26	3,952
		======	=========
		20,710	3,420,338

Coverage: Respondents (DISAB=1) who answered C13=1
Source: Participation and Activity Limitation Survey, 2001

Variable Name: **C15AD** *Position:* 339 *Length:* 2
Collection Name: AC15AD

HOW OFTEN do they help with getting to appointments and running errands?

		FREQ	WTD
1	Every day	111	14,135
2	At least once a week	318	56,424
3	Less than once a week	223	34,651
93	Not applicable	19,052	3,146,359
98	Not stated	995	168,142
R	Refusal	0	0
X	Don't know	11	627
		======	=========
		20,710	3,420,338

Coverage: Respondents (DISAB=1) who answered C15D=1
Source: Participation and Activity Limitation Survey, 2001

Variable Name: **C15E** *Position:* 341 *Length:* 2
Collection Name: AC15E

WHO usually helps you (.....) with getting to appointments and running errands; I will read you a list. Please answer yes or no to each: other help?

		FREQ	WTD
1	Yes	295	49,148
3	No	6,361	1,073,802
93	Not applicable	13,013	2,123,211
98	Not stated	1,015	170,209
R	Refusal	0	0
X	Don't know	26	3,968
		======	=========
		20,710	3,420,338

Coverage: Respondents (DISAB=1) who answered C13=1
Source: Participation and Activity Limitation Survey, 2001

Variable Name: **C15AE** *Position:* 343 *Length:* 2
Collection Name: AC15AE

HOW OFTEN do they help with getting to appointments and running errands?

		FREQ	WTD
1	Every day	45	7,083
2	At least once a week	145	23,307
3	Less than once a week	90	16,578
93	Not applicable	19,400	3,200,981
98	Not stated	1,027	171,977
R	Refusal	0	0
X	Don't know	3	412
		======	=========
		20,710	3,420,338

Coverage: Respondents (DISAB=1) who answered C15E=1
Source: Participation and Activity Limitation Survey, 2001

Variable Name: **C16** *Position:* 345 *Length:* 2
Collection Name: AC16

Do you (Does.....) need ADDITIONAL help with getting to appointments and running errands?

		FREQ	WTD
1	Yes	1,074	187,920
3	No	5,891	988,789
93	Not applicable	13,013	2,123,211
98	Not stated	710	116,539
R	Refusal	0	0
X	Don't know	22	3,879
		======	=========
		20,710	3,420,338

Coverage: Respondents (DISAB=1) who answered C13=1
Source: Participation and Activity Limitation Survey, 2001

Variable Name: **C17** *Position:* 347 *Length:* 2
Collection Name: AC17

Because of your (his / her) condition, do you (does.....) usually RECEIVE help with looking after your (his / her) personal finances, such as making bank transactions or paying bills?

		FREQ	WTD
1	Yes	3,747	622,556
3	No	16,308	2,687,447
93	Not applicable	0	0
98	Not stated	627	108,296
R	Refusal	4	452
X	Don't know	24	1,586
		======	=========
		20,710	3,420,337

Coverage: All respondents (DISAB=1)
Source: Participation and Activity Limitation Survey, 2001

Variable Name: **C18** *Position:* 349 *Length:* 2
Collection Name: AC18

Do you think you NEED (Does..... think he / she NEEDS) help with looking after your (his / her) personal finances?

		FREQ	WTD
1	Yes	191	31,855
3	No	16,020	2,631,740
93	Not applicable	3,775	624,595
98	Not stated	717	130,652
R	Refusal	0	0
X	Don't know	7	1,495
		======	=========
		20,710	3,420,337

Coverage: Respondents (DISAB=1) who answered C17=3
Source: Participation and Activity Limitation Survey, 2001

Variable Name: **C19A** *Position:* 351 *Length:* 2
Collection Name: AC19A

WHO usually helps you (.....) with looking after your (his / her) personal finances, I will read you a list. Please answer yes or no to each: Family living with you (him / her)?

		FREQ	WTD
1	Yes	2,498	414,104
3	No	1,052	169,694
93	Not applicable	16,336	2,689,485
98	Not stated	821	145,656
R	Refusal	0	0
X	Don't know	3	1,400
		======	=========
		20,710	3,420,339

Coverage: Respondents (DISAB=1) who answered C17=1
Source: Participation and Activity Limitation Survey, 2001

Variable Name: **C19AA** *Position:* 353 *Length:* 2
Collection Name: AC19AA

HOW OFTEN do they help with looking after your (his / her) personal finances?

		FREQ	WTD
1	At least once a week	1,193	200,735
2	At least once a month	1,101	179,944
3	Less than once a month	141	20,852
93	Not applicable	17,391	2,860,578
98	Not stated	868	153,016
R	Refusal	0	0
X	Don't know	16	5,213
		======	=========
		20,710	3,420,338

Coverage: Respondents (DISAB=1) who answered C19A=1
Source: Participation and Activity Limitation Survey, 2001

Variable Name: **C19B** *Position:* 355 *Length:* 2
Collection Name: AC19B

WHO usually helps you (.....) with looking after your (his / her) personal finances, I will read you a list. Please answer yes or no to each: Family not living with you (him / her)?

		FREQ	WTD
1	Yes	1,042	180,917
3	No	2,455	396,525
93	Not applicable	16,336	2,689,485
98	Not stated	875	153,354
R	Refusal	0	0
X	Don't know	2	56
		======	=========
		20,710	3,420,337

Coverage: Respondents (DISAB=1) who answered C17=1
Source: Participation and Activity Limitation Survey, 2001

Variable Name:	**C19AB**	*Position:*	357	*Length:*	2
Collection Name:	AC19AB				

HOW OFTEN do they help with looking after your (his / her) personal finances?

		FREQ	WTD
1	At least once a week	330	61,170
2	At least once a month	522	86,224
3	Less than once a month	145	24,871
93	Not applicable	18,793	3,086,067
98	Not stated	906	158,602
R	Refusal	0	0
X	Don't know	14	3,404
		======	=========
		20,710	3,420,338

Coverage: Respondents (DISAB=1) who answered C19B=1
Source: Participation and Activity Limitation Survey, 2001

Variable Name:	**C19C**	*Position:*	359	*Length:*	2
Collection Name:	AC19C				

WHO usually helps you (.....) with looking after your (his / her) personal finances, I will read you a list. Please answer yes or no to each: Friends or neighbours?

		FREQ	WTD
1	Yes	151	25,893
3	No	3,321	548,013
93	Not applicable	16,336	2,689,485
98	Not stated	898	155,522
R	Refusal	0	0
X	Don't know	4	1,425
		======	=========
		20,710	3,420,338

Coverage: Respondents (DISAB=1) who answered C17=1
Source: Participation and Activity Limitation Survey, 2001

Variable Name:	**C19AC**	*Position:*	361	*Length:*	2
Collection Name:	AC19AC				

HOW OFTEN do they help with looking after your (his / her) personal finances?

		FREQ	WTD
1	At least once a week	35	5,127
2	At least once a month	79	14,071
3	Less than once a month	33	5,565
93	Not applicable	19,661	3,238,923
98	Not stated	900	155,960
R	Refusal	0	0
X	Don't know	2	692
		======	=========
		20,710	3,420,338

Coverage: Respondents (DISAB=1) who answered C19C=1
Source: Participation and Activity Limitation Survey, 2001

Variable Name:	**C19D**	*Position:*	363	*Length:*	2
Collection Name:	AC19D				

WHO usually helps you (.....) with looking after your (his / her) personal finances, I will read you a list. Please answer yes or no to each: Organization or agency (Include voluntary, private and government agencies)?

		FREQ	WTD
1	Yes	209	26,001
3	No	3,276	548,168
93	Not applicable	16,336	2,689,485
98	Not stated	885	155,249
R	Refusal	0	0
X	Don't know	4	1,435
		======	=========
		20,710	3,420,338

Coverage: Respondents (DISAB=1) who answered C17=1
Source: Participation and Activity Limitation Survey, 2001

Variable Name:	**C19AD**	*Position:*	365	*Length:*	2
Collection Name:	AC19AD				

HOW OFTEN do they help with looking after your (his / her) personal finances?

		FREQ	WTD
1	At least once a week	76	7,900
2	At least once a month	87	12,410
3	Less than once a month	34	4,521
93	Not applicable	19,616	3,239,089
98	Not stated	896	156,376
R	Refusal	0	0
X	Don't know	1	42
		======	=========
		20,710	3,420,338

Coverage: Respondents (DISAB=1) who answered C19D=1
Source: Participation and Activity Limitation Survey, 2001

Variable Name:	**C19E**	*Position:*	367	*Length:*	2
Collection Name:	AC19E				

WHO usually helps you (.....) with looking after your (his / her) personal finances, I will read you a list. Please answer yes or no to each: Other?

		FREQ	WTD
1	Yes	100	13,190
3	No	3,371	559,150
93	Not applicable	16,336	2,689,485
98	Not stated	901	157,146
R	Refusal	0	0
X	Don't know	2	1,366
		======	=========
		20,710	3,420,337

Coverage: Respondents (DISAB=1) who answered C17=1
Source: Participation and Activity Limitation Survey, 2001

Variable Name: **C19AE** *Position:* 369 *Length:* 2
Collection Name: AC19AE

HOW OFTEN do they help with looking after your (his / her) personal finances?

		FREQ	WTD
1	At least once a week	20	2,213
2	At least once a month	47	5,590
3	Less than once a month	25	4,160
93	Not applicable	19,709	3,250,002
98	Not stated	907	158,332
R	Refusal	0	0
X	Don't know	2	41
		======	=========
		20,710	3,420,338

Coverage: Respondents (DISAB=1) who answered C19E=1
Source: Participation and Activity Limitation Survey, 2001

Variable Name: **C20** *Position:* 371 *Length:* 2
Collection Name: AC20

Do you (Does.....) need ADDITIONAL help with looking after your (his / her) personal finances?

		FREQ	WTD
1	Yes	346	49,413
3	No	3,318	553,376
93	Not applicable	16,336	2,689,485
98	Not stated	704	127,723
R	Refusal	0	0
X	Don't know	6	340
		======	=========
		20,710	3,420,337

Coverage: Respondents (DISAB=1) who answered C17=1
Source: Participation and Activity Limitation Survey, 2001

Variable Name:	**C22**	*Position:*	373	*Length:*	2
Collection Name:	AC22				

Because of your (his / her) condition, do you (does.....) usually RECEIVE help with child care?

		FREQ	WTD
1	Yes	422	45,862
3	No	2,939	389,179
93	Not applicable	16,685	2,873,644
98	Not stated	654	110,385
R	Refusal	2	44
X	Don't know	8	1,223
		======	=========
		20,710	3,420,337

Coverage: Respondents (DISAB=1) who answered C21=1
Source: Participation and Activity Limitation Survey, 2001

Variable Name:	**C23**	*Position:*	375	*Length:*	2
Collection Name:	AC23				

Do you think you NEED (Does..... think he / she NEEDS) help with child care?

		FREQ	WTD
1	Yes	133	16,258
3	No	2,781	368,206
93	Not applicable	17,117	2,920,773
98	Not stated	675	114,551
R	Refusal	0	0
X	Don't know	4	551
		======	=========
		20,710	3,420,339

Coverage: Respondents (DISAB=1) who answered C22=3
Source: Participation and Activity Limitation Survey, 2001

Variable Name: **C24A** *Position:* 377 *Length:* 2
Collection Name: AC24A

WHO usually helps you (.....) with child care? I will read you a list. Please answer yes or no to each: Family living with you (him / her)?

		FREQ	WTD
1	Yes	267	25,929
3	No	137	16,589
93	Not applicable	19,634	3,264,091
98	Not stated	672	113,729
R	Refusal	0	0
X	Don't know	0	0
		======	=========
		20,710	3,420,338

Coverage: Respondents (DISAB=1) who answered C22=1
Source: Participation and Activity Limitation Survey, 2001

Variable Name: **C24AA** *Position:* 379 *Length:* 2
Collection Name: AC24AA

HOW OFTEN do they help with child care?

		FREQ	WTD
1	Every day	204	21,440
2	At least once a week	44	3,373
3	Less than once a week	17	1,062
93	Not applicable	19,771	3,280,680
98	Not stated	674	113,783
R	Refusal	0	0
X	Don't know	0	0
		======	=========
		20,710	3,420,338

Coverage: Respondents (DISAB=1) who answered C24A=1
Source: Participation and Activity Limitation Survey, 2001

Variable Name: **C24B** *Position:* 381 *Length:* 2
Collection Name: AC24B

WHO usually helps you (.....) with child care? I will read you a list. Please answer yes or no to each: Family not living with you (him / her)?

		FREQ	WTD
1	Yes	197	19,739
3	No	202	23,543
93	Not applicable	19,634	3,264,091
98	Not stated	677	112,965
R	Refusal	0	0
X	Don't know	0	0
		======	=========
		20,710	3,420,338

Coverage: Respondents (DISAB=1) who answered C22=1
Source: Participation and Activity Limitation Survey, 2001

Variable Name: **C24AB** *Position:* 383 *Length:* 2
Collection Name: AC24AB

HOW OFTEN do they help with child care?

		FREQ	WTD
1	Every day	31	3,356
2	At least once a week	94	10,076
3	Less than once a week	69	5,758
93	Not applicable	19,836	3,287,633
98	Not stated	680	113,515
R	Refusal	0	0
X	Don't know	0	0
		======	=========
		20,710	3,420,338

Coverage: Respondents (DISAB=1) who answered C24B=1
Source: Participation and Activity Limitation Survey, 2001

Variable Name: **C24C** *Position:* 385 *Length:* 2
Collection Name: AC24C

WHO usually helps you (.....) with child care? I will read you a list. Please answer yes or no to each: Friends or neighbours?

		FREQ	WTD
1	Yes	99	9,684
3	No	296	31,902
93	Not applicable	19,634	3,264,091
98	Not stated	680	114,550
R	Refusal	0	0
X	Don't know	1	111
		======	=========
		20,710	3,420,338

Coverage: Respondents (DISAB=1) who answered C22=1
Source: Participation and Activity Limitation Survey, 2001

Variable Name: **C24AC** *Position:* 387 *Length:* 2
Collection Name: AC24AC

HOW OFTEN do they help with child care?

		FREQ	WTD
1	Every day	9	848
2	At least once a week	45	5,771
3	Less than once a week	43	3,009
93	Not applicable	19,931	3,296,104
98	Not stated	682	114,605
R	Refusal	0	0
X	Don't know	0	0
		======	=========
		20,710	3,420,337

Coverage: Respondents (DISAB=1) who answered C24C=1
Source: Participation and Activity Limitation Survey, 2001

Variable Name:	**C24D**	*Position:*	389	*Length:*	2
Collection Name:	AC24D				

WHO usually helps you (.....) with child care? I will read you a list. Please answer yes or no to each: Organization or agency (Include voluntary, private and government agencies)?

		FREQ	WTD
1	Yes	72	7,105
3	No	321	34,780
93	Not applicable	19,634	3,264,091
98	Not stated	682	114,250
R	Refusal	0	0
X	Don't know	1	111
		======	=========
		20,710	3,420,337

Coverage: Respondents (DISAB=1) who answered C22=1
Source: Participation and Activity Limitation Survey, 2001

Variable Name:	**C24AD**	*Position:*	391	*Length:*	2
Collection Name:	AC24AD				

HOW OFTEN do they help with child care?

		FREQ	WTD
1	Every day	28	3,379
2	At least once a week	29	2,695
3	Less than once a week	10	655
93	Not applicable	19,956	3,298,982
98	Not stated	687	114,626
R	Refusal	0	0
X	Don't know	0	0
		======	=========
		20,710	3,420,337

Coverage: Respondents (DISAB=1) who answered C24D=1
Source: Participation and Activity Limitation Survey, 2001

Variable Name:	**C24E**	*Position:*	393	*Length:*	2
Collection Name:	AC24E				

WHO usually helps you (.....) with child care? I will read you a list. Please answer yes or no to each: Other help?

		FREQ	WTD
1	Yes	30	3,128
3	No	358	38,539
93	Not applicable	19,634	3,264,091
98	Not stated	687	114,468
R	Refusal	0	0
X	Don't know	1	111
		======	=========
		20,710	3,420,337

Coverage: Respondents (DISAB=1) who answered C22=1
Source: Participation and Activity Limitation Survey, 2001

Variable Name:	**C24AE**	*Position:*	395	*Length:*	2
Collection Name:	AC24AE				

HOW OFTEN do they help with child care?

		FREQ	WTD
1	Every day	9	1,177
2	At least once a week	13	914
3	Less than once a week	4	703
93	Not applicable	19,993	3,302,741
98	Not stated	691	114,802
R	Refusal	0	0
X	Don't know	0	0
		======	=========
		20,710	3,420,337

Coverage: Respondents (DISAB=1) who answered C24E=1
Source: Participation and Activity Limitation Survey, 2001

Variable Name:	**C25**	*Position:* 397	*Length:* 2
Collection Name:	AC25		

Do you (Does.....) need ADDITIONAL help with child care?

		FREQ	WTD
1	Yes	75	8,851
3	No	344	36,819
93	Not applicable	19,634	3,264,091
98	Not stated	657	110,577
R	Refusal	0	0
X	Don't know	0	0
		======	=========
		20,710	3,420,338

Coverage: Respondents (DISAB=1) who answered C22=1
Source: Participation and Activity Limitation Survey, 2001

Variable Name:	**C26**	*Position:* 399	*Length:* 2
Collection Name:	AC26		

Because of your (his / her) condition, do you (does.....) usually RECEIVE help with personal care, such as washing, dressing or taking medication?

		FREQ	WTD
1	Yes	2,633	422,020
3	No	17,446	2,890,836
93	Not applicable	0	0
98	Not stated	621	105,627
R	Refusal	1	312
X	Don't know	9	1,543
		======	=========
		20,710	3,420,338

Coverage: All respondents (DISAB=1)
Source: Participation and Activity Limitation Survey, 2001

Variable Name:	**C27**	*Position:*	401	*Length:*	2
Collection Name:	AC27				

Do you think you NEED (Does..... think he / she NEEDS) help with personal care?

		FREQ	WTD
1	Yes	190	37,309
3	No	17,146	2,823,094
93	Not applicable	2,643	423,875
98	Not stated	722	132,154
R	Refusal	0	0
X	Don't know	9	3,906
		======	=========
		20,710	3,420,338

Coverage: Respondents (DISAB=1) who answered C26=3
Source: Participation and Activity Limitation Survey, 2001

Variable Name:	**C28A**	*Position:*	403	*Length:*	2
Collection Name:	AC28A				

WHO usually helps you (.....) with personal care? I will read you a list. Please answer yes or no to each: Family living with you (him / her)?

		FREQ	WTD
1	Yes	1,775	273,497
3	No	704	122,953
93	Not applicable	17,456	2,892,691
98	Not stated	770	130,182
R	Refusal	0	0
X	Don't know	5	1,014
		======	=========
		20,710	3,420,337

Coverage: Respondents (DISAB=1) who answered C26=1
Source: Participation and Activity Limitation Survey, 2001

Variable Name:	**C28AA**	*Position:*	405	*Length:*	3
Collection Name:	AC28AA				

On average, how many days a week do family living with you (him / her) help with personal care?

Allowed values: 001 : 007

		FREQ	WTD
-3	Not applicable	18,165	3,016,659
-8	Not stated	819	140,157
001 : 007	# days per week/help with personal care	1,726	263,522
		======	=========
		20,710	3,420,338

Coverage: Respondents (DISAB=1) who answered C28A=1
Source: Participation and Activity Limitation Survey, 2001

Variable Name: **C28B** *Position:* 408 *Length:* 2
Collection Name: AC28B

WHO usually helps you (.....) with personal care? I will read you a list. Please answer yes or no to each: Family not living with you (him / her)?

		FREQ	WTD
1	Yes	296	42,247
3	No	2,105	340,221
93	Not applicable	17,456	2,892,691
98	Not stated	846	140,201
R	Refusal	0	0
X	Don't know	7	4,977
		======	=========
		20,710	3,420,337

Coverage: Respondents (DISAB=1) who answered C26=1
Source: Participation and Activity Limitation Survey, 2001

Variable Name: **C28AB** *Position:* 410 *Length:* 3
Collection Name: AC28AB

On average, how many days a week do family not living with you help with personal care?

Allowed values: 001 : 007

		FREQ	WTD
-3	Not applicable	19,568	3,237,890
-8	Not stated	877	145,697
001 : 007	# days per week/help with personal care	265	36,750
		======	=========
		20,710	3,420,337

Coverage: Respondents (DISAB=1) who answered C28B=1
Source: Participation and Activity Limitation Survey, 2001

Variable Name: **C28C** *Position:* 413 *Length:* 2
Collection Name: AC28C

WHO usually helps you (.....) with personal care? I will read you a list: Please answer yes or no to each: Friends or neighbours?

		FREQ	WTD
1	Yes	105	19,898
3	No	2,298	361,072
93	Not applicable	17,456	2,892,691
98	Not stated	844	141,699
R	Refusal	0	0
X	Don't know	7	4,977
		======	=========
		20,710	3,420,337

Coverage: Respondents (DISAB=1) who answered C26=1
Source: Participation and Activity Limitation Survey, 2001

Variable Name:	**C28AC**	*Position:*	415	*Length:*	3
Collection Name:	AC28AC				

On average, how many days a week do friends or neighbours help with personal care?

Allowed values: 001 : 007

		FREQ	WTD
-3	Not applicable	19,761	3,258,741
-8	Not stated	852	143,292
001 : 007	# days per week/help with personal care	97	18,305
		======	=========
		20,710	3,420,338

Coverage: Respondents (DISAB=1) who answered C28C=1
Source: Participation and Activity Limitation Survey, 2001

Variable Name:	**C28D**	*Position:*	418	*Length:*	2
Collection Name:	AC28D				

WHO usually helps you (.....) with personal care? I will read you a list: Please answer yes or no to each: Organization or agency (Include voluntary, private and government agencies)?

		FREQ	WTD
1	Yes	866	139,926
3	No	1,576	251,840
93	Not applicable	17,456	2,892,691
98	Not stated	805	134,169
R	Refusal	0	0
X	Don't know	7	1,711
		======	=========
		20,710	3,420,337

Coverage: Respondents (DISAB=1) who answered C26=1
Source: Participation and Activity Limitation Survey, 2001

Variable Name:	**C28AD**	*Position:*	420	*Length:*	3
Collection Name:	AC28AD				

On average, how many days a week do organization or agency (Include voluntary, private and government agencies) help with personal care?

Allowed values: 001 : 007

		FREQ	WTD
-3	Not applicable	19,039	3,146,242
-8	Not stated	845	143,397
001 : 007	# days per week/help with personal care	826	130,699
		======	=========
		20,710	3,420,338

Coverage: Respondents (DISAB=1) who answered C28D=1
Source: Participation and Activity Limitation Survey, 2001

Variable Name:	**C28E**	*Position:*	423	*Length:*	2
Collection Name:	AC28E				

WHO usually helps you (.....) with personal care? I will read you a list: Please answer yes or no to each: other?

		FREQ	WTD
1	Yes	147	21,321
3	No	2,229	355,960
93	Not applicable	17,456	2,892,691
98	Not stated	871	145,989
R	Refusal	0	0
X	Don't know	7	4,375
		======	=========
		20,710	3,420,336

Coverage: Respondents (DISAB=1) who answered C26=1
Source: Participation and Activity Limitation Survey, 2001

Variable Name:	**C28AE**	*Position:*	425	*Length:*	3
Collection Name:	AC28AE				

On average, how many days a week do other help with personal care?

Allowed values: 001 : 007

		FREQ	WTD
-3	Not applicable	19,692	3,253,027
-8	Not stated	879	146,519
001 : 007	# days per week/help with personal care	139	20,792
		======	=========
		20,710	3,420,338

Coverage: Respondents (DISAB=1) who answered C28E=1
Source: Participation and Activity Limitation Survey, 2001

Variable Name:	**C29**	*Position:*	428	*Length:*	2
Collection Name:	AC29				

Do you (Does.....) need ADDITIONAL help with personal care?

		FREQ	WTD
1	Yes	397	62,413
3	No	2,143	339,060
93	Not applicable	17,456	2,892,691
98	Not stated	707	125,109
R	Refusal	0	0
X	Don't know	7	1,065
		======	=========
		20,710	3,420,338

Coverage: Respondents (DISAB=1) who answered C26=1
Source: Participation and Activity Limitation Survey, 2001

Variable Name: **C30** *Position:* 430 *Length:* 2
Collection Name: AC30

Because of your (his / her) condition, do you (does.....) usually RECEIVE specialized nursing care or medical treatment at home such as injections, therapy, blood, urine testing or catheter care?

		FREQ	WTD
1	Yes	914	165,982
3	No	19,096	3,131,691
93	Not applicable	0	0
98	Not stated	674	116,479
R	Refusal	3	1,151
X	Don't know	23	5,034
		======	=========
		20,710	3,420,337

Coverage: All respondents (DISAB=1)
Source: Participation and Activity Limitation Survey, 2001

Variable Name: **C31** *Position:* 432 *Length:* 2
Collection Name: AC31

Do you think you NEED (Does..... think he / she NEEDS) specialized nursing care or medical treatment at home?

		FREQ	WTD
1	Yes	210	44,221
3	No	18,744	3,056,792
93	Not applicable	940	172,167
98	Not stated	778	139,969
R	Refusal	1	89
X	Don't know	37	7,100
		======	=========
		20,710	3,420,338

Coverage: Respondents (DISAB=1) who answered C30=3
Source: Participation and Activity Limitation Survey, 2001

Variable Name:	**C32A**	*Position:*	434	*Length:*	2
Collection Name:	AC32A				

WHO usually provides you (.....) specialized nursing care or medical treatment at home? I will read you a list. Please answer yes or no to each: Family living with you (him / her)?

		FREQ	WTD
1	Yes	151	20,954
3	No	710	134,381
93	Not applicable	19,122	3,137,876
98	Not stated	725	127,071
R	Refusal	0	0
X	Don't know	2	56
		======	=========
		20,710	3,420,338

Coverage: Respondents (DISAB=1) who answered C30=1
Source: Participation and Activity Limitation Survey, 2001

Variable Name:	**C32AA**	*Position:*	436	*Length:*	2
Collection Name:	AC32AA				

HOW OFTEN do family living with you (him / her) provide specialized nursing care or medical treatment at home?

		FREQ	WTD
1	Every day	84	12,549
2	At least once a week	41	5,867
3	Less than once a week	17	1,959
93	Not applicable	19,834	3,272,313
98	Not stated	733	127,630
R	Refusal	1	20
X	Don't know	0	0
		======	=========
		20,710	3,420,338

Coverage: Respondents (DISAB=1) who answered C32A=1
Source: Participation and Activity Limitation Survey, 2001

Variable Name: **C32B** *Position:* 438 *Length:* 2
Collection Name: AC32B

WHO usually provides you (.....) specialized nursing care or medical treatment at home? I will read you a list. Please answer yes or no to each: Family not living with you (him / her)?

		FREQ	WTD
1	Yes	44	5,645
3	No	810	149,361
93	Not applicable	19,122	3,137,876
98	Not stated	731	127,233
R	Refusal	0	0
X	Don't know	3	223
		======	=========
		20,710	3,420,338

Coverage: Respondents (DISAB=1) who answered C30=1
Source: Participation and Activity Limitation Survey, 2001

Variable Name: **C32AB** *Position:* 440 *Length:* 2
Collection Name: AC32AB

HOW OFTEN do family not living with you provide specialized nursing care or medical treatment at home?

		FREQ	WTD
1	Every day	10	947
2	At least once a week	14	2,113
3	Less than once a week	18	2,449
93	Not applicable	19,935	3,287,460
98	Not stated	733	127,368
R	Refusal	0	0
X	Don't know	0	0
		======	=========
		20,710	3,420,337

Coverage: Respondents (DISAB=1) who answered C32B=1
Source: Participation and Activity Limitation Survey, 2001

Variable Name:	**C32C**	*Position:* 442	*Length:* 2
Collection Name:	AC32C		

WHO usually provides you (.....) specialized nursing care or medical treatment at home? I will read you a list. Please answer yes or no to each: Friends or neighbours?

		FREQ	WTD
1	Yes	13	1,677
3	No	837	150,728
93	Not applicable	19,122	3,137,876
98	Not stated	736	130,001
R	Refusal	0	0
X	Don't know	2	56
		======	=========
		20,710	3,420,338

Coverage: Respondents (DISAB=1) who answered C30=1
Source: Participation and Activity Limitation Survey, 2001

Variable Name:	**C32AC**	*Position:* 444	*Length:* 2
Collection Name:	AC32AC		

HOW OFTEN do friends or neighbours provide specialized nursing care or medical treatment at home?

		FREQ	WTD
1	Every day	2	354
2	At least once a week	2	165
3	Less than once a week	7	1,022
93	Not applicable	19,961	3,288,660
98	Not stated	738	130,136
R	Refusal	0	0
X	Don't know	0	0
		======	=========
		20,710	3,420,337

Coverage: Respondents (DISAB=1) who answered C32C=1
Source: Participation and Activity Limitation Survey, 2001

Variable Name:	**C32D**	*Position:*	446	*Length:*	2
Collection Name:	AC32D				

WHO usually provides you (.....) specialized nursing care or medical treatment at home? I will read you a list. Please answer yes or no to each: Organization or agency (Include voluntary, private and government agencies)?

		FREQ	WTD
1	Yes	704	126,114
3	No	165	30,999
93	Not applicable	19,122	3,137,876
98	Not stated	718	124,521
R	Refusal	0	0
X	Don't know	1	827
		======	=========
		20,710	3,420,337

Coverage: Respondents (DISAB=1) who answered C30=1
Source: Participation and Activity Limitation Survey, 2001

Variable Name:	**C32AD**	*Position:*	448	*Length:*	2
Collection Name:	AC32AD				

HOW OFTEN do organization or agency (include voluntary, private and government agencies) provide specialized nursing care or medical treatment at home?

		FREQ	WTD
1	Every day	145	23,917
2	At least once a week	228	38,817
3	Less than once a week	300	57,387
93	Not applicable	19,288	3,169,702
98	Not stated	737	126,834
R	Refusal	0	0
X	Don't know	12	3,681
		======	=========
		20,710	3,420,338

Coverage: Respondents (DISAB=1) who answered C32D=1
Source: Participation and Activity Limitation Survey, 2001

Variable Name: **C32E** *Position:* 450 *Length:* 2
Collection Name: AC32E

WHO usually provides you (.....) specialized nursing care or medical treatment at home? I will read you a list. Please answer yes or no to each: Other?

		FREQ	WTD
1	Yes	115	26,410
3	No	725	125,960
93	Not applicable	19,122	3,137,876
98	Not stated	746	130,035
R	Refusal	0	0
X	Don't know	2	56
		======	=========
		20,710	3,420,337

Coverage: Respondents (DISAB=1) who answered C30=1
Source: Participation and Activity Limitation Survey, 2001

Variable Name: **C32AE** *Position:* 452 *Length:* 2
Collection Name: AC32AE

HOW OFTEN do other provide specialized nursing care or medical treatment at home?

		FREQ	WTD
1	Every day	24	7,420
2	At least once a week	20	4,435
3	Less than once a week	67	13,270
93	Not applicable	19,849	3,263,892
98	Not stated	749	131,298
R	Refusal	0	0
X	Don't know	1	23
		======	=========
		20,710	3,420,338

Coverage: Respondents (DISAB=1) who answered C32E=1
Source: Participation and Activity Limitation Survey, 2001

Variable Name:	**C33**	*Position:*	454	*Length:*	2
Collection Name:	AC33				

Do you (Does.....) need ADDITIONAL specialized nursing care or medical treatment at home?

		FREQ	WTD
1	Yes	94	16,446
3	No	803	145,832
93	Not applicable	19,122	3,137,876
98	Not stated	688	118,828
R	Refusal	0	0
X	Don't know	3	1,356
		======	=========
		20,710	3,420,338

Coverage: Respondents (DISAB=1) who answered C30=1
Source: Participation and Activity Limitation Survey, 2001

Variable Name:	**C34**	*Position:*	456	*Length:*	2
Collection Name:	AC34				

Because of your (his / her) condition, do you (does.....) usually RECEIVE help with moving about inside your (his / her) residence?

		FREQ	WTD
1	Yes	929	145,491
3	No	19,076	3,152,165
93	Not applicable	0	0
98	Not stated	697	121,007
R	Refusal	1	312
X	Don't know	7	1,363
		======	=========
		20,710	3,420,338

Coverage: All respondents (DISAB=1)
Source: Participation and Activity Limitation Survey, 2001

Variable Name: **C35** *Position:* 458 *Length:* 2
Collection Name: AC35

Do you think you NEED (Does..... think he / she NEEDS) help with moving about inside your (his / her) residence?

		FREQ	WTD
1	Yes	89	17,886
3	No	18,717	3,092,561
93	Not applicable	937	147,166
98	Not stated	954	160,323
R	Refusal	1	458
X	Don't know	12	1,944
		======	=========
		20,710	3,420,338

Coverage: Respondents (DISAB=1) who answered C34=3
Source: Participation and Activity Limitation Survey, 2001

Variable Name: **C36A** *Position:* 460 *Length:* 2
Collection Name: AC36A

WHO usually helps you (.....) with moving about inside your (his / her) residence? I will read you a list. Please answer yes or no to each: Family living with you (him / her)?

		FREQ	WTD
1	Yes	719	113,937
3	No	162	24,559
93	Not applicable	19,084	3,153,840
98	Not stated	744	127,970
R	Refusal	0	0
X	Don't know	1	32
		======	=========
		20,710	3,420,338

Coverage: Respondents (DISAB=1) who answered C34=1
Source: Participation and Activity Limitation Survey, 2001

Variable Name: **C36B** *Position:* 462 *Length:* 2
Collection Name: AC36B

WHO usually helps you (.....) with moving about inside your (his / her) residence? I will read you a list. Please answer yes or no to each: Family not living with you (him / her)?

		FREQ	WTD
1	Yes	189	26,241
3	No	667	107,391
93	Not applicable	19,084	3,153,840
98	Not stated	770	132,866
R	Refusal	0	0
X	Don't know	0	0
		======	=========
		20,710	3,420,338

Coverage: Respondents (DISAB=1) who answered C34=1
Source: Participation and Activity Limitation Survey, 2001

Variable Name: **C36C** *Position:* 464 *Length:* 2
Collection Name: AC36C

WHO usually helps you (.....) with moving about inside your (his / her) residence? I will read you a list. Please answer yes or no to each: Friends or neighbours?

		FREQ	WTD
1	Yes	119	16,032
3	No	735	117,684
93	Not applicable	19,084	3,153,840
98	Not stated	772	132,782
R	Refusal	0	0
X	Don't know	0	0
		======	=========
		20,710	3,420,338

Coverage: Respondents (DISAB=1) who answered C34=1
Source: Participation and Activity Limitation Survey, 2001

Variable Name: **C36D** *Position:* 466 *Length:* 2
Collection Name: AC36D

WHO usually helps you (.....) with moving about inside your (his / her) residence? I will read you a list. Please answer yes or no to each: Organization or agency (Include voluntary, private and government agencies)?

		FREQ	WTD
1	Yes	197	27,940
3	No	661	105,992
93	Not applicable	19,084	3,153,840
98	Not stated	767	132,263
R	Refusal	0	0
X	Don't know	1	303
		======	=========
		20,710	3,420,338

Coverage: Respondents (DISAB=1) who answered C34=1
Source: Participation and Activity Limitation Survey, 2001

Variable Name: **C36E** *Position:* 468 *Length:* 2
Collection Name: AC36E

WHO usually helps you (.....) with moving about inside your (his / her) residence? I will read you a list. Please answer yes or no to each: Other?

		FREQ	WTD
1	Yes	62	8,403
3	No	791	124,220
93	Not applicable	19,084	3,153,840
98	Not stated	773	133,876
R	Refusal	0	0
X	Don't know	0	0
		======	=========
		20,710	3,420,339

Coverage: Respondents (DISAB=1) who answered C34=1
Source: Participation and Activity Limitation Survey, 2001

Variable Name:	**C37**	*Position:*	470	*Length:*	2
Collection Name:	AC37				

Do you (Does.....) need ADDITIONAL help with moving about inside your (his / her) residence?

		FREQ	WTD
1	Yes	135	17,900
3	No	770	124,920
93	Not applicable	19,084	3,153,840
98	Not stated	716	123,399
R	Refusal	0	0
X	Don't know	5	279
		======	=========
		20,710	3,420,338

Coverage: Respondents (DISAB=1) who answered C34=1
Source: Participation and Activity Limitation Survey, 2001

Variable Name:	**C38**	*Position:*	472	*Length:*	2
Collection Name:	AC38				

Was it difficult to make the arrangements for the help you receive (..... receives)?

		FREQ	WTD
1	Yes	1,368	214,384
3	No	11,283	1,922,562
93	Not applicable	6,596	1,029,114
98	Not stated	1,432	249,595
R	Refusal	2	257
X	Don't know	29	4,426
		======	=========
		20,710	3,420,338

Coverage: Respondents (DISAB=1) who reported receiving help with everyday activities (RECHELP=1)
Source: Participation and Activity Limitation Survey, 2001

Variable Name: **C39A** *Position:* 474 *Length:* 2
Collection Name: AC39A

What were the difficulties? I will read you a list. Please answer yes or no to each: Finding qualified help?

		FREQ	WTD
1	Yes	616	79,995
3	No	654	112,888
93	Not applicable	17,910	2,956,359
98	Not stated	1,522	270,444
R	Refusal	0	0
X	Don't know	8	652
		======	=========
		20,710	3,420,338

Coverage: Respondents (DISAB=1) who answered C38=1
Source: Participation and Activity Limitation Survey, 2001

Variable Name: **C39B** *Position:* 476 *Length:* 2
Collection Name: AC39B

What were the difficulties? I will read you a list. Please answer yes or no to each: Delay in obtaining assistance?

		FREQ	WTD
1	Yes	691	102,898
3	No	572	88,550
93	Not applicable	17,910	2,956,359
98	Not stated	1,527	270,673
R	Refusal	0	0
X	Don't know	10	1,858
		======	=========
		20,710	3,420,338

Coverage: Respondents (DISAB=1) who answered C38=1
Source: Participation and Activity Limitation Survey, 2001

Variable Name: **C39C** *Position:* 478 *Length:* 2
Collection Name: AC39C

What were the difficulties? I will read you a list. Please answer yes or no to each: Did not know where to look for help?

		FREQ	WTD
1	Yes	424	54,516
3	No	836	135,476
93	Not applicable	17,910	2,956,359
98	Not stated	1,532	272,759
R	Refusal	0	0
X	Don't know	8	1,228
		======	=========
		20,710	3,420,338

Coverage: Respondents (DISAB=1) who answered C38=1
Source: Participation and Activity Limitation Survey, 2001

Variable Name: **C39D** *Position:* 480 *Length:* 2
Collection Name: AC39D

What were the difficulties? I will read you a list. Please answer yes or no to each: Too expensive?

		FREQ	WTD
1	Yes	681	101,160
3	No	583	90,357
93	Not applicable	17,910	2,956,359
98	Not stated	1,525	271,111
R	Refusal	0	0
X	Don't know	11	1,350
		======	=========
		20,710	3,420,337

Coverage: Respondents (DISAB=1) who answered C38=1
Source: Participation and Activity Limitation Survey, 2001

Variable Name: **C39E** *Position:* 482 *Length:* 2
Collection Name: AC39E

What were the difficulties? I will read you a list. Please answer yes or no to each: Other?

		FREQ	WTD
1	Yes	519	79,219
3	No	748	113,443
93	Not applicable	17,910	2,956,359
98	Not stated	1,525	270,190
R	Refusal	0	0
X	Don't know	8	1,126
		======	=========
		20,710	3,420,337

Coverage: Respondents (DISAB=1) who answered C38=1
Source: Participation and Activity Limitation Survey, 2001

Variable Name: **C40** *Position:* 484 *Length:* 2
Collection Name: AC40

Who pays for the help you receive (..... receives)?

		FREQ	WTD
0	Valid data	12,447	2,088,321
93	Not applicable	6,596	1,029,114
98	Not stated	1,598	292,848
R	Refusal	3	586
X	Don't know	66	9,468
		======	=========
		20,710	3,420,337

Coverage: Respondents (DISAB=1) who reported receiving help with everyday activities (RECHELP=1)
Source: Participation and Activity Limitation Survey, 2001

Variable Name: **C40A** *Position:* 486 *Length:* 2
Collection Name: AC40A

Who pays for the help you receive (..... receives): no one, it's free?

		FREQ	WTD
0	Not selected	12,408	2,018,441
1	Yes	8,302	1,401,897
		======	=========
		20,710	3,420,338

Coverage: Respondents (DISAB=1) who reported receiving help with everyday activities (RECHELP=1)
Source: Participation and Activity Limitation Survey, 2001

Variable Name: **C40B** *Position:* 488 *Length:* 2
Collection Name: AC40B

Who pays for the help you receive (..... receives): yourself (.....) or family living with you (him / her)?

		FREQ	WTD
0	Not selected	16,902	2,756,350
1	Yes	3,808	663,988
		======	=========
		20,710	3,420,338

Coverage: Respondents (DISAB=1) who reported receiving help with everyday activities (RECHELP=1)
Source: Participation and Activity Limitation Survey, 2001

Variable Name: **C40C** *Position:* 490 *Length:* 2
Collection Name: AC40C

Who pays for the help you receive (..... receives): family not living with you (him / her)?

		FREQ	WTD
0	Not selected	20,513	3,386,230
1	Yes	197	34,108
		======	=========
		20,710	3,420,338

Coverage: Respondents (DISAB=1) who reported receiving help with everyday activities (RECHELP=1)
Source: Participation and Activity Limitation Survey, 2001

Variable Name: **C40D** *Position:* 492 *Length:* 2
Collection Name: AC40D

Who pays for the help you receive (..... receives): private health insurance, e.g., employer insurance plan?

		FREQ	WTD
0	Not selected	20,620	3,404,322
1	Yes	90	16,016
		======	=========
		20,710	3,420,338

Coverage: Respondents (DISAB=1) who reported receiving help with everyday activities (RECHELP=1)
Source: Participation and Activity Limitation Survey, 2001

Variable Name:	**C40E**	*Position:*	494	*Length:*	2
Collection Name:	AC40E				

Who pays for the help you receive (..... receives): home care program?

		FREQ	WTD
0	Not selected	20,256	3,355,213
1	Yes	454	65,125
		======	=========
		20,710	3,420,338

Coverage: Respondents (DISAB=1) who reported receiving help with everyday activities (RECHELP=1)
Source: Participation and Activity Limitation Survey, 2001

Variable Name:	**C40F**	*Position:*	496	*Length:*	2
Collection Name:	AC40F				

Who pays for the help you receive (..... receives): voluntary organization?

		FREQ	WTD
0	Not selected	20,649	3,409,836
1	Yes	61	10,502
		======	=========
		20,710	3,420,338

Coverage: Respondents (DISAB=1) who reported receiving help with everyday activities (RECHELP=1)
Source: Participation and Activity Limitation Survey, 2001

Variable Name:	**C40G**	*Position:*	498	*Length:*	2
Collection Name:	AC40G				

Who pays for the help you receive (..... receives): other private source?

		FREQ	WTD
0	Not selected	20,592	3,403,261
1	Yes	118	17,077
		======	=========
		20,710	3,420,338

Coverage: Respondents (DISAB=1) who reported receiving help with everyday activities (RECHELP=1)
Source: Participation and Activity Limitation Survey, 2001

Variable Name: **C40H** *Position:* 500 *Length:* 2
Collection Name: AC40H

Who pays for the help you receive (..... receives): other public source, e.g., government health insurance plan?

		FREQ	WTD
0	Not selected	19,647	3,272,104
1	Yes	1,063	148,234
		======	=========
		20,710	3,420,338

Coverage: Respondents (DISAB=1) who reported receiving help with everyday activities (RECHELP=1)
Source: Participation and Activity Limitation Survey, 2001

Variable Name: **C41** *Position:* 502 *Length:* 2
Collection Name: AC41

Is the cost to you (.....) or your (his / her) family living with you (him/ her)...

		FREQ	WTD
1	fully reimbursed	138	19,760
2	partially reimbursed	282	35,532
3	not reimbursed	3,231	582,609
93	Not applicable	15,304	2,463,502
98	Not stated	1,696	311,409
R	Refusal	1	155
X	Don't know	58	7,371
		======	=========
		20,710	3,420,338

Coverage: Respondents (DISAB=1) who answered C40B=1
Source: Participation and Activity Limitation Survey, 2001

Variable Name: **C42A** *Position:* 504 *Length:* 2
Collection Name: AC42A

From what source does the reimbursement come? I will read you a list Please answer yes or no to each: Government tax credit?

		FREQ	WTD
1	Yes	81	11,510
3	No	266	32,731
93	Not applicable	18,594	3,053,636
98	Not stated	1,752	319,250
R	Refusal	1	263
X	Don't know	16	2,948
		======	=========
		20,710	3,420,338

Coverage: Respondents (DISAB=1) who answered C41=1 or 2
Source: Participation and Activity Limitation Survey, 2001

Variable Name: **C42B** *Position:* 506 *Length:* 2
Collection Name: AC42B

From what source does the reimbursement come? I will read you a list Please answer yes or no to each: Direct government financial support?

		FREQ	WTD
1	Yes	192	25,355
3	No	175	21,604
93	Not applicable	18,594	3,053,636
98	Not stated	1,741	318,885
R	Refusal	1	263
X	Don't know	7	595
		======	=========
		20,710	3,420,338

Coverage: Respondents (DISAB=1) who answered C41=1 or 2
Source: Participation and Activity Limitation Survey, 2001

Variable Name: **C42C** *Position:* 508 *Length:* 2
Collection Name: AC42C

From what source does the reimbursement come? I will read you a list Please answer yes or no to each: Private health insurance?

		FREQ	WTD
1	Yes	98	11,859
3	No	253	32,756
93	Not applicable	18,594	3,053,636
98	Not stated	1,757	319,885
R	Refusal	1	263
X	Don't know	7	1,938
		======	=========
		20,710	3,420,337

Coverage: Respondents (DISAB=1) who answered C41=1 or 2
Source: Participation and Activity Limitation Survey, 2001

Variable Name: **C42D** *Position:* 510 *Length:* 2
Collection Name: AC42D

From what source does the reimbursement come? I will read you a list Please answer yes or no to each: Other source?

		FREQ	WTD
1	Yes	99	10,759
3	No	259	36,217
93	Not applicable	18,594	3,053,636
98	Not stated	1,748	318,223
R	Refusal	1	263
X	Don't know	9	1,241
		======	=========
		20,710	3,420,339

Coverage: Respondents (DISAB=1) who answered C41=1 or 2
Source: Participation and Activity Limitation Survey, 2001

Variable Name: **C45A** *Position:* 512 *Length:* 2
Collection Name: AC45A

Why do you (does.....) not receive the help you NEED (he / she NEEDS)? I will read you a list of possible reasons. Please answer yes or no to each: you (.....) applied for home care and were (was) turned down?

		FREQ	WTD
1	Yes	318	53,229
3	No	3,524	591,405
93	Not applicable	14,492	2,345,414
98	Not stated	2,344	424,040
R	Refusal	0	0
X	Don't know	32	6,250
		======	=========
		20,710	3,420,338

Coverage: Respondents (DISAB=1) who reported needing help with everyday activities (NEEDHELP=1)
Source: Participation and Activity Limitation Survey, 2001

Variable Name: **C45B** *Position:* 514 *Length:* 2
Collection Name: AC45B

Why do you (does.....) not receive the help you NEED (he / she NEEDS)? I will read you a list of possible reasons. Please answer yes or no to each: you are (.....is) presently on a waiting list?

		FREQ	WTD
1	Yes	187	33,484
3	No	3,651	611,456
93	Not applicable	14,492	2,345,414
98	Not stated	2,346	423,860
R	Refusal	0	0
X	Don't know	34	6,124
		======	=========
		20,710	3,420,338

Coverage: Respondents (DISAB=1) who reported needing help with everyday activities (NEEDHELP=1)
Source: Participation and Activity Limitation Survey, 2001

Variable Name: **C45C** *Position:* 516 *Length:* 2
Collection Name: AC45C

Why do you (does.....) not receive the help you NEED (he / she NEEDS)? I will read you a list of possible reasons. Please answer yes or no to each: it is not covered by insurance?

		FREQ	WTD
1	Yes	1,072	192,956
3	No	2,551	418,811
93	Not applicable	14,492	2,345,414
98	Not stated	2,351	423,893
R	Refusal	0	0
X	Don't know	244	39,264
		======	=========
		20,710	3,420,338

Coverage: Respondents (DISAB=1) who reported needing help with everyday activities (NEEDHELP=1)
Source: Participation and Activity Limitation Survey, 2001

Variable Name:	**C45D**	*Position:*	518	*Length:*	2
Collection Name:	AC45D				

Why do you (does.....) not receive the help you NEED (he / she NEEDS)? I will read you a list of possible reasons. Please answer yes or no to each: it is too expensive?

		FREQ	WTD
1	Yes	2,114	376,512
3	No	1,611	249,813
93	Not applicable	14,492	2,345,414
98	Not stated	2,360	427,985
R	Refusal	0	0
X	Don't know	133	20,613
		======	=========
		20,710	3,420,337

Coverage: Respondents (DISAB=1) who reported needing help with everyday activities (NEEDHELP=1)
Source: Participation and Activity Limitation Survey, 2001

Variable Name:	**C45E**	*Position:*	520	*Length:*	2
Collection Name:	AC45E				

Why do you (does.....) not receive the help you NEED (he / she NEEDS)? I will read you a list of possible reasons. Please answer yes or no to each: you do (.....does) not know where to obtain it?

		FREQ	WTD
1	Yes	978	176,732
3	No	2,824	464,620
93	Not applicable	14,492	2,345,414
98	Not stated	2,354	425,119
R	Refusal	0	0
X	Don't know	62	8,453
		======	=========
		20,710	3,420,338

Coverage: Respondents (DISAB=1) who reported needing help with everyday activities (NEEDHELP=1)
Source: Participation and Activity Limitation Survey, 2001

Variable Name: **C45F** *Position:* 522 *Length:* 2
Collection Name: AC45F

Why do you (does.....) not receive the help you NEED (he / she NEEDS)? I will read you a list of possible reasons. Please answer yes or no to each: help is not available in the area?

		FREQ	WTD
1	Yes	493	76,373
3	No	2,954	498,413
93	Not applicable	14,492	2,345,414
98	Not stated	2,361	428,793
R	Refusal	0	0
X	Don't know	410	71,345
		======	=========
		20,710	3,420,338

Coverage: Respondents (DISAB=1) who reported needing help with everyday activities (NEEDHELP=1)
Source: Participation and Activity Limitation Survey, 2001

Variable Name: **C45G** *Position:* 524 *Length:* 2
Collection Name: AC45G

Why do you (does.....) not receive the help you NEED (he / she NEEDS)? I will read you a list of possible reasons. Please answer yes or no to each: informal help, e.g., from family or friends, is not available?

		FREQ	WTD
1	Yes	1,172	197,663
3	No	2,646	441,853
93	Not applicable	14,492	2,345,414
98	Not stated	2,356	427,558
R	Refusal	0	0
X	Don't know	44	7,850
		======	=========
		20,710	3,420,338

Coverage: Respondents (DISAB=1) who reported needing help with everyday activities (NEEDHELP=1)
Source: Participation and Activity Limitation Survey, 2001

Variable Name: **C45H** *Position:* 526 *Length:* 2
Collection Name: AC45H

Why do you (does.....) not receive the help you NEED (he / she NEEDS)? I will read you a list of possible reasons. Please answer yes or no to each: another reason?

		FREQ	WTD
1	Yes	804	132,862
3	No	2,974	506,591
93	Not applicable	14,492	2,345,414
98	Not stated	2,394	429,321
R	Refusal	0	0
X	Don't know	46	6,149
		======	=========
		20,710	3,420,337

Coverage: Respondents (DISAB=1) who reported needing help with everyday activities (NEEDHELP=1)
Source: Participation and Activity Limitation Survey, 2001

Variable Name: **C46** *Position:* 528 *Length:* 2
Collection Name: AC46

Which of the following best describes the control you have (.....has) in making decisions:

		FREQ	WTD
1	I make (He / She makes) all decisions about my (his / her) everyday activities....	13,445	2,268,541
2	I make (He / She makes) the majority of decisions about my (his / her) everyday activities...	4,019	653,776
3	I make (He / She makes) some of the decisions about my (his / her) everyday activities....	1,961	297,586
4	I don't (He / She does not) make any decisions about my (his / her) everyday activities	530	73,119
93	Not applicable	0	0
98	Not stated	728	121,971
R	Refusal	5	401
X	Don't know	22	4,944
		======	=========
		20,710	3,420,338

Coverage: All respondents (DISAB=1)
Source: Participation and Activity Limitation Survey, 2001

Variable Name: **C47A** *Position:* 530 *Length:* 2
Collection Name: AC47A

In the PAST 12 MONTHS, how often have you (has.....) seen or talked about your (his / her) physical, emotional or mental condition, with a physician (including general practitioners and specialists)?

		FREQ	WTD
1	At least once a week	838	135,870
2	At least once a month	6,317	1,134,410
3	Less than once a month	10,764	1,761,353
4	Never	2,037	267,574
93	Not applicable	0	0
98	Not stated	688	113,944
R	Refusal	5	379
X	Don't know	61	6,807
		======	=========
		20,710	3,420,337

Coverage: All respondents (DISAB=1)
Source: Participation and Activity Limitation Survey, 2001

Variable Name: **C47B** *Position:* 532 *Length:* 2
Collection Name: AC47B

In the PAST 12 MONTHS, how often have you (has.....) seen or talked about your (his / her) physical, emotional or mental condition, with a physiotherapist or occupational therapist?

		FREQ	WTD
1	At least once a week	1,203	182,834
2	At least once a month	837	129,214
3	Less than once a month	2,061	360,126
4	Never	15,818	2,620,553
93	Not applicable	0	0
98	Not stated	700	114,449
R	Refusal	7	1,219
X	Don't know	84	11,942
		======	=========
		20,710	3,420,337

Coverage: All respondents (DISAB=1)
Source: Participation and Activity Limitation Survey, 2001

Variable Name:	**C47C**	*Position:*	534	*Length:*	2
Collection Name:	AC47C				

In the PAST 12 MONTHS, how often have you (has.....) seen or talked about your (his / her) physical, emotional or mental condition, with an audiologist or speech therapist?

		FREQ	WTD
1	At least once a week	54	6,866
2	At least once a month	104	14,944
3	Less than once a month	1,224	216,156
4	Never	18,549	3,055,019
93	Not applicable	0	0
98	Not stated	694	114,526
R	Refusal	7	1,219
X	Don't know	78	11,608
		======	=========
		20,710	3,420,338

Coverage: All respondents (DISAB=1)
Source: Participation and Activity Limitation Survey, 2001

Variable Name:	**C47D**	*Position:*	536	*Length:*	2
Collection Name:	AC47D				

In the PAST 12 MONTHS, how often have you (has.....) seen or talked about your (his / her) physical, emotional or mental condition, with a chiropractor?

		FREQ	WTD
1	At least once a week	366	66,142
2	At least once a month	755	148,132
3	Less than once a month	1,231	201,618
4	Never	17,586	2,877,517
93	Not applicable	0	0
98	Not stated	695	116,328
R	Refusal	7	1,219
X	Don't know	70	9,382
		======	=========
		20,710	3,420,338

Coverage: All respondents (DISAB=1)
Source: Participation and Activity Limitation Survey, 2001

Variable Name: **C47E** *Position:* 538 *Length:* 2
Collection Name: AC47E

In the PAST 12 MONTHS, how often have you (has.....) seen or talked about your (his / her) physical, emotional or mental condition, with a massage therapist?

		FREQ	WTD
1	At least once a week	322	48,392
2	At least once a month	515	87,098
3	Less than once a month	1,114	176,925
4	Never	17,972	2,983,838
93	Not applicable	0	0
98	Not stated	708	111,694
R	Refusal	7	1,219
X	Don't know	72	11,171
		======	=========
		20,710	3,420,337

Coverage: All respondents (DISAB=1)
Source: Participation and Activity Limitation Survey, 2001

Variable Name: **C47F** *Position:* 540 *Length:* 2
Collection Name: AC47F

In the PAST 12 MONTHS, how often have you (has.....) seen or talked about your (his / her) physical, emotional or mental condition, with a psychologist, social worker or counselor?

		FREQ	WTD
1	At least once a week	411	62,020
2	At least once a month	878	126,003
3	Less than once a month	1,599	228,816
4	Never	17,018	2,871,930
93	Not applicable	0	0
98	Not stated	705	114,722
R	Refusal	7	1,219
X	Don't know	92	15,628
		======	=========
		20,710	3,420,338

Coverage: All respondents (DISAB=1)
Source: Participation and Activity Limitation Survey, 2001

Variable Name:	**C47G**	*Position:*	542	*Length:*	2
Collection Name:	AC47G				

In the PAST 12 MONTHS, how often have you (has.....) seen or talked about your (his / her) physical, emotional or mental condition, with a psychologist, another health care or social service provider?

		FREQ	WTD
1	At least once a week	335	53,637
2	At least once a month	503	82,962
3	Less than once a month	1,320	237,007
4	Never	17,561	2,885,320
6	Undetermined	83	14,130
93	Not applicable	0	0
98	Not stated	806	129,405
R	Refusal	7	1,219
X	Don't know	95	16,658
		======	=========
		20,710	3,420,338

Coverage: All respondents (DISAB=1)
Source: Participation and Activity Limitation Survey, 2001

Variable Name:	**C48**	*Position:*	544	*Length:*	2
Collection Name:	AC48				

INCLUDE amounts not covered by insurance such as exclusions, deductibles and expenses over limits. EXCLUDE payments for which you have (..... has) been or will be reimbursed by any insurance or government program.

		FREQ	WTD
1	Yes	3,796	676,542
3	No	14,426	2,393,044
93	Not applicable	1,494	191,717
98	Not stated	883	145,777
R	Refusal	3	1,150
X	Don't know	108	12,108
		======	=========
		20,710	3,420,338

Coverage: Respondents (DISAB=1) who reported some contacts with health care and social service providers in past year (C47A-G=1 or 2 or 3)
Source: Participation and Activity Limitation Survey, 2001

Variable Name:	**C51**	*Position:*	546	*Length:*	2
Collection Name:	AC51				

In the past 12 months, was there ever a time when you felt you (..... felt that he / she) NEEDED health care or social services because of your (his / her) condition, but you (he / she) did not receive them?

		FREQ	WTD
1	Yes	2,918	486,579
3	No	16,945	2,786,491
93	Not applicable	0	0
98	Not stated	756	130,111
R	Refusal	5	1,188
X	Don't know	86	15,968
		======	=========
		20,710	3,420,337

Coverage: All respondents (DISAB=1)
Source: Participation and Activity Limitation Survey, 2001

Variable Name:	**C52A**	*Position:*	548	*Length:*	2
Collection Name:	AC52A				

Why did you (.....) not get these services? I will read you a list of reasons. Please answer yes or no to each: They are not covered by insurance?

		FREQ	WTD
1	Yes	1,236	202,307
3	No	1,516	251,594
93	Not applicable	17,036	2,803,647
98	Not stated	862	150,173
R	Refusal	0	0
X	Don't know	60	12,616
		======	=========
		20,710	3,420,337

Coverage: Respondents (DISAB=1) who answered C51=1
Source: Participation and Activity Limitation Survey, 2001

Variable Name:	**C52B**	*Position:*	550	*Length:*	2
Collection Name:	AC52B				

Why did you (.....) not get these services? I will read you a list of reasons. Please answer yes or no to each: They are too expensive?

		FREQ	WTD
1	Yes	1,456	242,354
3	No	1,318	216,614
93	Not applicable	17,036	2,803,647
98	Not stated	866	151,857
R	Refusal	0	0
X	Don't know	34	5,865
		======	=========
		20,710	3,420,337

Coverage: Respondents (DISAB=1) who answered C51=1
Source: Participation and Activity Limitation Survey, 2001

Variable Name:	**C52C**	*Position:*	552	*Length:*	2
Collection Name:	AC52C				

Why did you (.....) not get these services? I will read you a list of reasons. Please answer yes or no to each: Your (.....'s) condition is not serious enough

		FREQ	WTD
1	Yes	397	61,494
3	No	2,339	389,132
93	Not applicable	17,036	2,803,647
98	Not stated	873	153,822
R	Refusal	1	31
X	Don't know	64	12,212
		======	=========
		20,710	3,420,338

Coverage: Respondents (DISAB=1) who answered C51=1
Source: Participation and Activity Limitation Survey, 2001

Variable Name:	**C52D**	*Position:*	554	*Length:*	2
Collection Name:	AC52D				

Why did you (.....) not get these services? I will read you a list of reasons. Please answer yes or no to each: You do (.....does) not know where or how to obtain them?

		FREQ	WTD
1	Yes	560	83,446
3	No	2,222	376,759
93	Not applicable	17,036	2,803,647
98	Not stated	873	152,955
R	Refusal	0	0
X	Don't know	19	3,531
		======	=========
		20,710	3,420,338

Coverage: Respondents (DISAB=1) who answered C51=1
Source: Participation and Activity Limitation Survey, 2001

Variable Name:	**C52E**	*Position:*	556	*Length:*	2
Collection Name:	AC52E				

Why did you (.....) not get these services? I will read you a list of reasons. Please answer yes or no to each: They are not available in the area?

		FREQ	WTD
1	Yes	555	89,291
3	No	2,123	349,210
93	Not applicable	17,036	2,803,647
98	Not stated	878	154,425
R	Refusal	0	0
X	Don't know	118	23,764
		======	=========
		20,710	3,420,337

Coverage: Respondents (DISAB=1) who answered C51=1
Source: Participation and Activity Limitation Survey, 2001

Variable Name:	**C52F**	*Position:*	558	*Length:*	2
Collection Name:	AC52F				

Why did you (.....) not get these services? I will read you a list of reasons. Please answer yes or no to each: Another reason?

		FREQ	WTD
1	Yes	1,342	219,939
3	No	1,455	245,493
93	Not applicable	17,036	2,803,647
98	Not stated	856	148,159
R	Refusal	0	0
X	Don't know	21	3,099
		======	=========
		20,710	3,420,337

Coverage: Respondents (DISAB=1) who answered C51=1
Source: Participation and Activity Limitation Survey, 2001

Section: EDUCATION

Variable Name:	**D1**	*Position:*	560	*Length:*	2
Collection Name:	AD1				

In April 2001, were you (was.....) attending a school, college or university? (Include private schools, colleges or universities.

		FREQ	WTD
1	Yes	2,022	143,464
3	No	10,486	1,754,436
93	Not applicable	7,796	1,451,844
98	Not stated	401	69,988
R	Refusal	2	396
X	Don't know	3	209
		======	=========
		20,710	3,420,337

Coverage: Respondents (DISAB=1) aged 15-64
Source: Participation and Activity Limitation Survey, 2001

Variable Name: **D2** *Position:* 562 *Length:* 2
Collection Name: AD2

Did you (.....) attend school after April, 1996?

		FREQ	WTD
1	Yes	2,302	273,227
3	No	8,142	1,474,378
93	Not applicable	9,818	1,595,309
98	Not stated	402	70,350
R	Refusal	2	404
X	Don't know	44	6,670
		======	=========
		20,710	3,420,338

Coverage: Respondents (DISAB=1) aged 15-64 who DID NOT answer D1=1
Source: Participation and Activity Limitation Survey, 2001

Variable Name: **D3** *Position:* 564 *Length:* 2
Collection Name: AD3

Were you (Was.....) enrolled as a:

		FREQ	WTD
1	full-time student	1,629	103,967
2	part-time student	382	38,620
93	Not applicable	18,287	3,206,885
98	Not stated	406	70,322
R	Refusal	0	0
X	Don't know	6	542
		======	=========
		20,710	3,420,336

Coverage: Respondents (DISAB=1) aged 15-64 who answered D1=1
Source: Participation and Activity Limitation Survey, 2001

Variable Name: **D4** *Position:* 566 *Length:* 2
Collection Name: AD4

Were you (Was.....) studying part-time because of your (his / her) condition?

		FREQ	WTD
1	Yes	129	10,020
3	No	247	28,279
93	Not applicable	19,922	3,311,395
98	Not stated	411	70,607
R	Refusal	0	0
X	Don't know	1	36
		======	=========
		20,710	3,420,337

Coverage: Respondents (DISAB=1) aged 15-64 who answered D3=2
Source: Participation and Activity Limitation Survey, 2001

Variable Name: **D5** *Position:* 568 *Length:* 2
Collection Name: AD5

Were you (Was.....) taking any courses by correspondence or home study in April 2001? Consider only courses which can be used as credits towards a certificate, diploma or degree.

		FREQ	WTD
1	Yes	209	15,301
3	No	1,799	127,644
93	Not applicable	18,287	3,206,885
98	Not stated	410	70,366
R	Refusal	0	0
X	Don't know	5	141
		======	=========
		20,710	3,420,337

Coverage: Respondents (DISAB=1) aged 15-64 who answered D1=1
Source: Participation and Activity Limitation Survey, 2001

Variable Name: **D6** *Position:* 570 *Length:* 2
Collection Name: AD6

In April 2001, in what kind of school were you (was.....) enrolled? I will read you a list. Please specify one only.

		FREQ	WTD
1	Regular primary or secondary school	941	46,180
2	Special education school	147	9,060
3	Community college, CEGEP or technical institute	357	36,415
4	Private training institutes, for example, business schools or trade or vocational schools	129	14,151
5	University	342	26,626
6	Other,Specify	98	10,598
93	Not applicable	18,287	3,206,885
98	Not stated	405	70,196
R	Refusal	0	0
X	Don't know	4	227
		======	=========
		20,710	3,420,338

Coverage: Respondents (DISAB=1) aged 15-64 who answered D1=1
Source: Participation and Activity Limitation Survey, 2001

Variable Name: **D8** *Position:* 572 *Length:* 2
Collection Name: AD8

What type of certificate, diploma or degree were you (was.. ...) seeking? I will read you a list. Please indicate all that apply.

		FREQ	WTD
0	Valid data	817	77,329
93	Not applicable	19,379	3,262,352
98	Not stated	469	75,896
R	Refusal	0	0
X	Don't know	45	4,760
		======	=========
		20,710	3,420,337

Coverage: Respondents (DISAB=1) aged 15-64 who answered D6=3,4,5 or 6
Source: Participation and Activity Limitation Survey, 2001

Variable Name: **D8A** *Position:* 574 *Length:* 2
Collection Name: AD8A

What type of certificate, diploma or degree were you (was.....) seeking? I will read you a list. Please indicate all that apply.: Trades certificate or diploma?

		FREQ	WTD
0	Not selected	20,500	3,401,227
1	Yes	210	19,111
		======	=========
		20,710	3,420,338

Coverage: Respondents (DISAB=1) aged 15-64 who answered D6=3,4,5 or 6
Source: Participation and Activity Limitation Survey, 2001

Variable Name: **D8B** *Position:* 576 *Length:* 2
Collection Name: AD8B

What type of certificate, diploma or degree were you (was.....) seeking? I will read you a list. Please indicate all that apply.: Other non-university certificate or diploma (obtained at community college, CEGEP, technical institute, etc.)?

		FREQ	WTD
0	Not selected	20,453	3,389,966
1	Yes	257	30,372
		======	=========
		20,710	3,420,338

Coverage: Respondents (DISAB=1) aged 15-64 who answered D6=3,4,5 or 6
Source: Participation and Activity Limitation Survey, 2001

Variable Name:	**D8C**	*Position:*	578	*Length:*	2
Collection Name:	AD8C				

What type of certificate, diploma or degree were you (was.....) seeking? I will read you a list. Please indicate all that apply.: University certificate or diploma below bachelor level?

		FREQ	WTD
0	Not selected	20,648	3,414,273
1	Yes	62	6,064
		======	=========
		20,710	3,420,337

Coverage: Respondents (DISAB=1) aged 15-64 who answered D6=3,4,5 or 6
Source: Participation and Activity Limitation Survey, 2001

Variable Name:	**D8I**	*Position:*	580	*Length:*	2
Collection Name:	AD8I				

Derived variable: Bachelor's degree or higher

		FREQ	WTD
0	Not selected	20,366	3,394,639
1	Yes	344	25,699
		======	=========
		20,710	3,420,338

Coverage: Respondents (DISAB=1) aged 15-64 who answered D6=3,4,5 or 6
Source: Participation and Activity Limitation Survey, 2001
Note: D8I is derived from variables D8D-D8H

Variable Name:	**D9**	*Position:*	582	*Length:*	2
Collection Name:	AD9				

Did you (.....) require modified building features or services to attend school?

		FREQ	WTD
1	Yes	214	15,022
3	No	1,786	127,498
93	Not applicable	18,287	3,206,885
98	Not stated	416	70,660
R	Refusal	0	0
X	Don't know	7	273
		======	=========
		20,710	3,420,338

Coverage: Respondents (DISAB=1) aged 15-64 who answered D1=1
Source: Participation and Activity Limitation Survey, 2001

Variable Name: **D10A** *Position:* 584 *Length:* 2
Collection Name: AD10A

Did you (.....) require accessible classrooms?

		FREQ	WTD
1	Yes	103	7,505
3	No	102	6,960
93	Not applicable	20,080	3,334,656
98	Not stated	424	71,195
R	Refusal	0	0
X	Don't know	1	21
		======	=========
		20,710	3,420,337

Coverage: Respondents (DISAB=1) aged 15-64 who answered D9=1
Source: Participation and Activity Limitation Survey, 2001

Variable Name: **D10AA** *Position:* 586 *Length:* 2
Collection Name: AD10AA

Was this available to you (.....), classrooms?

		FREQ	WTD
1	Yes	92	6,844
3	No	8	484
93	Not applicable	20,183	3,341,637
98	Not stated	427	71,373
R	Refusal	0	0
X	Don't know	0	0
		======	=========
		20,710	3,420,338

Coverage: Respondents (DISAB=1) aged 15-64 who answered D10A=1
Source: Participation and Activity Limitation Survey, 2001

Variable Name: **D10B** *Position:* 588 *Length:* 2
Collection Name: AD10B

Did you (.....) require accessible washrooms?

		FREQ	WTD
1	Yes	100	7,256
3	No	105	7,240
93	Not applicable	20,080	3,334,656
98	Not stated	425	71,186
R	Refusal	0	0
X	Don't know	0	0
		======	=========
		20,710	3,420,338

Coverage: Respondents (DISAB=1) aged 15-64 who answered D9=1
Source: Participation and Activity Limitation Survey, 2001

Variable Name:	**D10AB**	*Position:*	590	*Length:*	2
Collection Name:	AD10AB				

Was this available to you (.....), accessible washrooms?

		FREQ	WTD
1	Yes	92	6,818
3	No	5	359
93	Not applicable	20,185	3,341,896
98	Not stated	428	71,265
R	Refusal	0	0
X	Don't know	0	0
		======	=========
		20,710	3,420,338

Coverage: Respondents (DISAB=1) aged 15-64 who answered D10B=1
Source: Participation and Activity Limitation Survey, 2001

Variable Name:	**D10C**	*Position:*	592	*Length:*	2
Collection Name:	AD10C				

Did you (.....) require accessible residences?

		FREQ	WTD
1	Yes	36	1,887
3	No	167	12,636
93	Not applicable	20,080	3,334,656
98	Not stated	425	71,089
R	Refusal	0	0
X	Don't know	2	69
		======	=========
		20,710	3,420,337

Coverage: Respondents (DISAB=1) aged 15-64 who answered D9=1
Source: Participation and Activity Limitation Survey, 2001

Variable Name:	**D10AC**	*Position:*	594	*Length:*	2
Collection Name:	AD10AC				

Was this available to you (.....), accessible residences?

		FREQ	WTD
1	Yes	31	1,473
3	No	3	267
93	Not applicable	20,249	3,347,361
98	Not stated	427	71,236
R	Refusal	0	0
X	Don't know	0	0
		======	=========
		20,710	3,420,337

Coverage: Respondents (DISAB=1) aged 15-64 who answered D10C=1
Source: Participation and Activity Limitation Survey, 2001

Variable Name: **D10D** *Position:* 596 *Length:* 2
Collection Name: AD10D

Did you (.....) require accessible buildings, excluding residences?

		FREQ	WTD
1	Yes	80	6,849
3	No	124	7,577
93	Not applicable	20,080	3,334,656
98	Not stated	425	71,231
R	Refusal	0	0
X	Don't know	1	25
		======	=========
		20,710	3,420,338

Coverage: Respondents (DISAB=1) aged 15-64 who answered D9=1
Source: Participation and Activity Limitation Survey, 2001

Variable Name: **D10AD** *Position:* 598 *Length:* 2
Collection Name: AD10AD

Was this available to you (.....), accessible buildings, excluding residences?

		FREQ	WTD
1	Yes	71	5,878
3	No	7	915
93	Not applicable	20,205	3,342,257
98	Not stated	427	71,288
R	Refusal	0	0
X	Don't know	0	0
		======	=========
		20,710	3,420,338

Coverage: Respondents (DISAB=1) aged 15-64 who answered D10D=1
Source: Participation and Activity Limitation Survey, 2001

Variable Name: **D10E** *Position:* 600 *Length:* 2
Collection Name: AD10E

Did you (.....) require accessible transportation?

		FREQ	WTD
1	Yes	101	6,500
3	No	105	8,037
93	Not applicable	20,080	3,334,656
98	Not stated	424	71,145
R	Refusal	0	0
X	Don't know	0	0
		======	=========
		20,710	3,420,338

Coverage: Respondents (DISAB=1) aged 15-64 who answered D9=1
Source: Participation and Activity Limitation Survey, 2001

Variable Name: **D10AE** *Position:* 602 *Length:* 2
Collection Name: AD10AE

Was this available to you (.....),accessible transportation?

		FREQ	WTD
1	Yes	92	5,721
3	No	7	720
93	Not applicable	20,185	3,342,693
98	Not stated	426	71,205
R	Refusal	0	0
X	Don't know	0	0
		======	=========
		20,710	3,420,339

Coverage: Respondents (DISAB=1) aged 15-64 who answered D10E=1
Source: Participation and Activity Limitation Survey, 2001

Variable Name: **D10F** *Position:* 604 *Length:* 2
Collection Name: AD10F

Did you (.....) require other feature or service?

		FREQ	WTD
1	Yes	71	5,443
3	No	134	9,154
93	Not applicable	20,080	3,334,656
98	Not stated	425	71,085
R	Refusal	0	0
X	Don't know	0	0
		======	=========
		20,710	3,420,338

Coverage: Respondents (DISAB=1) aged 15-64 who answered D9=1
Source: Participation and Activity Limitation Survey, 2001

Variable Name: **D10AF** *Position:* 606 *Length:* 2
Collection Name: AD10AF

Was this available to you (.....), other feature or service?

		FREQ	WTD
1	Yes	48	4,002
3	No	13	939
93	Not applicable	20,214	3,343,810
98	Not stated	435	71,587
R	Refusal	0	0
X	Don't know	0	0
		======	=========
		20,710	3,420,338

Coverage: Respondents (DISAB=1) aged 15-64 who answered D10F=1
Source: Participation and Activity Limitation Survey, 2001

Variable Name: **D11** *Position:* 608 *Length:* 2
Collection Name: AD11

Did you (.....) need any assistive devices or services to follow your (his / her) courses?

		FREQ	WTD
1	Yes	509	28,743
3	No	1,495	113,902
93	Not applicable	18,287	3,206,885
98	Not stated	406	70,176
R	Refusal	0	0
X	Don't know	13	631
		======	=========
		20,710	3,420,337

Coverage: Respondents (DISAB=1) aged 15-64 who answered D1=1
Source: Participation and Activity Limitation Survey, 2001

Variable Name: **D12A** *Position:* 610 *Length:* 2
Collection Name: AD12A

Did you (.....) need note takers or readers?

		FREQ	WTD
1	Yes	215	11,675
3	No	277	16,196
93	Not applicable	19,795	3,321,419
98	Not stated	419	70,841
R	Refusal	0	0
X	Don't know	4	207
		======	=========
		20,710	3,420,338

Coverage: Respondents (DISAB=1) aged 15-64 who answered D11=1
Source: Participation and Activity Limitation Survey, 2001

Variable Name: **D12AA** *Position:* 612 *Length:* 2
Collection Name: AD12AA

Was it made available to you (.....)?

		FREQ	WTD
1	Yes	180	9,876
3	No	30	1,674
93	Not applicable	20,076	3,337,821
98	Not stated	423	70,939
R	Refusal	0	0
X	Don't know	1	27
		======	=========
		20,710	3,420,337

Coverage: Respondents (DISAB=1) aged 15-64 who answered D12A=1
Source: Participation and Activity Limitation Survey, 2001

Variable Name: **D12B** *Position:* 614 *Length:* 2
Collection Name: AD12B

Did you (.....) need a tutor or teacher's aide?

		FREQ	WTD
1	Yes	376	20,474
3	No	121	7,450
93	Not applicable	19,795	3,321,419
98	Not stated	416	70,887
R	Refusal	0	0
X	Don't know	2	107
		======	=========
		20,710	3,420,337

Coverage: Respondents (DISAB=1) aged 15-64 who answered D11=1
Source: Participation and Activity Limitation Survey, 2001

Variable Name: **D12AB** *Position:* 616 *Length:* 2
Collection Name: AD12AB

Was it made available to you (.....)?

		FREQ	WTD
1	Yes	324	16,305
3	No	43	3,766
93	Not applicable	19,918	3,328,977
98	Not stated	425	71,290
R	Refusal	0	0
X	Don't know	0	0
		======	=========
		20,710	3,420,338

Coverage: Respondents (DISAB=1) aged 15-64 who answered D12B=1
Source: Participation and Activity Limitation Survey, 2001

Variable Name: **D12C** *Position:* 618 *Length:* 2
Collection Name: AD12C

Did you (.....) need a computer with Braille, large print or speech access?

		FREQ	WTD
1	Yes	80	4,186
3	No	403	23,236
93	Not applicable	19,795	3,321,419
98	Not stated	424	71,211
R	Refusal	0	0
X	Don't know	8	285
		======	=========
		20,710	3,420,337

Coverage: Respondents (DISAB=1) aged 15-64 who answered D11=1
Source: Participation and Activity Limitation Survey, 2001

Variable Name: **D12AC** *Position:* 620 *Length:* 2
Collection Name: AD12AC

Was it made available to you (.....)?

		FREQ	WTD
1	Yes	56	2,510
3	No	20	1,554
93	Not applicable	20,206	3,344,941
98	Not stated	426	71,261
R	Refusal	0	0
X	Don't know	2	72
		======	=========
		20,710	3,420,338

Coverage: Respondents (DISAB=1) aged 15-64 who answered D12C=1
Source: Participation and Activity Limitation Survey, 2001

Variable Name: **D12D** *Position:* 622 *Length:* 2
Collection Name: AD12D

Did you (.....) need talking books?

		FREQ	WTD
1	Yes	60	2,846
3	No	419	24,291
93	Not applicable	19,795	3,321,419
98	Not stated	425	71,253
R	Refusal	1	20
X	Don't know	10	509
		======	=========
		20,710	3,420,338

Coverage: Respondents (DISAB=1) aged 15-64 who answered D11=1
Source: Participation and Activity Limitation Survey, 2001

Variable Name: **D12AD** *Position:* 624 *Length:* 2
Collection Name: AD12AD

Was it made available to you (.....)?

		FREQ	WTD
1	Yes	36	1,381
3	No	19	1,317
93	Not applicable	20,225	3,346,239
98	Not stated	428	71,352
R	Refusal	0	0
X	Don't know	2	48
		======	=========
		20,710	3,420,337

Coverage: Respondents (DISAB=1) aged 15-64 who answered D12D=1
Source: Participation and Activity Limitation Survey, 2001

Variable Name:	**D12E**	*Position:*	626	*Length:*	2
Collection Name:	AD12E				

Did you (.....) need magnifiers or CCTV's (Closed circuit television readers)?

		FREQ	WTD
1	Yes	39	2,153
3	No	447	25,418
93	Not applicable	19,795	3,321,419
98	Not stated	421	71,094
R	Refusal	1	36
X	Don't know	7	217
		======	=========
		20,710	3,420,337

Coverage: Respondents (DISAB=1) aged 15-64 who answered D11=1
Source: Participation and Activity Limitation Survey, 2001

Variable Name:	**D12AE**	*Position:*	628	*Length:*	2
Collection Name:	AD12AE				

Was it made available to you (.....)?

		FREQ	WTD
1	Yes	30	1,610
3	No	7	493
93	Not applicable	20,250	3,347,090
98	Not stated	422	71,125
R	Refusal	1	20
X	Don't know	0	0
		======	=========
		20,710	3,420,338

Coverage: Respondents (DISAB=1) aged 15-64 who answered D12E=1
Source: Participation and Activity Limitation Survey, 2001

Variable Name:	**D12F**	*Position:*	630	*Length:*	2
Collection Name:	AD12F				

Did you (.....) need Braille or large print reading materials?

		FREQ	WTD
1	Yes	42	1,352
3	No	448	26,341
93	Not applicable	19,795	3,321,419
98	Not stated	423	71,150
R	Refusal	0	0
X	Don't know	2	75
		======	=========
		20,710	3,420,337

Coverage: Respondents (DISAB=1) aged 15-64 who answered D11=1
Source: Participation and Activity Limitation Survey, 2001

Variable Name: **D12AF** *Position:* 632 *Length:* 2
Collection Name: AD12AF

Was it made available to you (.....)?

		FREQ	WTD
1	Yes	30	882
3	No	10	419
93	Not applicable	20,245	3,347,836
98	Not stated	425	71,201
R	Refusal	0	0
X	Don't know	0	0
		======	=========
		20,710	3,420,338

Coverage: Respondents (DISAB=1) aged 15-64 who answered D12F=1
Source: Participation and Activity Limitation Survey, 2001

Variable Name: **D12G** *Position:* 634 *Length:* 2
Collection Name: AD12G

Did you (.....) need a Sign language interpreter?

		FREQ	WTD
1	Yes	29	1,157
3	No	459	26,339
93	Not applicable	19,795	3,321,419
98	Not stated	425	71,341
R	Refusal	1	36
X	Don't know	1	46
		======	=========
		20,710	3,420,338

Coverage: Respondents (DISAB=1) aged 15-64 who answered D11=1
Source: Participation and Activity Limitation Survey, 2001

Variable Name: **D12AG** *Position:* 636 *Length:* 2
Collection Name: AD12AG

Was it made available to you (.....)?

		FREQ	WTD
1	Yes	19	752
3	No	8	328
93	Not applicable	20,256	3,347,840
98	Not stated	427	71,417
R	Refusal	0	0
X	Don't know	0	0
		======	=========
		20,710	3,420,337

Coverage: Respondents (DISAB=1) aged 15-64 who answered D12G=1
Source: Participation and Activity Limitation Survey, 2001

Variable Name: **D12H** *Position:* 638 *Length:* 2
Collection Name: AD12H

Did you (.....) need recording equipment or portable note-takers?

		FREQ	WTD
1	Yes	99	6,930
3	No	389	20,735
93	Not applicable	19,795	3,321,419
98	Not stated	422	71,092
R	Refusal	0	0
X	Don't know	5	162
		======	=========
		20,710	3,420,338

Coverage: Respondents (DISAB=1) aged 15-64 who answered D11=1
Source: Participation and Activity Limitation Survey, 2001

Variable Name: **D12AH** *Position:* 640 *Length:* 2
Collection Name: AD12AH

Was it made available to you (.....)?

		FREQ	WTD
1	Yes	59	3,950
3	No	33	2,634
93	Not applicable	20,189	3,342,316
98	Not stated	427	71,391
R	Refusal	1	20
X	Don't know	1	27
		======	=========
		20,710	3,420,338

Coverage: Respondents (DISAB=1) aged 15-64 who answered D12H=1
Source: Participation and Activity Limitation Survey, 2001

Variable Name: **D12I** *Position:* 642 *Length:* 2
Collection Name: AD12I

Did you (.....) need attendant care services?

		FREQ	WTD
1	Yes	89	4,740
3	No	402	23,005
93	Not applicable	19,795	3,321,419
98	Not stated	423	71,128
R	Refusal	0	0
X	Don't know	1	46
		======	=========
		20,710	3,420,338

Coverage: Respondents (DISAB=1) aged 15-64 who answered D11=1
Source: Participation and Activity Limitation Survey, 2001

Variable Name:	**D12AI**	*Position:*	644	*Length:*	2
Collection Name:	AD12AI				

Was it made available to you (.....)?

		FREQ	WTD
1	Yes	70	3,839
3	No	12	501
93	Not applicable	20,198	3,344,470
98	Not stated	430	71,529
R	Refusal	0	0
X	Don't know	0	0
		======	=========
		20,710	3,420,339

Coverage: Respondents (DISAB=1) aged 15-64 who answered D12I=1
Source: Participation and Activity Limitation Survey, 2001

Variable Name:	**D12J**	*Position:*	646	*Length:*	2
Collection Name:	AD12J				

Did you (.....) need other aid or service?

		FREQ	WTD
1	Yes	111	7,834
3	No	379	19,935
93	Not applicable	19,795	3,321,419
98	Not stated	420	71,005
R	Refusal	1	20
X	Don't know	4	125
		======	=========
		20,710	3,420,338

Coverage: Respondents (DISAB=1) aged 15-64 who answered D11=1
Source: Participation and Activity Limitation Survey, 2001

Variable Name:	**D12AJ**	*Position:*	648	*Length:*	2
Collection Name:	AD12AJ				

Was it made available to you (.....)?

		FREQ	WTD
1	Yes	79	4,721
3	No	16	1,362
93	Not applicable	20,179	3,341,499
98	Not stated	435	72,733
R	Refusal	0	0
X	Don't know	1	23
		======	=========
		20,710	3,420,338

Coverage: Respondents (DISAB=1) aged 15-64 who answered D12J=1
Source: Participation and Activity Limitation Survey, 2001

Variable Name: **D13** *Position:* 650 *Length:* 2
Collection Name: AD13

Did you (.....) have your (his / her) condition when you were (he / she was) attending school (after April, 1996)?

		FREQ	WTD
1	Yes	1,860	220,025
3	No	403	48,110
93	Not applicable	18,006	3,076,761
98	Not stated	430	73,453
R	Refusal	0	0
X	Don't know	11	1,990
		======	=========
		20,710	3,420,339

Coverage: Respondents (DISAB=1) aged 15-64 who answered D2=1
Source: Participation and Activity Limitation Survey, 2001

Variable Name: **D14** *Position:* 652 *Length:* 2
Collection Name: AD14

Did you (.....) require modified building features or services to attend school?

		FREQ	WTD
1	Yes	236	25,863
3	No	1,578	185,894
93	Not applicable	18,420	3,126,861
98	Not stated	474	81,315
R	Refusal	0	0
X	Don't know	2	405
		======	=========
		20,710	3,420,338

Coverage: Respondents (DISAB=1) aged 15-64 who answered D13=1
Source: Participation and Activity Limitation Survey, 2001

Variable Name: **D15A** *Position:* 654 *Length:* 2
Collection Name: AD15A

Did you (.....) require accessible classrooms?

		FREQ	WTD
1	Yes	96	11,631
3	No	126	12,531
93	Not applicable	20,000	3,313,160
98	Not stated	486	82,697
R	Refusal	1	33
X	Don't know	1	287
		======	=========
		20,710	3,420,339

Coverage: Respondents (DISAB=1) aged 15-64 who answered D14=1
Source: Participation and Activity Limitation Survey, 2001

Variable Name: **D15AA** *Position:* 656 *Length:* 2
Collection Name: AD15AA

Was this available to you (.....)?

		FREQ	WTD
1	Yes	82	8,610
3	No	9	1,069
93	Not applicable	20,128	3,326,010
98	Not stated	488	83,297
R	Refusal	3	1,352
X	Don't know	0	0
		======	=========
		20,710	3,420,338

Coverage: Respondents (DISAB=1) aged 15-64 who answered D15A=1
Source: Participation and Activity Limitation Survey, 2001

Variable Name: **D15B** *Position:* 658 *Length:* 2
Collection Name: AD15B

Did you (.....) require accessible washrooms?

		FREQ	WTD
1	Yes	84	8,703
3	No	140	15,615
93	Not applicable	20,000	3,313,160
98	Not stated	484	82,541
R	Refusal	2	320
X	Don't know	0	0
		======	=========
		20,710	3,420,339

Coverage: Respondents (DISAB=1) aged 15-64 who answered D14=1
Source: Participation and Activity Limitation Survey, 2001

Variable Name:	**D15AB**	*Position:*	660	*Length:*	2
Collection Name:	AD15AB				

Was this available to you (.....)?

		FREQ	WTD
1	Yes	70	5,840
3	No	6	606
93	Not applicable	20,142	3,329,094
98	Not stated	489	83,446
R	Refusal	3	1,352
X	Don't know	0	0
		======	=========
		20,710	3,420,338

Coverage: Respondents (DISAB=1) aged 15-64 who answered D15B=1
Source: Participation and Activity Limitation Survey, 2001

Variable Name:	**D15C**	*Position:*	662	*Length:*	2
Collection Name:	AD15C				

Did you (.....) require accessible residences?

		FREQ	WTD
1	Yes	42	4,582
3	No	180	19,692
93	Not applicable	20,000	3,313,160
98	Not stated	483	82,171
R	Refusal	3	690
X	Don't know	2	44
		======	=========
		20,710	3,420,339

Coverage: Respondents (DISAB=1) aged 15-64 who answered D14=1
Source: Participation and Activity Limitation Survey, 2001

Variable Name:	**D15AC**	*Position:*	664	*Length:*	2
Collection Name:	AD15AC				

Was this available to you (.....)?

		FREQ	WTD
1	Yes	34	2,255
3	No	3	264
93	Not applicable	20,185	3,333,585
98	Not stated	484	82,302
R	Refusal	4	1,932
X	Don't know	0	0
		======	=========
		20,710	3,420,338

Coverage: Respondents (DISAB=1) aged 15-64 who answered D15C=1
Source: Participation and Activity Limitation Survey, 2001

Variable Name: **D15D** *Position:* 666 *Length:* 2
Collection Name: AD15D

Did you (.....) require accessible buildings, excluding residences?

		FREQ	WTD
1	Yes	76	8,587
3	No	149	15,787
93	Not applicable	20,000	3,313,160
98	Not stated	482	82,082
R	Refusal	2	695
X	Don't know	1	28
		======	=========
		20,710	3,420,339

Coverage: Respondents (DISAB=1) aged 15-64 who answered D14=1
Source: Participation and Activity Limitation Survey, 2001

Variable Name: **D15AD** *Position:* 668 *Length:* 2
Collection Name: AD15AD

Was this available to you (.....)?

		FREQ	WTD
1	Yes	61	4,532
3	No	4	1,750
93	Not applicable	20,152	3,329,669
98	Not stated	488	82,460
R	Refusal	5	1,927
X	Don't know	0	0
		======	=========
		20,710	3,420,338

Coverage: Respondents (DISAB=1) aged 15-64 who answered D15D=1
Source: Participation and Activity Limitation Survey, 2001

Variable Name: **D15E** *Position:* 670 *Length:* 2
Collection Name: AD15E

Did you (.....) require accessible transportation?

		FREQ	WTD
1	Yes	93	11,370
3	No	132	13,208
93	Not applicable	20,000	3,313,160
98	Not stated	483	82,281
R	Refusal	1	33
X	Don't know	1	287
		======	=========
		20,710	3,420,339

Coverage: Respondents (DISAB=1) aged 15-64 who answered D14=1
Source: Participation and Activity Limitation Survey, 2001

Variable Name: **D15AE** *Position:* 672 *Length:* 2
Collection Name: AD15AE

Was this available to you (.....)?

		FREQ	WTD
1	Yes	71	4,548
3	No	13	2,720
93	Not applicable	20,134	3,326,687
98	Not stated	486	84,048
R	Refusal	6	2,335
X	Don't know	0	0
		======	=========
		20,710	3,420,338

Coverage: Respondents (DISAB=1) aged 15-64 who answered D15E=1
Source: Participation and Activity Limitation Survey, 2001

Variable Name: **D15F** *Position:* 674 *Length:* 2
Collection Name: AD15F

Did you (.....) require other feature or service?

		FREQ	WTD
1	Yes	93	9,768
3	No	136	15,050
93	Not applicable	20,000	3,313,160
98	Not stated	479	81,905
R	Refusal	2	455
X	Don't know	0	0
		======	=========
		20,710	3,420,338

Coverage: Respondents (DISAB=1) aged 15-64 who answered D14=1
Source: Participation and Activity Limitation Survey, 2001

Variable Name: **D15AF** *Position:* 676 *Length:* 2
Collection Name: AD15AF

Was this available to you (.....)?

		FREQ	WTD
1	Yes	66	6,113
3	No	15	1,233
93	Not applicable	20,138	3,328,665
98	Not stated	486	82,284
R	Refusal	4	1,932
X	Don't know	1	111
		======	=========
		20,710	3,420,338

Coverage: Respondents (DISAB=1) aged 15-64 who answered D15F=1
Source: Participation and Activity Limitation Survey, 2001

Variable Name: **D16** *Position:* 678 *Length:* 2
Collection Name: AD16

Did you (.....) need any assistive devices or services to follow your (his / her) courses?

		FREQ	WTD
1	Yes	381	36,972
3	No	1,437	176,458
93	Not applicable	18,420	3,126,861
98	Not stated	467	79,750
R	Refusal	0	0
X	Don't know	5	296
		======	=========
		20,710	3,420,337

Coverage: Respondents (DISAB=1) aged 15-64 who answered D13=1
Source: Participation and Activity Limitation Survey, 2001

Variable Name: **D17A** *Position:* 680 *Length:* 2
Collection Name: AD17A

Did you (.....) need note takers or readers?

		FREQ	WTD
1	Yes	131	10,942
3	No	227	23,091
93	Not applicable	19,862	3,303,615
98	Not stated	490	82,691
R	Refusal	0	0
X	Don't know	0	0
		======	=========
		20,710	3,420,339

Coverage: Respondents (DISAB=1) aged 15-64 who answered D16=1
Source: Participation and Activity Limitation Survey, 2001

Variable Name: **D17AA** *Position:* 682 *Length:* 2
Collection Name: AD17AA

Was it made available to you (.....)?

		FREQ	WTD
1	Yes	98	7,601
3	No	29	3,223
93	Not applicable	20,089	3,326,706
98	Not stated	493	82,787
R	Refusal	0	0
X	Don't know	1	21
		======	=========
		20,710	3,420,338

Coverage: Respondents (DISAB=1) aged 15-64 who answered D17A=1
Source: Participation and Activity Limitation Survey, 2001

Variable Name:	**D17B**	*Position:*	684	*Length:*	2
Collection Name:	AD17B				

Did you (.....) need a tutor or teacher's aide?

		FREQ	WTD
1	Yes	239	17,656
3	No	121	16,480
93	Not applicable	19,862	3,303,615
98	Not stated	488	82,587
R	Refusal	0	0
X	Don't know	0	0
		======	=========
		20,710	3,420,338

Coverage: Respondents (DISAB=1) aged 15-64 who answered D16=1
Source: Participation and Activity Limitation Survey, 2001

Variable Name:	**D17AB**	*Position:*	686	*Length:*	2
Collection Name:	AD17AB				

Was it made available to you (.....)?

		FREQ	WTD
1	Yes	199	14,433
3	No	30	2,511
93	Not applicable	19,983	3,320,095
98	Not stated	498	83,299
R	Refusal	0	0
X	Don't know	0	0
		======	=========
		20,710	3,420,338

Coverage: Respondents (DISAB=1) aged 15-64 who answered D17B=1
Source: Participation and Activity Limitation Survey, 2001

Variable Name:	**D17C**	*Position:*	688	*Length:*	2
Collection Name:	AD17C				

Did you (.....) need a computer with Braille, large print or speech access?

		FREQ	WTD
1	Yes	54	4,523
3	No	300	29,218
93	Not applicable	19,862	3,303,615
98	Not stated	491	82,694
R	Refusal	0	0
X	Don't know	3	287
		======	=========
		20,710	3,420,337

Coverage: Respondents (DISAB=1) aged 15-64 who answered D16=1
Source: Participation and Activity Limitation Survey, 2001

Variable Name: **D17AC** *Position:* 690 *Length:* 2
Collection Name: AD17AC

Was it made available to you (.....)?

		FREQ	WTD
1	Yes	40	2,992
3	No	14	1,531
93	Not applicable	20,165	3,333,120
98	Not stated	491	82,694
R	Refusal	0	0
X	Don't know	0	0
		======	=========
		20,710	3,420,337

Coverage: Respondents (DISAB=1) aged 15-64 who answered D17C=1
Source: Participation and Activity Limitation Survey, 2001

Variable Name: **D17D** *Position:* 692 *Length:* 2
Collection Name: AD17D

Did you (.....) need talking books?

		FREQ	WTD
1	Yes	46	5,086
3	No	307	28,430
93	Not applicable	19,862	3,303,615
98	Not stated	493	82,940
R	Refusal	0	0
X	Don't know	2	266
		======	=========
		20,710	3,420,337

Coverage: Respondents (DISAB=1) aged 15-64 who answered D16=1
Source: Participation and Activity Limitation Survey, 2001

Variable Name: **D17AD** *Position:* 694 *Length:* 2
Collection Name: AD17AD

Was it made available to you (.....)?

		FREQ	WTD
1	Yes	30	2,305
3	No	14	2,623
93	Not applicable	20,171	3,332,311
98	Not stated	495	83,098
R	Refusal	0	0
X	Don't know	0	0
		======	=========
		20,710	3,420,337

Coverage: Respondents (DISAB=1) aged 15-64 who answered D17D=1
Source: Participation and Activity Limitation Survey, 2001

Variable Name: **D17E** *Position:* 696 *Length:* 2
Collection Name: AD17E

Did you (.....) need magnifiers or CCTV's (Closed circuit television readers)?

		FREQ	WTD
1	Yes	34	3,116
3	No	322	30,653
93	Not applicable	19,862	3,303,615
98	Not stated	490	82,780
R	Refusal	0	0
X	Don't know	2	174
		======	=========
		20,710	3,420,338

Coverage: Respondents (DISAB=1) aged 15-64 who answered D16=1
Source: Participation and Activity Limitation Survey, 2001

Variable Name: **D17AE** *Position:* 698 *Length:* 2
Collection Name: AD17AE

Was it made available to you (.....)?

		FREQ	WTD
1	Yes	25	2,220
3	No	5	739
93	Not applicable	20,186	3,334,442
98	Not stated	493	82,846
R	Refusal	0	0
X	Don't know	1	91
		======	=========
		20,710	3,420,338

Coverage: Respondents (DISAB=1) aged 15-64 who answered D17E=1
Source: Participation and Activity Limitation Survey, 2001

Variable Name: **D17F** *Position:* 700 *Length:* 2
Collection Name: AD17F

Did you (.....) need Braille or large print reading materials?

		FREQ	WTD
1	Yes	35	4,090
3	No	321	29,630
93	Not applicable	19,862	3,303,615
98	Not stated	491	82,978
R	Refusal	0	0
X	Don't know	1	25
		======	=========
		20,710	3,420,338

Coverage: Respondents (DISAB=1) aged 15-64 who answered D16=1
Source: Participation and Activity Limitation Survey, 2001

Variable Name: **D17AF** *Position:* 702 *Length:* 2
Collection Name: AD17AF

Was it made available to you (.....)?

		FREQ	WTD
1	Yes	28	2,295
3	No	5	1,755
93	Not applicable	20,184	3,333,270
98	Not stated	493	83,018
R	Refusal	0	0
X	Don't know	0	0
		======	=========
		20,710	3,420,338

Coverage: Respondents (DISAB=1) aged 15-64 who answered D17F=1
Source: Participation and Activity Limitation Survey, 2001

Variable Name: **D17G** *Position:* 704 *Length:* 2
Collection Name: AD17G

Did you (.....) need a Sign language interpreter?

		FREQ	WTD
1	Yes	19	1,557
3	No	337	32,274
93	Not applicable	19,862	3,303,615
98	Not stated	491	82,864
R	Refusal	0	0
X	Don't know	1	28
		======	=========
		20,710	3,420,338

Coverage: Respondents (DISAB=1) aged 15-64 who answered D16=1
Source: Participation and Activity Limitation Survey, 2001

Variable Name: **D17AG** *Position:* 706 *Length:* 2
Collection Name: AD17AG

Was it made available to you (.....)?

		FREQ	WTD
1	Yes	13	1,380
3	No	3	111
93	Not applicable	20,200	3,335,917
98	Not stated	494	82,930
R	Refusal	0	0
X	Don't know	0	0
		======	=========
		20,710	3,420,338

Coverage: Respondents (DISAB=1) aged 15-64 who answered D17G=1
Source: Participation and Activity Limitation Survey, 2001

Variable Name: **D17H** *Position:* 708 *Length:* 2
Collection Name: AD17H

Did you (.....) need recording equipment or portable note-takers?

		FREQ	WTD
1	Yes	83	8,559
3	No	270	24,950
93	Not applicable	19,862	3,303,615
98	Not stated	492	82,921
R	Refusal	0	0
X	Don't know	3	292
		======	=========
		20,710	3,420,337

Coverage: Respondents (DISAB=1) aged 15-64 who answered D16=1
Source: Participation and Activity Limitation Survey, 2001

Variable Name: **D17AH** *Position:* 710 *Length:* 2
Collection Name: AD17AH

Was it made available to you (.....)?

		FREQ	WTD
1	Yes	50	4,508
3	No	29	3,650
93	Not applicable	20,135	3,328,858
98	Not stated	496	83,321
R	Refusal	0	0
X	Don't know	0	0
		======	=========
		20,710	3,420,337

Coverage: Respondents (DISAB=1) aged 15-64 who answered D17H=1
Source: Participation and Activity Limitation Survey, 2001

Variable Name: **D17I** *Position:* 712 *Length:* 2
Collection Name: AD17I

Did you (.....) need attendant care services?

		FREQ	WTD
1	Yes	47	3,935
3	No	309	30,091
93	Not applicable	19,862	3,303,615
98	Not stated	491	82,671
R	Refusal	0	0
X	Don't know	1	25
		======	=========
		20,710	3,420,337

Coverage: Respondents (DISAB=1) aged 15-64 who answered D16=1
Source: Participation and Activity Limitation Survey, 2001

Variable Name: **D17AI** *Position:* 714 *Length:* 2
Collection Name: AD17AI

Was it made available to you (.....)?

		FREQ	WTD
1	Yes	33	1,990
3	No	11	1,514
93	Not applicable	20,172	3,333,731
98	Not stated	494	83,102
R	Refusal	0	0
X	Don't know	0	0
		======	=========
		20,710	3,420,337

Coverage: Respondents (DISAB=1) aged 15-64 who answered D17I=1
Source: Participation and Activity Limitation Survey, 2001

Variable Name: **D17J** *Position:* 716 *Length:* 2
Collection Name: AD17J

Did you (.....) need other aid or service?

		FREQ	WTD
1	Yes	83	12,939
3	No	273	20,373
93	Not applicable	19,862	3,303,615
98	Not stated	489	82,941
R	Refusal	0	0
X	Don't know	3	469
		======	=========
		20,710	3,420,337

Coverage: Respondents (DISAB=1) aged 15-64 who answered D16=1
Source: Participation and Activity Limitation Survey, 2001

Variable Name: **D17AJ** *Position:* 718 *Length:* 2
Collection Name: AD17AJ

Was it made available to you (.....)?

		FREQ	WTD
1	Yes	47	4,260
3	No	19	7,082
93	Not applicable	20,138	3,324,457
98	Not stated	506	84,539
R	Refusal	0	0
X	Don't know	0	0
		======	=========
		20,710	3,420,338

Coverage: Respondents (DISAB=1) aged 15-64 who answered D17J=1
Source: Participation and Activity Limitation Survey, 2001

Variable Name:	**D18**	*Position:*	720	*Length:*	2
Collection Name:	AD18				

Did you (.....) have your (his / her) condition before completing all your (his / her) formal education or training?

		FREQ	WTD
1	Yes	4,348	599,139
3	No	5,947	1,127,675
93	Not applicable	9,818	1,595,309
98	Not stated	539	88,488
R	Refusal	3	264
X	Don't know	55	9,462
		======	=========
		20,710	3,420,337

Coverage: Respondents (DISAB=1) aged 15-64 who DID NOT answer D1=1
Source: Participation and Activity Limitation Survey, 2001

Variable Name:	**D19**	*Position:*	722	*Length:*	2
Collection Name:	AD19				

Did you (.....) discontinue your (his / her) formal education or training because of your (his / her) condition?

		FREQ	WTD
1	Yes	1,266	174,005
3	No	2,587	349,577
93	Not applicable	16,084	2,777,724
98	Not stated	742	113,761
R	Refusal	0	0
X	Don't know	31	5,271
		======	=========
		20,710	3,420,338

Coverage: Respondents (DISAB=1) aged 15-64 who answered D18=1
Source: Participation and Activity Limitation Survey, 2001

Variable Name:	**D21A**	*Position:*	724	*Length:*	2
Collection Name:	AD21A				

Because of your (..... 's) condition did you (he / she) begin school later than most other people your (his / her) age?

		FREQ	WTD
1	Yes	587	66,402
3	No	5,510	628,476
93	Not applicable	13,801	2,589,246
98	Not stated	692	123,025
R	Refusal	1	22
X	Don't know	119	13,167
		======	=========
		20,710	3,420,338

Coverage: Respondents (DISAB=1) aged 15-64 who answered D1=1 or D13=1 or D18=1
Source: Participation and Activity Limitation Survey, 2001

Variable Name:	**D21B**	*Position:*	726	*Length:*	2
Collection Name:	AD21B				

Because of your (..... 's) condition, was your (his / her) education interrupted for long periods of time?

		FREQ	WTD
1	Yes	1,637	181,453
3	No	4,489	516,393
93	Not applicable	13,801	2,589,246
98	Not stated	694	121,402
R	Refusal	1	22
X	Don't know	88	11,822
		======	=========
		20,710	3,420,338

Coverage: Respondents (DISAB=1) aged 15-64 who answered D1=1 or D13=1 or D18=1
Source: Participation and Activity Limitation Survey, 2001

Variable Name:	**D21C**	*Position:*	728	*Length:*	2
Collection Name:	AD21C				

Because of your (..... 's) condition, did you (he / she) ever attend a special education school or special education classes in a regular school?

		FREQ	WTD
1	Yes	1,477	132,942
3	No	4,665	569,849
93	Not applicable	13,801	2,589,246
98	Not stated	697	120,266
R	Refusal	1	22
X	Don't know	69	8,013
		======	=========
		20,710	3,420,338

Coverage: Respondents (DISAB=1) aged 15-64 who answered D1=1 or D13=1 or D18=1
Source: Participation and Activity Limitation Survey, 2001

Variable Name:	**D21D**	*Position:*	730	*Length:*	2
Collection Name:	AD21D				

Because of your (..... 's) condition, did you (he / she) take fewer courses or subjects than you (he / she) otherwise would have?

		FREQ	WTD
1	Yes	1,705	171,189
3	No	4,399	525,233
93	Not applicable	13,801	2,589,246
98	Not stated	689	119,362
R	Refusal	1	22
X	Don't know	115	15,285
		======	=========
		20,710	3,420,337

Coverage: Respondents (DISAB=1) aged 15-64 who answered D1=1 or D13=1 or D18=1
Source: Participation and Activity Limitation Survey, 2001

Variable Name: **D21E** *Position:* 732 *Length:* 2
Collection Name: AD21E

Because of your (..... 's) condition, did you (he / she) take any courses by correspondence or home study?

		FREQ	WTD
1	Yes	754	88,136
3	No	5,382	613,834
93	Not applicable	13,801	2,589,246
98	Not stated	696	120,204
R	Refusal	1	22
X	Don't know	76	8,897
		======	=========
		20,710	3,420,339

Coverage: Respondents (DISAB=1) aged 15-64 who answered D1=1 or D13=1 or D18=1
Source: Participation and Activity Limitation Survey, 2001

Variable Name: **D21F** *Position:* 734 *Length:* 2
Collection Name: AD21F

Because of your (..... 's) condition, did you (he / she) have to leave your (his / her) community to attend school?

		FREQ	WTD
1	Yes	760	70,754
3	No	5,385	632,381
93	Not applicable	13,801	2,589,246
98	Not stated	695	119,587
R	Refusal	1	22
X	Don't know	68	8,347
		======	=========
		20,710	3,420,337

Coverage: Respondents (DISAB=1) aged 15-64 who answered D1=1 or D13=1 or D18=1
Source: Participation and Activity Limitation Survey, 2001

Variable Name:	**D21G**	*Position:*	736	*Length:*	2
Collection Name:	AD21G				

Because of your (..... 's) condition, did it take you (him / her) longer to achieve your (his / her) present level of education?

		FREQ	WTD
1	Yes	1,920	194,388
3	No	4,158	497,713
93	Not applicable	13,801	2,589,246
98	Not stated	693	121,344
R	Refusal	2	51
X	Don't know	136	17,595
		======	=========
		20,710	3,420,337

Coverage: Respondents (DISAB=1) aged 15-64 who answered D1=1 or D13=1 or D18=1
Source: Participation and Activity Limitation Survey, 2001

Section: **EMPLOYMENT**

Variable Name:	**LFSTAT**	*Position:*	738	*Length:*	1
Collection Name:	LFSTAT				

Labour force status

		FREQ	WTD
0	Not Specified	526	88,715
1	Employed	5,627	817,299
2	Unemployed	854	97,848
3	Not in Labour Force	5,907	964,632
9	Not applicable	7,796	1,451,844
		======	=========
		20,710	3,420,338

Coverage: Respondents aged 15-64
Source: Participation and Activity Limitation Survey, 2001 for disabled respondents (DISAB=1)
2001 Census of Population for non-disabled respondents (DISAB=0)

Note: This is a derived variable. It represents the respondent's Labour Force Status and is derived from Section E of the PALS questionnaire, for persons with disabilities aged 15 - 64 years. For persons without disabilities, aged 15 - 64 years, their labour force status is taken from the 1991 Census of Population. There is no labour force status for persons 65 years and over. Users should note that the reference week is the week preceding enumeration, and therefore the time frame for PALS and Census differs.

Variable Name: **E1HRS** *Position:* 739 *Length:* 3
Collection Name: E1HRS

Derived variable: Hours worked in reference week

Allowed values: 001 : 066

		FREQ	WTD
-3	Not applicable	7,796	1,451,844
-8	Not stated	704	116,166
0	0 hours	7,174	1,122,411
001 : 065	1-65 hours	4,857	705,875
66	66 hours or more	179	24,046
		======	=========
		20,710	3,420,342

Coverage: Respondents (DISAB=1) aged 15-64
Source: Participation and Activity Limitation Survey, 2001
Note: E1HRS is derived from variables E1 and E1S (write-in response). This variable represents the hours worked in the week prior to enumeration and is available only for the disabled population (DISAB = 1). For non-disabled persons (DISAB=0), the Census variable HOURS represents the hours worked in the week prior to enumeration. Possible values for E1HRS and HOURS are between 01 and 66, with value 66 including 66 or more hours worked in the reference week. Users should note that the reference week is the week preceding enumeration and therefore, the timeframe for PALS and Census differs.

Section: **EMPLOYMENT-EMPLOYED**

Variable Name: **E7HRS** *Position:* 742 *Length:* 3
Collection Name: E7HRS

Derived variable: Employed - Hours usually worked

Allowed values: 001 : 066

		FREQ	WTD
-3	Not applicable	14,557	2,514,324
-8	Not specified	721	122,128
001 : 065	1-65 hours	5,283	767,596
66	66 hours or more	149	16,288
		======	=========
		20,710	3,420,336

Coverage: Respondents (DISAB=1) aged 15-64 who were employed in reference week (LFSTAT=1)
Source: Participation and Activity Limitation Survey, 2001
Note: E7HRS is derived from variables E7 and E7S (write-in response).

Variable Name:	**E8**	*Position:*	745	*Length:*	2
Collection Name:	AE8				

What is the main reason you (.....) usually work(s) less than 30 hours per week?

		FREQ	WTD
1	Own illness, condition or disability	394	64,908
2	Caring for own children	40	5,028
3	Caring for elder relative (60 years of age or older)	3	324
4	Other personal or family responsibilities	31	4,626
5	Going to school	331	20,108
6	Business conditions	108	19,284
7	Could not find work with 30 or more hours per week	118	19,386
8	Other	223	32,540
93	Not applicable	18,685	3,123,414
98	Not stated	765	128,283
R	Refusal	1	330
X	Don't know	11	2,106
		======	=========
		20,710	3,420,337

Coverage: Respondents (DISAB=1) aged 15-64, employed in reference week (LFSTAT=1), who answered E7=Less than 30
Source: Participation and Activity Limitation Survey, 2001

Variable Name:	**E9JSTART**	*Position:*	747	*Length:*	2
Collection Name:	E9JSTART				

Derived variable: Employed - When started job

		FREQ	WTD
1	Before 1996	2,057	388,851
2	Between 1996 and 1999	1,265	173,032
3	In 2000	620	75,247
4	In 2001	1,424	141,656
93	Not applicable	14,557	2,514,324
98	Not stated	787	127,227
		======	=========
		20,710	3,420,337

Coverage: Respondents (DISAB=1) aged 15-64 who were employed in reference week (LFSTAT=1)
Source: Participation and Activity Limitation Survey, 2001
Note: E9JSTART is derived from variable E9YR.

Variable Name:	**E13**	*Position:*	749	*Length:*	2
Collection Name:	AE13				

In this job are you (is.....) mainly...

		FREQ	WTD
1	working for wages, salary, tips or commission?	4,893	684,174
2	working without pay for your (his / her) spouse or another relative in a family farm or business?	71	12,822
3	self-employed alone or in partnership?	618	116,493
93	Not applicable	14,557	2,514,324
98	Not stated	527	88,736
R	Refusal	2	122
X	Don't know	42	3,667
		======	=========
		20,710	3,420,338

Coverage: Respondents (DISAB=1) aged 15-64 who were employed in reference week (LFSTAT=1)
Source: Participation and Activity Limitation Survey, 2001

Variable Name:	**E14**	*Position:*	751	*Length:*	2
Collection Name:	AE14				

In this job, are you (is.....) a union member?

		FREQ	WTD
1	Yes	1,426	222,577
3	No	3,340	445,075
93	Not applicable	15,291	2,647,449
98	Not stated	612	101,648
R	Refusal	1	33
X	Don't know	40	3,554
		======	=========
		20,710	3,420,336

Coverage: Respondents (DISAB=1) aged 15-64, employed in reference week (LFSTAT=1), who answered E13=1
Source: Participation and Activity Limitation Survey, 2001

Variable Name:	**E15**	*Position:*	753	*Length:*	2
Collection Name:	AE15				

Are you (is.....) covered by a union contract or collective agreement?

		FREQ	WTD
1	Yes	105	14,482
3	No	3,179	425,652
93	Not applicable	16,717	2,870,027
98	Not stated	623	103,173
R	Refusal	0	0
X	Don't know	86	7,004
		======	=========
		20,710	3,420,338

Coverage: Respondents (DISAB=1) aged 15-64, employed in reference week (LFSTAT=1), who DID NOT answer E14=1
Source: Participation and Activity Limitation Survey, 2001

Variable Name:	**E16**	*Position:*	755	*Length:*	2
Collection Name:	AE16				

About how many persons are employed at the location where you (.....) now work(s)?

		FREQ	WTD
1	Less than 20	1,829	238,897
2	20 to 99	1,256	166,130
3	100 to 500	910	145,768
4	Over 500	567	83,738
93	Not applicable	15,291	2,647,449
98	Not stated	624	102,885
R	Refusal	1	24
X	Don't know	232	35,447
		======	=========
		20,710	3,420,338

Coverage: Respondents (DISAB=1) aged 15-64, employed in reference week (LFSTAT=1), who answered E13=1
Source: Participation and Activity Limitation Survey, 2001

Variable Name:	**E17**	*Position:*	757	*Length:*	2
Collection Name:	AE17				

Does your (..... 's) employer operate at more than one location?

		FREQ	WTD
1	Yes	2,882	414,744
3	No	1,729	227,661
93	Not applicable	15,291	2,647,449
98	Not stated	660	108,065
R	Refusal	1	24
X	Don't know	147	22,396
		======	=========
		20,710	3,420,339

Coverage: Respondents (DISAB=1) aged 15-64, employed in reference week (LFSTAT=1), who answered E13=1
Source: Participation and Activity Limitation Survey, 2001

Variable Name:	**E18**	*Position:*	759	*Length:*	2
Collection Name:	AE18				

In total, about how many persons are employed at all locations?

		FREQ	WTD
1	Less than 20	144	19,280
2	20 to 99	255	37,851
3	100 to 500	351	50,077
4	Over 500	1,568	215,158
93	Not applicable	17,168	2,897,529
98	Not stated	672	109,226
R	Refusal	2	166
X	Don't know	550	91,051
		======	=========
		20,710	3,420,338

Coverage: Respondents (DISAB=1) aged 15-64, employed in reference week (LFSTAT=1), who answered E17=1
Source: Participation and Activity Limitation Survey, 2001

Variable Name:	**E24**	*Position:*	761	*Length:*	2
Collection Name:	AE24				

Is your (..... 's) job a permanent job?

		FREQ	WTD
1	Yes	3,932	576,575
3	No	827	86,156
93	Not applicable	15,291	2,647,449
98	Not stated	606	100,676
R	Refusal	1	19
X	Don't know	53	9,463
		======	=========
		20,710	3,420,338

Coverage: Respondents (DISAB=1) aged 15-64, employed in reference week (LFSTAT=1), who answered E13=1
Source: Participation and Activity Limitation Survey, 2001

Variable Name:	**E25**	*Position:*	763	*Length:*	2
Collection Name:	AE25				

In what way is your (..... 's) job not permanent?

		FREQ	WTD
1	It is seasonal	137	11,563
2	Temporary, term or contract (non-seasonal)	355	40,165
3	Casual job	192	19,057
4	Work done through a temporary help agency	11	535
5	Other, specify	118	13,924
93	Not applicable	19,277	3,233,507
98	Not stated	613	101,073
R	Refusal	0	0
X	Don't know	7	515
		======	=========
		20,710	3,420,339

Coverage: Respondents (DISAB=1) aged 15-64, employed in reference week (LFSTAT=1), who answered E24=3
Source: Participation and Activity Limitation Survey, 2001

Variable Name: **E26** *Position:* 765 *Length:* 2
Collection Name: AE26

Have you (Has.....) had any periods of unemployment in the last twelve months, that is to say, periods when you were (he /she was) unemployed or did not have a job?

		FREQ	WTD
1	Yes	1,496	180,838
3	No	4,072	628,012
93	Not applicable	14,557	2,514,324
98	Not stated	566	94,878
R	Refusal	2	119
X	Don't know	17	2,166
		======	=========
		20,710	3,420,337

Coverage: Respondents (DISAB=1) aged 15-64, employed in reference week (LFSTAT=1)
Source: Participation and Activity Limitation Survey, 2001

Variable Name: **E27** *Position:* 767 *Length:* 2
Collection Name: AE27

How many different periods of unemployment did you (.....) have?

		FREQ	WTD
1	One	1,107	188,880
2	Two	225	31,508
3	Three or more	179	31,089
93	Not applicable	18,584	3,059,989
98	Not stated	589	105,774
R	Refusal	2	268
X	Don't know	24	2,829
		======	=========
		20,710	3,420,337

Coverage: Respondents (DISAB=1) aged 15-64, employed in reference week (LFSTAT=1), who answered E26=1
Source: Participation and Activity Limitation Survey, 2001

Variable Name:	**E28**	*Position:*	769	*Length:*	2
Collection Name:	AE28				

What was the length of the longest period of unemployment?

		FREQ	WTD
1	Under three months	563	70,836
2	Three to five months	328	40,579
3	Six months or more	548	61,320
93	Not applicable	18,671	3,146,596
98	Not stated	586	99,383
R	Refusal	0	0
X	Don't know	14	1,623
		======	=========
		20,710	3,420,337

Coverage: Respondents (DISAB=1) aged 15-64, employed in reference week (LFSTAT=1), who answered E27=1,2 or 3
Source: Participation and Activity Limitation Survey, 2001

Variable Name:	**E29A**	*Position:*	771	*Length:*	2
Collection Name:	AE29A				

Because of your (..... 's) condition, have you (has.....) ever changed the kind of work you do (he / she does)?

		FREQ	WTD
1	Yes	1,807	273,660
3	No	3,685	525,889
93	Not applicable	14,557	2,514,324
98	Not stated	638	103,747
R	Refusal	1	100
X	Don't know	22	2,618
		======	=========
		20,710	3,420,338

Coverage: Respondents (DISAB=1) aged 15-64, employed in reference week (LFSTAT=1)
Source: Participation and Activity Limitation Survey, 2001

Variable Name:	**E29B**	*Position:*	773	*Length:*	2
Collection Name:	AE29B				

Because of your (..... 's) condition, have you (has.....) ever changed the amount of work you do (he / she does)?

		FREQ	WTD
1	Yes	2,219	350,387
3	No	3,274	448,348
93	Not applicable	14,557	2,514,324
98	Not stated	633	102,975
R	Refusal	1	100
X	Don't know	26	4,203
		======	=========
		20,710	3,420,337

Coverage: Respondents (DISAB=1) aged 15-64, employed in reference week (LFSTAT=1)
Source: Participation and Activity Limitation Survey, 2001

Variable Name:	**E29C**	*Position:*	775	*Length:*	2
Collection Name:	AE29C				

Because of your (..... 's) condition, have you (has.....) ever changed your (his / her) job?

		FREQ	WTD
1	Yes	1,594	230,050
3	No	3,896	568,096
93	Not applicable	14,557	2,514,324
98	Not stated	642	104,057
R	Refusal	1	100
X	Don't know	20	3,711
		======	=========
		20,710	3,420,338

Coverage: Respondents (DISAB=1) aged 15-64, employed in reference week (LFSTAT=1)
Source: Participation and Activity Limitation Survey, 2001

Variable Name:	**E30**	*Position:*	777	*Length:*	2
Collection Name:	AE30				

Does your (..... 's) condition limit the amount or kind of work you (he / she) can do at your (his / her) present job or business?

		FREQ	WTD
1	Yes	2,746	420,015
3	No	2,818	391,040
93	Not applicable	14,557	2,514,324
98	Not stated	555	92,425
R	Refusal	1	22
X	Don't know	33	2,513
		======	=========
		20,710	3,420,339

Coverage: Respondents (DISAB=1) aged 15-64, employed in reference week (LFSTAT=1)
Source: Participation and Activity Limitation Survey, 2001

Variable Name:	**E31**	*Position:*	779	*Length:*	2
Collection Name:	AE31				

Where were you (was.....) employed when you (he / she) first experienced work limitations?

		FREQ	WTD
1	Present employer	1,124	200,837
2	Elsewhere	1,016	190,358
3	Not working	453	77,488
93	Not applicable	17,435	2,831,942
98	Not stated	646	113,428
R	Refusal	4	449
X	Don't know	32	5,836
		======	=========
		20,710	3,420,338

Coverage: Respondents (DISAB=1) aged 15-64, employed in reference week (LFSTAT=1), who answered E30=1
Source: Participation and Activity Limitation Survey, 2001

Variable Name: **E32** *Position:* 781 *Length:* 2
Collection Name: AE32

Are you (Is.....) now doing the same kind of work as you were (he / she was) doing at the time you (he / she) first experienced work limitations?

		FREQ	WTD
1	Yes	1,011	167,131
3	No	1,168	182,035
93	Not applicable	17,871	2,963,414
98	Not stated	637	103,267
R	Refusal	0	0
X	Don't know	23	4,492
		======	=========
		20,710	3,420,339

Coverage: Respondents (DISAB=1) aged 15-64, employed in reference week (LFSTAT=1), who answered E31=1 or 2
Source: Participation and Activity Limitation Survey, 2001

Variable Name: **E33** *Position:* 783 *Length:* 2
Collection Name: AE33

Is your (..... 's) condition the reason you are (he / she is) now doing a different kind of work?

		FREQ	WTD
1	Yes	877	144,913
3	No	278	35,334
93	Not applicable	18,905	3,135,037
98	Not stated	642	103,711
R	Refusal	0	0
X	Don't know	8	1,343
		======	=========
		20,710	3,420,338

Coverage: Respondents (DISAB=1) aged 15-64, employed in reference week (LFSTAT=1), who answered E32=3
Source: Participation and Activity Limitation Survey, 2001

Variable Name:	**E34**	*Position:*	785	*Length:*	2
Collection Name:	AE34				

Because of your (..... 's) condition, would you say that you are (he / she is) now doing:

		FREQ	WTD
1	about the same amount of work?	707	103,351
2	more work now?	232	36,273
3	less work now?	1,206	205,296
93	Not applicable	17,871	2,963,414
98	Not stated	636	102,876
R	Refusal	0	0
X	Don't know	58	9,128
		======	=========
		20,710	3,420,338

Coverage: Respondents (DISAB=1) aged 15-64, employed in reference week (LFSTAT=1), who answered E31=1 or 2
Source: Participation and Activity Limitation Survey, 2001

Variable Name:	**E35**	*Position:*	787	*Length:*	2
Collection Name:	AE35				

Is your (..... 's) condition the reason you are (he / she is) doing less?

		FREQ	WTD
1	Yes	1,124	191,330
3	No	59	9,685
93	Not applicable	18,868	3,112,166
98	Not stated	657	106,451
R	Refusal	0	0
X	Don't know	2	706
		======	=========
		20,710	3,420,338

Coverage: Respondents (DISAB=1) aged 15-64, employed in reference week (LFSTAT=1), who answered E34=3
Source: Participation and Activity Limitation Survey, 2001

Variable Name: **E36** *Position:* 789 *Length:* 2
Collection Name: AE36

Do you (Does.....) believe that your (his / her) condition makes it difficult for you (him/ her) to change jobs or to advance at your (his / her) present job?

		FREQ	WTD
1	Yes, very difficult	876	142,776
2	Yes, difficult	1,097	157,002
3	No, not difficult	2,619	337,764
93	Not applicable	15,291	2,647,449
98	Not stated	719	115,647
R	Refusal	1	56
X	Don't know	107	19,644
		======	=========
		20,710	3,420,338

Coverage: Respondents (DISAB=1) aged 15-64, employed in reference week (LFSTAT=1), who answered E13=1
Source: Participation and Activity Limitation Survey, 2001

Variable Name: **E37** *Position:* 791 *Length:* 2
Collection Name: AE37

Does your (..... 's) job give you (him/ her) the opportunity to use all your (his / her) education, skills or work experience?

		FREQ	WTD
1	Yes	3,099	454,032
3	No	1,557	196,773
93	Not applicable	15,291	2,647,449
98	Not stated	707	112,998
R	Refusal	1	56
X	Don't know	55	9,030
		======	=========
		20,710	3,420,338

Coverage: Respondents (DISAB=1) aged 15-64, employed in reference week (LFSTAT=1), who answered E13=1
Source: Participation and Activity Limitation Survey, 2001

Variable Name: **E38** *Position:* 793 *Length:* 2
Collection Name: AE38

Does your (..... 's) job require the level of education you have (he / she has)?

		FREQ	WTD
1	Yes	2,914	424,958
3	No	1,707	218,508
93	Not applicable	15,291	2,647,449
98	Not stated	712	113,823
R	Refusal	1	56
X	Don't know	85	15,544
		======	=========
		20,710	3,420,338

Coverage: Respondents (DISAB=1) aged 15-64, employed in reference week (LFSTAT=1), who answered E13=1
Source: Participation and Activity Limitation Survey, 2001

Variable Name: **E39A** *Position:* 795 *Length:* 2
Collection Name: AE39A

Because of your (his / her) condition, do you (does.....) require any of the following to be able to work; Job redesign (modified or different duties)?

		FREQ	WTD
1	Yes	961	147,709
3	No	3,760	512,711
93	Not applicable	15,291	2,647,449
98	Not stated	636	101,826
R	Refusal	2	352
X	Don't know	60	10,290
		======	=========
		20,710	3,420,337

Coverage: Respondents (DISAB=1) aged 15-64, employed in reference week (LFSTAT=1), who answered E13=1
Source: Participation and Activity Limitation Survey, 2001

Variable Name:	**E39AA**	*Position:*	797	*Length:*	2
Collection Name:	AE39AA				

Has job redesign required been made available to you (him/ her)?

		FREQ	WTD
1	Yes	706	110,207
3	No	189	28,009
93	Not applicable	19,113	3,170,803
98	Not stated	674	107,606
R	Refusal	0	0
X	Don't know	28	3,713
		======	=========
		20,710	3,420,338

Coverage: Respondents (DISAB=1) aged 15-64, employed in reference week (LFSTAT=1), who answered E39A=1
Source: Participation and Activity Limitation Survey, 2001

Variable Name:	**E39B**	*Position:*	799	*Length:*	2
Collection Name:	AE39B				

Because of your (his / her) condition, do you (does.....) require any of the following to be able to work; Modified hours or days or reduced work hours?

		FREQ	WTD
1	Yes	1,073	160,489
3	No	3,663	501,744
93	Not applicable	15,291	2,647,449
98	Not stated	634	103,010
R	Refusal	1	21
X	Don't know	48	7,624
		======	=========
		20,710	3,420,337

Coverage: Respondents (DISAB=1) aged 15-64, employed in reference week (LFSTAT=1), who answered E13=1
Source: Participation and Activity Limitation Survey, 2001

Variable Name:	**E39AB**	*Position:*	801	*Length:*	2
Collection Name:	AE39AB				

Has modified hours required to work been provided to you (him/ her)?

		FREQ	WTD
1	Yes	840	126,144
3	No	176	26,276
93	Not applicable	19,003	3,156,838
98	Not stated	671	108,503
R	Refusal	0	0
X	Don't know	20	2,576
		======	=========
		20,710	3,420,337

Coverage: Respondents (DISAB=1) aged 15-64, employed in reference week (LFSTAT=1), who answered E39B=1
Source: Participation and Activity Limitation Survey, 2001

Variable Name:	**E39C**	*Position:*	803	*Length:*	2
Collection Name:	AE39C				

Because of your (his / her) condition, do you (does.....) require any of the following to be able to work; Human support, such as a reader, Sign language interpreter, job coach or personal assistant?

		FREQ	WTD
1	Yes	228	24,122
3	No	4,526	641,861
93	Not applicable	15,291	2,647,449
98	Not stated	631	101,980
R	Refusal	3	376
X	Don't know	31	4,549
		======	=========
		20,710	3,420,337

Coverage: Respondents (DISAB=1) aged 15-64, employed in reference week (LFSTAT=1), who answered E13=1
Source: Participation and Activity Limitation Survey, 2001

Variable Name: **E39AC** *Position:* 805 *Length:* 2
Collection Name: AE39AC

Has human support required to work been provided to you (him/ her)?

		FREQ	WTD
1	Yes	185	18,477
3	No	30	3,684
93	Not applicable	19,851	3,294,235
98	Not stated	642	103,857
R	Refusal	0	0
X	Don't know	2	84
		======	=========
		20,710	3,420,337

Coverage: Respondents (DISAB=1) aged 15-64, employed in reference week (LFSTAT=1), who answered E39C=1
Source: Participation and Activity Limitation Survey, 2001

Variable Name: **E39D** *Position:* 807 *Length:* 2
Collection Name: AE39D

Because of your (his / her) condition, do you (does.....) require any of the following to be able to work: Technical aids, such as a voice synthesizer, a TTY or TDD, an infrared system or portable note-takers?

		FREQ	WTD
1	Yes	93	14,045
3	No	4,657	651,961
93	Not applicable	15,291	2,647,449
98	Not stated	635	102,171
R	Refusal	1	323
X	Don't know	33	4,388
		======	=========
		20,710	3,420,337

Coverage: Respondents (DISAB=1) aged 15-64, employed in reference week (LFSTAT=1), who answered E13=1
Source: Participation and Activity Limitation Survey, 2001

Variable Name: **E39AD** *Position:* 809 *Length:* 2
Collection Name: AE39AD

Has technical aids required to work been provided to you (him/ her)?

		FREQ	WTD
1	Yes	54	9,307
3	No	31	3,467
93	Not applicable	19,982	3,304,122
98	Not stated	643	103,442
R	Refusal	0	0
X	Don't know	0	0
		======	=========
		20,710	3,420,338

Coverage: Respondents (DISAB=1) aged 15-64, employed in reference week (LFSTAT=1), who answered E39D=1
Source: Participation and Activity Limitation Survey, 2001

Variable Name: **E39E** *Position:* 811 *Length:* 2
Collection Name: AE39E

Because of your (his / her) condition, do you (does.....) require any of the following to be able to work: A computer with Braille, large print or speech access, or a scanner?

		FREQ	WTD
1	Yes	67	7,924
3	No	4,687	658,635
93	Not applicable	15,291	2,647,449
98	Not stated	635	102,247
R	Refusal	2	356
X	Don't know	28	3,727
		======	=========
		20,710	3,420,338

Coverage: Respondents (DISAB=1) aged 15-64, employed in reference week (LFSTAT=1), who answered E13=1
Source: Participation and Activity Limitation Survey, 2001

Variable Name:	**E39AE**	*Position:*	813	*Length:*	2
Collection Name:	AE39AE				

Has specialized computer required to work been provided to you (him/ her)?

		FREQ	WTD
1	Yes	33	2,870
3	No	22	3,225
93	Not applicable	20,008	3,310,167
98	Not stated	643	103,793
R	Refusal	0	0
X	Don't know	4	284
		======	=========
		20,710	3,420,339

Coverage: Respondents (DISAB=1) aged 15-64, employed in reference week (LFSTAT=1), who answered E39E=1
Source: Participation and Activity Limitation Survey, 2001

Variable Name:	**E39F**	*Position:*	815	*Length:*	2
Collection Name:	AE39F				

Because of your (his / her) condition, do you (does.....) require any of the following to be able to work: Communication aids, such as Braille or large print reading material or recording equipment?

		FREQ	WTD
1	Yes	46	5,555
3	No	4,709	660,992
93	Not applicable	15,291	2,647,449
98	Not stated	634	102,058
R	Refusal	2	356
X	Don't know	28	3,927
		======	=========
		20,710	3,420,337

Coverage: Respondents (DISAB=1) aged 15-64, employed in reference week (LFSTAT=1), who answered E13=1
Source: Participation and Activity Limitation Survey, 2001

Variable Name:	**E39AF**	*Position:*	817	*Length:*	2
Collection Name:	AE39AF				

Has communication aids required to work been provided to you (him/ her)?

		FREQ	WTD
1	Yes	23	1,992
3	No	16	2,742
93	Not applicable	20,030	3,312,724
98	Not stated	639	102,766
R	Refusal	0	0
X	Don't know	2	114
		======	=========
		20,710	3,420,338

Coverage: Respondents (DISAB=1) aged 15-64, employed in reference week (LFSTAT=1), who answered E39F=1
Source: Participation and Activity Limitation Survey, 2001

Variable Name:	**E39G**	*Position:*	819	*Length:*	2
Collection Name:	AE39G				

Because of your (his / her) condition, do you (does.....) require any of the following to be able to work: Other equipment, help or work arrangement?

		FREQ	WTD
1	Yes	270	40,434
3	No	4,469	620,670
93	Not applicable	15,291	2,647,449
98	Not stated	644	105,363
R	Refusal	1	323
X	Don't know	35	6,099
		======	=========
		20,710	3,420,338

Coverage: Respondents (DISAB=1) aged 15-64, employed in reference week (LFSTAT=1), who answered E13=1
Source: Participation and Activity Limitation Survey, 2001

Variable Name:	**E39AG**	*Position:*	821	*Length:*	2
Collection Name:	AE39AG				

Has other equipment required to work been provided to you (him/ her)?

		FREQ	WTD
1	Yes	170	26,166
3	No	65	10,712
93	Not applicable	19,796	3,274,541
98	Not stated	676	108,187
R	Refusal	0	0
X	Don't know	3	732
		======	=========
		20,710	3,420,338

Coverage: Respondents (DISAB=1) aged 15-64, employed in reference week (LFSTAT=1), who answered E39G=1
Source: Participation and Activity Limitation Survey, 2001

Variable Name:	**E40A**	*Position:*	823	*Length:*	2
Collection Name:	AE40A				

Because of your (his / her) condition, do you (does.....) require any of the following to be able to work: Handrails, ramps?

		FREQ	WTD
1	Yes	219	26,949
3	No	5,338	781,033
93	Not applicable	14,557	2,514,324
98	Not stated	556	92,476
R	Refusal	2	133
X	Don't know	38	5,422
		======	=========
		20,710	3,420,337

Coverage: Respondents (DISAB=1) aged 15-64, employed in reference week (LFSTAT=1)
Source: Participation and Activity Limitation Survey, 2001

Variable Name: **E40AA** *Position:* 825 *Length:* 2
Collection Name: AE40AA

Has this been made available to you (.....): Handrails, ramps?

		FREQ	WTD
1	Yes	163	19,907
3	No	39	4,764
93	Not applicable	19,935	3,300,913
98	Not stated	567	93,674
R	Refusal	0	0
X	Don't know	6	1,080
		======	=========
		20,710	3,420,338

Coverage: Respondents (DISAB=1) aged 15-64, employed in reference week (LFSTAT=1), who answered E40A=1
Source: Participation and Activity Limitation Survey, 2001

Variable Name: **E40B** *Position:* 827 *Length:* 2
Collection Name: AE40B

Because of your (his / her) condition, do you (does.....) require any of the following to be able to work: Appropriate parking?

		FREQ	WTD
1	Yes	317	44,169
3	No	5,245	765,336
93	Not applicable	14,557	2,514,324
98	Not stated	552	91,047
R	Refusal	1	100
X	Don't know	38	5,362
		======	=========
		20,710	3,420,338

Coverage: Respondents (DISAB=1) aged 15-64, employed in reference week (LFSTAT=1)
Source: Participation and Activity Limitation Survey, 2001

Variable Name:	**E40AB**	*Position:*	829	*Length:*	2
Collection Name:	AE40AB				

Has this been made available to you (....): Appropriate parking?

		FREQ	WTD
1	Yes	230	31,268
3	No	66	8,057
93	Not applicable	19,841	3,285,122
98	Not stated	567	94,906
R	Refusal	0	0
X	Don't know	6	984
		======	=========
		20,710	3,420,337

Coverage: Respondents (DISAB=1) aged 15-64, employed in reference week (LFSTAT=1), who answered E40B=1
Source: Participation and Activity Limitation Survey, 2001

Variable Name:	**E40C**	*Position:*	831	*Length:*	2
Collection Name:	AE40C				

Because of your (his / her) condition, do you (does.....) require any of the following to be able to work:Accessible elevator?

		FREQ	WTD
1	Yes	244	36,444
3	No	5,311	771,021
93	Not applicable	14,557	2,514,324
98	Not stated	562	93,830
R	Refusal	2	133
X	Don't know	34	4,585
		======	=========
		20,710	3,420,337

Coverage: Respondents (DISAB=1) aged 15-64, employed in reference week (LFSTAT=1)
Source: Participation and Activity Limitation Survey, 2001

Variable Name:	**E40AC**	*Position:*	833	*Length:*	2
Collection Name:	AE40AC				

Has this been made available to you (....): Accessible elevator?

		FREQ	WTD
1	Yes	179	26,447
3	No	46	7,149
93	Not applicable	19,904	3,290,063
98	Not stated	574	95,577
R	Refusal	0	0
X	Don't know	7	1,101
		======	=========
		20,710	3,420,337

Coverage: Respondents (DISAB=1) aged 15-64, employed in reference week (LFSTAT=1), who answered E40C=1
Source: Participation and Activity Limitation Survey, 2001

Variable Name:	**E40D**	*Position:*	835	*Length:*	2
Collection Name:	AE40D				

Because of your (his / her) condition, do you (does.....) require any of the following to be able to work: Modified workstation?

		FREQ	WTD
1	Yes	399	59,314
3	No	5,161	749,994
93	Not applicable	14,557	2,514,324
98	Not stated	554	91,763
R	Refusal	2	133
X	Don't know	37	4,810
		======	=========
		20,710	3,420,338

Coverage: Respondents (DISAB=1) aged 15-64, employed in reference week (LFSTAT=1)
Source: Participation and Activity Limitation Survey, 2001

Variable Name:	**E40AD**	*Position:* 837	*Length:* 2
Collection Name:	AE40AD		

Has this been made available to you (....): Modified workstation?

		FREQ	WTD
1	Yes	279	44,170
3	No	90	10,882
93	Not applicable	19,757	3,269,260
98	Not stated	574	94,153
R	Refusal	0	0
X	Don't know	10	1,873
		======	=========
		20,710	3,420,338

Coverage: Respondents (DISAB=1) aged 15-64, employed in reference week (LFSTAT=1), who answered E40D=1
Source: Participation and Activity Limitation Survey, 2001

Variable Name:	**E40E**	*Position:* 839	*Length:* 2
Collection Name:	AE40E		

Because of your (his / her) condition, do you (does.....) require any of the following to be able to work: Accessible washrooms?

		FREQ	WTD
1	Yes	266	34,361
3	No	5,293	774,427
93	Not applicable	14,557	2,514,324
98	Not stated	556	91,881
R	Refusal	2	133
X	Don't know	36	5,212
		======	=========
		20,710	3,420,338

Coverage: Respondents (DISAB=1) aged 15-64, employed in reference week (LFSTAT=1)
Source: Participation and Activity Limitation Survey, 2001

Variable Name:	**E40AE**	*Position:*	841	*Length:*	2
Collection Name:	AE40AE				

Has this been made available to you (....): Accessible washrooms?

		FREQ	WTD
1	Yes	209	26,427
3	No	29	3,613
93	Not applicable	19,888	3,294,096
98	Not stated	579	95,154
R	Refusal	0	0
X	Don't know	5	1,048
		======	=========
		20,710	3,420,338

Coverage: Respondents (DISAB=1) aged 15-64, employed in reference week (LFSTAT=1), who answered E40E=1
Source: Participation and Activity Limitation Survey, 2001

Variable Name:	**E40F**	*Position:*	843	*Length:*	2
Collection Name:	AE40F				

Because of your (his / her) condition, do you (does.....) require any of the following to be able to work: Accessible transportation?

		FREQ	WTD
1	Yes	206	26,834
3	No	5,356	782,821
93	Not applicable	14,557	2,514,324
98	Not stated	553	90,993
R	Refusal	2	133
X	Don't know	36	5,233
		======	=========
		20,710	3,420,338

Coverage: Respondents (DISAB=1) aged 15-64, employed in reference week (LFSTAT=1)
Source: Participation and Activity Limitation Survey, 2001

Variable Name:	**E40AF**	*Position:*	845	*Length:*	2
Collection Name:	AE40AF				

Has this been made available to you (....): Accessible transportation?

		FREQ	WTD
1	Yes	148	17,637
3	No	37	6,168
93	Not applicable	19,951	3,302,511
98	Not stated	573	93,861
R	Refusal	0	0
X	Don't know	1	160
		======	=========
		20,710	3,420,337

Coverage: Respondents (DISAB=1) aged 15-64, employed in reference week (LFSTAT=1), who answered E40F=1
Source: Participation and Activity Limitation Survey, 2001

Variable Name:	**E40G**	*Position:*	847	*Length:*	2
Collection Name:	AE40G				

Because of your (his / her) condition, do you (does.....) require any of the following to be able to work: Other?

		FREQ	WTD
1	Yes	95	12,841
3	No	5,425	791,328
93	Not applicable	14,557	2,514,324
98	Not stated	594	95,587
R	Refusal	1	100
X	Don't know	38	6,157
		======	=========
		20,710	3,420,337

Coverage: Respondents (DISAB=1) aged 15-64, employed in reference week (LFSTAT=1)
Source: Participation and Activity Limitation Survey, 2001

Variable Name: **E40AG** *Position:* 849 *Length:* 2
Collection Name: AE40AG

Has this been made available to you (....): Other?

		FREQ	WTD
1	Yes	41	6,641
3	No	26	3,103
93	Not applicable	20,021	3,311,909
98	Not stated	617	98,251
R	Refusal	0	0
X	Don't know	5	433
		======	=========
		20,710	3,420,337

Coverage: Respondents (DISAB=1) aged 15-64, employed in reference week (LFSTAT=1), who answered E40G=1
Source: Participation and Activity Limitation Survey, 2001

Variable Name: **E41** *Position:* 851 *Length:* 2
Collection Name: AE41

In the past five years, have you (has.....) taken any work-related training courses to either improve your (his / her) skills or to learn new skills?

		FREQ	WTD
1	Yes	2,847	414,853
3	No	2,756	399,550
93	Not applicable	14,557	2,514,324
98	Not stated	539	90,167
R	Refusal	1	100
X	Don't know	10	1,344
		======	=========
		20,710	3,420,338

Coverage: Respondents (DISAB=1) aged 15-64, employed in reference week (LFSTAT=1)
Source: Participation and Activity Limitation Survey, 2001

Variable Name:	**E42**	*Position:*	853	*Length:*	2
Collection Name:	AE42				

What was the main reason you (.....) took this course? Was it...

		FREQ	WTD
1	for your (his / her) current or a future job?	2,334	343,123
2	because of your (his / her) condition?	76	11,432
3	for personal interest?	260	32,722
4	for another reason?	116	19,485
93	Not applicable	17,324	2,915,318
98	Not stated	595	97,760
R	Refusal	0	0
X	Don't know	5	496
		======	=========
		20,710	3,420,336

Coverage: Respondents (DISAB=1) aged 15-64, employed in reference week (LFSTAT=1), who answered E41=1
Source: Participation and Activity Limitation Survey, 2001

Variable Name:	**E43**	*Position:*	855	*Length:*	2
Collection Name:	AE43				

At work, to what extent are you (is he / she) using the skills or knowledge acquired in this course?

		FREQ	WTD
1	To a great extent	1,558	228,971
2	Somewhat	747	111,905
3	Very little	232	34,985
4	Not at all	270	33,442
93	Not applicable	17,324	2,915,318
98	Not stated	563	94,051
R	Refusal	0	0
X	Don't know	16	1,666
		======	=========
		20,710	3,420,338

Coverage: Respondents (DISAB=1) aged 15-64, employed in reference week (LFSTAT=1), who answered E41=1
Source: Participation and Activity Limitation Survey, 2001

Variable Name: **E44** *Position:* 857 *Length:* 2
Collection Name: AE44

Did you (.....) want to take some work-related training courses?

		FREQ	WTD
1	Yes	749	100,548
3	No	1,952	290,307
93	Not applicable	17,404	2,929,177
98	Not stated	552	93,343
R	Refusal	2	306
X	Don't know	51	6,656
		======	=========
		20,710	3,420,337

Coverage: Respondents (DISAB=1) aged 15-64, employed in reference week (LFSTAT=1), who DID NOT answer E41=1
Source: Participation and Activity Limitation Survey, 2001

Variable Name: **E45A** *Position:* 859 *Length:* 2
Collection Name: AE45A

Did any of the following prevent you (.....) from taking work-related training courses: Location was not physically accessible to you (him / her)?

		FREQ	WTD
1	Yes	93	13,659
3	No	635	83,338
93	Not applicable	19,409	3,226,447
98	Not stated	567	95,580
R	Refusal	0	0
X	Don't know	6	1,314
		======	=========
		20,710	3,420,338

Coverage: Respondents (DISAB=1) aged 15-64, employed in reference week (LFSTAT=1), who answered E44=1
Source: Participation and Activity Limitation Survey, 2001

Variable Name: **E45B** *Position:* 861 *Length:* 2
Collection Name: AE45B

Did any of the following prevent you (.....) from taking work-related training courses: Courses were not adapted to your (his / her) needs?

		FREQ	WTD
1	Yes	88	16,482
3	No	631	80,354
93	Not applicable	19,409	3,226,447
98	Not stated	570	96,023
R	Refusal	0	0
X	Don't know	12	1,032
		======	=========
		20,710	3,420,338

Coverage: Respondents (DISAB=1) aged 15-64, employed in reference week (LFSTAT=1), who answered E44=1
Source: Participation and Activity Limitation Survey, 2001

Variable Name: **E45C** *Position:* 863 *Length:* 2
Collection Name: AE45C

Did any of the following prevent you (.....) from taking work-related training courses: You (He / She) requested courses, but were denied them (by employer)?

		FREQ	WTD
1	Yes	74	8,175
3	No	654	89,336
93	Not applicable	19,409	3,226,447
98	Not stated	570	96,292
R	Refusal	0	0
X	Don't know	3	89
		======	=========
		20,710	3,420,339

Coverage: Respondents (DISAB=1) aged 15-64, employed in reference week (LFSTAT=1), who answered E44=1
Source: Participation and Activity Limitation Survey, 2001

Variable Name:	**E45D**	*Position:*	865	*Length:*	2
Collection Name:	AE45D				

Did any of the following prevent you (.....) from taking work-related training courses: Your (His / Her) condition?

		FREQ	WTD
1	Yes	185	28,893
3	No	545	69,084
93	Not applicable	19,409	3,226,447
98	Not stated	568	95,828
R	Refusal	0	0
X	Don't know	3	86
		======	=========
		20,710	3,420,338

Coverage: Respondents (DISAB=1) aged 15-64, employed in reference week (LFSTAT=1), who answered E44=1
Source: Participation and Activity Limitation Survey, 2001

Variable Name:	**E45E**	*Position:*	867	*Length:*	2
Collection Name:	AE45E				

Did any of the following prevent you (.....) from taking work-related training courses: Inadequate transportation?

		FREQ	WTD
1	Yes	74	7,066
3	No	655	90,003
93	Not applicable	19,409	3,226,447
98	Not stated	570	96,325
R	Refusal	0	0
X	Don't know	2	498
		======	=========
		20,710	3,420,339

Coverage: Respondents (DISAB=1) aged 15-64, employed in reference week (LFSTAT=1), who answered E44=1
Source: Participation and Activity Limitation Survey, 2001

Variable Name: **E45F** *Position:* 869 *Length:* 2
Collection Name: AE45F

Did any of the following prevent you (.....) from taking work-related training courses: Too costly?

		FREQ	WTD
1	Yes	370	46,160
3	No	359	51,539
93	Not applicable	19,409	3,226,447
98	Not stated	566	95,652
R	Refusal	0	0
X	Don't know	6	541
		======	=========
		20,710	3,420,339

Coverage: Respondents (DISAB=1) aged 15-64, employed in reference week (LFSTAT=1), who answered E44=1
Source: Participation and Activity Limitation Survey, 2001

Variable Name: **E45G** *Position:* 871 *Length:* 2
Collection Name: AE45G

Did any of the following prevent you (.....) from taking work-related training courses: Other reason?

		FREQ	WTD
1	Yes	218	31,018
3	No	511	65,714
93	Not applicable	19,409	3,226,447
98	Not stated	567	96,281
R	Refusal	0	0
X	Don't know	5	879
		======	=========
		20,710	3,420,339

Coverage: Respondents (DISAB=1) aged 15-64, employed in reference week (LFSTAT=1), who answered E44=1
Source: Participation and Activity Limitation Survey, 2001

Variable Name: **E46A** *Position:* 873 *Length:* 2
Collection Name: AE46A

In the past five years, do you (does.....) believe that because of your (his / her) condition, you have (he / she has) been refused for employment?

		FREQ	WTD
1	Yes	640	86,871
3	No	4,789	694,653
93	Not applicable	14,557	2,514,324
98	Not stated	608	102,272
R	Refusal	6	1,148
X	Don't know	110	21,070
		======	=========
		20,710	3,420,338

Coverage: Respondents (DISAB=1) aged 15-64, employed in reference week (LFSTAT=1)
Source: Participation and Activity Limitation Survey, 2001

Variable Name: **E46B** *Position:* 875 *Length:* 2
Collection Name: AE46B

In the past five years, do you (does.....) believe that because of your (his / her) condition, you have (he / she has) been refused for a promotion?

		FREQ	WTD
1	Yes	348	48,235
3	No	5,058	732,277
93	Not applicable	14,557	2,514,324
98	Not stated	591	97,211
R	Refusal	5	1,409
X	Don't know	151	26,882
		======	=========
		20,710	3,420,338

Coverage: Respondents (DISAB=1) aged 15-64, employed in reference week (LFSTAT=1)
Source: Participation and Activity Limitation Survey, 2001

Variable Name:	**E46C**	*Position:*	877	*Length:*	2
Collection Name:	AE46C				

In the past five years, do you (does.....) believe that because of your (his / her) condition, you have (he / she has) refused for access to training programs?

		FREQ	WTD
1	Yes	159	22,583
3	No	5,276	763,364
93	Not applicable	14,557	2,514,324
98	Not stated	589	96,312
R	Refusal	4	1,047
X	Don't know	125	22,707
		======	=========
		20,710	3,420,337

Coverage: Respondents (DISAB=1) aged 15-64, employed in reference week (LFSTAT=1)
Source: Participation and Activity Limitation Survey, 2001

Variable Name:	**E46D**	*Position:*	879	*Length:*	2
Collection Name:	AE46D				

In the past five years, do you (does.....) believe that because of your (his / her) condition, you have (he / she has) refused or, has your (his / her) employment been terminated?

		FREQ	WTD
1	Yes	397	54,003
3	No	5,054	735,639
93	Not applicable	14,557	2,514,324
98	Not stated	588	97,831
R	Refusal	8	1,441
X	Don't know	106	17,100
		======	=========
		20,710	3,420,338

Coverage: Respondents (DISAB=1) aged 15-64, employed in reference week (LFSTAT=1)
Source: Participation and Activity Limitation Survey, 2001

Variable Name:	**E47**	*Position:*	881	*Length:*	2
Collection Name:	AE47				

Do you (Does.....) consider yourself (himself / herself) to be disadvantaged in employment because of your (his / her) condition?

		FREQ	WTD
1	Yes	1,937	279,801
3	No	3,454	494,829
93	Not applicable	14,557	2,514,324
98	Not stated	698	122,315
R	Refusal	1	100
X	Don't know	63	8,969
		======	=========
		20,710	3,420,338

Coverage: Respondents (DISAB=1) aged 15-64, employed in reference week (LFSTAT=1)
Source: Participation and Activity Limitation Survey, 2001

Variable Name:	**E48**	*Position:*	883	*Length:*	2
Collection Name:	AE48				

Do you (Does.....) believe that your (his / her) current employer or any potential employer would be likely to consider you (him/ her) disadvantaged in employment because of your (his / her) condition?

		FREQ	WTD
1	Yes	1,935	289,260
3	No	3,378	481,895
93	Not applicable	14,557	2,514,324
98	Not stated	609	100,619
R	Refusal	1	100
X	Don't know	230	34,140
		======	=========
		20,710	3,420,338

Coverage: Respondents (DISAB=1) aged 15-64, employed in reference week (LFSTAT=1)
Source: Participation and Activity Limitation Survey, 2001

Section: **EMPLOYMENT-UNEMPLOYED**

Variable Name: **E49_LSTWK** *Position:* 885 *Length:* 2
Collection Name: E49_LSTWK

Derived variable: Unemployed - When last worked

		FREQ	WTD
1	Before 2000	129	18,873
2	In 2000	124	15,314
3	In 2001	544	58,790
4	Never worked	47	3,863
93	Not applicable	19,330	3,233,775
98	Not stated	536	89,723
		======	=========
		20,710	3,420,338

Coverage: Respondents (DISAB=1) aged 15-64, who were unemployed in reference week (LFSTAT=2)
Source: Participation and Activity Limitation Survey, 2001
Note: E49_LSTWK is derived from variables E49 and E49S (write-in response). This variable is available only for disabled persons (DISAB=1).

Variable Name: **E50HRS** *Position:* 887 *Length:* 3
Collection Name: E50HRS

Derived variable: Unemployed - Hours usually worked when last worked

Allowed values: 001 : 066

		FREQ	WTD
-3	Not applicable	19,409	3,243,205
-8	Not specified	576	96,213
002 : 065	1-65 hours	699	78,897
66	66 hours or more	26	2,022
		======	=========
		20,710	3,420,337

Coverage: Respondents (DISAB=1) aged 15-64, unemployed in reference week (LFSTAT=2), who answered E49 equal to or greater than 1996
Source: Participation and Activity Limitation Survey, 2001
Note: E50HRS is derived from variables E50 and E50S (write-in response).

Variable Name: **E54** *Position:* 890 *Length:* 2
Collection Name: AE54

In that job, were you (was.....) mainly...

		FREQ	WTD
1	working for wages, salary, tips or commission?	711	80,655
2	working without pay for spouse or another relative in a family farm or business?	13	2,016
3	self-employed alone or in partnership?	32	4,182
93	Not applicable	19,409	3,243,205
98	Not stated	541	90,126
R	Refusal	0	0
X	Don't know	4	153
		======	=========
		20,710	3,420,337

Coverage: Respondents (DISAB=1) aged 15-64, unemployed in reference week (LFSTAT=2), who answered E49 equal to or greater than 1996
Source: Participation and Activity Limitation Survey, 2001

Variable Name: **E55** *Position:* 892 *Length:* 2
Collection Name: AE55

Have you (Has.....) had any periods of employment in the last twelve months; that is to say, periods when you (he / she) had a job?

		FREQ	WTD
1	Yes	467	54,580
3	No	131	15,437
93	Not applicable	19,516	3,257,519
98	Not stated	596	92,802
R	Refusal	0	0
X	Don't know	0	0
		======	=========
		20,710	3,420,338

Coverage: Respondents (DISAB=1) aged 15-64, unemployed in reference week (LFSTAT=2), who answered E49 equal to or greater than 2000
Source: Participation and Activity Limitation Survey, 2001

Variable Name:	**E56**	*Position:*	894	*Length:*	2
Collection Name:	AE56				

How many different periods of employment did you (.....) have?

		FREQ	WTD
1	One	283	34,575
2	Two	95	11,906
3	Three or more	71	7,008
93	Not applicable	19,647	3,272,956
98	Not stated	600	93,059
R	Refusal	0	0
X	Don't know	14	834
		======	=========
		20,710	3,420,338

Coverage: Respondents (DISAB=1) aged 15-64, unemployed in reference week (LFSTAT=2), who answered E55=1
Source: Participation and Activity Limitation Survey, 2001

Variable Name:	**E57**	*Position:*	896	*Length:*	2
Collection Name:	AE57				

What was the length of the longest period of employment?

		FREQ	WTD
1	Under three months	134	17,126
2	Three to five months	90	9,676
3	Six months or more	222	26,595
93	Not applicable	19,661	3,273,789
98	Not stated	603	93,151
R	Refusal	0	0
X	Don't know	0	0
		======	=========
		20,710	3,420,337

Coverage: Respondents (DISAB=1) aged 15-64, unemployed in reference week (LFSTAT=2), who answered E56=1,2 or 3
Source: Participation and Activity Limitation Survey, 2001

Variable Name: **E58** *Position:* 898 *Length:* 2
Collection Name: AE58

Does your (..... 's) condition limit the amount or kind of work you (he / she) can do at a job or business?

		FREQ	WTD
1	Yes	553	69,385
3	No	264	24,908
93	Not applicable	19,330	3,233,775
98	Not stated	555	91,625
R	Refusal	0	0
X	Don't know	8	645
		======	=========
		20,710	3,420,338

Coverage: Respondents (DISAB=1) aged 15-64, unemployed in reference week (LFSTAT=2)
Source: Participation and Activity Limitation Survey, 2001

Variable Name: **E59** *Position:* 900 *Length:* 2
Collection Name: AE59

Were you (Was.....) working at a job or business at the time you (he / she) became limited in the kind or amount of work you (he / she) can do?

		FREQ	WTD
1	Yes	289	41,811
3	No	172	15,618
93	Not applicable	19,626	3,261,201
98	Not stated	621	101,662
R	Refusal	0	0
X	Don't know	2	46
		======	=========
		20,710	3,420,338

Coverage: Respondents (DISAB=1) aged 15-64, unemployed in reference week (LFSTAT=2), who DID NOT answer E49=1
Source: Participation and Activity Limitation Survey, 2001

Variable Name: **E60** *Position:* 902 *Length:* 2
Collection Name: AE60

Does your (..... 's) condition affect your (his / her) ability to look for work?

		FREQ	WTD
1	Yes	332	44,687
3	No	491	48,095
93	Not applicable	19,330	3,233,775
98	Not stated	548	91,188
R	Refusal	0	0
X	Don't know	9	2,592
		======	=========
		20,710	3,420,337

Coverage: Respondents (DISAB=1) aged 15-64, unemployed in reference week (LFSTAT=2)
Source: Participation and Activity Limitation Survey, 2001

Variable Name: **E61** *Position:* 904 *Length:* 2
Collection Name: AE61

Would you (.....)prefer to work...

		FREQ	WTD
1	either full-time or part-time?	197	22,965
2	full-time only?	387	46,504
3	part-time only?	245	23,184
93	Not applicable	19,330	3,233,775
98	Not stated	538	91,392
R	Refusal	0	0
X	Don't know	13	2,517
		======	=========
		20,710	3,420,337

Coverage: Respondents (DISAB=1) aged 15-64, unemployed in reference week (LFSTAT=2)
Source: Participation and Activity Limitation Survey, 2001

Variable Name:	**E62A**	*Position:*	906	*Length:*	2
Collection Name:	AE62A				

Because of your (..... 's) condition, are you (is he / she) limited in your (his / her) ability to work at a full-time job?

		FREQ	WTD
1	Yes	108	14,119
3	No	109	7,652
93	Not applicable	19,927	3,305,761
98	Not stated	561	92,480
R	Refusal	0	0
X	Don't know	5	326
		======	=========
		20,710	3,420,338

Coverage: Respondents (DISAB=1) aged 15-64, unemployed in reference week (LFSTAT=2), who answered E61=3
Source: Participation and Activity Limitation Survey, 2001

Variable Name:	**E62B**	*Position:*	908	*Length:*	2
Collection Name:	AE62B				

Because of your (..... 's) condition, are you (is he / she) limited in your (his / her) ability to work at a part-time job?

		FREQ	WTD
1	Yes	33	6,822
3	No	180	14,707
93	Not applicable	19,927	3,305,761
98	Not stated	562	92,516
R	Refusal	0	0
X	Don't know	8	532
		======	=========
		20,710	3,420,338

Coverage: Respondents (DISAB=1) aged 15-64, unemployed in reference week (LFSTAT=2), who answered E61=3
Source: Participation and Activity Limitation Survey, 2001

Variable Name:	**E63A**	*Position:*	910	*Length:*	2
Collection Name:	AE63A				

Because of your (his / her) condition, do you (does.....) require any of the following to be able to work: Job redesign (modified or different duties)?

		FREQ	WTD
1	Yes	324	40,214
3	No	494	52,569
93	Not applicable	19,330	3,233,775
98	Not stated	538	89,991
R	Refusal	0	0
X	Don't know	24	3,789
		======	=========
		20,710	3,420,338

Coverage: Respondents (DISAB=1) aged 15-64, unemployed in reference week (LFSTAT=2)
Source: Participation and Activity Limitation Survey, 2001

Variable Name:	**E63B**	*Position:*	912	*Length:*	2
Collection Name:	AE63B				

Because of your (his / her) condition, do you (does.....) require any of the following to be able to work: Modified hours or days or reduced work hours?

		FREQ	WTD
1	Yes	262	34,148
3	No	563	59,933
93	Not applicable	19,330	3,233,775
98	Not stated	539	90,114
R	Refusal	0	0
X	Don't know	16	2,368
		======	=========
		20,710	3,420,338

Coverage: Respondents (DISAB=1) aged 15-64, unemployed in reference week (LFSTAT=2)
Source: Participation and Activity Limitation Survey, 2001

Variable Name: **E63C** *Position:* 914 *Length:* 2
Collection Name: AE63C

Because of your (his / her) condition, do you (does.....) require any of the following to be able to work: Human support such as a reader, Sign language interpreter, job coach or personal assistant?

		FREQ	WTD
1	Yes	66	9,687
3	No	766	85,867
93	Not applicable	19,330	3,233,775
98	Not stated	538	90,004
R	Refusal	0	0
X	Don't know	10	1,004
		======	=========
		20,710	3,420,337

Coverage: Respondents (DISAB=1) aged 15-64, unemployed in reference week (LFSTAT=2)
Source: Participation and Activity Limitation Survey, 2001

Variable Name: **E63D** *Position:* 916 *Length:* 2
Collection Name: AE63D

Because of your (his / her) condition, do you (does.....) require any of the following to be able to work: Technical aids, such as a voice synthesizer, a TTY or TDD, an infrared system or portable note-takers?

		FREQ	WTD
1	Yes	33	3,632
3	No	806	92,260
93	Not applicable	19,330	3,233,775
98	Not stated	536	89,904
R	Refusal	0	0
X	Don't know	5	767
		======	=========
		20,710	3,420,338

Coverage: Respondents (DISAB=1) aged 15-64, unemployed in reference week (LFSTAT=2)
Source: Participation and Activity Limitation Survey, 2001

Variable Name: **E63E** *Position:* 918 *Length:* 2
Collection Name: AE63E

Because of your (his / her) condition, do you (does.....) require any of the following to be able to work: A computer with Braille, large print or speech access, or a scanner?

		FREQ	WTD
1	Yes	19	1,959
3	No	817	92,446
93	Not applicable	19,330	3,233,775
98	Not stated	538	89,966
R	Refusal	0	0
X	Don't know	6	2,192
		======	=========
		20,710	3,420,338

Coverage: Respondents (DISAB=1) aged 15-64, unemployed in reference week (LFSTAT=2)
Source: Participation and Activity Limitation Survey, 2001

Variable Name: **E63F** *Position:* 920 *Length:* 2
Collection Name: AE63F

Because of your (his / her) condition, do you (does.....) require any of the following to be able to work: Communication aids, such as Braille or large print reading material or recording equipment?

		FREQ	WTD
1	Yes	12	1,182
3	No	825	94,292
93	Not applicable	19,330	3,233,775
98	Not stated	538	90,036
R	Refusal	0	0
X	Don't know	5	1,052
		======	=========
		20,710	3,420,337

Coverage: Respondents (DISAB=1) aged 15-64, unemployed in reference week (LFSTAT=2)
Source: Participation and Activity Limitation Survey, 2001

Variable Name: **E63G** *Position:* 922 *Length:* 2
Collection Name: AE63G

Because of your (his / her) condition, do you (does.....) require any of the following to be able to work: Other equipment, help or work arrangement?

		FREQ	WTD
1	Yes	63	6,218
3	No	770	88,966
93	Not applicable	19,330	3,233,775
98	Not stated	538	89,999
R	Refusal	0	0
X	Don't know	9	1,379
		======	=========
		20,710	3,420,337

Coverage: Respondents (DISAB=1) aged 15-64, unemployed in reference week (LFSTAT=2)
Source: Participation and Activity Limitation Survey, 2001

Variable Name: **E64A** *Position:* 924 *Length:* 2
Collection Name: AE64A

Do you (Does.....) require modified features or arrangements in the workplace, such as: handrails, ramps?

		FREQ	WTD
1	Yes	47	6,143
3	No	793	89,620
93	Not applicable	19,330	3,233,775
98	Not stated	536	89,904
R	Refusal	0	0
X	Don't know	4	896
		======	=========
		20,710	3,420,338

Coverage: Respondents (DISAB=1) aged 15-64, unemployed in reference week (LFSTAT=2)
Source: Participation and Activity Limitation Survey, 2001

Variable Name: **E64B** *Position:* 926 *Length:* 2
Collection Name: AE64B

Do you (Does.....) require modified features or arrangements in the workplace, such as: appropriate parking?

		FREQ	WTD
1	Yes	76	11,315
3	No	762	84,712
93	Not applicable	19,330	3,233,775
98	Not stated	537	90,004
R	Refusal	0	0
X	Don't know	5	532
		======	=========
		20,710	3,420,338

Coverage: Respondents (DISAB=1) aged 15-64, unemployed in reference week (LFSTAT=2)
Source: Participation and Activity Limitation Survey, 2001

Variable Name: **E64C** *Position:* 928 *Length:* 2
Collection Name: AE64C

Do you (Does.....) require modified features or arrangements in the workplace, such as: accessible elevator?

		FREQ	WTD
1	Yes	77	9,255
3	No	761	86,835
93	Not applicable	19,330	3,233,775
98	Not stated	538	89,978
R	Refusal	0	0
X	Don't know	4	495
		======	=========
		20,710	3,420,338

Coverage: Respondents (DISAB=1) aged 15-64, unemployed in reference week (LFSTAT=2)
Source: Participation and Activity Limitation Survey, 2001

Variable Name:	**E64D**	*Position:*	930	*Length:*	2
Collection Name:	AE64D				

Do you (Does.....) require modified features or arrangements in the workplace, such as: modified workstation?

		FREQ	WTD
1	Yes	98	11,906
3	No	741	84,293
93	Not applicable	19,330	3,233,775
98	Not stated	536	89,904
R	Refusal	0	0
X	Don't know	5	461
		======	=========
		20,710	3,420,339

Coverage: Respondents (DISAB=1) aged 15-64, unemployed in reference week (LFSTAT=2)
Source: Participation and Activity Limitation Survey, 2001

Variable Name:	**E64E**	*Position:*	932	*Length:*	2
Collection Name:	AE64E				

Do you (Does.....) require modified features or arrangements in the workplace, such as: accessible washrooms?

		FREQ	WTD
1	Yes	55	7,518
3	No	785	88,946
93	Not applicable	19,330	3,233,775
98	Not stated	538	90,010
R	Refusal	0	0
X	Don't know	2	88
		======	=========
		20,710	3,420,337

Coverage: Respondents (DISAB=1) aged 15-64, unemployed in reference week (LFSTAT=2)
Source: Participation and Activity Limitation Survey, 2001

Variable Name: **E64F** *Position:* 934 *Length:* 2
Collection Name: AE64F

Do you (Does.....) require modified features or arrangements in the workplace, such as: accessible transportation?

		FREQ	WTD
1	Yes	72	7,549
3	No	767	88,744
93	Not applicable	19,330	3,233,775
98	Not stated	537	89,925
R	Refusal	0	0
X	Don't know	4	345
		======	=========
		20,710	3,420,338

Coverage: Respondents (DISAB=1) aged 15-64, unemployed in reference week (LFSTAT=2)
Source: Participation and Activity Limitation Survey, 2001

Variable Name: **E64G** *Position:* 936 *Length:* 2
Collection Name: AE64G

Do you (Does.....) require modified features or arrangements in the workplace, such as: Other?

		FREQ	WTD
1	Yes	17	694
3	No	814	94,984
93	Not applicable	19,330	3,233,775
98	Not stated	546	90,523
R	Refusal	0	0
X	Don't know	3	361
		======	=========
		20,710	3,420,337

Coverage: Respondents (DISAB=1) aged 15-64, unemployed in reference week (LFSTAT=2)
Source: Participation and Activity Limitation Survey, 2001

Variable Name: **E65** *Position:* 938 *Length:* 2
Collection Name: AE65

In the past five years, have you (has.....) taken any work-related training courses to either improve your (his / her) skills or to learn new skills?

		FREQ	WTD
1	Yes	356	42,617
3	No	483	53,370
93	Not applicable	19,330	3,233,775
98	Not stated	536	89,990
R	Refusal	0	0
X	Don't know	5	586
		======	=========
		20,710	3,420,338

Coverage: Respondents (DISAB=1) aged 15-64, unemployed in reference week (LFSTAT=2)
Source: Participation and Activity Limitation Survey, 2001

Variable Name: **E66** *Position:* 940 *Length:* 2
Collection Name: AE66

What was the main reason you (.....) took the course? Was it...

		FREQ	WTD
1	for your (his / her) job or a future job?	289	35,592
2	because of your (his / her) condition?	15	2,773
3	for personal interest?	35	2,786
4	for another reason?	12	740
93	Not applicable	19,818	3,287,731
98	Not stated	541	90,715
R	Refusal	0	0
X	Don't know	0	0
		======	=========
		20,710	3,420,337

Coverage: Respondents (DISAB=1) aged 15-64, unemployed in reference week (LFSTAT=2), who answered E65=1
Source: Participation and Activity Limitation Survey, 2001

Variable Name: **E67**	*Position:* 942	*Length:* 2	
Collection Name: AE67			

At work, to what extent were you (was he / she) using the skills or knowledge acquired in the course?

		FREQ	WTD
1	To a great extent	176	19,556
2	Somewhat	70	7,341
3	Very little	27	4,812
4	Not at all	68	9,872
93	Not applicable	19,818	3,287,731
98	Not stated	538	90,193
R	Refusal	0	0
X	Don't know	13	833
		======	=========
		20,710	3,420,338

Coverage: Respondents (DISAB=1) aged 15-64, unemployed in reference week (LFSTAT=2), who answered E65=1
Source: Participation and Activity Limitation Survey, 2001

Variable Name: **E68**	*Position:* 944	*Length:* 2	
Collection Name: AE68			

Did you (.....) want to take some work-related training courses?

		FREQ	WTD
1	Yes	205	20,797
3	No	270	32,245
93	Not applicable	19,686	3,276,392
98	Not stated	538	90,249
R	Refusal	0	0
X	Don't know	11	655
		======	=========
		20,710	3,420,338

Coverage: Respondents (DISAB=1) aged 15-64, unemployed in reference week (LFSTAT=2), who DID NOT answer E65=1
Source: Participation and Activity Limitation Survey, 2001

Variable Name:	**E69A**	*Position:*	946	*Length:*	2
Collection Name:	AE69A				

Did any of the following prevent you (.....) from taking those courses: Location was not physically accessible to you (him / her)?

		FREQ	WTD
1	Yes	39	3,475
3	No	164	17,119
93	Not applicable	19,967	3,309,292
98	Not stated	539	90,416
R	Refusal	0	0
X	Don't know	1	36
		======	=========
		20,710	3,420,338

Coverage: Respondents (DISAB=1) aged 15-64, unemployed in reference week (LFSTAT=2), who answered E68=1
Source: Participation and Activity Limitation Survey, 2001

Variable Name:	**E69B**	*Position:*	948	*Length:*	2
Collection Name:	AE69B				

Did any of the following prevent you (.....) from taking those courses: Courses were not adapted to your (his / her) needs?

		FREQ	WTD
1	Yes	28	3,074
3	No	172	17,200
93	Not applicable	19,967	3,309,292
98	Not stated	538	90,249
R	Refusal	0	0
X	Don't know	5	523
		======	=========
		20,710	3,420,338

Coverage: Respondents (DISAB=1) aged 15-64, unemployed in reference week (LFSTAT=2), who answered E68=1
Source: Participation and Activity Limitation Survey, 2001

Variable Name:	**E69C**	*Position:*	950	*Length:*	2
Collection Name:	AE69C				

Did any of the following prevent you (.....) from taking those courses: You (He / She) requested courses, but were denied them (by employer)?

		FREQ	WTD
1	Yes	19	2,399
3	No	182	16,917
93	Not applicable	19,967	3,309,292
98	Not stated	540	91,408
R	Refusal	0	0
X	Don't know	2	323
		======	=========
		20,710	3,420,339

Coverage: Respondents (DISAB=1) aged 15-64, unemployed in reference week (LFSTAT=2), who answered E68=1
Source: Participation and Activity Limitation Survey, 2001

Variable Name:	**E69D**	*Position:*	952	*Length:*	2
Collection Name:	AE69D				

Did any of the following prevent you (.....) from taking those courses: Your (His / Her) condition?

		FREQ	WTD
1	Yes	58	6,212
3	No	146	14,416
93	Not applicable	19,967	3,309,292
98	Not stated	538	90,249
R	Refusal	0	0
X	Don't know	1	169
		======	=========
		20,710	3,420,338

Coverage: Respondents (DISAB=1) aged 15-64, unemployed in reference week (LFSTAT=2), who answered E68=1
Source: Participation and Activity Limitation Survey, 2001

Variable Name:	**E69E**	*Position:*	954	*Length:*	2
Collection Name:	AE69E				

Did any of the following prevent you (.....) from taking those courses: Inadequate transportation?

		FREQ	WTD
1	Yes	44	4,088
3	No	161	16,709
93	Not applicable	19,967	3,309,292
98	Not stated	538	90,249
R	Refusal	0	0
X	Don't know	0	0
		======	=========
		20,710	3,420,338

Coverage: Respondents (DISAB=1) aged 15-64, unemployed in reference week (LFSTAT=2), who answered E68=1
Source: Participation and Activity Limitation Survey, 2001

Variable Name:	**E69F**	*Position:*	956	*Length:*	2
Collection Name:	AE69F				

Did any of the following prevent you (.....) from taking those courses: Too costly?

		FREQ	WTD
1	Yes	117	12,096
3	No	87	8,306
93	Not applicable	19,967	3,309,292
98	Not stated	538	90,249
R	Refusal	0	0
X	Don't know	1	394
		======	=========
		20,710	3,420,337

Coverage: Respondents (DISAB=1) aged 15-64, unemployed in reference week (LFSTAT=2), who answered E68=1
Source: Participation and Activity Limitation Survey, 2001

Variable Name:	**E69G**	*Position:*	958	*Length:*	2
Collection Name:	AE69G				

Did any of the following prevent you (.....) from taking those courses: Other reason?

		FREQ	WTD
1	Yes	54	4,510
3	No	149	16,228
93	Not applicable	19,967	3,309,292
98	Not stated	540	90,308
R	Refusal	0	0
X	Don't know	0	0
		======	=========
		20,710	3,420,338

Coverage: Respondents (DISAB=1) aged 15-64, unemployed in reference week (LFSTAT=2), who answered E68=1
Source: Participation and Activity Limitation Survey, 2001

Variable Name: **E70A** *Position:* 960 *Length:* 2
Collection Name: AE70A

In the past five years, do you (does.....) believe that because of your (his / her) condition, you have (he / she has) been refused... employment?

		FREQ	WTD
1	Yes	206	22,456
3	No	620	69,197
93	Not applicable	19,330	3,233,775
98	Not stated	533	89,720
R	Refusal	0	0
X	Don't know	21	5,189
		======	=========
		20,710	3,420,337

Coverage: Respondents (DISAB=1) aged 15-64, unemployed in reference week (LFSTAT=2)
Source: Participation and Activity Limitation Survey, 2001

Variable Name: **E70B** *Position:* 962 *Length:* 2
Collection Name: AE70B

In the past five years, do you (does.....) believe that because of your (his / her) condition, you have (he / she has) been refused... a promotion?

		FREQ	WTD
1	Yes	69	7,866
3	No	755	83,182
93	Not applicable	19,330	3,233,775
98	Not stated	532	89,700
R	Refusal	0	0
X	Don't know	24	5,815
		======	=========
		20,710	3,420,338

Coverage: Respondents (DISAB=1) aged 15-64, unemployed in reference week (LFSTAT=2)
Source: Participation and Activity Limitation Survey, 2001

Variable Name:	**E70C**	*Position:*	964	*Length:*	2
Collection Name:	AE70C				

In the past five years, do you (does.....) believe that because of your (his / her) condition, you have (he / she has) been refused... access to training programs?

		FREQ	WTD
1	Yes	57	7,377
3	No	771	85,568
93	Not applicable	19,330	3,233,775
98	Not stated	533	89,767
R	Refusal	0	0
X	Don't know	19	3,850
		======	=========
		20,710	3,420,337

Coverage: Respondents (DISAB=1) aged 15-64, unemployed in reference week (LFSTAT=2)
Source: Participation and Activity Limitation Survey, 2001

Variable Name:	**E70D**	*Position:*	966	*Length:*	2
Collection Name:	AE70D				

In the past five years, do you (does.....) believe that because of your (his / her) condition, you have (he / she has) been refused... or, has your (his / her) employment been terminated?

		FREQ	WTD
1	Yes	134	14,367
3	No	695	78,312
93	Not applicable	19,330	3,233,775
98	Not stated	534	89,770
R	Refusal	1	22
X	Don't know	16	4,091
		======	=========
		20,710	3,420,337

Coverage: Respondents (DISAB=1) aged 15-64, unemployed in reference week (LFSTAT=2)
Source: Participation and Activity Limitation Survey, 2001

Variable Name: **E71** *Position:* 968 *Length:* 2
Collection Name: AE71

Would you (.....) consider yourself (himself / herself) to be disadvantaged in employment because of your (his / her) condition, if you (he / she) were employed?

		FREQ	WTD
1	Yes	373	47,061
3	No	450	46,720
93	Not applicable	19,330	3,233,775
98	Not stated	535	90,569
R	Refusal	1	22
X	Don't know	21	2,191
		======	=========
		20,710	3,420,338

Coverage: Respondents (DISAB=1) aged 15-64, unemployed in reference week (LFSTAT=2)
Source: Participation and Activity Limitation Survey, 2001

Variable Name: **E72** *Position:* 970 *Length:* 2
Collection Name: AE72

Do you (Does.....) believe that any potential employer would be likely to consider you (him/ her) disadvantaged in employment because of your (his / her) condition?

		FREQ	WTD
1	Yes	396	52,944
3	No	388	35,135
93	Not applicable	19,330	3,233,775
98	Not stated	539	89,995
R	Refusal	1	22
X	Don't know	56	8,467
		======	=========
		20,710	3,420,338

Coverage: Respondents (DISAB=1) aged 15-64, unemployed in reference week (LFSTAT=2)
Source: Participation and Activity Limitation Survey, 2001

Section: **EMPLOYMENT-NOT IN LABOUR FORCE**

Variable Name: **E73_LSTWK** *Position:* 972 *Length:* 2
Collection Name: E73_LSTWK

Derived variable: Not in labour force - When last worked

		FREQ	WTD
1	Before 1996	2,100	442,342
2	Between 1996 and 1999	1,227	195,014
3	In 2000	487	67,616
4	In 2001	749	95,804
5	Never worked	1,099	118,888
93	Not applicable	14,344	2,376,583
98	Not stated	704	124,089
		======	=========
		20,710	3,420,336

Coverage: Respondents (DISAB=1) aged 15-64, who were not in the labour force in reference week (LFSTAT=3)
Source: Participation and Activity Limitation Survey, 2001
Note: E73_LSTWK is derived from variables E73 and E73S (write-in response). This variable is available only for disabled persons (DISAB = 1).

Variable Name: **E82_102** *Position:* 974 *Length:* 2
Collection Name: E82_102

Does your (..... 's) condition limit the amount or kind of work you (he / she) could do at a job or business?

		FREQ	WTD
1	Yes	2,788	420,979
3	No	635	88,486
93	Not applicable	16,410	2,762,254
98	Not stated	791	139,279
R	Refusal	1	21
X	Don't know	85	9,319
		======	=========
		20,710	3,420,338

Coverage: Respondents (DISAB=1) aged 15-64, not in the labour force in reference week (LFSTAT=3), who DID NOT answer E81=1 and E101=1
Source: Participation and Activity Limitation Survey, 2001
Note: E82_102 is derived from variables E82 and E102. Therefore, it covers both respondents who are retired and those who are not.

Variable Name: **E91_104A** *Position:* 976 *Length:* 2
Collection Name: E91_104A

Because of your (..... 's) condition, would you (he / she) require any of the following to be able to work: Job redesign (modified or different duties)?

		FREQ	WTD
1	Yes	2,202	334,350
3	No	2,928	488,227
93	Not applicable	14,283	2,368,311
98	Not stated	713	122,820
R	Refusal	24	4,568
X	Don't know	560	102,062
		======	=========
		20,710	3,420,338

Coverage: Respondents (DISAB=1) aged 15-64, not in the labour force in reference week (LFSTAT=3)
Source: Participation and Activity Limitation Survey, 2001
Note: E91_104A is derived from variables from E91A and E104A. Therefore, it covers both respondents who are retired and those who are not.

Variable Name: **E91_104B** *Position:* 978 *Length:* 2
Collection Name: E91_104B

Because of your (..... 's) condition, would you (he / she) require any of the following to be able to work: Modified hours or days or reduced work hours?

		FREQ	WTD
1	Yes	2,286	358,767
3	No	2,924	479,223
93	Not applicable	14,283	2,368,311
98	Not stated	716	123,317
R	Refusal	23	3,996
X	Don't know	478	86,725
		======	=========
		20,710	3,420,339

Coverage: Respondents (DISAB=1) aged 15-64, not in the labour force in reference week (LFSTAT=3)
Source: Participation and Activity Limitation Survey, 2001
Note: E91_104B is derived from variables from E91B and E104B. Therefore, it covers both respondents who are retired and those who are not.

Variable Name: **E91_104C** *Position:* 980 *Length:* 2
Collection Name: E91_104C

Because of your (..... 's) condition, would you (he / she) require any of the following to be able to work: Human support such as a reader, Sign language interpreter, job coach or personal assistant?

		FREQ	WTD
1	Yes	747	96,514
3	No	4,598	763,187
93	Not applicable	14,283	2,368,311
98	Not stated	711	123,679
R	Refusal	25	4,084
X	Don't know	346	64,564
		======	=========
		20,710	3,420,339

Coverage: Respondents (DISAB=1) aged 15-64, not in the labour force in reference week (LFSTAT=3)
Source: Participation and Activity Limitation Survey, 2001
Note: E91_104C is derived from variables from E91C and E104C. Therefore, it covers both respondents who are retired and those who are not.

Variable Name: **E91_104D** *Position:* 982 *Length:* 2
Collection Name: E91_104D

Because of your (..... 's) condition, would you (he / she) require any of the following to be able to work: Technical aids, such as a voice synthesizer, a TTY or TDD, an infrared system or portable note-takers?

		FREQ	WTD
1	Yes	275	35,281
3	No	5,093	825,857
93	Not applicable	14,283	2,368,311
98	Not stated	714	123,797
R	Refusal	26	4,286
X	Don't know	319	62,805
		======	=========
		20,710	3,420,337

Coverage: Respondents (DISAB=1) aged 15-64, not in the labour force in reference week (LFSTAT=3)
Source: Participation and Activity Limitation Survey, 2001
Note: E91_104D is derived from variables from E91D and E104D. Therefore, it covers both respondents who are retired and those who are not.

Variable Name:	**E91_104E**	*Position:*	984	*Length:*	2
Collection Name:	E91_104E				

Because of your (..... 's) condition, would you (he / she) require any of the following to be able to work: A computer with Braille, large print or speech access, or a scanner?

		FREQ	WTD
1	Yes	289	38,326
3	No	5,101	827,484
93	Not applicable	14,283	2,368,311
98	Not stated	715	124,241
R	Refusal	26	4,286
X	Don't know	296	57,690
		======	=========
		20,710	3,420,338

Coverage: Respondents (DISAB=1) aged 15-64, not in the labour force in reference week (LFSTAT=3)
Source: Participation and Activity Limitation Survey, 2001
Note: E91_104E is derived from variables from E91E and E104E. Therefore, it covers both respondents who are retired and those who are not.

Variable Name:	**E91_104F**	*Position:*	986	*Length:*	2
Collection Name:	E91_104F				

Because of your (..... 's) condition, would you (he / she) require any of the following to be able to work: Communication aids, such as Braille or large print reading material or recording equipment?

		FREQ	WTD
1	Yes	260	37,225
3	No	5,134	828,919
93	Not applicable	14,283	2,368,311
98	Not stated	714	124,596
R	Refusal	26	4,286
X	Don't know	293	57,000
		======	=========
		20,710	3,420,337

Coverage: Respondents (DISAB=1) aged 15-64, not in the labour force in reference week (LFSTAT=3)
Source: Participation and Activity Limitation Survey, 2001
Note: E91_104F is derived from variables from E91F and E104F. Therefore, it covers both respondents who are retired and those who are not.

Variable Name: **E91_104G** *Position:* 988 *Length:* 2
Collection Name: E91_104G

Because of your (..... 's) condition, would you (he / she) require any of the following to be able to work: Other equipment, help or work arrangement?

		FREQ	WTD
1	Yes	380	59,496
3	No	4,950	795,415
93	Not applicable	14,283	2,368,311
98	Not stated	725	127,313
R	Refusal	25	4,262
X	Don't know	347	65,542
		======	=========
		20,710	3,420,339

Coverage: Respondents (DISAB=1) aged 15-64, not in the labour force in reference week (LFSTAT=3)
Source: Participation and Activity Limitation Survey, 2001
Note: E91_104G is derived from variables from E91G and E104G. Therefore, it covers both respondents who are retired and those who are not.

Variable Name: **E92_105A** *Position:* 990 *Length:* 2
Collection Name: E92_105A

Because of your (..... 's) condition, would you (he / she) require modified features or arrangements in the workplace, such as: handrails, ramps?

		FREQ	WTD
1	Yes	1,081	184,211
3	No	4,383	695,186
93	Not applicable	14,283	2,368,311
98	Not stated	707	123,272
R	Refusal	26	6,287
X	Don't know	230	43,073
		======	=========
		20,710	3,420,340

Coverage: Respondents (DISAB=1) aged 15-64, not in the labour force in reference week (LFSTAT=3)
Source: Participation and Activity Limitation Survey, 2001
Note: E92_105A is derived from variables from E92A and E105A. Therefore, it covers both respondents who are retired and those who are not.

Variable Name:	**E92_105B**	*Position:*	992	*Length:*	2
Collection Name:	E92_105B				

Because of your (..... 's) condition, would you (he / she) require modified features or arrangements in the workplace, such as: appropriate parking?

		FREQ	WTD
1	Yes	1,201	211,366
3	No	4,263	670,788
93	Not applicable	14,283	2,368,311
98	Not stated	717	124,833
R	Refusal	24	4,909
X	Don't know	222	40,131
		======	=========
		20,710	3,420,338

Coverage: Respondents (DISAB=1) aged 15-64, not in the labour force in reference week (LFSTAT=3)
Source: Participation and Activity Limitation Survey, 2001
Note: E92_105B is derived from variables from E92B and E105B. Therefore, it covers both respondents who are retired and those who are not.

Variable Name:	**E92_105C**	*Position:*	994	*Length:*	2
Collection Name:	E92_105C				

Because of your (..... 's) condition, would you (he / she) require modified features or arrangements in the workplace, such as: accessible elevator?

		FREQ	WTD
1	Yes	1,368	234,374
3	No	4,090	647,117
93	Not applicable	14,283	2,368,311
98	Not stated	717	124,644
R	Refusal	24	4,909
X	Don't know	228	40,982
		======	=========
		20,710	3,420,337

Coverage: Respondents (DISAB=1) aged 15-64, not in the labour force in reference week (LFSTAT=3)
Source: Participation and Activity Limitation Survey, 2001
Note: E92_105C is derived from variables from E92C and E105C. Therefore, it covers both respondents who are retired and those who are not.

Variable Name:	**E92_105D**	*Position:*	996	*Length:*	2
Collection Name:	E92_105D				

Because of your (..... 's) condition, would you (he / she) require modified features or arrangements in the workplace, such as: modified workstation?

		FREQ	WTD
1	Yes	1,176	197,026
3	No	4,198	665,305
93	Not applicable	14,283	2,368,311
98	Not stated	722	127,960
R	Refusal	24	4,909
X	Don't know	307	56,827
		======	=========
		20,710	3,420,338

Coverage: Respondents (DISAB=1) aged 15-64, not in the labour force in reference week (LFSTAT=3)
Source: Participation and Activity Limitation Survey, 2001
Note: E92_105D is derived from variables from E92D and E105D. Therefore, it covers both respondents who are retired and those who are not.

Variable Name:	**E92_105E**	*Position:*	998	*Length:*	2
Collection Name:	E92_105E				

Because of your (..... 's) condition, would you (he / she) require modified features or arrangements in the workplace, such as: accessible washrooms?

		FREQ	WTD
1	Yes	1,013	169,642
3	No	4,453	712,328
93	Not applicable	14,283	2,368,311
98	Not stated	719	124,767
R	Refusal	24	4,909
X	Don't know	218	40,381
		======	=========
		20,710	3,420,338

Coverage: Respondents (DISAB=1) aged 15-64, not in the labour force in reference week (LFSTAT=3)
Source: Participation and Activity Limitation Survey, 2001
Note: E92_105E is derived from variables from E92E and E105E. Therefore, it covers both respondents who are retired and those who are not.

Variable Name: **E92_105F** *Position:* 1000 *Length:* 2
Collection Name: E92_105F

Because of your (..... 's) condition, would you (he / she) require modified features or arrangements in the workplace, such as: accessible transportation?

		FREQ	WTD
1	Yes	1,052	175,697
3	No	4,401	704,160
93	Not applicable	14,283	2,368,311
98	Not stated	719	124,977
R	Refusal	24	4,909
X	Don't know	231	42,283
		======	=========
		20,710	3,420,337

Coverage: Respondents (DISAB=1) aged 15-64, not in the labour force in reference week (LFSTAT=3)
Source: Participation and Activity Limitation Survey, 2001
Note: E92_105F is derived from variables from E92F and E105F. Therefore, it covers both respondents who are retired and those who are not.

Variable Name: **E92_105G** *Position:* 1002 *Length:* 2
Collection Name: E92_105G

Because of your (..... 's) condition, would you (he / she) require modified features or arrangements in the workplace, such as: Other?

		FREQ	WTD
1	Yes	212	35,706
3	No	5,200	834,287
93	Not applicable	14,283	2,368,311
98	Not stated	744	130,667
R	Refusal	23	4,760
X	Don't know	248	46,608
		======	=========
		20,710	3,420,339

Coverage: Respondents (DISAB=1) aged 15-64, not in the labour force in reference week (LFSTAT=3)
Source: Participation and Activity Limitation Survey, 2001
Note: E92_105G is derived from variables from E92G and E105G. Therefore, it covers both respondents who are retired and those who are not.

Variable Name:	**E98_106A**	*Position:*	1004	*Length:*	2
Collection Name:	E98_106A				

In the past five years, do you (does.....) believe that because of your (his / her) condition or health problem, you have (he / she has) been refused employment?

		FREQ	WTD
1	Yes	618	89,281
3	No	4,870	798,136
93	Not applicable	14,283	2,368,311
98	Not stated	692	121,419
R	Refusal	28	2,969
X	Don't know	219	40,222
		======	=========
		20,710	3,420,338

Coverage: Respondents (DISAB=1) aged 15-64, not in the labour force in reference week (LFSTAT=3)
Source: Participation and Activity Limitation Survey, 2001
Note: E98_106A is derived from variables from E98A and E106A. Therefore, it covers both respondents who are retired and those who are not.

Variable Name:	**E98_106B**	*Position:*	1006	*Length:*	2
Collection Name:	E98_106B				

In the past five years, do you (does.....) believe that because of your (his / her) condition or health problem, you have (he / she has) been refused a promotion?

		FREQ	WTD
1	Yes	171	25,676
3	No	5,322	857,285
93	Not applicable	14,283	2,368,311
98	Not stated	689	124,197
R	Refusal	28	2,969
X	Don't know	217	41,900
		======	=========
		20,710	3,420,338

Coverage: Respondents (DISAB=1) aged 15-64, not in the labour force in reference week (LFSTAT=3)
Source: Participation and Activity Limitation Survey, 2001
Note: E98_106B is derived from variables from E98B and E106B. Therefore, it covers both respondents who are retired and those who are not.

Variable Name: **E98_106C** *Position:* 1008 *Length:* 2
Collection Name: E98_106C

In the past five years, do you (does.....) believe that because of your (his / her) condition or health problem, you have (he / she has) been refused access to training programs?

		FREQ	WTD
1	Yes	177	22,814
3	No	5,325	867,016
93	Not applicable	14,283	2,368,311
98	Not stated	691	122,124
R	Refusal	29	2,993
X	Don't know	205	37,080
		======	=========
		20,710	3,420,338

Coverage: Respondents (DISAB=1) aged 15-64, not in the labour force in reference week (LFSTAT=3)
Source: Participation and Activity Limitation Survey, 2001
Note: E98_106C is derived from variables from E98C and E106C. Therefore, it covers both respondents who are retired and those who are not.

Variable Name: **E98_106D** *Position:* 1010 *Length:* 2
Collection Name: E98_106D

In the past five years, do you (does.....) believe that because of your (his / her) condition or health problem, you have (he / she has) been refused or, has your (his / her) employment been terminated?

		FREQ	WTD
1	Yes	601	88,221
3	No	4,913	800,569
93	Not applicable	14,283	2,368,311
98	Not stated	691	122,003
R	Refusal	28	2,969
X	Don't know	194	38,264
		======	=========
		20,710	3,420,337

Coverage: Respondents (DISAB=1) aged 15-64, not in the labour force in reference week (LFSTAT=3)
Source: Participation and Activity Limitation Survey, 2001
Note: E98_106D is derived from variables from E98D and E106D. Therefore, it covers both respondents who are retired and those who are not.

Variable Name: **E99_107** *Position:* 1012 *Length:* 2
Collection Name: E99_107

Would you (.....) consider yourself (himself / herself) to be disadvantaged in employment because of your (his / her) condition, if you (he / she) were employed?

		FREQ	WTD
1	Yes	3,816	651,963
3	No	1,591	222,950
93	Not applicable	14,283	2,368,311
98	Not stated	717	126,691
R	Refusal	17	2,398
X	Don't know	286	48,025
		======	=========
		20,710	3,420,338

Coverage: Respondents (DISAB=1) aged 15-64, not in the labour force in reference week (LFSTAT=3)
Source: Participation and Activity Limitation Survey, 2001
Note: E99_107 is derived from variables from E99 and E107. Therefore, it covers both respondents who are retired and those who are not.

Variable Name: **E100_108** *Position:* 1014 *Length:* 2
Collection Name: E100_108

Do you (Does.....) believe that any potential employer would be likely to consider you (him / her) disadvantaged in employment because of your (his / her) condition?

		FREQ	WTD
1	Yes	3,839	661,666
3	No	1,397	195,482
93	Not applicable	14,283	2,368,311
98	Not stated	709	123,388
R	Refusal	18	2,134
X	Don't know	464	69,356
		======	=========
		20,710	3,420,337

Coverage: Respondents (DISAB=1) aged 15-64, not in the labour force in reference week (LFSTAT=3)
Source: Participation and Activity Limitation Survey, 2001
Note: E100_108 is derived from variables from E100 and E108. Therefore, it covers both respondents who are retired and those who are not.

Section: **SOCIAL PARTICIPATION**

Variable Name: **F1** *Position:* 1016 *Length:* 2
Collection Name: AF1

In general, would you say your (..... 's) health is?

		FREQ	WTD
1	Excellent	1,449	197,541
2	Very good	4,211	606,734
3	Good	6,608	1,081,206
4	Fair	5,041	906,852
5	Poor	2,367	449,274
93	Not applicable	0	0
98	Not stated	984	168,240
R	Refusal	3	1,028
X	Don't know	47	9,463
		======	=========
		20,710	3,420,338

Coverage: All respondents (DISAB=1)
Source: Participation and Activity Limitation Survey, 2001

Variable Name: **F2** *Position:* 1018 *Length:* 2
Collection Name: AF2

Do you (Does.....) smoke cigarettes?

		FREQ	WTD
1	Not at all	14,862	2,500,392
2	Regularly, that is usually every day	4,248	654,271
3	Occasionally, not every day	787	118,047
93	Not applicable	0	0
98	Not stated	795	146,012
R	Refusal	6	861
X	Don't know	12	754
		======	=========
		20,710	3,420,337

Coverage: All respondents (DISAB=1)
Source: Participation and Activity Limitation Survey, 2001

Variable Name:	**F3**	*Position:*	1020	*Length:*	2
Collection Name:	AF3				

In the past twelve months, how often have you (has.....) had a drink?

		FREQ	WTD
1	Never	8,701	1,400,470
2	Every day	772	199,707
3	4 to 6 times a week	469	94,531
4	2 to 3 times a week	1,385	228,905
5	Once a week	1,620	265,955
6	Once or twice a month	2,737	418,456
7	Less than once a month	4,062	658,461
93	Not applicable	0	0
98	Not stated	869	142,638
R	Refusal	19	3,095
X	Don't know	76	8,121
		======	=========
		20,710	3,420,339

Coverage: All respondents (DISAB=1)
Source: Participation and Activity Limitation Survey, 2001

Variable Name:	**F4A**	*Position:*	1022	*Length:*	2
Collection Name:	AF4A				

In the past 12 months did you (.....) do any of the following activities WITHIN YOUR (HIS/ HER) HOME: exercice?

		FREQ	WTD
1	Every day	4,551	772,571
2	At least once a week	4,904	771,702
3	At least once a month	1,188	180,219
4	Less than once a month	1,103	163,684
5	Never	8,164	1,404,534
93	Not applicable	0	0
98	Not stated	747	120,442
R	Refusal	5	1,287
X	Don't know	48	5,898
		======	=========
		20,710	3,420,337

Coverage: All respondents (DISAB=1)
Source: Participation and Activity Limitation Survey, 2001

Variable Name: **F4B** *Position:* 1024 *Length:* 2
Collection Name: AF4B

In the past 12 months did you (.....) do any of the following activities WITHIN YOUR (HIS/ HER) HOME: stay in touch by email with family or friends?

		FREQ	WTD
1	Every day	1,923	263,523
2	At least once a week	2,346	335,731
3	At least once a month	884	130,676
4	Less than once a month	401	61,355
5	Never	14,366	2,501,112
93	Not applicable	0	0
98	Not stated	745	119,093
R	Refusal	7	1,625
X	Don't know	38	7,222
		======	=========
		20,710	3,420,337

Coverage: All respondents (DISAB=1)
Source: Participation and Activity Limitation Survey, 2001

Variable Name: **F4C** *Position:* 1026 *Length:* 2
Collection Name: AF4C

In the past 12 months did you (.....) do any of the following activities WITHIN YOUR (HIS/ HER) HOME: participate in electronic news groups or chat groups?

		FREQ	WTD
1	Every day	481	61,520
2	At least once a week	544	69,665
3	At least once a month	227	28,151
4	Less than once a month	292	42,332
5	Never	18,355	3,087,425
93	Not applicable	0	0
98	Not stated	726	119,263
R	Refusal	7	1,625
X	Don't know	78	10,357
		======	=========
		20,710	3,420,338

Coverage: All respondents (DISAB=1)
Source: Participation and Activity Limitation Survey, 2001

Variable Name: **F4D** *Position:* 1028 *Length:* 2
Collection Name: AF4D

In the past 12 months did you (.....) do any of the following activities WITHIN YOUR (HIS/ HER) HOME: surf the internet for information or e-commerce?

		FREQ	WTD
1	Every day	1,597	224,353
2	At least once a week	2,432	335,193
3	At least once a month	976	136,100
4	Less than once a month	583	93,804
5	Never	14,312	2,498,197
93	Not applicable	0	0
98	Not stated	748	123,687
R	Refusal	7	1,625
X	Don't know	55	7,379
		======	=========
		20,710	3,420,338

Coverage: All respondents (DISAB=1)
Source: Participation and Activity Limitation Survey, 2001

Variable Name: **F4E** *Position:* 1030 *Length:* 2
Collection Name: AF4E

In the past 12 months did you (.....) do any of the following activities WITHIN YOUR (HIS/ HER) HOME: do arts, crafts or hobbies within the home?

		FREQ	WTD
1	Every day	2,634	417,556
2	At least once a week	4,042	614,149
3	At least once a month	1,945	289,299
4	Less than once a month	1,179	201,118
5	Never	10,027	1,750,276
93	Not applicable	0	0
98	Not stated	812	133,853
R	Refusal	7	1,626
X	Don't know	64	12,461
		======	=========
		20,710	3,420,338

Coverage: All respondents (DISAB=1)
Source: Participation and Activity Limitation Survey, 2001

Variable Name: **F5A** *Position:* 1032 *Length:* 2
Collection Name: AF5A

How often do you (does he / she): watch TV or videos, listen to radio or CD's?

		FREQ	WTD
1	Every day	18,163	3,017,315
2	At least once a week	1,305	197,783
3	At least once a month	94	12,886
4	Less than once a month	71	11,600
5	Never	209	29,646
93	Not applicable	0	0
98	Not stated	842	147,232
R	Refusal	4	1,191
X	Don't know	22	2,684
		======	=========
		20,710	3,420,337

Coverage: All respondents (DISAB=1)
Source: Participation and Activity Limitation Survey, 2001

Variable Name: **F5B** *Position:* 1034 *Length:* 2
Collection Name: AF5B

How often do you (does he / she): read?

		FREQ	WTD
1	Every day	11,734	2,011,985
2	At least once a week	3,882	607,268
3	At least once a month	736	113,262
4	Less than once a month	649	99,815
5	Never	2,791	435,266
93	Not applicable	0	0
98	Not stated	874	144,648
R	Refusal	6	1,336
X	Don't know	38	6,757
		======	=========
		20,710	3,420,337

Coverage: All respondents (DISAB=1)
Source: Participation and Activity Limitation Survey, 2001

Variable Name: **F5C** *Position:* 1036 *Length:* 2
Collection Name: AF5C

How often do you (does he / she): talk on the telephone with family or friends?

		FREQ	WTD
1	Every day	9,400	1,456,947
2	At least once a week	7,737	1,347,692
3	At least once a month	1,176	207,880
4	Less than once a month	531	99,436
5	Never	994	158,048
93	Not applicable	0	0
98	Not stated	827	136,732
R	Refusal	7	1,812
X	Don't know	38	11,790
		======	=========
		20,710	3,420,337

Coverage: All respondents (DISAB=1)
Source: Participation and Activity Limitation Survey, 2001

Variable Name: **F6A** *Position:* 1038 *Length:* 2
Collection Name: AF6A

In the past 12 months, how often did you (.....) participate in any of the following activities OUTSIDE YOUR (HIS/ HER) HOME: visit family or friends?

		FREQ	WTD
1	Every day	1,780	198,447
2	At least once a week	9,415	1,489,328
3	At least once a month	4,866	859,383
4	Less than once a month	2,434	479,636
5	Never	1,359	249,279
93	Not applicable	0	0
98	Not stated	802	135,554
R	Refusal	9	1,269
X	Don't know	45	7,441
		======	=========
		20,710	3,420,337

Coverage: All respondents (DISAB=1)
Source: Participation and Activity Limitation Survey, 2001

Variable Name: **F6B** *Position:* 1040 *Length:* 2
Collection Name: AF6B

In the past 12 months, how often did you (.....) participate in any of the following activities OUTSIDE YOUR (HIS/ HER) HOME: do physical activities such as exercise, walk or play sports?

		FREQ	WTD
1	Every day	6,021	997,369
2	At least once a week	6,499	1,018,019
3	At least once a month	1,168	188,953
4	Less than once a month	781	128,723
5	Never	5,358	931,897
93	Not applicable	0	0
98	Not stated	843	147,469
R	Refusal	5	973
X	Don't know	35	6,936
		======	=========
		20,710	3,420,339

Coverage: All respondents (DISAB=1)
Source: Participation and Activity Limitation Survey, 2001

Variable Name: **F6C** *Position:* 1042 *Length:* 2
Collection Name: AF6C

In the past 12 months, how often did you (.....) participate in any of the following activities OUTSIDE YOUR (HIS/ HER) HOME: do hobbies outside the home such as playing cards, bridge or bingo?

		FREQ	WTD
1	Every day	423	66,251
2	At least once a week	3,851	563,044
3	At least once a month	1,905	295,727
4	Less than once a month	1,200	197,222
5	Never	12,354	2,127,615
93	Not applicable	0	0
98	Not stated	932	156,817
R	Refusal	6	996
X	Don't know	39	12,665
		======	=========
		20,710	3,420,337

Coverage: All respondents (DISAB=1)
Source: Participation and Activity Limitation Survey, 2001

Variable Name:	**F6D**	*Position:*	1044	*Length:*	2
Collection Name:	AF6D				

In the past 12 months, how often did you (.....) participate in any of the following activities OUTSIDE YOUR (HIS/ HER) HOME: shop?

		FREQ	WTD
1	Every day	709	130,623
2	At least once a week	11,066	1,906,740
3	At least once a month	4,397	618,459
4	Less than once a month	1,292	201,037
5	Never	2,282	401,855
93	Not applicable	0	0
98	Not stated	929	155,773
R	Refusal	5	894
X	Don't know	30	4,955
		======	=========
		20,710	3,420,336

Coverage: All respondents (DISAB=1)
Source: Participation and Activity Limitation Survey, 2001

Variable Name:	**F6E**	*Position:*	1046	*Length:*	2
Collection Name:	AF6E				

In the past 12 months, how often did you (.....) participate in any of the following activities OUTSIDE YOUR (HIS/ HER) HOME: attend sporting or cultural events, such as plays or movies?

		FREQ	WTD
2	At least once a week	1,464	185,626
3	At least once a month	3,770	523,785
4	Less than once a month	4,251	722,621
5	Never	10,277	1,822,372
93	Not applicable	0	0
98	Not stated	868	153,232
R	Refusal	6	993
X	Don't know	74	11,708
		======	=========
		20,710	3,420,337

Coverage: All respondents (DISAB=1)
Source: Participation and Activity Limitation Survey, 2001

Variable Name: **F6F** *Position:* 1048 *Length:* 2
Collection Name: AF6F

In the past 12 months, how often did you (.....) participate in any of the following activities OUTSIDE YOUR (HIS/ HER) HOME: take personal interest courses?

		FREQ	WTD
1	Every day	62	4,871
2	At least once a week	569	86,558
3	At least once a month	388	65,700
4	Less than once a month	1,241	201,588
5	Never	17,539	2,905,162
93	Not applicable	0	0
98	Not stated	834	142,334
R	Refusal	5	973
X	Don't know	72	13,152
		======	=========
		20,710	3,420,338

Coverage: All respondents (DISAB=1)
Source: Participation and Activity Limitation Survey, 2001

Variable Name: **F6G** *Position:* 1050 *Length:* 2
Collection Name: AF6G

In the past 12 months, how often did you (.....) participate in any of the following activities OUTSIDE YOUR (HIS/ HER) HOME: visit museums, libraries or national or provincial parks?

		FREQ	WTD
2	At least once a week	1,226	193,632
3	At least once a month	2,937	449,166
4	Less than once a month	5,479	856,638
5	Never	10,159	1,765,813
93	Not applicable	0	0
98	Not stated	824	139,334
R	Refusal	5	973
X	Don't know	80	14,782
		======	=========
		20,710	3,420,338

Coverage: All respondents (DISAB=1)
Source: Participation and Activity Limitation Survey, 2001

Variable Name: **F6H** *Position:* 1052 *Length:* 2
Collection Name: AF6H

In the past 12 months, how often did you (.....) participate in any of the following activities OUTSIDE YOUR (HIS/ HER) HOME: travel for business or personal reasons?

		FREQ	WTD
2	At least once a week	1,773	269,108
3	At least once a month	2,286	342,675
4	Less than once a month	6,984	1,131,434
5	Never	8,731	1,518,210
93	Not applicable	0	0
98	Not stated	874	147,228
R	Refusal	6	995
X	Don't know	56	10,689
		======	=========
		20,710	3,420,339

Coverage: All respondents (DISAB=1)
Source: Participation and Activity Limitation Survey, 2001

Variable Name: **F7** *Position:* 1054 *Length:* 2
Collection Name: AF7

Would you (.....) like to do more activities during your (his/her) spare time?

		FREQ	WTD
1	Yes	8,480	1,331,883
3	No	11,103	1,883,662
93	Not applicable	0	0
98	Not stated	728	125,922
R	Refusal	5	1,749
X	Don't know	394	77,121
		======	=========
		20,710	3,420,337

Coverage: All respondents (DISAB=1)
Source: Participation and Activity Limitation Survey, 2001

Variable Name: **F8A** *Position:* 1056 *Length:* 2
Collection Name: AF8A

What PREVENTS you (.....) from doing more leisure activities: Your (His/Her) condition prevents you (him/her) from doing more?

		FREQ	WTD
1	Yes	6,018	983,028
3	No	2,322	327,987
93	Not applicable	11,502	1,962,532
98	Not stated	844	144,287
R	Refusal	0	0
X	Don't know	24	2,504
		======	=========
		20,710	3,420,338

Coverage: Respondents (DISAB=1) who answered F7=1
Source: Participation and Activity Limitation Survey, 2001

Variable Name: **F8B** *Position:* 1058 *Length:* 2
Collection Name: AF8B

What PREVENTS you (.....) from doing more leisure activities: You need (He /She needs) specialized aid(s) or equipment that you don't (he /she doesn't) have?

		FREQ	WTD
1	Yes	564	93,241
3	No	7,644	1,200,151
93	Not applicable	11,502	1,962,532
98	Not stated	901	151,465
R	Refusal	0	0
X	Don't know	99	12,948
		======	=========
		20,710	3,420,337

Coverage: Respondents (DISAB=1) who answered F7=1
Source: Participation and Activity Limitation Survey, 2001

Variable Name: **F8C** *Position:* 1060 *Length:* 2
Collection Name: AF8C

What PREVENTS you (.....) from doing more leisure activities: You need (He /She needs) someone's assistance?

		FREQ	WTD
1	Yes	1,577	239,654
3	No	6,671	1,054,506
93	Not applicable	11,502	1,962,532
98	Not stated	909	154,538
R	Refusal	0	0
X	Don't know	51	9,108
		======	=========
		20,710	3,420,338

Coverage: Respondents (DISAB=1) who answered F7=1
Source: Participation and Activity Limitation Survey, 2001

Variable Name: **F8D** *Position:* 1062 *Length:* 2
Collection Name: AF8D

What PREVENTS you (.....) from doing more leisure activities: Your (His/Her) transportation services are inadequate or not accessible?

		FREQ	WTD
1	Yes	1,374	202,658
3	No	6,877	1,092,120
93	Not applicable	11,502	1,962,532
98	Not stated	907	155,337
R	Refusal	0	0
X	Don't know	50	7,691
		======	=========
		20,710	3,420,338

Coverage: Respondents (DISAB=1) who answered F7=1
Source: Participation and Activity Limitation Survey, 2001

Variable Name:	**F8E**	*Position:*	1064	*Length:*	2
Collection Name:	AF8E				

What PREVENTS you (.....) from doing more leisure activities: Your (His/Her) community has no facilities or programs available?

		FREQ	WTD
1	Yes	1,116	129,516
3	No	6,790	1,102,232
93	Not applicable	11,502	1,962,532
98	Not stated	914	153,632
R	Refusal	2	42
X	Don't know	386	72,384
		======	=========
		20,710	3,420,338

Coverage: Respondents (DISAB=1) who answered F7=1
Source: Participation and Activity Limitation Survey, 2001

Variable Name:	**F8F**	*Position:*	1066	*Length:*	2
Collection Name:	AF8F				

What PREVENTS you (.....) from doing more leisure activities: The facilities, equipment or programs are not accessible?

		FREQ	WTD
1	Yes	737	99,708
3	No	7,251	1,149,286
93	Not applicable	11,502	1,962,532
98	Not stated	917	156,942
R	Refusal	1	20
X	Don't know	302	51,851
		======	=========
		20,710	3,420,339

Coverage: Respondents (DISAB=1) who answered F7=1
Source: Participation and Activity Limitation Survey, 2001

Variable Name: **F8G** *Position:* 1068 *Length:* 2
Collection Name: AF8G

What PREVENTS you (.....) from doing more leisure activities: It is too expensive?

		FREQ	WTD
1	Yes	3,502	525,532
3	No	4,631	747,339
93	Not applicable	11,502	1,962,532
98	Not stated	893	153,486
R	Refusal	0	0
X	Don't know	182	31,449
		======	=========
		20,710	3,420,338

Coverage: Respondents (DISAB=1) who answered F7=1
Source: Participation and Activity Limitation Survey, 2001

Variable Name: **F8H** *Position:* 1070 *Length:* 2
Collection Name: AF8H

What PREVENTS you (.....) from doing more leisure activities: Other?

		FREQ	WTD
1	Yes	1,881	287,553
3	No	6,240	989,109
93	Not applicable	11,502	1,962,532
98	Not stated	1,016	169,393
R	Refusal	0	0
X	Don't know	71	11,751
		======	=========
		20,710	3,420,338

Coverage: Respondents (DISAB=1) who answered F7=1
Source: Participation and Activity Limitation Survey, 2001

Variable Name: **F9** *Position:* 1072 *Length:* 2
Collection Name: AF9

Does the design and layout of buildings and places in your (..... 's) community make it DIFFICULT for you (him/ her) to participate in leisure activities?

		FREQ	WTD
1	Yes	1,322	217,779
3	No	18,312	3,004,828
93	Not applicable	0	0
98	Not stated	781	130,247
R	Refusal	6	2,452
X	Don't know	289	65,033
		======	=========
		20,710	3,420,339

Coverage: All respondents (DISAB=1)
Source: Participation and Activity Limitation Survey, 2001

Variable Name: **F10** *Position:* 1074 *Length:* 2
Collection Name: AF10

In the past 12 months, how often has the design and layout of buildings and places made it DIFFICULT for you (.....) to participate in leisure activities?

		FREQ	WTD
1	Daily	254	44,965
2	Weekly	257	47,291
3	Monthly	315	56,310
4	Less than once a month	409	56,894
93	Not applicable	18,607	3,072,312
98	Not stated	817	134,758
R	Refusal	0	0
X	Don't know	51	7,808
		======	=========
		20,710	3,420,338

Coverage: Respondents (DISAB=1) who answered F9=1
Source: Participation and Activity Limitation Survey, 2001

Variable Name:	**F11**	*Position:*	1076	*Length:*	2
Collection Name:	AF11				

When this problem occurred, was it a big problem or a little problem?

		FREQ	WTD
1	Big problem	694	118,406
2	Little problem	529	83,028
93	Not applicable	18,607	3,072,312
98	Not stated	836	137,472
R	Refusal	0	0
X	Don't know	44	9,119
		======	=========
		20,710	3,420,337

Coverage: Respondents (DISAB=1) who answered F9=1
Source: Participation and Activity Limitation Survey, 2001

Variable Name:	**F12A**	*Position:*	1078	*Length:*	2
Collection Name:	AF12A				

In the past 12 months, did you (.....): help to organize or supervise activities or events for an organization?

		FREQ	WTD
1	Yes	3,445	506,355
3	No	16,347	2,764,037
93	Not applicable	0	0
98	Not stated	837	136,304
R	Refusal	6	2,121
X	Don't know	75	11,521
		======	=========
		20,710	3,420,338

Coverage: All respondents (DISAB=1)
Source: Participation and Activity Limitation Survey, 2001

Variable Name: **F12B** *Position:* 1080 *Length:* 2
Collection Name: AF12B

In the past 12 months, did you (.....): canvass, campaign or fund raise as an unpaid volunteer?

		FREQ	WTD
1	Yes	2,718	349,395
3	No	17,080	2,926,715
93	Not applicable	0	0
98	Not stated	834	132,095
R	Refusal	6	2,121
X	Don't know	72	10,012
		======	=========
		20,710	3,420,338

Coverage: All respondents (DISAB=1)
Source: Participation and Activity Limitation Survey, 2001

Variable Name: **F12C** *Position:* 1082 *Length:* 2
Collection Name: AF12C

In the past 12 months, did you (.....): sit as an unpaid member of a board or committee?

		FREQ	WTD
1	Yes	2,228	360,336
3	No	17,583	2,914,537
93	Not applicable	0	0
98	Not stated	825	132,492
R	Refusal	6	2,121
X	Don't know	68	10,852
		======	=========
		20,710	3,420,338

Coverage: All respondents (DISAB=1)
Source: Participation and Activity Limitation Survey, 2001

Variable Name: **F12D** *Position:* 1084 *Length:* 2
Collection Name: AF12D

In the past 12 months, did you (.....): do any consulting, executive, office or administrative work as a volunteer?

		FREQ	WTD
1	Yes	1,469	229,579
3	No	18,358	3,050,856
93	Not applicable	0	0
98	Not stated	808	128,113
R	Refusal	6	2,121
X	Don't know	69	9,669
		======	=========
		20,710	3,420,338

Coverage: All respondents (DISAB=1)
Source: Participation and Activity Limitation Survey, 2001

Variable Name: **F12E** *Position:* 1086 *Length:* 2
Collection Name: AF12E

In the past 12 months, did you (.....): provide information, help to educate, lobby or influence public opinion on behalf of an organization?

		FREQ	WTD
1	Yes	1,577	232,205
3	No	18,233	3,043,318
93	Not applicable	0	0
98	Not stated	822	132,631
R	Refusal	6	2,121
X	Don't know	72	10,063
		======	=========
		20,710	3,420,338

Coverage: All respondents (DISAB=1)
Source: Participation and Activity Limitation Survey, 2001

Variable Name:	**F12F**	*Position:*	1088	*Length:*	2
Collection Name:	AF12F				

In the past 12 months, did you (.....): teach, coach, provide care or friendly visits through an organization?

		FREQ	WTD
1	Yes	2,303	345,025
3	No	17,503	2,928,334
93	Not applicable	0	0
98	Not stated	825	134,769
R	Refusal	6	2,121
X	Don't know	73	10,089
		======	=========
		20,710	3,420,338

Coverage: All respondents (DISAB=1)
Source: Participation and Activity Limitation Survey, 2001

Variable Name:	**F12G**	*Position:*	1090	*Length:*	2
Collection Name:	AF12G				

In the past 12 months, did you (.....): collect, serve or deliver food or other goods as a volunteer through an organization?

		FREQ	WTD
1	Yes	1,619	225,204
3	No	18,204	3,049,693
93	Not applicable	0	0
98	Not stated	816	133,845
R	Refusal	6	2,121
X	Don't know	65	9,475
		======	=========
		20,710	3,420,338

Coverage: All respondents (DISAB=1)
Source: Participation and Activity Limitation Survey, 2001

Variable Name: **F12H** *Position:* 1092 *Length:* 2
Collection Name: AF12H

In the past 12 months, did you (.....): do any other unpaid volunteer activities (including help given to schools, religious organizations and community organizations)?

		FREQ	WTD
1	Yes	4,623	695,513
3	No	15,170	2,571,699
93	Not applicable	0	0
98	Not stated	836	140,076
R	Refusal	6	2,121
X	Don't know	75	10,928
		======	=========
		20,710	3,420,337

Coverage: All respondents (DISAB=1)
Source: Participation and Activity Limitation Survey, 2001

Variable Name: **F13** *Position:* 1094 *Length:* 2
Collection Name: AF13

In the past 12 months, did you (.....) travel locally by CAR for personal or business reasons?

		FREQ	WTD
1	Yes	17,633	2,894,680
3	No	2,378	412,511
93	Not applicable	0	0
98	Not stated	670	108,669
R	Refusal	6	1,430
X	Don't know	23	3,047
		======	=========
		20,710	3,420,337

Coverage: All respondents (DISAB=1)
Source: Participation and Activity Limitation Survey, 2001

Variable Name:	**F14**	*Position:*	1096	*Length:*	2
Collection Name:	AF14				

Were you (Was.....) PREVENTED from travelling locally by car?

		FREQ	WTD
1	Yes	386	60,059
3	No	1,995	350,504
93	Not applicable	17,633	2,894,680
98	Not stated	687	111,483
R	Refusal	4	1,612
X	Don't know	5	2,000
		======	=========
		20,710	3,420,338

Coverage: Respondents (DISAB=1) who DID NOT answer F13=1
Source: Participation and Activity Limitation Survey, 2001

Variable Name:	**F15A**	*Position:*	1098	*Length:*	2
Collection Name:	AF15A				

What PREVENTED you (.....) from travelling locally by car: The lack of proper equipment on your (his / her) car (for example, hand or brake controls, power steering, etc.)?

		FREQ	WTD
1	Yes	38	5,022
3	No	284	47,088
93	Not applicable	19,637	3,248,796
98	Not stated	748	119,125
R	Refusal	1	59
X	Don't know	2	248
		======	=========
		20,710	3,420,338

Coverage: Respondents (DISAB=1) who answered F14=1
Source: Participation and Activity Limitation Survey, 2001

Variable Name: **F15B** *Position:* 1100 *Length:* 2
Collection Name: AF15B

What PREVENTED you (.....) from travelling locally by car: You need (.....needs) an attendant to help you (him / her)?

		FREQ	WTD
1	Yes	108	16,646
3	No	217	35,505
93	Not applicable	19,637	3,248,796
98	Not stated	747	119,352
R	Refusal	0	0
X	Don't know	1	39
		======	=========
		20,710	3,420,338

Coverage: Respondents (DISAB=1) who answered F14=1
Source: Participation and Activity Limitation Survey, 2001

Variable Name: **F15C** *Position:* 1102 *Length:* 2
Collection Name: AF15C

What PREVENTED you (.....) from travelling locally by car: The lack of space for wheelchairs or other specialized equipment?

		FREQ	WTD
1	Yes	55	9,852
3	No	263	41,426
93	Not applicable	19,637	3,248,796
98	Not stated	753	120,207
R	Refusal	0	0
X	Don't know	2	57
		======	=========
		20,710	3,420,338

Coverage: Respondents (DISAB=1) who answered F14=1
Source: Participation and Activity Limitation Survey, 2001

Variable Name: **F15D** *Position:* 1104 *Length:* 2
Collection Name: AF15D

What PREVENTED you (.....) from travelling locally by car: Other reason?

		FREQ	WTD
1	Yes	264	40,984
3	No	88	14,135
93	Not applicable	19,637	3,248,796
98	Not stated	720	116,292
R	Refusal	0	0
X	Don't know	1	131
		======	=========
		20,710	3,420,338

Coverage: Respondents (DISAB=1) who answered F14=1
Source: Participation and Activity Limitation Survey, 2001

Variable Name: **F16** *Position:* 1106 *Length:* 2
Collection Name: AF16

In the past 12 months, did you (.....) have DIFFICULTY travelling locally by car due to your (his / her) condition?

		FREQ	WTD
1	Yes	3,282	554,965
3	No	14,219	2,316,340
93	Not applicable	2,407	416,989
98	Not stated	782	128,269
R	Refusal	1	283
X	Don't know	19	3,491
		======	=========
		20,710	3,420,337

Coverage: Respondents (DISAB=1) who answered F13=1
Source: Participation and Activity Limitation Survey, 2001

Variable Name:	**F17**	*Position:*	1108	*Length:*	2
Collection Name:	AF17				

Does this DIFFICULTY occur when you are (he / she is) the driver?

		FREQ	WTD
1	Yes	1,955	326,461
3	No	1,272	219,701
93	Not applicable	16,646	2,737,103
98	Not stated	815	132,602
R	Refusal	3	142
X	Don't know	19	4,329
		======	=========
		20,710	3,420,338

Coverage: Respondents (DISAB=1) who answered F16=1
Source: Participation and Activity Limitation Survey, 2001

Variable Name:	**F18A**	*Position:*	1110	*Length:*	2
Collection Name:	AF18A				

Is this DIFFICULTY...because you (he / she) lack(s) the proper equipment on your (his / her) car? (e.g., hand or brake controls, power steering)?

		FREQ	WTD
1	Yes	112	13,753
3	No	1,756	296,643
93	Not applicable	17,940	2,961,275
98	Not stated	893	146,328
R	Refusal	1	115
X	Don't know	8	2,224
		======	=========
		20,710	3,420,338

Coverage: Respondents (DISAB=1) who answered F17=1
Source: Participation and Activity Limitation Survey, 2001

Variable Name:	F18B	Position:	1112	Length:	2
Collection Name:	AF18B				

Is this DIFFICULTY...because you need (he / she needs) an attendant to help you (him / her)?

		FREQ	WTD
1	Yes	204	28,425
3	No	1,670	281,995
93	Not applicable	17,940	2,961,275
98	Not stated	892	147,587
R	Refusal	1	115
X	Don't know	3	942
		======	=========
		20,710	3,420,339

Coverage: Respondents (DISAB=1) who answered F17=1
Source: Participation and Activity Limitation Survey, 2001

Variable Name:	F18C	Position:	1114	Length:	2
Collection Name:	AF18C				

Is this DIFFICULTY...due to the lack of space for wheelchairs or other specialized equipment?

		FREQ	WTD
1	Yes	30	5,615
3	No	1,844	305,753
93	Not applicable	17,940	2,961,275
98	Not stated	892	146,754
R	Refusal	1	115
X	Don't know	3	826
		======	=========
		20,710	3,420,338

Coverage: Respondents (DISAB=1) who answered F17=1
Source: Participation and Activity Limitation Survey, 2001

Variable Name:	F18D	Position:	1116	Length:	2
Collection Name:	AF18D				

Is this DIFFICULTY...due to another reason?

		FREQ	WTD
1	Yes	1,631	275,149
3	No	295	45,689
93	Not applicable	17,940	2,961,275
98	Not stated	842	137,890
R	Refusal	1	115
X	Don't know	1	220
		======	=========
		20,710	3,420,338

Coverage: Respondents (DISAB=1) who answered F17=1
Source: Participation and Activity Limitation Survey, 2001

Variable Name:	**F19**	*Position:*	1118	*Length:*	2
Collection Name:	AF19				

How often was this a problem for you (.....)?

		FREQ	WTD
1	Daily	798	127,904
2	Weekly	570	108,411
3	Monthly or less often	490	75,869
4	Never	20	2,385
93	Not applicable	17,940	2,961,275
98	Not stated	861	141,490
R	Refusal	2	249
X	Don't know	29	2,756
		======	=========
		20,710	3,420,339

Coverage: Respondents (DISAB=1) who answered F17=1
Source: Participation and Activity Limitation Survey, 2001

Variable Name:	**F20**	*Position:*	1120	*Length:*	2
Collection Name:	AF20				

When this problem occurred, was it a big problem or a little problem?

		FREQ	WTD
1	Big problem	1,164	198,235
2	Little problem	671	109,406
93	Not applicable	17,960	2,963,659
98	Not stated	904	147,978
R	Refusal	0	0
X	Don't know	11	1,060
		======	=========
		20,710	3,420,338

Coverage: Respondents (DISAB=1) who DID NOT answer F19=4
Source: Participation and Activity Limitation Survey, 2001

Variable Name: **F21** *Position:* 1122 *Length:* 2
Collection Name: AF21

Does this DIFFICULTY occur when you are (..... is) a passenger?

		FREQ	WTD
1	Yes	2,549	432,664
3	No	674	112,092
93	Not applicable	16,646	2,737,103
98	Not stated	824	135,849
R	Refusal	0	0
X	Don't know	17	2,630
		======	=========
		20,710	3,420,338

Coverage: Respondents (DISAB=1) who answered F16=1
Source: Participation and Activity Limitation Survey, 2001

Variable Name: **F22A** *Position:* 1124 *Length:* 2
Collection Name: AF22A

Is this DIFFICULTY...because you need (.....needs) an attendant to help you (him / her)?

		FREQ	WTD
1	Yes	495	69,033
3	No	1,939	342,212
93	Not applicable	17,337	2,851,825
98	Not stated	935	156,948
R	Refusal	1	19
X	Don't know	3	300
		======	=========
		20,710	3,420,337

Coverage: Respondents (DISAB=1) who answered F21=1
Source: Participation and Activity Limitation Survey, 2001

Variable Name: **F22B** *Position:* 1126 *Length:* 2
Collection Name: AF22B

Is this DIFFICULTY...because there is no space for wheelchairs or other specialized equipment?

		FREQ	WTD
1	Yes	143	19,203
3	No	2,278	389,359
93	Not applicable	17,337	2,851,825
98	Not stated	946	158,932
R	Refusal	1	19
X	Don't know	5	1,000
		======	=========
		20,710	3,420,338

Coverage: Respondents (DISAB=1) who answered F21=1
Source: Participation and Activity Limitation Survey, 2001

Variable Name:	**F22C**	*Position:*	1128	*Length:*	2
Collection Name:	AF22C				

Is this DIFFICULTY...due to another reason?

		FREQ	WTD
1	Yes	2,018	352,383
3	No	490	72,811
93	Not applicable	17,337	2,851,825
98	Not stated	855	141,836
R	Refusal	1	19
X	Don't know	9	1,463
		======	=========
		20,710	3,420,337

Coverage: Respondents (DISAB=1) who answered F21=1
Source: Participation and Activity Limitation Survey, 2001

Variable Name:	**F23**	*Position:*	1130	*Length:*	2
Collection Name:	AF23				

How often was this a problem for you (.....)?

		FREQ	WTD
1	Daily	813	133,001
2	Weekly	804	137,147
3	Monthly or less often	835	146,837
4	Never	21	6,369
93	Not applicable	17,337	2,851,825
98	Not stated	863	141,430
R	Refusal	1	190
X	Don't know	36	3,539
		======	=========
		20,710	3,420,338

Coverage: Respondents (DISAB=1) who answered F21=1
Source: Participation and Activity Limitation Survey, 2001

Variable Name:	**F24**	*Position:*	1132	*Length:*	2
Collection Name:	AF24				

When this problem occurred, was it a big problem or a little problem?

		FREQ	WTD
1	Big problem	1,486	253,617
2	Little problem	972	162,821
93	Not applicable	17,358	2,858,194
98	Not stated	874	142,702
R	Refusal	0	0
X	Don't know	20	3,004
		======	=========
		20,710	3,420,338

Coverage: Respondents (DISAB=1) who DID NOT answer F23=4
Source: Participation and Activity Limitation Survey, 2001

Variable Name:	**F25**	*Position:*	1134	*Length:*	2
Collection Name:	AF25				

In the past 12 months, did you (.....) travel locally by specialized bus services, or local public transportation, including buses, subways and taxis?

		FREQ	WTD
1	Yes	6,368	1,066,414
3	No	13,641	2,237,720
93	Not applicable	0	0
98	Not stated	676	111,116
R	Refusal	7	2,408
X	Don't know	18	2,679
		======	=========
		20,710	3,420,337

Coverage: All respondents (DISAB=1)
Source: Participation and Activity Limitation Survey, 2001

Variable Name:	**F26**	*Position:*	1136	*Length:*	2
Collection Name:	AF26				

Were you (Was.....) PREVENTED from travelling locally by specialized bus services, or local public transportation, including buses, subways and taxis?

		FREQ	WTD
1	Yes	795	134,606
3	No	12,705	2,073,139
93	Not applicable	6,393	1,071,501
98	Not stated	783	132,657
R	Refusal	0	0
X	Don't know	34	8,434
		======	=========
		20,710	3,420,337

Coverage: Respondents (DISAB=1) who answered F25=3
Source: Participation and Activity Limitation Survey, 2001

Variable Name:	**F27A**	*Position:*	1138	*Length:*	2
Collection Name:	AF27A				

What PREVENTS you (.....) from travelling locally by specialized bus services, or local public transportation (including buses, subways and taxis): service not available on a 24 hour, 7 day a week basis?

		FREQ	WTD
1	Yes	265	38,884
3	No	473	86,462
93	Not applicable	19,132	3,153,074
98	Not stated	831	140,005
R	Refusal	0	0
X	Don't know	9	1,913
		======	=========
		20,710	3,420,338

Coverage: Respondents (DISAB=1) who answered F26=1
Source: Participation and Activity Limitation Survey, 2001

Variable Name: **F27B** *Position:* 1140 *Length:* 2
Collection Name: AF27B

What PREVENTS you (.....) from travelling locally by specialized bus services, or local public transportation (including buses, subways and taxis): booking rules don't allow for last minute arrangements?

		FREQ	WTD
1	Yes	61	9,951
3	No	662	112,722
93	Not applicable	19,132	3,153,074
98	Not stated	835	140,621
R	Refusal	0	0
X	Don't know	20	3,970
		======	=========
		20,710	3,420,338

Coverage: Respondents (DISAB=1) who answered F26=1
Source: Participation and Activity Limitation Survey, 2001

Variable Name: **F27C** *Position:* 1142 *Length:* 2
Collection Name: AF27C

What PREVENTS you (.....) from travelling locally by specialized bus services, or local public transportation (including buses, subways and taxis): getting to or locating bus stops?

		FREQ	WTD
1	Yes	236	41,830
3	No	504	83,800
93	Not applicable	19,132	3,153,074
98	Not stated	831	139,985
R	Refusal	0	0
X	Don't know	7	1,649
		======	=========
		20,710	3,420,338

Coverage: Respondents (DISAB=1) who answered F26=1
Source: Participation and Activity Limitation Survey, 2001

Variable Name: **F27D** *Position:* 1144 *Length:* 2
Collection Name: AF27D

What PREVENTS you (.....) from travelling locally by specialized bus services, or local public transportation (including buses, subways and taxis): getting on or off vehicles?

		FREQ	WTD
1	Yes	253	49,362
3	No	483	75,888
93	Not applicable	19,132	3,153,074
98	Not stated	835	139,981
R	Refusal	0	0
X	Don't know	7	2,033
		======	=========
		20,710	3,420,338

Coverage: Respondents (DISAB=1) who answered F26=1
Source: Participation and Activity Limitation Survey, 2001

Variable Name: **F27E** *Position:* 1146 *Length:* 2
Collection Name: AF27E

What PREVENTS you (.....) from travelling locally by specialized bus services, or local public transportation (including buses, subways and taxis): seeing signs or notices?

		FREQ	WTD
1	Yes	91	16,521
3	No	647	109,093
93	Not applicable	19,132	3,153,074
98	Not stated	834	140,057
R	Refusal	0	0
X	Don't know	6	1,594
		======	=========
		20,710	3,420,339

Coverage: Respondents (DISAB=1) who answered F26=1
Source: Participation and Activity Limitation Survey, 2001

Variable Name: **F27F** *Position:* 1148 *Length:* 2
Collection Name: AF27F

What PREVENTS you (.....) from travelling locally by specialized bus services, or local public transportation (including buses, subways and taxis): Other?

		FREQ	WTD
1	Yes	440	76,645
3	No	331	54,547
93	Not applicable	19,132	3,153,074
98	Not stated	804	135,820
R	Refusal	0	0
X	Don't know	3	251
		======	=========
		20,710	3,420,337

Coverage: Respondents (DISAB=1) who answered F26=1
Source: Participation and Activity Limitation Survey, 2001

Variable Name: **F28** *Position:* 1150 *Length:* 2
Collection Name: AF28

In the past 12 months, did you (.....) have any DIFFICULTY travelling locally by specialized bus or van services, or local public transportation, because of your (his/her) condition?

		FREQ	WTD
1	Yes	932	179,349
3	No	5,327	869,713
93	Not applicable	13,666	2,242,807
98	Not stated	772	126,656
R	Refusal	1	312
X	Don't know	12	1,502
		======	=========
		20,710	3,420,339

Coverage: Respondents (DISAB=1) who answered F25=1
Source: Participation and Activity Limitation Survey, 2001

Variable Name: **F29A** *Position:* 1152 *Length:* 2
Collection Name: AF29A

What type of difficulty did you (.....) have: service not available on a 24 hour, 7 days a week basis?

		FREQ	WTD
1	Yes	174	27,960
3	No	709	143,082
93	Not applicable	19,006	3,114,333
98	Not stated	815	134,481
R	Refusal	0	0
X	Don't know	6	482
		======	=========
		20,710	3,420,338

Coverage: Respondents (DISAB=1) who answered F28=1
Source: Participation and Activity Limitation Survey, 2001

Variable Name: **F29B** *Position:* 1154 *Length:* 2
Collection Name: AF29B

What type of difficulty did you (.....) have: booking rules don't allow for last minute arrangements

		FREQ	WTD
1	Yes	133	20,789
3	No	746	147,843
93	Not applicable	19,006	3,114,333
98	Not stated	816	136,151
R	Refusal	0	0
X	Don't know	9	1,221
		======	=========
		20,710	3,420,337

Coverage: Respondents (DISAB=1) who answered F28=1
Source: Participation and Activity Limitation Survey, 2001

Variable Name: **F29C** *Position:* 1156 *Length:* 2
Collection Name: AF29C

What type of difficulty did you (.....) have: getting to or locating bus stops?

		FREQ	WTD
1	Yes	240	43,569
3	No	646	127,070
93	Not applicable	19,006	3,114,333
98	Not stated	815	134,927
R	Refusal	0	0
X	Don't know	3	439
		======	=========
		20,710	3,420,338

Coverage: Respondents (DISAB=1) who answered F28=1
Source: Participation and Activity Limitation Survey, 2001

Variable Name: **F29D** *Position:* 1158 *Length:* 2
Collection Name: AF29D

What type of difficulty did you (.....) have: getting on or off vehicles?

		FREQ	WTD
1	Yes	453	96,466
3	No	444	76,546
93	Not applicable	19,006	3,114,333
98	Not stated	805	132,445
R	Refusal	0	0
X	Don't know	2	548
		======	=========
		20,710	3,420,338

Coverage: Respondents (DISAB=1) who answered F28=1
Source: Participation and Activity Limitation Survey, 2001

Variable Name: **F29E** *Position:* 1160 *Length:* 2
Collection Name: AF29E

What type of difficulty did you (.....) have: seeing signs or notices?

		FREQ	WTD
1	Yes	108	18,974
3	No	777	151,384
93	Not applicable	19,006	3,114,333
98	Not stated	818	135,446
R	Refusal	0	0
X	Don't know	1	201
		======	=========
		20,710	3,420,338

Coverage: Respondents (DISAB=1) who answered F28=1
Source: Participation and Activity Limitation Survey, 2001

Variable Name:	**F29F**	*Position:*	1162	*Length:*	2
Collection Name:	AF29F				

What type of difficulty did you (.....) have: Other reason?

		FREQ	WTD
1	Yes	488	91,801
3	No	420	84,441
93	Not applicable	19,006	3,114,333
98	Not stated	795	129,729
R	Refusal	0	0
X	Don't know	1	33
		======	=========
		20,710	3,420,337

Coverage: Respondents (DISAB=1) who answered F28=1
Source: Participation and Activity Limitation Survey, 2001

Variable Name:	**F30**	*Position:*	1164	*Length:*	2
Collection Name:	AF30				

How often was this a problem for you (.....)?

		FREQ	WTD
1	Daily	222	38,485
2	Weekly	265	46,780
3	Monthly or less often	410	84,167
4	Never	13	2,901
93	Not applicable	19,006	3,114,333
98	Not stated	786	132,230
R	Refusal	0	0
X	Don't know	8	1,442
		======	=========
		20,710	3,420,338

Coverage: Respondents (DISAB=1) who answered F28=1
Source: Participation and Activity Limitation Survey, 2001

Variable Name:	**F31**	*Position:* 1166	*Length:* 2
Collection Name:	AF31		

When this problem occurred, was it a big problem or a little problem?

		FREQ	WTD
1	Big problem	540	101,504
2	Little problem	292	54,683
93	Not applicable	19,019	3,117,234
98	Not stated	846	144,803
R	Refusal	0	0
X	Don't know	13	2,114
		======	=========
		20,710	3,420,338

Coverage: Respondents (DISAB=1) who DID NOT answer F30=4
Source: Participation and Activity Limitation Survey, 2001

Variable Name:	**F32**	*Position:* 1168	*Length:* 2
Collection Name:	AF32		

In the past 12 months, did you (.....) take any long distance trips for personal or business reasons?

		FREQ	WTD
1	Yes	13,022	2,041,687
3	No	6,874	1,241,924
93	Not applicable	0	0
98	Not stated	755	124,034
R	Refusal	5	1,891
X	Don't know	54	10,802
		======	=========
		20,710	3,420,338

Coverage: All respondents (DISAB=1)
Source: Participation and Activity Limitation Survey, 2001

Variable Name:	**F33**	*Position:*	1170	*Length:*	2
Collection Name:	AF33				

Are you (Is.....) PREVENTED from travelling long distance?

		FREQ	WTD
1	Yes	1,367	274,640
3	No	5,516	969,025
93	Not applicable	13,022	2,041,687
98	Not stated	790	131,410
R	Refusal	4	1,522
X	Don't know	11	2,054
		======	=========
		20,710	3,420,338

Coverage: Respondents (DISAB=1) who DID NOT answer F32=1
Source: Participation and Activity Limitation Survey, 2001

Variable Name:	**F34A**	*Position:*	1172	*Length:*	2
Collection Name:	AF34A				

What PREVENTS you (.....) from travelling long distance: Flight or ride aggravates your (his / her) condition?

		FREQ	WTD
1	Yes	885	176,003
3	No	412	82,128
93	Not applicable	18,553	3,014,288
98	Not stated	844	145,480
R	Refusal	2	95
X	Don't know	14	2,344
		======	=========
		20,710	3,420,338

Coverage: Respondents (DISAB=1) who answered F33=1
Source: Participation and Activity Limitation Survey, 2001

Variable Name: **F34B** *Position:* 1174 *Length:* 2
Collection Name: AF34B

What PREVENTS you (.....) from travelling long distance: Lack of appropriate transportation to and from terminal or station?

		FREQ	WTD
1	Yes	159	39,206
3	No	1,128	217,294
93	Not applicable	18,553	3,014,288
98	Not stated	849	145,151
R	Refusal	2	95
X	Don't know	19	4,305
		======	=========
		20,710	3,420,339

Coverage: Respondents (DISAB=1) who answered F33=1
Source: Participation and Activity Limitation Survey, 2001

Variable Name: **F34C** *Position:* 1176 *Length:* 2
Collection Name: AF34C

What PREVENTS you (.....) from travelling long distance: Moving around terminal or station?

		FREQ	WTD
1	Yes	439	93,623
3	No	845	161,900
93	Not applicable	18,553	3,014,288
98	Not stated	849	145,892
R	Refusal	2	95
X	Don't know	22	4,540
		======	=========
		20,710	3,420,338

Coverage: Respondents (DISAB=1) who answered F33=1
Source: Participation and Activity Limitation Survey, 2001

Variable Name: **F34D** *Position:* 1178 *Length:* 2
Collection Name: AF34D

What PREVENTS you (.....) from travelling long distance: Boarding or disembarking?

		FREQ	WTD
1	Yes	428	93,237
3	No	846	160,686
93	Not applicable	18,553	3,014,288
98	Not stated	855	146,847
R	Refusal	2	95
X	Don't know	26	5,184
		======	=========
		20,710	3,420,337

Coverage: Respondents (DISAB=1) who answered F33=1
Source: Participation and Activity Limitation Survey, 2001

Variable Name: **F34E** *Position:* 1180 *Length:* 2
Collection Name: AF34E

What PREVENTS you (.....) from travelling long distance: Seating on board?

		FREQ	WTD
1	Yes	470	99,347
3	No	808	156,278
93	Not applicable	18,553	3,014,288
98	Not stated	849	145,822
R	Refusal	2	95
X	Don't know	28	4,507
		======	=========
		20,710	3,420,337

Coverage: Respondents (DISAB=1) who answered F33=1
Source: Participation and Activity Limitation Survey, 2001

Variable Name: **F34F** *Position:* 1182 *Length:* 2
Collection Name: AF34F

What PREVENTS you (.....) from travelling long distance: Seeing signs or notices?

		FREQ	WTD
1	Yes	157	30,606
3	No	1,128	225,184
93	Not applicable	18,553	3,014,288
98	Not stated	854	147,230
R	Refusal	2	95
X	Don't know	16	2,936
		======	=========
		20,710	3,420,339

Coverage: Respondents (DISAB=1) who answered F33=1
Source: Participation and Activity Limitation Survey, 2001

Variable Name:	**F34G**	*Position:*	1184	*Length:*	2
Collection Name:	AF34G				

What PREVENTS you (.....) from travelling long distance: Hearing announcements?

		FREQ	WTD
1	Yes	156	27,685
3	No	1,126	228,613
93	Not applicable	18,553	3,014,288
98	Not stated	856	147,119
R	Refusal	2	95
X	Don't know	17	2,539
		======	=========
		20,710	3,420,339

Coverage: Respondents (DISAB=1) who answered F33=1
Source: Participation and Activity Limitation Survey, 2001

Variable Name:	**F34H**	*Position:*	1186	*Length:*	2
Collection Name:	AF34H				

What PREVENTS you (.....) from travelling long distance: Washroom facilities?

		FREQ	WTD
1	Yes	192	45,250
3	No	1,090	210,487
93	Not applicable	18,553	3,014,288
98	Not stated	852	146,377
R	Refusal	2	95
X	Don't know	21	3,841
		======	=========
		20,710	3,420,338

Coverage: Respondents (DISAB=1) who answered F33=1
Source: Participation and Activity Limitation Survey, 2001

Variable Name:	**F34I**	*Position:*	1188	*Length:*	2
Collection Name:	AF34I				

What PREVENTS you (.....) from travelling long distance: Unsupportive staff?

		FREQ	WTD
1	Yes	100	23,005
3	No	1,152	223,857
93	Not applicable	18,553	3,014,288
98	Not stated	853	146,081
R	Refusal	2	95
X	Don't know	50	13,011
		======	=========
		20,710	3,420,337

Coverage: Respondents (DISAB=1) who answered F33=1
Source: Participation and Activity Limitation Survey, 2001

Variable Name:	**F34J**	*Position:*	1190	*Length:*	2
Collection Name:	AF34J				

What PREVENTS you (.....) from travelling long distance: Transporting wheelchair or other specialized aids?

		FREQ	WTD
1	Yes	146	24,986
3	No	1,134	227,866
93	Not applicable	18,553	3,014,288
98	Not stated	852	145,301
R	Refusal	2	95
X	Don't know	23	7,802
		======	=========
		20,710	3,420,338

Coverage: Respondents (DISAB=1) who answered F33=1
Source: Participation and Activity Limitation Survey, 2001

Variable Name:	**F34K**	*Position:*	1192	*Length:*	2
Collection Name:	AF34K				

What PREVENTS you (.....) from travelling long distance: Too costly?

		FREQ	WTD
1	Yes	540	105,548
3	No	738	146,685
93	Not applicable	18,553	3,014,288
98	Not stated	852	145,238
R	Refusal	2	95
X	Don't know	25	8,485
		======	=========
		20,710	3,420,339

Coverage: Respondents (DISAB=1) who answered F33=1
Source: Participation and Activity Limitation Survey, 2001

Variable Name:	**F34L**	*Position:*	1194	*Length:*	2
Collection Name:	AF34L				

What PREVENTS you (.....) from travelling long distance: Other?

		FREQ	WTD
1	Yes	424	81,232
3	No	868	179,716
93	Not applicable	18,553	3,014,288
98	Not stated	854	143,512
R	Refusal	2	95
X	Don't know	9	1,496
		======	=========
		20,710	3,420,339

Coverage: Respondents (DISAB=1) who answered F33=1
Source: Participation and Activity Limitation Survey, 2001

Variable Name:	**F35A**	*Position:*	1196	*Length:*	2
Collection Name:	AF35A				

In the past 12 months, did you (.....) travel long distance for personal or business reasons by Car?

		FREQ	WTD
1	Yes	11,101	1,706,031
3	No	1,611	287,259
93	Not applicable	6,933	1,254,617
98	Not stated	1,063	172,183
R	Refusal	1	219
X	Don't know	1	29
		======	=========
		20,710	3,420,338

Coverage: Respondents (DISAB=1) who answered F32=1
Source: Participation and Activity Limitation Survey, 2001

Variable Name: **F35B** *Position:* 1198 *Length:* 2
Collection Name: AF35B

In the past 12 months, did you (.....) travel long distance for personal or business reasons by Inter-city bus?

		FREQ	WTD
1	Yes	1,297	191,790
3	No	11,213	1,764,701
93	Not applicable	6,933	1,254,617
98	Not stated	1,259	207,775
R	Refusal	0	0
X	Don't know	8	1,455
		======	=========
		20,710	3,420,338

Coverage: Respondents (DISAB=1) who answered F32=1
Source: Participation and Activity Limitation Survey, 2001

Variable Name: **F35C** *Position:* 1200 *Length:* 2
Collection Name: AF35C

In the past 12 months, did you (.....) travel long distance for personal or business reasons by Train?

		FREQ	WTD
1	Yes	467	100,544
3	No	12,001	1,849,765
93	Not applicable	6,933	1,254,617
98	Not stated	1,303	214,570
R	Refusal	0	0
X	Don't know	6	841
		======	=========
		20,710	3,420,337

Coverage: Respondents (DISAB=1) who answered F32=1
Source: Participation and Activity Limitation Survey, 2001

Variable Name: **F35D** *Position:* 1202 *Length:* 2
Collection Name: AF35D

In the past 12 months, did you (.....) travel long distance for personal or business reasons by Airplane?

		FREQ	WTD
1	Yes	3,610	602,736
3	No	8,962	1,365,058
93	Not applicable	6,933	1,254,617
98	Not stated	1,199	197,451
R	Refusal	0	0
X	Don't know	6	476
		======	=========
		20,710	3,420,338

Coverage: Respondents (DISAB=1) who answered F32=1
Source: Participation and Activity Limitation Survey, 2001

Variable Name: **F36** *Position:* 1204 *Length:* 2
Collection Name: AF36

In the past 12 months, did you (.....) have any DIFFICULTY travelling by bus, train or airplane?

		FREQ	WTD
1	Yes	852	148,201
3	No	3,751	603,544
93	Not applicable	14,825	2,453,523
98	Not stated	1,275	214,443
R	Refusal	0	0
X	Don't know	7	626
		======	=========
		20,710	3,420,337

Coverage: Respondents (DISAB=1) who answered F35B=1 or F35C=1 or F35D=1
Source: Participation and Activity Limitation Survey, 2001

Variable Name:	**F37A**	*Position:*	1206	*Length:*	2
Collection Name:	AF37A				

What kind of DIFFICULTY did you (.....) have travelling by bus, train or airplane: Flight or ride aggravates your (his / her) condition?

		FREQ	WTD
1	Yes	570	98,329
3	No	265	46,709
93	Not applicable	18,583	3,057,693
98	Not stated	1,289	216,940
R	Refusal	1	214
X	Don't know	2	454
		======	=========
		20,710	3,420,339

Coverage: Respondents (DISAB=1) who answered F36=1
Source: Participation and Activity Limitation Survey, 2001

Variable Name:	**F37B**	*Position:*	1208	*Length:*	2
Collection Name:	AF37B				

What kind of DIFFICULTY did you (.....) have travelling by bus, train or airplane: Lack of appropriate transportation to and from terminal or station?

		FREQ	WTD
1	Yes	100	14,796
3	No	720	128,564
93	Not applicable	18,583	3,057,693
98	Not stated	1,304	218,763
R	Refusal	1	214
X	Don't know	2	308
		======	=========
		20,710	3,420,338

Coverage: Respondents (DISAB=1) who answered F36=1
Source: Participation and Activity Limitation Survey, 2001

Variable Name:	**F37C**	*Position:*	1210	*Length:*	2
Collection Name:	AF37C				

What kind of DIFFICULTY did you (.....) have travelling by bus, train or airplane: Moving around terminal or station?

		FREQ	WTD
1	Yes	288	51,065
3	No	538	93,700
93	Not applicable	18,583	3,057,693
98	Not stated	1,299	217,644
R	Refusal	2	236
X	Don't know	0	0
		======	=========
		20,710	3,420,338

Coverage: Respondents (DISAB=1) who answered F36=1
Source: Participation and Activity Limitation Survey, 2001

Variable Name:	**F37D**	*Position:*	1212	*Length:*	2
Collection Name:	AF37D				

What kind of DIFFICULTY did you (.....) have travelling by bus, train or airplane: Boarding or disembarking?

		FREQ	WTD
1	Yes	256	40,801
3	No	566	103,290
93	Not applicable	18,583	3,057,693
98	Not stated	1,303	218,319
R	Refusal	2	236
X	Don't know	0	0
		======	=========
		20,710	3,420,339

Coverage: Respondents (DISAB=1) who answered F36=1
Source: Participation and Activity Limitation Survey, 2001

Variable Name: **F37E** *Position:* 1214 *Length:* 2
Collection Name: AF37E

What kind of DIFFICULTY did you (.....) have travelling by bus, train or airplane: Seating on board?

		FREQ	WTD
1	Yes	475	82,196
3	No	355	63,237
93	Not applicable	18,583	3,057,693
98	Not stated	1,295	216,976
R	Refusal	2	236
X	Don't know	0	0
		======	=========
		20,710	3,420,338

Coverage: Respondents (DISAB=1) who answered F36=1
Source: Participation and Activity Limitation Survey, 2001

Variable Name: **F37F** *Position:* 1216 *Length:* 2
Collection Name: AF37F

What kind of DIFFICULTY did you (.....) have travelling by bus, train or airplane: Seeing signs or notices?

		FREQ	WTD
1	Yes	54	7,972
3	No	767	136,524
93	Not applicable	18,583	3,057,693
98	Not stated	1,303	217,877
R	Refusal	2	236
X	Don't know	1	37
		======	=========
		20,710	3,420,339

Coverage: Respondents (DISAB=1) who answered F36=1
Source: Participation and Activity Limitation Survey, 2001

Variable Name:	**F37G**	*Position:*	1218	*Length:*	2
Collection Name:	AF37G				

What kind of DIFFICULTY did you (.....) have travelling by bus, train or airplane: Hearing announcements?

		FREQ	WTD
1	Yes	92	15,689
3	No	731	128,885
93	Not applicable	18,583	3,057,693
98	Not stated	1,301	217,811
R	Refusal	2	236
X	Don't know	1	24
		======	=========
		20,710	3,420,338

Coverage: Respondents (DISAB=1) who answered F36=1
Source: Participation and Activity Limitation Survey, 2001

Variable Name:	**F37H**	*Position:*	1220	*Length:*	2
Collection Name:	AF37H				

What kind of DIFFICULTY did you (.....) have travelling by bus, train or airplane: Washroom facilities?

		FREQ	WTD
1	Yes	106	16,759
3	No	714	126,771
93	Not applicable	18,583	3,057,693
98	Not stated	1,303	218,387
R	Refusal	2	236
X	Don't know	2	493
		======	=========
		20,710	3,420,339

Coverage: Respondents (DISAB=1) who answered F36=1
Source: Participation and Activity Limitation Survey, 2001

Variable Name:	**F37I**	*Position:*	1222	*Length:*	2
Collection Name:	AF37I				

What kind of DIFFICULTY did you (.....) have travelling by bus, train or airplane: Unsupportive staff?

		FREQ	WTD
1	Yes	70	7,657
3	No	752	136,838
93	Not applicable	18,583	3,057,693
98	Not stated	1,302	217,891
R	Refusal	2	236
X	Don't know	1	24
		======	=========
		20,710	3,420,339

Coverage: Respondents (DISAB=1) who answered F36=1
Source: Participation and Activity Limitation Survey, 2001

Variable Name:	**F37J**	*Position:*	1224	*Length:*	2
Collection Name:	AF37J				

What kind of DIFFICULTY did you (.....) have travelling by bus, train or airplane: Transporting wheelchair or other specialized aids?

		FREQ	WTD
1	Yes	36	4,718
3	No	786	139,153
93	Not applicable	18,583	3,057,693
98	Not stated	1,301	218,212
R	Refusal	2	236
X	Don't know	2	326
		======	=========
		20,710	3,420,338

Coverage: Respondents (DISAB=1) who answered F36=1
Source: Participation and Activity Limitation Survey, 2001

Variable Name:	**F37K**	*Position:*	1226	*Length:*	2
Collection Name:	AF37K				

What kind of DIFFICULTY did you (.....) have travelling by bus, train or airplane: Too costly?

		FREQ	WTD
1	Yes	145	20,441
3	No	669	121,810
93	Not applicable	18,583	3,057,693
98	Not stated	1,310	219,748
R	Refusal	1	214
X	Don't know	2	432
		======	=========
		20,710	3,420,338

Coverage: Respondents (DISAB=1) who answered F36=1
Source: Participation and Activity Limitation Survey, 2001

Variable Name:	**F37L**	*Position:*	1228	*Length:*	2
Collection Name:	AF37L				

What kind of DIFFICULTY did you (.....) have travelling by bus, train or airplane: Other?

		FREQ	WTD
1	Yes	161	28,433
3	No	654	115,671
93	Not applicable	18,583	3,057,693
98	Not stated	1,311	218,327
R	Refusal	1	214
X	Don't know	0	0
		======	=========
		20,710	3,420,338

Coverage: Respondents (DISAB=1) who answered F36=1
Source: Participation and Activity Limitation Survey, 2001

Variable Name:	**F38**	*Position:*	1230	*Length:*	2
Collection Name:	AF38				

In the past 12 months, was long distance travel a problem for you (.....) because of your (his / her) condition?

		FREQ	WTD
1	Yes	4,476	767,009
3	No	9,750	1,511,713
93	Not applicable	5,531	972,601
98	Not stated	898	154,682
R	Refusal	3	1,282
X	Don't know	52	13,050
		======	=========
		20,710	3,420,337

Coverage: Respondents (DISAB=1) who answered F32=1
Source: Participation and Activity Limitation Survey, 2001

Variable Name:	**F39**	*Position:*	1232	*Length:*	2
Collection Name:	AF39				

How often was this a problem for you (.....)?

		FREQ	WTD
1	Daily	835	135,917
2	Weekly	275	50,456
3	Monthly or less often	2,995	504,452
4	Never	123	22,839
93	Not applicable	15,336	2,498,646
98	Not stated	1,057	189,718
R	Refusal	1	190
X	Don't know	88	18,119
		======	=========
		20,710	3,420,337

Coverage: Respondents (DISAB=1) who answered F38=1
Source: Participation and Activity Limitation Survey, 2001

Variable Name:	**F40**	*Position:*	1234	*Length:*	2
Collection Name:	AF40				

When this problem occurred, was it a big problem or a little problem?

		FREQ	WTD
1	Big problem	2,452	434,734
2	Little problem	1,651	257,504
93	Not applicable	15,459	2,521,485
98	Not stated	1,094	197,187
R	Refusal	1	78
X	Don't know	53	9,350
		======	=========
		20,710	3,420,338

Coverage: Respondents (DISAB=1) who DID NOT answer F39=4
Source: Participation and Activity Limitation Survey, 2001

Variable Name:	**F41**	*Position:*	1236	*Length:*	2
Collection Name:	AF41				

In the past 12 months, did you (.....) have any OUT-OF-POCKET or DIRECT EXPENSES for transportation, for example, travel to and from treatment, therapy or other medical or rehabilitation services; or extra expenses due to the need for more expensive transportation? INCLUDE amounts not covered by insurance such as exclusions, deductibles and expenses over limits. EXCLUDE payments for which you have (he / she has) been or will be reimbursed by any insurance or government program.

		FREQ	WTD
1	Yes	5,583	909,539
3	No	14,291	2,370,925
93	Not applicable	0	0
98	Not stated	760	126,557
R	Refusal	9	2,720
X	Don't know	67	10,596
		======	=========
		20,710	3,420,337

Coverage: All respondents (DISAB=1)
Source: Participation and Activity Limitation Survey, 2001

Variable Name:	**F44**	*Position:*	1238	*Length:*	2
Collection Name:	AF44				

Because of your (..... 's) condition, do you (does.....) use any specialized features to enter or leave your (his/her) residence, or inside your (his/her) residence?

		FREQ	WTD
1	Yes	2,226	369,942
3	No	17,608	2,902,623
93	Not applicable	0	0
98	Not stated	846	141,735
R	Refusal	2	956
X	Don't know	28	5,081
		======	=========
		20,710	3,420,337

Coverage: All respondents (DISAB=1)
Source: Participation and Activity Limitation Survey, 2001

Variable Name:	**F45B**	*Position:*	1240	*Length:*	2
Collection Name:	AF45B				

Do you (Does.....) now use automatic or easy to open doors (includes lever handles)?

		FREQ	WTD
1	Yes	543	95,738
3	No	1,569	249,851
93	Not applicable	17,638	2,908,660
98	Not stated	953	159,014
R	Refusal	3	936
X	Don't know	4	6,139
		======	=========
		20,710	3,420,338

Coverage: Respondents (DISAB=1) who answered F44=1
Source: Participation and Activity Limitation Survey, 2001

Variable Name: **F45C** *Position:* 1242 *Length:* 2
Collection Name: AF45C

Do you (Does.....) now use widened doorways or hallways?

		FREQ	WTD
1	Yes	521	80,301
3	No	1,594	270,469
93	Not applicable	17,638	2,908,660
98	Not stated	951	159,879
R	Refusal	3	936
X	Don't know	3	92
		======	=========
		20,710	3,420,337

Coverage: Respondents (DISAB=1) who answered F44=1
Source: Participation and Activity Limitation Survey, 2001

Variable Name: **F45D** *Position:* 1244 *Length:* 2
Collection Name: AF45D

Do you (Does.....) now use elevator or lift device?

		FREQ	WTD
1	Yes	520	97,193
3	No	1,593	253,859
93	Not applicable	17,638	2,908,660
98	Not stated	952	159,200
R	Refusal	3	936
X	Don't know	4	489
		======	=========
		20,710	3,420,337

Coverage: Respondents (DISAB=1) who answered F44=1
Source: Participation and Activity Limitation Survey, 2001

Variable Name: **F45E** *Position:* 1246 *Length:* 2
Collection Name: AF45E

Do you (Does.....) now use visual alarms or audio warning devices?

		FREQ	WTD
1	Yes	279	54,542
3	No	1,806	292,733
93	Not applicable	17,638	2,908,660
98	Not stated	970	162,268
R	Refusal	3	936
X	Don't know	14	1,198
		======	=========
		20,710	3,420,337

Coverage: Respondents (DISAB=1) who answered F44=1
Source: Participation and Activity Limitation Survey, 2001

Variable Name:	**F45F**	*Position:*	1248	*Length:*	2
Collection Name:	AF45F				

Do you (Does.....) now use grab bars or a bath lift (in the bathroom)?

		FREQ	WTD
1	Yes	1,650	272,930
3	No	503	85,476
93	Not applicable	17,638	2,908,660
98	Not stated	915	153,001
R	Refusal	0	0
X	Don't know	4	270
		======	=========
		20,710	3,420,337

Coverage: Respondents (DISAB=1) who answered F44=1
Source: Participation and Activity Limitation Survey, 2001

Variable Name:	**F45G**	*Position:*	1250	*Length:*	2
Collection Name:	AF45G				

Do you (Does.....) now use lowered counters in the kitchen?

		FREQ	WTD
1	Yes	159	22,722
3	No	1,950	329,528
93	Not applicable	17,638	2,908,660
98	Not stated	949	157,440
R	Refusal	3	936
X	Don't know	11	1,051
		======	=========
		20,710	3,420,337

Coverage: Respondents (DISAB=1) who answered F44=1
Source: Participation and Activity Limitation Survey, 2001

Variable Name:	**F45_OTH**	*Position:*	1252	*Length:*	2
Collection Name:	F45_OTH				

Derived variable: Uses ramps/street level entrances or other specialized feature to enter or leave residence

		FREQ	WTD
1	Yes	1,356	226,993
3	No	798	131,791
93	Not applicable	18,484	3,050,396
98	Not stated	66	9,980
R	Refusal	4	973
X	Don't know	2	205
		======	=========
		20,710	3,420,338

Coverage: Respondents (DISAB=1) who answered F44=1
Source: Participation and Activity Limitation Survey, 2001
Note: F45_OTH is derived from variables F45A and F45H.

Variable Name:	**F46**	*Position:*	1254	*Length:*	2
Collection Name:	AF46				

Do you (Does.....) need any other specialized features, which you do (he /she does) not already have?

		FREQ	WTD
1	Yes	314	53,798
3	No	1,853	303,359
93	Not applicable	18,484	3,050,396
98	Not stated	14	3,332
R	Refusal	3	936
X	Don't know	42	8,518
		======	=========
		20,710	3,420,339

Coverage: Respondents (DISAB=1) who answered F44=1
Source: Participation and Activity Limitation Survey, 2001

Variable Name: **F47** *Position:* 1256 *Length:* 2
Collection Name: AF47

Are there any specialized features that you NEED (..... NEEDS) but do not have (does not have)?

		FREQ	WTD
1	Yes	753	128,766
3	No	16,824	2,763,543
93	Not applicable	2,226	369,942
98	Not stated	820	137,619
R	Refusal	4	1,071
X	Don't know	83	19,396
		======	=========
		20,710	3,420,337

Coverage: Respondents (DISAB=1) who DID NOT answer F44=1
Source: Participation and Activity Limitation Survey, 2001

Variable Name: **F48A** *Position:* 1258 *Length:* 2
Collection Name: AF48A

Which specialized features do you (does.....) need, but do(es) not have: Ramps or street level entrances?

		FREQ	WTD
1	Yes	286	52,585
3	No	705	115,377
93	Not applicable	18,809	3,096,822
98	Not stated	909	155,533
R	Refusal	0	0
X	Don't know	1	21
		======	=========
		20,710	3,420,338

Coverage: Respondents (DISAB=1) who answered F46=1 or F47=1
Source: Participation and Activity Limitation Survey, 2001

Variable Name: **F48B** *Position:* 1260 *Length:* 2
Collection Name: AF48B

Which specialized features do you (does.....) need, but do(es) not have: Automatic or easy to open doors (includes lever handles)?

		FREQ	WTD
1	Yes	178	27,945
3	No	801	136,114
93	Not applicable	18,809	3,096,822
98	Not stated	921	159,416
R	Refusal	0	0
X	Don't know	1	40
		======	=========
		20,710	3,420,337

Coverage: Respondents (DISAB=1) who answered F46=1 or F47=1
Source: Participation and Activity Limitation Survey, 2001

Variable Name: **F48C** *Position:* 1262 *Length:* 2
Collection Name: AF48C

Which specialized features do you (does.....) need, but do(es) not have: Widened doorways or hallways?

		FREQ	WTD
1	Yes	117	20,746
3	No	856	140,830
93	Not applicable	18,809	3,096,822
98	Not stated	926	161,715
R	Refusal	0	0
X	Don't know	2	225
		======	=========
		20,710	3,420,338

Coverage: Respondents (DISAB=1) who answered F46=1 or F47=1
Source: Participation and Activity Limitation Survey, 2001

Variable Name: **F48D** *Position:* 1264 *Length:* 2
Collection Name: AF48D

Which specialized features do you (does.....) need, but do(es) not have: Elevator or lift device?

		FREQ	WTD
1	Yes	205	39,624
3	No	780	126,914
93	Not applicable	18,809	3,096,822
98	Not stated	915	156,937
R	Refusal	0	0
X	Don't know	1	40
		======	=========
		20,710	3,420,337

Coverage: Respondents (DISAB=1) who answered F46=1 or F47=1
Source: Participation and Activity Limitation Survey, 2001

Variable Name: **F48E** *Position:* 1266 *Length:* 2
Collection Name: AF48E

Which specialized features do you (does.....) need, but do(es) not have: Visual alarms or audio warning devices?

		FREQ	WTD
1	Yes	82	13,767
3	No	891	149,654
93	Not applicable	18,809	3,096,822
98	Not stated	925	159,435
R	Refusal	0	0
X	Don't know	3	660
		======	=========
		20,710	3,420,338

Coverage: Respondents (DISAB=1) who answered F46=1 or F47=1
Source: Participation and Activity Limitation Survey, 2001

Variable Name:	**F48F**	*Position:*	1268	*Length:*	2
Collection Name:	AF48F				

Which specialized features do you (does.....) need, but do(es) not have: Grab bars or a bath lift (in the bathroom)?

		FREQ	WTD
1	Yes	526	87,446
3	No	473	81,222
93	Not applicable	18,809	3,096,822
98	Not stated	901	154,808
R	Refusal	0	0
X	Don't know	1	40
		======	=========
		20,710	3,420,338

Coverage: Respondents (DISAB=1) who answered F46=1 or F47=1
Source: Participation and Activity Limitation Survey, 2001

Variable Name:	**F48G**	*Position:*	1270	*Length:*	2
Collection Name:	AF48G				

Which specialized features do you (does.....) need, but do(es) not have: Lowered counters in the kitchen?

		FREQ	WTD
1	Yes	99	17,888
3	No	880	146,533
93	Not applicable	18,809	3,096,822
98	Not stated	921	159,054
R	Refusal	0	0
X	Don't know	1	40
		======	=========
		20,710	3,420,337

Coverage: Respondents (DISAB=1) who answered F46=1 or F47=1
Source: Participation and Activity Limitation Survey, 2001

Variable Name: **F48H** *Position:* 1272 *Length:* 2
Collection Name: AF48H

Which specialized features do you (does.....) need, but do(es) not have: Other?

		FREQ	WTD
1	Yes	352	57,749
3	No	653	113,807
93	Not applicable	18,809	3,096,822
98	Not stated	892	151,086
R	Refusal	0	0
X	Don't know	4	873
		======	=========
		20,710	3,420,337

Coverage: Respondents (DISAB=1) who answered F46=1 or F47=1
Source: Participation and Activity Limitation Survey, 2001

Variable Name: **F49A** *Position:* 1274 *Length:* 2
Collection Name: AF49A

Why don't you (doesn't.....) have this (these) feature(s): Not covered by insurance?

		FREQ	WTD
1	Yes	441	77,729
3	No	519	79,449
93	Not applicable	18,809	3,096,822
98	Not stated	892	154,107
R	Refusal	0	0
X	Don't know	49	12,231
		======	=========
		20,710	3,420,338

Coverage: Respondents (DISAB=1) who answered F46=1 or F47=1
Source: Participation and Activity Limitation Survey, 2001

Variable Name: **F49B** *Position:* 1276 *Length:* 2
Collection Name: AF49B

Why don't you (doesn't.....) have this (these) feature(s): Too expensive?

		FREQ	WTD
1	Yes	686	112,680
3	No	298	51,653
93	Not applicable	18,809	3,096,822
98	Not stated	886	153,384
R	Refusal	0	0
X	Don't know	31	5,798
		======	=========
		20,710	3,420,337

Coverage: Respondents (DISAB=1) who answered F46=1 or F47=1
Source: Participation and Activity Limitation Survey, 2001

Variable Name: **F49C** *Position:* 1278 *Length:* 2
Collection Name: AF49C

Why don't you (doesn't.....) have this (these) feature(s): Specialized features not approved or recommended by health professional?

		FREQ	WTD
1	Yes	62	10,562
3	No	876	142,619
93	Not applicable	18,809	3,096,822
98	Not stated	898	155,379
R	Refusal	0	0
X	Don't know	65	14,956
		======	=========
		20,710	3,420,338

Coverage: Respondents (DISAB=1) who answered F46=1 or F47=1
Source: Participation and Activity Limitation Survey, 2001

Variable Name: **F49D** *Position:* 1280 *Length:* 2
Collection Name: AF49D

Why don't you (doesn't.....) have this (these) feature(s): Currently on a waiting list for aids (features)?

		FREQ	WTD
1	Yes	60	9,209
3	No	927	155,032
93	Not applicable	18,809	3,096,822
98	Not stated	896	154,648
R	Refusal	0	0
X	Don't know	18	4,626
		======	=========
		20,710	3,420,337

Coverage: Respondents (DISAB=1) who answered F46=1 or F47=1
Source: Participation and Activity Limitation Survey, 2001

Variable Name: **F49E** *Position:* 1282 *Length:* 2
Collection Name: AF49E

Why don't you (doesn't.....) have this (these) feature(s): Other reason?

		FREQ	WTD
1	Yes	360	61,075
3	No	648	111,355
93	Not applicable	18,809	3,096,822
98	Not stated	883	150,225
R	Refusal	1	230
X	Don't know	9	630
		======	=========
		20,710	3,420,337

Coverage: Respondents (DISAB=1) who answered F46=1 or F47=1
Source: Participation and Activity Limitation Survey, 2001

Variable Name: **F50** *Position:* 1284 *Length:* 2
Collection Name: AF50

Has the design and layout of your (..... 's) home, including entrance and exits, made it difficult to participate in the activities you (he / she) want(s) or need(s) to do? (INCLUDE ALL activities of daily living, not just leisure or recreational activities.)

		FREQ	WTD
1	Yes	806	144,427
3	No	19,071	3,132,812
93	Not applicable	0	0
98	Not stated	789	131,747
R	Refusal	6	2,332
X	Don't know	38	9,019
		======	=========
		20,710	3,420,337

Coverage: All respondents (DISAB=1)
Source: Participation and Activity Limitation Survey, 2001

Variable Name: **F51** *Position:* 1286 *Length:* 2
Collection Name: AF51

In the past 12 months, how often has the design and layout of your (.....'s) home, including entrance and exits, made it difficult to participate in the activities you (he / she) want(s) or need(s) to do? (INCLUDE ALL activities of daily living, not just leisure or recreational activities.)

		FREQ	WTD
1	Daily	500	90,418
2	Weekly	149	23,376
3	Monthly or less often	134	27,106
93	Not applicable	19,115	3,144,164
98	Not stated	804	134,337
R	Refusal	0	0
X	Don't know	8	938
		======	=========
		20,710	3,420,339

Coverage: Respondents (DISAB=1) who answered F50=1
Source: Participation and Activity Limitation Survey, 2001

Variable Name: **F52** *Position:* 1288 *Length:* 2
Collection Name: AF52

When this problem occurred, was it a big problem or a little problem?

		FREQ	WTD
1	Big problem	486	85,780
2	Little problem	283	53,114
93	Not applicable	19,115	3,144,164
98	Not stated	820	136,823
R	Refusal	0	0
X	Don't know	6	457
		======	=========
		20,710	3,420,338

Coverage: Respondents (DISAB=1) who answered F50=1
Source: Participation and Activity Limitation Survey, 2001

Variable Name:	**F53**	*Position:*	1290	*Length:*	2
Collection Name:	AF53				

In the past 12 months, did you (.....) or your (his / her) family living with you (him/ her), have any OUT-OF-POCKET or DIRECT EXPENSES for modifications to your (his / her) residence because of your (his / her) condition? INCLUDE amounts not covered by insurance such as exclusions, deductibles and expenses over limits. EXCLUDE payments for which you have (he / she has) been or will be reimbursed by any insurance or government program.

		FREQ	WTD
1	Yes	1,072	167,828
3	No	18,840	3,122,884
93	Not applicable	0	0
98	Not stated	743	120,479
R	Refusal	7	2,646
X	Don't know	48	6,502
		======	=========
		20,710	3,420,339

Coverage: All respondents (DISAB=1)
Source: Participation and Activity Limitation Survey, 2001

Section: **ECONOMIC CHARACTERISTICS**

Variable Name:	**G1A**	*Position:*	1292	*Length:*	2
Collection Name:	AG1A				

Do you (Does.....) have insurance that covers all or part of the cost of your (his / her) prescription medication?

		FREQ	WTD
1	Yes	15,067	2,505,347
3	No	4,494	730,878
93	Not applicable	0	0
98	Not stated	823	141,582
R	Refusal	13	2,451
X	Don't know	313	40,079
		======	=========
		20,710	3,420,337

Coverage: All respondents (DISAB=1)
Source: Participation and Activity Limitation Survey, 2001

Variable Name: **G1B** *Position:* 1294 *Length:* 2
Collection Name: AG1B

Do you (Does.....) have insurance that covers all or part of the cost of eye glasses or contact lenses?

		FREQ	WTD
1	Yes	10,209	1,609,904
3	No	8,843	1,550,759
93	Not applicable	0	0
98	Not stated	814	133,935
R	Refusal	14	2,580
X	Don't know	830	123,160
		======	=========
		20,710	3,420,338

Coverage: All respondents (DISAB=1)
Source: Participation and Activity Limitation Survey, 2001

Variable Name: **G1C** *Position:* 1296 *Length:* 2
Collection Name: AG1C

Do you (Does.....) have insurance that covers all or part of hospital charges for a private or semi-private room?

		FREQ	WTD
1	Yes	9,823	1,599,456
3	No	8,027	1,382,138
93	Not applicable	0	0
98	Not stated	824	137,554
R	Refusal	14	2,580
X	Don't know	2,022	298,610
		======	=========
		20,710	3,420,338

Coverage: All respondents (DISAB=1)
Source: Participation and Activity Limitation Survey, 2001

Variable Name:	**G2**	*Position:*	1298	*Length:*	2
Collection Name:	AG2				

Did you (.....) claim a Medical Expense Tax Credit with your (his / her) 2000 income tax return?

		FREQ	WTD
1	Yes	4,313	704,243
3	No	12,430	2,031,153
93	Not applicable	0	0
98	Not stated	799	136,930
R	Refusal	27	5,869
X	Don't know	3,141	542,143
		======	=========
		20,710	3,420,338

Coverage: All respondents (DISAB=1)
Source: Participation and Activity Limitation Survey, 2001

Variable Name:	**G3**	*Position:*	1300	*Length:*	2
Collection Name:	AG3				

Did you (.....) receive it?

		FREQ	WTD
1	Yes	3,260	525,188
3	No	463	77,209
93	Not applicable	15,598	2,579,164
98	Not stated	870	150,509
R	Refusal	0	0
X	Don't know	519	88,267
		======	=========
		20,710	3,420,337

Coverage: Respondents (DISAB=1) who answered G2=1
Source: Participation and Activity Limitation Survey, 2001

Variable Name: **G4** *Position:* 1302 *Length:* 2
Collection Name: AG4

Did you (.....), or someone else on your (his / her) behalf, claim the Disability Tax Credit on an income tax return for the year 2000?

		FREQ	WTD
1	Yes	2,254	344,663
3	No	14,901	2,472,236
93	Not applicable	0	0
98	Not stated	734	119,477
R	Refusal	26	5,695
X	Don't know	2,795	478,267
		======	=========
		20,710	3,420,338

Coverage: All respondents (DISAB=1)
Source: Participation and Activity Limitation Survey, 2001

Variable Name: **G5** *Position:* 1304 *Length:* 2
Collection Name: AG5

Did you (.....) receive it?

		FREQ	WTD
1	Yes	1,890	276,836
3	No	199	35,032
93	Not applicable	17,722	2,956,198
98	Not stated	749	122,259
R	Refusal	1	88
X	Don't know	149	29,925
		======	=========
		20,710	3,420,338

Coverage: Respondents (DISAB=1) who answered G4=1
Source: Participation and Activity Limitation Survey, 2001

Variable Name: **G6A** *Position:* 1306 *Length:* 2
Collection Name: AG6A

Why did you (.....) not claim the Disability Tax Credit: You (.....) did not know it existed....?

		FREQ	WTD
1	Yes	5,430	903,249
3	No	9,099	1,512,099
93	Not applicable	5,075	828,626
98	Not stated	798	129,658
R	Refusal	2	416
X	Don't know	306	46,291
		======	=========
		20,710	3,420,339

Coverage: Respondents (DISAB=1) who answered G4=3
Source: Participation and Activity Limitation Survey, 2001

Variable Name: **G6B** *Position:* 1308 *Length:* 2
Collection Name: AG6B

Why did you (.....) not claim the Disability Tax Credit: You (.....) did not think that you (he / she) would meet the eligibility requirements?

		FREQ	WTD
1	Yes	6,444	1,074,340
3	No	2,401	392,482
93	Not applicable	10,505	1,731,874
98	Not stated	1,010	167,062
R	Refusal	2	416
X	Don't know	348	54,163
		======	=========
		20,710	3,420,337

Coverage: Respondents (DISAB=1) who answered G4=3
Source: Participation and Activity Limitation Survey, 2001

Variable Name: **G6C** *Position:* 1310 *Length:* 2
Collection Name: AG6C

Why did you (.....) not claim the Disability Tax Credit: You were (.....was) not able to obtain the disability certificate (Form T2201) from your (his / her) doctor?

		FREQ	WTD
1	Yes	680	100,263
3	No	7,770	1,304,802
93	Not applicable	10,505	1,731,874
98	Not stated	1,232	203,765
R	Refusal	3	509
X	Don't know	520	79,125
		======	=========
		20,710	3,420,338

Coverage: Respondents (DISAB=1) who answered G4=3
Source: Participation and Activity Limitation Survey, 2001

Variable Name: **G6D** *Position:* 1312 *Length:* 2
Collection Name: AG6D

Why did you (.....) not claim the Disability Tax Credit: Other reason?

		FREQ	WTD
1	Yes	1,413	215,690
3	No	7,358	1,239,286
93	Not applicable	10,505	1,731,874
98	Not stated	1,179	192,195
R	Refusal	3	509
X	Don't know	252	40,783
		======	=========
		20,710	3,420,337

Coverage: Respondents (DISAB=1) who answered G4=3
Source: Participation and Activity Limitation Survey, 2001

Variable Name:	**G7A**	*Position:*	1314	*Length:*	2
Collection Name:	AG7A				

Did you (.....), or someone else on your (his / her) behalf, claim the following on an income tax return for the year 2000: Attendant care expenses?

		FREQ	WTD
1	Yes	202	23,390
3	No	18,239	3,017,996
93	Not applicable	0	0
98	Not stated	784	131,791
R	Refusal	28	6,165
X	Don't know	1,457	240,996
		======	=========
		20,710	3,420,338

Coverage: All respondents (DISAB=1)
Source: Participation and Activity Limitation Survey, 2001

Variable Name:	**G7B**	*Position:*	1316	*Length:*	2
Collection Name:	AG7B				

Did you (.....), or someone else on your (his / her) behalf, claim the following on an income tax return for the year 2000: Caregiver amount?

		FREQ	WTD
1	Yes	274	40,267
3	No	18,175	3,001,291
93	Not applicable	0	0
98	Not stated	789	133,325
R	Refusal	28	6,165
X	Don't know	1,444	239,290
		======	=========
		20,710	3,420,338

Coverage: All respondents (DISAB=1)
Source: Participation and Activity Limitation Survey, 2001

Variable Name:	**G7C**	*Position:*	1318	*Length:*	2
Collection Name:	AG7C				

Did you (.....), or someone else on your (his / her) behalf, claim the following on an income tax return for the year 2000: Tuition and education amounts?

		FREQ	WTD
1	Yes	980	117,456
3	No	17,603	2,948,795
93	Not applicable	0	0
98	Not stated	812	137,567
R	Refusal	27	5,871
X	Don't know	1,288	210,648
		======	=========
		20,710	3,420,337

Coverage: All respondents (DISAB=1)
Source: Participation and Activity Limitation Survey, 2001

Variable Name:	**G7D**	*Position:*	1320	*Length:*	2
Collection Name:	AG7D				

Did you (.....), or someone else on your (his / her) behalf, claim the following on an income tax return for the year 2000: Child care expenses?

		FREQ	WTD
1	Yes	566	69,531
3	No	18,092	3,011,452
93	Not applicable	0	0
98	Not stated	805	133,721
R	Refusal	27	5,871
X	Don't know	1,220	199,763
		======	=========
		20,710	3,420,338

Coverage: All respondents (DISAB=1)
Source: Participation and Activity Limitation Survey, 2001

Variable Name: **G7E** *Position:* 1322 *Length:* 2
Collection Name: AG7E

Did you (.....), or someone else on your (his / her) behalf, claim the following on an income tax return for the year 2000: Other?

		FREQ	WTD
1	Yes	195	28,264
3	No	18,297	3,030,722
93	Not applicable	0	0
98	Not stated	894	146,655
R	Refusal	28	6,165
X	Don't know	1,296	208,531
		======	=========
		20,710	3,420,337

Coverage: All respondents (DISAB=1)
Source: Participation and Activity Limitation Survey, 2001

Variable Name: **G8C** *Position:* 1324 *Length:* 2
Collection Name: AG8C

In the year 2000, did you (.....) personally receive income from the following sources: Employment Insurance?

		FREQ	WTD
1	Yes	1,138	146,076
3	No	18,323	3,066,546
93	Not applicable	0	0
98	Not stated	1,048	174,900
R	Refusal	71	14,713
X	Don't know	130	18,103
		======	=========
		20,710	3,420,338

Coverage: All respondents (DISAB=1)
Source: Participation and Activity Limitation Survey, 2001

Variable Name:	**G8D**	*Position:*	1326	*Length:*	2
Collection Name:	AG8D				

In the year 2000, did you (.....) personally receive income from the following sources: Worker's Compensation?

		FREQ	WTD
1	Yes	997	175,505
3	No	18,441	3,022,727
93	Not applicable	0	0
98	Not stated	1,083	189,746
R	Refusal	73	15,281
X	Don't know	116	17,079
		======	=========
		20,710	3,420,338

Coverage: All respondents (DISAB=1)
Source: Participation and Activity Limitation Survey, 2001

Variable Name:	**G8E**	*Position:*	1328	*Length:*	2
Collection Name:	AG8E				

In the year 2000, did you (.....) personally receive income from the following sources: Benefits from Canada or Quebec Pension Plan?

		FREQ	WTD
1	Yes	6,907	1,299,435
3	No	12,511	1,894,678
93	Not applicable	0	0
98	Not stated	1,034	177,383
R	Refusal	71	14,904
X	Don't know	187	33,938
		======	=========
		20,710	3,420,338

Coverage: All respondents (DISAB=1)
Source: Participation and Activity Limitation Survey, 2001

Variable Name: **G8F** *Position:* 1330 *Length:* 2
Collection Name: AG8F

In the year 2000, did you (.....) personally receive income from the following sources: Old Age Security Pension?

		FREQ	WTD
1	Yes	7,212	1,329,039
3	No	12,334	1,890,321
93	Not applicable	0	0
98	Not stated	953	156,947
R	Refusal	72	14,381
X	Don't know	139	29,650
		======	=========
		20,710	3,420,338

Coverage: All respondents (DISAB=1)
Source: Participation and Activity Limitation Survey, 2001

Variable Name: **G8G** *Position:* 1332 *Length:* 2
Collection Name: AG8G

In the year 2000, did you (.....) personally receive income from the following sources: Guaranteed Income Supplement or Spouse's Allowance?

		FREQ	WTD
1	Yes	2,647	439,938
3	No	16,540	2,708,565
93	Not applicable	0	0
98	Not stated	1,082	188,685
R	Refusal	75	16,219
X	Don't know	366	66,931
		======	=========
		20,710	3,420,338

Coverage: All respondents (DISAB=1)
Source: Participation and Activity Limitation Survey, 2001

Variable Name:	**G8H**	*Position:*	1334	*Length:*	2
Collection Name:	AG8H				

In the year 2000, did you (.....) personally receive income from the following sources: Disability pension from the Canada or Quebec Pension Plan?

		FREQ	WTD
1	Yes	1,845	343,016
3	No	17,495	2,827,980
93	Not applicable	0	0
98	Not stated	1,095	196,811
R	Refusal	77	16,326
X	Don't know	198	36,204
		======	=========
		20,710	3,420,337

Coverage: All respondents (DISAB=1)
Source: Participation and Activity Limitation Survey, 2001

Variable Name:	**G8I**	*Position:*	1336	*Length:*	2
Collection Name:	AG8I				

In the year 2000, did you (.....) personally receive income from the following sources: Insurance plans, such as private or employer disability insurance plan or motor vehicle accident insurance?

		FREQ	WTD
1	Yes	1,251	212,172
3	No	18,149	2,977,802
93	Not applicable	0	0
98	Not stated	1,066	182,260
R	Refusal	77	15,633
X	Don't know	167	32,472
		======	=========
		20,710	3,420,339

Coverage: All respondents (DISAB=1)
Source: Participation and Activity Limitation Survey, 2001

Variable Name: **G8J** *Position:* 1338 *Length:* 2
Collection Name: AG8J

In the year 2000, did you (.....) personally receive income from the following sources: Child Tax Benefit?

		FREQ	WTD
1	Yes	1,800	228,962
3	No	17,564	2,959,674
93	Not applicable	0	0
98	Not stated	1,082	187,269
R	Refusal	77	15,633
X	Don't know	187	28,800
		======	=========
		20,710	3,420,338

Coverage: All respondents (DISAB=1)
Source: Participation and Activity Limitation Survey, 2001

Variable Name: **G8K** *Position:* 1340 *Length:* 2
Collection Name: AG8K

In the year 2000, did you (.....) personally receive income from the following sources: Provincial or municipal social assistance or welfare?

		FREQ	WTD
1	Yes	2,339	340,449
3	No	17,042	2,843,660
93	Not applicable	0	0
98	Not stated	1,099	194,840
R	Refusal	77	15,633
X	Don't know	153	25,755
		======	=========
		20,710	3,420,337

Coverage: All respondents (DISAB=1)
Source: Participation and Activity Limitation Survey, 2001

Variable Name: **G8L** *Position:* 1342 *Length:* 2
Collection Name: AG8L

In the year 2000, did you (.....) personally receive income from the following sources: Other income, such as retirement pensions, dividends and interest on bonds, deposits and savings; alimony, child support, scholarships, etc?

		FREQ	WTD
1	Yes	5,600	1,051,545
3	No	13,789	2,141,115
93	Not applicable	0	0
98	Not stated	1,016	176,923
R	Refusal	84	16,939
X	Don't know	221	33,815
		======	=========
		20,710	3,420,337

Coverage: All respondents (DISAB=1)
Source: Participation and Activity Limitation Survey, 2001

Variable Name: **G8M** *Position:* 1344 *Length:* 2
Collection Name: AG8M

In the year 2000, did you (.....) personally receive income from the following sources: Other, for example, Veteran's Disability Pension or Allowance, federal or provincial assistance not mentioned above, etc.)?

		FREQ	WTD
1	Yes	1,443	219,574
3	No	17,889	2,960,034
93	Not applicable	0	0
98	Not stated	1,132	190,754
R	Refusal	80	15,839
X	Don't know	166	34,137
		======	=========
		20,710	3,420,338

Coverage: All respondents (DISAB=1)
Source: Participation and Activity Limitation Survey, 2001

Section: **DERIVED/CENSUS VARIABLES**

Variable Name: **AGILIM** *Position:* 1346 *Length:* 2
Collection Name: AGILIM

Derived variable: Flag to indicate an agility disability.

		FREQ	WTD
1	Yes	12,873	2,277,070
3	No	7,637	1,108,594
4	Undetermined	60	15,103
98	Not stated	140	19,570
		======	=========
		20,710	3,420,337

Coverage: All respondents (DISAB=1)
Source: Participation and Activity Limitation Survey, 2001
Note: AGILIM is a derived variable. A value=1 in this field indicates that this person has an agility disability.

Variable Name: **ATTENDRP** *Position:* 1348 *Length:* 1
Collection Name: ATTENDRP

Derived variable: School attendance

		FREQ	WTD
1	None	0	0
2	Yes, part-time, day or evening	0	0
3	Yes, full-time	0	0
9	Not applicable	20,710	3,420,338
		======	=========
		20,710	3,420,338

Coverage: Respondents (DISAB=0) aged 15-64
Source: 2001 Census of Population
Note: This variable is available only for non-disabled persons (DISAB=0). It refers to either full-time or part-time attendance at school, college or university (courses used as credits towards a certificate, diploma or degree only) during the nine-month period between September 2000 and May 15, 2001.

Variable Name:	**BUILT**	*Position:*	1349	*Length:*	2
Collection Name:	BUILT				

Period dwelling constructed

		FREQ	WTD
01	Built in 1920 or before	1,644	286,380
02	Built during period 1921-1945	1,657	298,440
03	Built during period 1946-1960	3,468	603,995
04	Built during period 1961-1970	3,379	586,846
05	Built during period 1971-1980	4,563	686,180
06	Built during period 1981-1985	1,793	275,612
07	Built during period 1986-1990	1,550	255,403
08	Built during period 1991-1995	1,339	230,234
09	Built during period 1996-2001	1,075	179,428
10	Invalid data	242	17,819
		======	=========
		20,710	3,420,337

Coverage: All respondents
Source: 2001 Census of Population
Note: BUILT refers to the period in which the building was originally built, not the time of any later remodelling, additions or conversions. Respondents were asked to indicate the period of construction, to the best of their knowledge.

Variable Name:	**CFAMSTR**	*Position:*	1351	*Length:*	1
Collection Name:	CFAMSTR				

Derived variable: Census family status

		FREQ	WTD
1	Spouses	9,222	1,684,824
2	Common-law partners	1,242	184,865
3	Lone parents	1,401	269,044
4	Children	2,903	210,735
5	Non-family persons	5,700	1,053,050
9	Invalid data	242	17,819
		======	=========
		20,710	3,420,337

Coverage: All respondents
Source: 2001 Census of Population

Variable Name: **CFINCR** *Position:* 1352 *Length:* 2
Collection Name: CFINCR

Derived variable: Census family income

		FREQ	WTD
01	Less than $5,000	855	130,277
02	$5,000 - $9,999	966	153,126
03	$10,000 - $14,999	2,239	389,754
04	$15,000 - $19,999	1,868	302,613
05	$20,000 - $29,999	3,478	606,580
06	$30,000 - $39,999	2,657	431,404
07	$40,000 - $49,999	2,053	326,892
08	$50,000 - $59,999	1,589	252,176
09	$60,000 - $79,999	2,224	366,361
10	$80,000 or more	2,531	441,673
11	Invalid data	250	19,483
		======	=========
		20,710	3,420,339

Coverage: All respondents
Source: 2001 Census of Population

Variable Name: **CHDNUMBER** *Position:* 1354 *Length:* 1
Collection Name: CHDNUMBER

Derived variable: Number of children

		FREQ	WTD
1	No child	15,034	2,465,280
2	1 child	2,868	519,981
3	2 children	1,796	302,955
4	3 or more children	749	113,012
9	Invalid data	263	19,110
		======	=========
		20,710	3,420,338

Coverage: All respondents aged 15 and over
Source: 2001 Census of Population

Variable Name:	**COM_LAW**	*Position:*	1355	*Length:*	1
Collection Name:	COM_LAW				

Common law status

		FREQ	WTD
1	Not in a common-law relationship	19,425	3,228,982
2	In a common-law relationship	1,246	185,344
9	Invalid data	39	6,012
		======	=========
		20,710	3,420,338

Coverage: All respondents
Source: 2001 Census of Population

Variable Name:	**COSTAIDS**	*Position:*	1356	*Length:*	2
Collection Name:	COSTAIDS				

Derived variable: Out-of-pocket expenses for specialized aids

		FREQ	WTD
1	Less than $200	1,266	223,456
2	$200 to less than $500	639	119,318
3	$500 to less than $1,000	315	56,443
4	$1,000 to less than $2,000	184	33,218
5	$2,000 to less than $5,000	156	33,275
6	$5,000 or more	37	5,492
93	Not applicable	15,855	2,564,641
98	Not stated	2,181	371,035
R	Refusal	0	0
X	Don't know	77	13,459
		======	=========
		20,710	3,420,337

Coverage: All respondents (DISAB=1), who answered B126=1
Source: Participation and Activity Limitation Survey, 2001
Note: COSTAIDS is derived from variables B127 and B128.

Variable Name:	**COSTHELP**	*Position:*	1358	*Length:*	2
Collection Name:	COSTHELP				

Derived variable: Out-of-pocket expenses for help with everyday activities

		FREQ	WTD
1	Less than $200	505	79,992
2	$200 to less than $500	775	144,728
3	$500 to less than $1,000	657	112,356
4	$1,000 to less than $2,000	525	103,357
5	$2,000 to less than $5,000	358	66,574
6	$5,000 or more	229	33,165
93	Not applicable	17,197	2,802,197
98	Not stated	165	29,150
R	Refusal	15	3,984
X	Don't know	284	44,835
		======	=========
		20,710	3,420,338

Coverage: All respondents (DISAB=1), who answered C40B=1 and (C41=2 or 3)
Source: Participation and Activity Limitation Survey, 2001
Note: COSTHELP is derived from variables C43 and C44.

Variable Name:	**COSTHLTH**	*Position:*	1360	*Length:*	2
Collection Name:	COSTHLTH				

Derived variable: Out-of-pocket expenses for health care services

		FREQ	WTD
1	Less than $200	1,472	272,378
2	$200 to less than $500	1,076	187,453
3	$500 to less than $1,000	600	112,044
4	$1,000 to less than $2,000	286	46,853
5	$2,000 to less than $5,000	177	28,876
6	$5,000 or more	66	8,622
93	Not applicable	16,699	2,706,563
98	Not stated	258	45,578
R	Refusal	1	213
X	Don't know	75	11,758
		======	=========
		20,710	3,420,338

Coverage: All respondents (DISAB=1), who answered C48=1
Source: Participation and Activity Limitation Survey, 2001
Note: COSTHLTH is derived from variables C49 and C50.

Variable Name: **COSTMED** *Position:* 1362 *Length:* 2
Collection Name: COSTMED

Derived variable: Out-of-pocket expenses for medication

		FREQ	WTD
1	Less than $100	2,304	401,774
2	$100 to less than $200	2,289	434,232
3	$200 to less than $500	3,382	577,437
4	$500 to less than $1,000	1,962	326,236
5	$1,000 to less than $2,000	1,297	192,936
6	$2,000 to less than $5,000	517	75,587
7	$5,000 or more	104	15,637
93	Not applicable	7,756	1,205,835
98	Not stated	627	106,554
R	Refusal	2	252
X	Don't know	470	83,858
		======	=========
		20,710	3,420,338

Coverage: All respondents (DISAB=1), who answered B118=1
Source: Participation and Activity Limitation Survey, 2001
Note: COSTMED is derived from variables B119 and B120.

Variable Name: **COSTRMOD** *Position:* 1364 *Length:* 2
Collection Name: COSTRMOD

Derived variable: Out-of-pocket expenses for modifications to residence

		FREQ	WTD
1	Less than $100	300	44,374
2	$100 to less than $200	103	20,714
3	$200 to less than $500	141	23,871
4	$500 to less than $1,000	105	14,379
5	$1,000 to less than $2,000	106	15,558
6	$2,000 to less than $5,000	136	19,421
7	$5,000 or more	140	20,936
93	Not applicable	18,895	3,132,031
98	Not stated	760	123,849
R	Refusal	0	0
X	Don't know	24	5,205
		======	=========
		20,710	3,420,338

Coverage: All respondents (DISAB=1), who answered F53=1
Source: Participation and Activity Limitation Survey, 2001
Note: COSTRMOD is derived from variables F54 and F55.

Variable Name:	**COSTTRAN**	*Position:*	1366	*Length:*	2
Collection Name:	COSTTRAN				

Derived variable: Out-of-pocket expenses for transportation

		FREQ	WTD
1	Less than $100	1,585	275,302
2	$100 to less than $200	1,279	211,629
3	$200 to less than $500	1,341	218,606
4	$500 to less than $1,000	604	89,681
5	$1,000 to less than $2,000	343	53,217
6	$2,000 to less than $5,000	147	19,407
7	$5,000 or more	41	4,868
93	Not applicable	14,367	2,384,241
98	Not stated	797	131,891
R	Refusal	1	312
X	Don't know	205	31,185
		======	=========
		20,710	3,420,339

Coverage: All respondents (DISAB=1), who answered F41=1
Source: Participation and Activity Limitation Survey, 2001
Note: COSTTRAN is derived from variables F42 and F43.

Variable Name:	**COWD**	*Position:*	1368	*Length:*	1
Collection Name:	COWD				

Derived variable: Class of worker

		FREQ	WTD
1	Working without pay in family business or farm	0	0
2	Not applicable	20,710	3,420,338
3	Paid worker (self-employed, w/o paid help, incorporated)	0	0
4	Paid worker (self-employed, with paid help, incorporated)	0	0
5	Working for wages, salary, tips or commission	0	0
6	Self-employed, w/o paid help, not incorporated	0	0
7	Self-employed, with paid help, not incorporated	0	0
		======	=========
		20,710	3,420,338

Coverage: Respondents (DISAB=0) aged 15-64, who worked since January 1, 2000
Source: 2001 Census of Population
Note: This derived variable classifies persons who worked since January 1, 2000 into unpaid, paid and self-employed workers (with or without paid help, incorporated or not). It is available only for non-disabled persons (DISAB=0).

Variable Name:	**DEG_AGILP**	*Position:* 1369	*Length:* 2
Collection Name:	DEG_AGILP		

Derived variable: Severity Scale - Agility

		FREQ	WTD
0	No disability	7,837	1,143,268
1	Less severe	11,075	1,943,394
2	More severe	1,798	333,676
		======	=========
		20,710	3,420,338

Coverage: All respondents (DISAB=1)
Source: Participation and Activity Limitation Survey, 2001

Note: DEG_AGILP is a derived variable constructed on the basis of an individual's responses to the agility screening questions in Section B of the questionnaire. It represents a score of the respondent's degree of severity of agility disability. The levels of severity are: less severe and more severe. Please refer to Appendix F for a description of the methodology used to construct all adult severity scales.

Variable Name:	**DEG_HEARP**	*Position:* 1371	*Length:* 2
Collection Name:	DEG_HEARP		

Derived variable: Severity Scale - Hearing

		FREQ	WTD
0	No disability	14,810	2,382,263
1	Less severe	4,109	729,936
2	More severe	1,791	308,139
		======	=========
		20,710	3,420,338

Coverage: All respondents (DISAB=1)
Source: Participation and Activity Limitation Survey, 2001

Note: DEG_HEARP is a derived variable constructed on the basis of an individual's responses to the hearing screening questions in Section B of the questionnaire. It represents a score of the respondent's degree of severity of hearing disability. The levels of severity are: less severe and more severe. Please refer to Appendix F for a description of the methodology used to construct all adult severity scales.

Variable Name:	**DEG_MOBP**	*Position:*	1373	*Length:*	2
Collection Name:	DEG_MOBP				

Derived variable: Severity Scale - Mobility

		FREQ	WTD
0	No disability	6,856	968,790
1	Less severe	9,012	1,566,798
2	More severe	4,842	884,750
		======	=========
		20,710	3,420,338

Coverage: All respondents (DISAB=1)
Source: Participation and Activity Limitation Survey, 2001
Note: DEG_MOBP is a derived variable constructed on the basis of an individual's responses to the mobility screening questions in Section B of the questionnaire. It represents a score of the respondent's degree of severity of mobility disability. The levels of severity are: less severe and more severe. Please refer to Appendix F for a description of the methodology used to construct all adult severity scales.

Variable Name:	**DEG_OTHEP**	*Position:*	1375	*Length:*	2
Collection Name:	DEG_OTHEP				

Derived variable: Severity Scale - Other disability

		FREQ	WTD
0	No disability	13,732	2,369,764
1	Less severe	3,624	502,364
2	More severe	3,354	548,210
		======	=========
		20,710	3,420,338

Coverage: All respondents (DISAB=1)
Source: Participation and Activity Limitation Survey, 2001
Note: DEG_OTHEP is a derived variable constructed on the basis of an individual's responses to the learning, developmental, memory, psychological and unknown disability screening questions in Sections A and B of the questionnaire. It represents a score of the respondent's degree of severity of other disabilities. The levels of severity are: less severe and more severe. Please refer to Appendix F for a description of the methodology used to construct all adult severity scales.

Variable Name: **DEG_PAINP** *Position:* 1377 *Length:* 2
Collection Name: DEG_PAINP

Derived variable: Severity Scale - Pain

		FREQ	WTD
0	No disability	6,832	1,043,422
1	Less severe	6,385	1,047,421
2	More severe	7,493	1,329,494
		======	=========
		20,710	3,420,337

Coverage: All respondents (DISAB=1)
Source: Participation and Activity Limitation Survey, 2001

Note: DEG_PAINP is a derived variable constructed on the basis of an individual's responses to the pain-related disability screening questions in Section B of the questionnaire. It represents a score of the respondent's degree of severity of pain-related disability. The levels of severity are: less severe and more severe. Please refer to Appendix F for a description of the methodology used to construct all adult severity scales.

Variable Name: **DEG_SEEP** *Position:* 1379 *Length:* 2
Collection Name: DEG_SEEP

Derived variable: Severity Scale - Seeing

		FREQ	WTD
0	No disability	17,154	2,826,777
1	Less severe	2,749	445,591
2	More severe	807	147,970
		======	=========
		20,710	3,420,338

Coverage: All respondents (DISAB=1)
Source: Participation and Activity Limitation Survey, 2001

Note: DEG_SEEP is a derived variable constructed on the basis of an individual's responses to the seeing screening questions in Section B of the questionnaire. It represents a score of the respondent's degree of severity of seeing disability. The levels of severity are: less severe and more severe. Please refer to Appendix F for a description of the methodology used to construct all adult severity scales.

Variable Name:	**DEG_SPCHP**	*Position:*	1381	*Length:*	2
Collection Name:	DEG_SPCHP				

Derived variable: Severity Scale - Speech

		FREQ	WTD
0	No disability	18,168	3,057,741
1	Less severe	1,084	158,485
2	More severe	1,458	204,112
		======	=========
		20,710	3,420,338

Coverage: All respondents (DISAB=1)
Source: Participation and Activity Limitation Survey, 2001
Note: DEG_SPCHP is a derived variable constructed on the basis of an individual's responses to the speech screening questions in Section B of the questionnaire. It represents a score of the respondent's degree of severity of speech disability. The levels of severity are: less severe and more severe. Please refer to Appendix F for a description of the methodology used to construct all adult severity scales.

Variable Name:	**DEGREE**	*Position:*	1383	*Length:*	2
Collection Name:	DEGREE				

Derived variable: Severity Scale - Global

		FREQ	WTD
1	Mild	7,391	1,165,470
2	Moderate	5,233	855,274
3	Severe	5,377	919,364
4	Very severe	2,709	480,230
		======	=========
		20,710	3,420,338

Coverage: All respondents (DISAB=1)
Source: Participation and Activity Limitation Survey, 2001
Note: DEGREE is a derived variable. This index for measuring the severity of disability was constructed on the basis of an individual's responses to the filter questions (Section A) and screening questions (Section B) of the questionnaire. It represents a score of the respondent's degree of severity of disability over all natures of disability, i.e. degree is independent of the variables of nature of disability. The levels of severity are: mild, moderate, severe and very severe. Please refer to Appendix F for a description of the methodology used to construct the adult global severity index.

Variable Name:	**DGMFSP**	*Position:* 1385	*Length:* 2
Collection Name:	DGMFSP		

Derived variable: Major field of study

		FREQ	WTD
01	No postsecondary qualifications	8,841	1,344,044
02	Educational, recreational and counselling services	449	66,986
03	Fine and applied arts	223	32,965
04	Humanities and related fields	233	47,649
05	Social sciences and related fields	288	58,233
06	Commerce, management and business administration	865	111,578
07	Agricultural, biological, nutritional, and food sciences	204	30,911
09	Applied science technologies and trades	1,091	155,709
10	Health professions and related technologies	484	84,343
11	Sciences, engineering and computers	149	24,231
12	No specialization	6	1,243
91	Invalid data	62	7,636
93	Not applicable	7,815	1,454,809
		======	=========
		20,710	3,420,337

Coverage: All respondents aged 15-64
Source: 2001 Census of Population

Note: Major categories of "Engineering and applied sciences" and "Mathematics and physical sciences" are grouped under "Sciences, engineering and computers".

Variable Name:	**DTYPE**	*Position:* 1387	*Length:* 1
Collection Name:	DTYPE		

Structural type of dwelling

		FREQ	WTD
1	Single detached or other single attached house	13,294	2,026,794
2	Semi-detached or double house	839	169,249
3	Row/apt in duplex/apt in building < 5 storeys	4,775	845,935
4	Apartment in building > 4 storeys	1,150	313,280
5	Mobile or other moveable dwelling	410	47,261
6	Invalid data	242	17,819
		======	=========
		20,710	3,420,338

Coverage: All respondents
Source: 2001 Census of Population

Note: DTYPE refers to the structural characteristics and/or dwelling configuration, that is, whether the dwelling is a single-detached house, an apartment in a high-rise building, a row house, a mobile home, etc.

Variable Name:	**EFAMSTR**	*Position:*	1388	*Length:*	1
Collection Name:	EFAMSTR				

Derived variable: Economic family status

		FREQ	WTD
1	Spouse or partner	10,282	1,832,360
2	Lone parent	1,299	253,306
3	Not applicable	203	11,808
4	Other members of family	1,023	201,231
5	Children	2,952	219,490
6	Unattached	4,912	896,132
9	Invalid data	39	6,012
		======	=========
		20,710	3,420,339

Coverage: All respondents
Source: 2001 Census of Population

Variable Name:	**EMPINR**	*Position:*	1389	*Length:*	2
Collection Name:	EMPINR				

Derived variable: Employment income

		FREQ	WTD
00	No income	5,757	924,491
01	Less than $5,000	1,617	173,408
02	$5,000 - $9,999	1,006	119,201
03	$10,000 - $14,999	803	108,198
04	$15,000 - $19,999	618	80,132
05	$20,000 - $29,999	1,010	167,917
06	$30,000 - $39,999	785	131,996
07	$40,000 - $49,999	558	95,379
08	$50,000 - $59,999	301	64,034
09	$60,000 - $79,999	277	65,153
10	$80,000 or more	140	34,732
11	Not applicable	7,778	1,448,393
91	Invalid data	60	7,303
		======	=========
		20,710	3,420,337

Coverage: All respondents aged 15-64
Source: 2001 Census of Population

Variable Name:	**HEARLIM**	*Position:* 1391	*Length:* 2
Collection Name:	HEARLIM		

Derived variable: Flag to indicate a hearing disability

		FREQ	WTD
1	Yes	5,900	1,038,075
3	No	14,450	2,325,025
4	Undetermined	40	5,135
98	Not stated	320	52,103
		======	=========
		20,710	3,420,338

Coverage: All respondents (DISAB=1)
Source: Participation and Activity Limitation Survey, 2001
Note: HEARLIM is a derived variable. A value=1 in this field indicates that this person has a hearing disability.

Variable Name:	**HHINCR**	*Position:* 1393	*Length:* 2
Collection Name:	HHINCR		

Derived variable: Household total income

		FREQ	WTD
01	Less than $5,000	773	106,341
02	$5,000 - $9,999	743	118,271
03	$10,000 - $14,999	1,784	303,686
04	$15,000 - $19,999	1,638	256,368
05	$20,000 - $29,999	3,327	574,517
06	$30,000 - $39,999	2,715	433,054
07	$40,000 - $49,999	2,173	351,841
08	$50,000 - $59,999	1,782	283,893
09	$60,000 - $79,999	2,549	414,953
10	$80,000 or more	3,187	571,402
91	Invalid data	39	6,012
		======	=========
		20,710	3,420,338

Coverage: All respondents
Source: 2001 Census of Population

Variable Name:	**HLNBP**	*Position:*	1395	*Length:*	1
Collection Name:	HLNBP				

Derived variable: Home language

		FREQ	WTD
1	English	16,845	2,553,316
2	French	2,581	505,286
3	Other	1,245	355,724
9	Invalid data	39	6,012
		======	=========
		20,710	3,420,338

Coverage: All respondents
Source: 2001 Census of Population

Variable Name:	**HLSA**	*Position:*	1396	*Length:*	1
Collection Name:	HLSA				

Derived variable: Highest level of educational attainment

		FREQ	WTD
1	Less than high school	5,012	728,415
2	High school	3,113	453,049
3	Trades certificate or diploma	1,598	248,252
4	College	1,873	310,861
5	University	1,276	224,066
6	Not applicable	7,778	1,448,393
9	Invalid data	60	7,303
		======	=========
		20,710	3,420,339

Coverage: All respondents aged 15-64
Source: 2001 Census of Population

Variable Name:	**HMAIN**	*Position:*	1397	*Length:*	1
Collection Name:	HMAIN				

Derived variable: Household maintainer

		FREQ	WTD
1	Person is not primary maintainer	9,480	1,388,994
2	Invalid data	242	17,819
3	Person is primary maintainer	10,988	2,013,524
		======	=========
		20,710	3,420,337

Coverage: All respondents
Source: 2001 Census of Population

Variable Name:	**HOURS**	*Position:*	1398	*Length:*	3
Collection Name:	HOURS				

Derived variable: Hours worked in reference week

Allowed values: 001 : 065

		FREQ	WTD
-1	Invalid data	0	0
-3	Not applicable	20,710	3,420,338
0	0 hours	0	0
001 : 065	1-65 hours	0	0
66	66 hours or more	0	0
		======	=========
		20,710	3,420,338

Coverage: Respondents (DISAB=0) aged 15-64
Source: 2001 Census of Population

Note: This derived variable represents the hours worked in the week prior to enumeration. This variable is available only for the non-disabled population (DISAB=0). For disabled persons (DISAB=1), the PALS variable E1HRS represents the hours worked in the week prior to enumeration. Possible values for HOURS and E1HRS are between 01 and 66, with value 66 including 66 or more hours worked in the reference week. Users should note that the reference week is the week preceding enumeration and therefore, the time frame for PALS and Census differs.

Variable Name:	**ICD9_1**	*Position:*	1401	*Length:*	1
Collection Name:	ICD9_1				

Derived variable: Underlying condition - Mental retardation or mental disorders

		FREQ	WTD
1	Yes	2,296	328,739
3	No	18,414	3,091,599
		======	=========
		20,710	3,420,338

Coverage: All respondents (DISAB=1)
Source: Participation and Activity Limitation Survey, 2001

Note: ICD9_1 is derived from responses to questions B103 and B108 on the questionnaire. Each write-in response could be coded to a maximum of three codes in the International Classification of Diseases - 9th revision - 1975 (ICD9), for a total of 6 possible ICD9 codes for each respondent. A value=1 for ICD9_1 indicates that this person reported "Mental Retardation or Mental Disorders" as an underlying condition of their disability in question B103 or B108.

Variable Name: **ICD9_2** *Position:* 1402 *Length:* 1
Collection Name: ICD9_2

Derived variable: Underlying condition - Sight disorders

		FREQ	WTD
1	Yes	998	160,157
3	No	19,712	3,260,181
		======	=========
		20,710	3,420,338

Coverage: All respondents (DISAB=1)
Source: Participation and Activity Limitation Survey, 2001
Note: ICD9_2 is derived from responses to questions B103 and B108 on the questionnaire. Each write-in response could be coded to a maximum of three codes in the International Classification of Diseases - 9th revision - 1975 (ICD9), for a total of 6 possible ICD9 codes for each respondent. A value=1 for ICD9_2 indicates that this person reported "Sight Disorders" as an underlying condition of their disability in question B103 or B108.

Variable Name: **ICD9_3** *Position:* 1403 *Length:* 1
Collection Name: ICD9_3

Derived variable: Underlying condition - Hearing disorders

		FREQ	WTD
1	Yes	2,025	335,475
3	No	18,685	3,084,863
		======	=========
		20,710	3,420,338

Coverage: All respondents (DISAB=1)
Source: Participation and Activity Limitation Survey, 2001
Note: ICD9_3 is derived from responses to questions B103 and B108 on the questionnaire. Each write-in response could be coded to a maximum of three codes in the International Classification of Diseases - 9th revision - 1975 (ICD9), for a total of 6 possible ICD9 codes for each respondent. A value=1 for ICD9_3 indicates that this person reported "Hearing Disorders" as an underlying condition of their disability in question B103 or B108.

Variable Name: **ICD9_4** *Position:* 1404 *Length:* 1
Collection Name: ICD9_4

Derived variable: Underlying condition - Other disorders of the nervous system

		FREQ	WTD
1	Yes	1,473	219,271
3	No	19,237	3,201,067
		======	=========
		20,710	3,420,338

Coverage: All respondents (DISAB=1)
Source: Participation and Activity Limitation Survey, 2001
Note: ICD9_4 is derived from responses to questions B103 and B108 on the questionnaire. Each write-in response could be coded to a maximum of three codes in the International Classification of Diseases - 9th revision - 1975 (ICD9), for a total of 6 possible ICD9 codes for each respondent. A value=1 for ICD9_4 indicates that this person reported "Other Disorders of the Nervous System" as an underlying condition of their disability in question B103 or B108.

Variable Name:	**ICD9_5**	*Position:*	1405	*Length:*	1
Collection Name:	ICD9_5				

Derived variable: Underlying condition - Hypertensive disease

		FREQ	WTD
1	Yes	291	69,224
3	No	20,419	3,351,114
		======	=========
		20,710	3,420,338

Coverage: All respondents (DISAB=1)
Source: Participation and Activity Limitation Survey, 2001
Note: ICD9_5 is derived from responses to questions B103 and B108 on the questionnaire. Each write-in response could be coded to a maximum of three codes in the International Classification of Diseases - 9th revision - 1975 (ICD9), for a total of 6 possible ICD9 codes for each respondent. A value=1 for ICD9_5 indicates that this person reported "Hypertensive Disease" as an underlying condition of their disability in question B103 or B108.

Variable Name:	**ICD9_6**	*Position:*	1406	*Length:*	1
Collection Name:	ICD9_6				

Derived variable: Underlying condition - Ischaemic heart disease

		FREQ	WTD
1	Yes	373	69,121
3	No	20,337	3,351,217
		======	=========
		20,710	3,420,338

Coverage: All respondents (DISAB=1)
Source: Participation and Activity Limitation Survey, 2001
Note: ICD9_6 is derived from responses to questions B103 and B108 on the questionnaire. Each write-in response could be coded to a maximum of three codes in the International Classification of Diseases - 9th revision - 1975 (ICD9), for a total of 6 possible ICD9 codes for each respondent. A value=1 for ICD9_6 indicates that this person reported "Ischaemic Heart Disease" as an underlying condition of their disability in question B103 or B108.

Variable Name:	**ICD9_7**	*Position:*	1407	*Length:*	1
Collection Name:	ICD9_7				

Derived variable: Underlying condition - Other heart conditions

		FREQ	WTD
1	Yes	1,263	244,226
3	No	19,447	3,176,111
		======	=========
		20,710	3,420,337

Coverage: All respondents (DISAB=1)
Source: Participation and Activity Limitation Survey, 2001
Note: ICD9_7 is derived from responses to questions B103 and B108 on the questionnaire. Each write-in response could be coded to a maximum of three codes in the International Classification of Diseases - 9th revision - 1975 (ICD9), for a total of 6 possible ICD9 codes for each respondent. A value=1 for ICD9_7 indicates that this person reported "Other Heart Conditions" as an underlying condition of their disability in question B103 or B108.

Variable Name: **ICD9_8** *Position:* 1408 *Length:* 1
Collection Name: ICD9_8

Derived variable: Underlying condition - Other circulatory disorders

		FREQ	WTD
1	Yes	816	152,763
3	No	19,894	3,267,575
		======	=========
		20,710	3,420,338

Coverage: All respondents (DISAB=1)
Source: Participation and Activity Limitation Survey, 2001
Note: ICD9_8 is derived from responses to questions B103 and B108 on the questionnaire. Each write-in response could be coded to a maximum of three codes in the International Classification of Diseases - 9th revision - 1975 (ICD9), for a total of 6 possible ICD9 codes for each respondent. A value=1 for ICD9_8 indicates that this person reported "Other Circulatory Disorders" as an underlying condition of their disability in question B103 or B108.

Variable Name: **ICD9_9** *Position:* 1409 *Length:* 1
Collection Name: ICD9_9

Derived variable: Underlying condition - Bronchitis and emphysema

		FREQ	WTD
1	Yes	197	37,892
3	No	20,513	3,382,446
		======	=========
		20,710	3,420,338

Coverage: All respondents (DISAB=1)
Source: Participation and Activity Limitation Survey, 2001
Note: ICD9_9 is derived from responses to questions B103 and B108 on the questionnaire. Each write-in response could be coded to a maximum of three codes in the International Classification of Diseases - 9th revision - 1975 (ICD9), for a total of 6 possible ICD9 codes for each respondent. A value=1 for ICD9_9 indicates that this person reported "Bronchitis and Emphysema" as an underlying condition of their disability in question B103 or B108.

Variable Name: **ICD9_10** *Position:* 1410 *Length:* 1
Collection Name: ICD9_10

Derived variable: Underlying condition - Asthma

		FREQ	WTD
1	Yes	729	112,694
3	No	19,981	3,307,643
		======	=========
		20,710	3,420,337

Coverage: All respondents (DISAB=1)
Source: Participation and Activity Limitation Survey, 2001
Note: ICD9_10 is derived from responses to questions B103 and B108 on the questionnaire. Each write-in response could be coded to a maximum of three codes in the International Classification of Diseases - 9th revision - 1975 (ICD9), for a total of 6 possible ICD9 codes for each respondent. A value=1 for ICD9_10 indicates that this person reported "Asthma" as an underlying condition of their disability in question B103 or B108.

Variable Name:	**ICD9_11**	*Position:*	1411	*Length:*	1
Collection Name:	ICD9_11				

Derived variable: Underlying condition - Other respiratory disorders

		FREQ	WTD
1	Yes	403	77,811
3	No	20,307	3,342,527
		======	=========
		20,710	3,420,338

Coverage: All respondents (DISAB=1)
Source: Participation and Activity Limitation Survey, 2001

Note: ICD9_11 is derived from responses to questions B103 and B108 on the questionnaire. Each write-in response could be coded to a maximum of three codes in the International Classification of Diseases - 9th revision - 1975 (ICD9), for a total of 6 possible ICD9 codes for each respondent. A value=1 for ICD9_11 indicates that this person reported "Other Respiratory Disorders" as an underlying condition of their disability in question B103 or B108.

Variable Name:	**ICD9_12**	*Position:*	1412	*Length:*	1
Collection Name:	ICD9_12				

Derived variable: Underlying condition - Disorders of the digestive system

		FREQ	WTD
1	Yes	524	81,722
3	No	20,186	3,338,616
		======	=========
		20,710	3,420,338

Coverage: All respondents (DISAB=1)
Source: Participation and Activity Limitation Survey, 2001

Note: ICD9_12 is derived from responses to questions B103 and B108 on the questionnaire. Each write-in response could be coded to a maximum of three codes in the International Classification of Diseases - 9th revision - 1975 (ICD9), for a total of 6 possible ICD9 codes for each respondent. A value=1 for ICD9_12 indicates that this person reported "Disorders of the Digestive System" as an underlying condition of their disability in question B103 or B108.

Variable Name:	**ICD9_13**	*Position:*	1413	*Length:*	1
Collection Name:	ICD9_13				

Derived variable: Underlying condition - Infectious and parasitic diseases

		FREQ	WTD
1	Yes	206	33,302
3	No	20,504	3,387,036
		======	=========
		20,710	3,420,338

Coverage: All respondents (DISAB=1)
Source: Participation and Activity Limitation Survey, 2001

Note: ICD9_13 is derived from responses to questions B103 and B108 on the questionnaire. Each write-in response could be coded to a maximum of three codes in the International Classification of Diseases - 9th revision - 1975 (ICD9), for a total of 6 possible ICD9 codes for each respondent. A value=1 for ICD9_13 indicates that this person reported "Infectious and Parasitic Diseases" as an underlying condition of their disability in question B103 or B108.

Variable Name:	**ICD9_14**	*Position:*	1414	*Length:*	1
Collection Name:	ICD9_14				

Derived variable: Underlying condition - Arthritis (Lower limbs)

		FREQ	WTD
1	Yes	442	83,654
3	No	20,268	3,336,683
		======	=========
		20,710	3,420,337

Coverage: All respondents (DISAB=1)
Source: Participation and Activity Limitation Survey, 2001
Note: ICD9_14 is derived from responses to questions B103 and B108 on the questionnaire. Each write-in response could be coded to a maximum of three codes in the International Classification of Diseases - 9th revision - 1975 (ICD9), for a total of 6 possible ICD9 codes for each respondent. A value=1 for ICD9_14 indicates that this person reported "Arthritis - Lower Limbs" as an underlying condition of their disability in question B103 or B108.

Variable Name:	**ICD9_15**	*Position:*	1415	*Length:*	1
Collection Name:	ICD9_15				

Derived variable: Underlying condition - Arthritis (Upper limbs)

		FREQ	WTD
1	Yes	115	20,831
3	No	20,595	3,399,506
		======	=========
		20,710	3,420,337

Coverage: All respondents (DISAB=1)
Source: Participation and Activity Limitation Survey, 2001
Note: ICD9_15 is derived from responses to questions B103 and B108 on the questionnaire. Each write-in response could be coded to a maximum of three codes in the International Classification of Diseases - 9th revision - 1975 (ICD9), for a total of 6 possible ICD9 codes for each respondent. A value=1 for ICD9_15 indicates that this person reported "Arthritis - Upper Limbs" as an underlying condition of their disability in question B103 or B108.

Variable Name:	**ICD9_16**	*Position:*	1416	*Length:*	1
Collection Name:	ICD9_16				

Derived variable: Underlying condition - Arthritis (Back and spine)

		FREQ	WTD
1	Yes	261	45,662
3	No	20,449	3,374,676
		======	=========
		20,710	3,420,338

Coverage: All respondents (DISAB=1)
Source: Participation and Activity Limitation Survey, 2001
Note: ICD9_16 is derived from responses to questions B103 and B108 on the questionnaire. Each write-in response could be coded to a maximum of three codes in the International Classification of Diseases - 9th revision - 1975 (ICD9), for a total of 6 possible ICD9 codes for each respondent. A value=1 for ICD9_16 indicates that this person reported "Arthritis - Back and Spine" as an underlying condition of their disability in question B103 or B108.

Variable Name:	**ICD9_17**	*Position:*	1417	*Length:*	1
Collection Name:	ICD9_17				

Derived variable: Underlying condition - Arthritis (Other and unspecified)

		FREQ	WTD
1	Yes	3,746	712,773
3	No	16,964	2,707,564
		======	=========
		20,710	3,420,337

Coverage: All respondents (DISAB=1)
Source: Participation and Activity Limitation Survey, 2001
Note: ICD9_17 is derived from responses to questions B103 and B108 on the questionnaire. Each write-in response could be coded to a maximum of three codes in the International Classification of Diseases - 9th revision - 1975 (ICD9), for a total of 6 possible ICD9 codes for each respondent. A value=1 for ICD9_17 indicates that this person reported "Arthritis - Other and Unspecified" as an underlying condition of their disability in question B103 or B108.

Variable Name:	**ICD9_18**	*Position:*	1418	*Length:*	1
Collection Name:	ICD9_18				

Derived variable: Underlying condition - Other musculoskeletal disorders (Lower limb)

		FREQ	WTD
1	Yes	2,177	368,064
3	No	18,533	3,052,274
		======	=========
		20,710	3,420,338

Coverage: All respondents (DISAB=1)
Source: Participation and Activity Limitation Survey, 2001
Note: ICD9_18 is derived from responses to questions B103 and B108 on the questionnaire. Each write-in response could be coded to a maximum of three codes in the International Classification of Diseases - 9th revision - 1975 (ICD9), for a total of 6 possible ICD9 codes for each respondent. A value=1 for ICD9_18 indicates that this person reported "Other Musculoskeletal Disorders - Lower Limbs" as an underlying condition of their disability in question B103 or B108.

Variable Name:	**ICD9_19**	*Position:*	1419	*Length:*	1
Collection Name:	ICD9_19				

Derived variable: Underlying condition - Other musculoskeletal disorders (Upper limb)

		FREQ	WTD
1	Yes	712	120,970
3	No	19,998	3,299,368
		======	=========
		20,710	3,420,338

Coverage: All respondents (DISAB=1)
Source: Participation and Activity Limitation Survey, 2001
Note: ICD9_19 is derived from responses to questions B103 and B108 on the questionnaire. Each write-in response could be coded to a maximum of three codes in the International Classification of Diseases - 9th revision - 1975 (ICD9), for a total of 6 possible ICD9 codes for each respondent. A value=1 for ICD9_19 indicates that this person reported "Other Musculoskeletal Disorders - Upper Limbs" as an underlying condition of their disability in question B103 or B108.

Variable Name: **ICD9_20** *Position:* 1420 *Length:* 1
Collection Name: ICD9_20

Derived variable: Underlying condition - Other musculoskeletal disorders (Back and spine)

		FREQ	WTD
1	Yes	4,685	782,755
3	No	16,025	2,637,582
		======	=========
		20,710	3,420,337

Coverage: All respondents (DISAB=1)
Source: Participation and Activity Limitation Survey, 2001
Note: ICD9_20 is derived from responses to questions B103 and B108 on the questionnaire. Each write-in response could be coded to a maximum of three codes in the International Classification of Diseases - 9th revision - 1975 (ICD9), for a total of 6 possible ICD9 codes for each respondent. A value=1 for ICD9_20 indicates that this person reported "Other Musculoskeletal Disorders - Back and Spine" as an underlying condition of their disability in question B103 or B108.

Variable Name: **ICD9_21** *Position:* 1421 *Length:* 1
Collection Name: ICD9_21

Derived variable: Underlying condition - Other and unspecified musculoskeletal disorders

		FREQ	WTD
1	Yes	701	122,024
3	No	20,009	3,298,314
		======	=========
		20,710	3,420,338

Coverage: All respondents (DISAB=1)
Source: Participation and Activity Limitation Survey, 2001
Note: ICD9_21 is derived from responses to questions B103 and B108 on the questionnaire. Each write-in response could be coded to a maximum of three codes in the International Classification of Diseases - 9th revision - 1975 (ICD9), for a total of 6 possible ICD9 codes for each respondent. A value=1 for ICD9_21 indicates that this person reported "Other and Unspecified Musculoskeletal Disorders" as an underlying condition of their disability in question B103 or B108.

Variable Name: **ICD9_22** *Position:* 1422 *Length:* 1
Collection Name: ICD9_22

Derived variable: Underlying condition - Neoplasms

		FREQ	WTD
1	Yes	431	79,837
3	No	20,279	3,340,500
		======	=========
		20,710	3,420,337

Coverage: All respondents (DISAB=1)
Source: Participation and Activity Limitation Survey, 2001
Note: ICD9_22 is derived from responses to questions B103 and B108 on the questionnaire. Each write-in response could be coded to a maximum of three codes in the International Classification of Diseases - 9th revision - 1975 (ICD9), for a total of 6 possible ICD9 codes for each respondent. A value=1 for ICD9_22 indicates that this person reported "Neoplasms" as an underlying condition of their disability in question B103 or B108.

Variable Name: **ICD9_23** *Position:* 1423 *Length:* 1
Collection Name: ICD9_23

Derived variable: Underlying condition - Endocrine, nutritional, metabolic and immunity disorders

		FREQ	WTD
1	Yes	1,000	172,628
3	No	19,710	3,247,710
		======	=========
		20,710	3,420,338

Coverage: All respondents (DISAB=1)
Source: Participation and Activity Limitation Survey, 2001

Note: ICD9_23 is derived from responses to questions B103 and B108 on the questionnaire. Each write-in response could be coded to a maximum of three codes in the International Classification of Diseases - 9th revision - 1975 (ICD9), for a total of 6 possible ICD9 codes for each respondent. A value=1 for ICD9_23 indicates that this person reported "Endocrine, Nutritional, Metabolic and Immunity Disorders" as an underlying condition of their disability in question B103 or B108.

Variable Name: **ICD9_24** *Position:* 1424 *Length:* 1
Collection Name: ICD9_24

Derived variable: Underlying condition - Other

		FREQ	WTD
1	Yes	9,483	1,614,144
3	No	11,227	1,806,194
		======	=========
		20,710	3,420,338

Coverage: All respondents (DISAB=1)
Source: Participation and Activity Limitation Survey, 2001

Note: ICD9_24 is derived from responses to questions B103 and B108 on the questionnaire. Each write-in response could be coded to a maximum of three codes in the International Classification of Diseases - 9th revision - 1975 (ICD9), for a total of 6 possible ICD9 codes for each respondent. A value=1 for ICD9_24 indicates that this person reported an "Other" underlying condition of their disability in question B103 or B108.

Variable Name: **LIMDUR** *Position:* 1425 *Length:* 1
Collection Name: LIMDUR

Derived variable: Duration of limitation

		FREQ	WTD
0	Less than one year	707	114,734
1	One to two years	2,320	373,769
2	Three to four years	2,338	401,425
3	Five to nine years	4,155	708,896
4	Ten to nineteen years	5,430	900,203
5	Twenty years or more	4,449	702,663
6	Not stated	1,311	218,648
		======	=========
		20,710	3,420,338

Coverage: All respondents (DISAB=1)
Source: Participation and Activity Limitation Survey, 2001
Note: Duration of limitation (LIMDUR) is a derived variable, and replaces the age reported in Question B102 on the questionnaire. The duration of disability was derived by subtracting the age reported in Question B102 from the age of the individual.

Variable Name: **LOINC** *Position:* 1426 *Length:* 1
Collection Name: LOINC

Derived variable: Low income status

		FREQ	WTD
1	Member of non-low income economic family	15,695	2,613,058
2	Member of low income economic family	4,767	789,312
3	Invalid data	248	17,968
		======	=========
		20,710	3,420,338

Coverage: All respondents
Source: 2001 Census of Population
Note: Measures of low income known as low income cut-offs are calculated on the basis of national family expenditure data, family size and degree of urbanization.

Variable Name: **LSTWK** *Position:* 1427 *Length:* 1
Collection Name: LSTWK

Derived variable: When last worked

		FREQ	WTD
1	Last worked before 2000	0	0
2	Last worked in 2000	0	0
3	Last worked in 2001	0	0
4	Never worked	0	0
5	Not applicable	20,710	3,420,338
		======	=========
		20,710	3,420,338

Coverage: Respondents (DISAB=0) aged 15-64
Source: 2001 Census of Population
Note: This derived variable is available only for non-disabled persons (DISAB=0).

Variable Name: **MARSTHP** *Position:* 1428 *Length:* 1
Collection Name: MARSTHP

Derived variable: Marital status

		FREQ	WTD
1	Divorced	1,330	299,827
2	Married/Common-law	10,618	1,893,766
3	Separated	545	114,230
4	Never married (single)	5,137	525,672
5	Widowed	3,041	580,831
9	Invalid data	39	6,012
		======	=========
		20,710	3,420,338

Coverage: All respondents
Source: 2001 Census of Population

Variable Name: **MOBLIM** *Position:* 1429 *Length:* 2
Collection Name: MOBLIM

Derived variable: Flag to indicate a mobility disability

		FREQ	WTD
1	Yes	13,854	2,451,547
3	No	6,596	929,146
4	Undetermined	173	25,884
98	Not stated	87	13,760
		======	=========
		20,710	3,420,337

Coverage: All respondents (DISAB=1)
Source: Participation and Activity Limitation Survey, 2001
Note: MOBLIM is a derived variable. A value=1 in this field indicates that this person has a mobility-related disability

Variable Name: **MTNDRP** *Position:* 1431 *Length:* 1
Collection Name: MTNDRP

Derived variable: Mother tongue

		FREQ	WTD
1	English	14,778	2,176,603
2	French	3,013	562,352
3	Other	2,880	675,370
9	Invalid data	39	6,012
		======	=========
		20,710	3,420,337

Coverage: All respondents
Source: 2001 Census of Population

Variable Name: **NAICS** *Position:* 1432 *Length:* 2
Collection Name: NAICS

Derived variable: Industry sector code - NAICS

		FREQ	WTD
00	Invalid data	0	0
01	Not applicable	20,710	3,420,338
02	Agriculture, Forestry, Fishing and Hunting	0	0
03	Mining and Oil and Gas Extraction	0	0
04	Utilities	0	0
05	Construction	0	0
06	Manufacturing	0	0
07	Wholesale Trade	0	0
08	Retail Trade	0	0
09	Transportation and Warehousing	0	0
10	Information and Cultural Industries	0	0
11	Finance and Insurance	0	0
12	Real Estate and Rental and Leasing	0	0
13	Professional, Scientific and Technical Services	0	0
14	Management of Companies and Enterprises	0	0
15	Administrative and Support, Waste Management and Remediation Services	0	0
16	Educational Services	0	0
17	Health Care and Social Assistance	0	0
18	Arts, Entertainment and Recreation	0	0
19	Accommodation and Food Services	0	0
20	Other Services (except Public Administration)	0	0
21	Public Administration	0	0
		======	=========
		20,710	3,420,338

Coverage: Respondents (DISAB=0) aged 15-64, who worked since January 1, 2000
Source: 2001 Census of Population
Note: NAICS refers to the general nature of the business carried out in the establishment where the person worked. The 2001 industry data are produced according to the 1997 North American Industry Classification System (NAICS). This variable is available only for non-disabled persons (DISAB=0).

Variable Name: **NAICSPALS** *Position:* 1434 *Length:* 2
Collection Name: NAICSPALS

Derived variable: Industry sector - NAICS

		FREQ	WTD
01	Not applicable	6,433	1,053,347
02	Agriculture, Forestry, Fishing and Hunting	217	29,829
03	Mining and Oil and Gas Extraction	85	8,450
04	Utilities	39	8,278
05	Construction	347	44,107
06	Manufacturing	629	108,527
07	Wholesale Trade	137	22,040
08	Retail Trade	731	75,967
09	Transportation and Warehousing	358	58,713
10	Information and Cultural Industries	134	14,547
11	Finance and Insurance	192	30,931
12	Real Estate and Rental and Leasing	75	14,918
13	Professional, Scientific and Technical Services	312	52,183
14	Management of Companies and Enterprises	0	0
15	Administrative and Support, Waste Management and Remediation Services	351	42,949
16	Educational Services	363	61,288
17	Health Care and Social Assistance	782	113,402
18	Arts, Entertainment and Recreation	143	23,042
19	Accommodation and Food Services	468	53,604
20	Other Services (except Public Administration)	331	42,340
21	Public Administration	362	48,909
98	Not stated	8,221	1,512,968
		======	=========
		20,710	3,420,339

Coverage: Respondents (DISAB=1) aged 15-64, who worked since January 1, 1996
Source: Participation and Activity Limitation Survey, 2001
Note: NAICS refers to the general nature of the business carried out in the establishment where the person worked. The 2001 industry data are produced according to the 1997 North American Industry Classification System (NAICS). This variable is available only for disabled persons (DISAB=1).

Variable Name: **NEEDAID** *Position:* 1436 *Length:* 2
Collection Name: NEEDAID

Derived variable: Need but do not have assistive aids and devices

		FREQ	WTD
1	Yes	4,006	657,152
3	No	13,520	2,218,770
4	Undetermined	1,363	228,733
98	Not stated	1,821	315,683
		======	=========
		20,710	3,420,338

Coverage: All respondents (DISAB=1)
Source: Participation and Activity Limitation Survey, 2001
Note: NEEDAID is derived from variables B11, B27, B38, B53, B71, B83 and B129. A value=1 in this field indicates that the person needs, but does not have, assistive aids and devices to enable them to carry out everyday activities. These include, for example, aids that would make it easier for them to get around, or would help them hear, see, speak or learn.

Variable Name: **NEEDHELP** *Position:* 1438 *Length:* 2
Collection Name: NEEDHELP

Derived variable: Need but do not receive help with everyday activities

		FREQ	WTD
1	Yes	4,565	783,205
3	No	13,935	2,228,058
4	Undetermined	557	117,356
98	Not stated	1,653	291,718
		======	=========
		20,710	3,420,337

Coverage: All respondents (DISAB=1)
Source: Participation and Activity Limitation Survey, 2001
Note: NEEDHELP is derived from variables C2, C4, C6, C8, C10, C12, C14, C16, C18, C20, C23, C25, C27, C29, C31, C33, C35 and C37. A value=1 in this field indicates that the person needs, but does not receive, help or additional help with everyday activities because of their condition. Such activities include meal preparation, everyday housework, household chores, getting to appointments and running errands, looking after personal finances, child care, personal or specialized nursing care, and moving around within the home.

Variable Name:	**NEEDOTH**	*Position:*	1440	*Length:*	2
Collection Name:	NEEDOTH				

Derived variable: Need but do not have other assistive aids and devices

		FREQ	WTD
1	Yes	1,334	186,384
3	No	18,055	3,022,281
93	Not applicable	0	0
98	Not stated	893	146,178
R	Refusal	1	106
X	Don't know	427	65,389
		======	=========
		20,710	3,420,338

Coverage: All respondents (DISAB=1)
Source: Participation and Activity Limitation Survey, 2001
Note: NEEDOTH is derived from responses to questions B83 and B129.

Variable Name:	**NOC**	*Position:*	1442	*Length:*	2
Collection Name:	NOC				

Derived variable: Occupation major group - NOC-S

		FREQ	WTD
00	Invalid data	0	0
01	Not applicable	20,710	3,420,338
02	Management Occupations	0	0
03	Business, Finance and Administrative Occupations	0	0
04	Natural and Applied Sciences and Related Occupations	0	0
05	Health Occupations	0	0
06	Occupations in Social Science, Education, Government Service and Religion	0	0
07	Occupations in Art, Culture, Recreation and Sport	0	0
08	Sales and Service Occupations	0	0
09	Trades, Transport and Equipment Operators and Related Occupations	0	0
10	Occupations Unique to Primary Industry	0	0
11	Occupations Unique to Processing, Manufacturing and Utilities	0	0
		======	=========
		20,710	3,420,338

Coverage: Respondents (DISAB=0) aged 15-64, who worked since January 1, 2000
Source: 2001 Census of Population
Note: NOC refers to the kind of work a person was doing during the reference week, as determined by their kind of work and the description of the main activities in their job. The 2001 occupation data are classified according to the 2001 National Occupational Classification for Statistics (NOC-S 2001). This variable is available only for non-disabled persons (DISAB=0).

Variable Name:	**NOCPALS**	*Position:*	1444	*Length:*	2
Collection Name:	NOCPALS				

Derived variable: Occupation major group - NOC-S

		FREQ	WTD
01	Not applicable	6,433	1,053,347
02	Management Occupations	325	47,194
03	Business, Finance and Administrative Occupations	1,108	150,458
04	Natural and Applied Sciences and Related Occupations	287	45,006
05	Health Occupations	311	50,097
06	Occupations in Social Science, Education, Government Service and Religion	479	81,997
07	Occupations in Art, Culture, Recreation and Sport	190	28,541
08	Sales and Service Occupations	1,787	210,055
09	Trades, Transport and Equipment Operators and Related Occupations	953	146,312
10	Occupations Unique to Primary Industry	257	34,425
11	Occupations Unique to Processing, Manufacturing and Utilities	384	62,350
98	Not stated	8,196	1,510,554
		======	=========
		20,710	3,420,336

Coverage: Respondents (DISAB=1) aged 15-64, who worked since January 1, 1996
Source: Participation and Activity Limitation Survey, 2001

Note: NOC refers to the kind of work a person was doing during the reference week, as determined by their kind of work and the description of the main activities in their job. The 2001 occupation data are classified according to the 2001 National Occupational Classification for Statistics (NOC-S 2001). This variable is available only for disabled persons (DISAB=1).

Variable Name:	**NSTIENP**	*Position:*	1446	*Length:*	2
Collection Name:	NSTIENP				

Derived variable: Number of maintainer(s) in household

		FREQ	WTD
1	1 maintainer	13,246	2,275,557
2	2 maintainers	6,739	1,045,698
3	3 maintainers	353	59,349
4	4 or more maintainers	130	21,915
91	Invalid data	242	17,819
		======	=========
		20,710	3,420,338

Coverage: All respondents
Source: 2001 Census of Population

Variable Name:	**NUM_COND**	*Position:*	1448	*Length:*	2
Collection Name:	NUM_COND				

Derived variable: Number of underlying conditions reported

		FREQ	WTD
0	No underlying condition reported	1,460	241,523
1	One underlying condition reported	10,398	1,631,534
2	Two underlying conditions reported	2,249	347,031
3	Three underlying conditions reported	6,004	1,088,367
4	Four underlying conditions reported	557	104,491
5	Five underlying conditions reported	41	7,271
6	Six underlying conditions reported	1	120
		======	=========
		20,710	3,420,337

Coverage: All respondents (DISAB=1)
Source: Participation and Activity Limitation Survey, 2001

Note: This variable is derived from responses to questions B103 (main underlying condition) and B108 (secondary underlying condition). Each of these write-in responses could be coded to a maximum of three codes in the International Classification of Diseases - 9th revision - 1975 (ICD9), for a total of six possible conditions being reported for each respondent. NUM_COND represents the total number of underlying conditions reported by each respondent in the write-in responses of B103 and B108.

Variable Name:	**OTH_LIM**	*Position:*	1450	*Length:*	2
Collection Name:	OTH_LIM				

Derived variable: Flag to indicate other limitations

		FREQ	WTD
1	Yes	6,941	1,045,137
3	No	13,167	2,258,251
4	Undetermined	280	56,070
98	Not stated	322	60,880
		======	=========
		20,710	3,420,338

Coverage: All respondents (DISAB=1)
Source: Participation and Activity Limitation Survey, 2001

Note: OTH_LIM is a derived variable. A value=1 in this field indicates that this person has an "other" disability. Included in this category are learning difficulties, developmental disabilities, activity limitations related to an emotional, psychological or psychiatric condition, limitations related to memory problems, and unknown types of disabilities.

Variable Name: **PAINLIM** *Position:* 1452 *Length:* 2
Collection Name: PAINLIM

Derived variable: Flag to indicate a pain-related disability

		FREQ	WTD
1	Yes	13,878	2,376,915
3	No	6,586	1,013,691
4	Undetermined	113	12,423
98	Not stated	133	17,309
		======	=========
		20,710	3,420,338

Coverage: All respondents (DISAB=1)
Source: Participation and Activity Limitation Survey, 2001
Note: PAINLIM is a derived variable. A value=1 in this field indicates that this person has activity limitations due to constant or recurring long-term pain.

Variable Name: **RECHELP** *Position:* 1454 *Length:* 2
Collection Name: RECHELP

Derived variable: Receives help with everyday activities

		FREQ	WTD
1	Yes	13,393	2,268,076
3	No	6,533	1,016,675
4	Undetermined	63	12,439
98	Not stated	721	123,148
		======	=========
		20,710	3,420,338

Coverage: All respondents (DISAB=1)
Source: Participation and Activity Limitation Survey, 2001
Note: RECHELP is derived from variables C1, C5, C9, C13, C17, C22, C26, C30 and C34. A value=1 in this field indicates that the person receives help with everyday activities because of their condition. Such activities include meal preparation, everyday housework, household chores, getting to appointments and running errands, looking after personal finances, child care, personal or specialized nursing care, and moving around within the home.

Variable Name:	**ROOMSP**	*Position:* 1456	*Length:* 2
Collection Name:	ROOMSP		

Derived variable: Number of rooms

		FREQ	WTD
1	1-3 Rooms	1,746	353,310
2	4 Rooms	2,638	442,300
3	5 Rooms	3,737	620,807
4	6 Rooms	3,509	566,701
5	7 Rooms	2,803	493,128
6	8 Rooms	2,413	404,682
7	9 Rooms	1,452	217,738
8	10 Rooms	1,133	166,859
9	11 Rooms or more	1,037	136,993
91	Invalid data	242	17,819
		======	=========
		20,710	3,420,337

Coverage: All respondents
Source: 2001 Census of Population

Variable Name:	**RPAIR**	*Position:* 1458	*Length:* 1
Collection Name:	RPAIR		

Dwelling in need of repairs

		FREQ	WTD
1	No, only regular maintenance	11,649	2,065,209
2	Invalid data	242	17,819
3	Yes, major repairs are needed	2,477	356,467
4	Yes, minor repairs are needed	6,342	980,842
		======	=========
		20,710	3,420,337

Coverage: All respondents
Source: 2001 Census of Population

Variable Name: **SEELIM** *Position:* 1459 *Length:* 2
Collection Name: SEELIM

Derived variable: Flag to indicate a seeing disability

		FREQ	WTD
1	Yes	3,561	594,346
3	No	16,775	2,765,324
4	Undetermined	183	27,341
98	Not stated	191	33,327
		======	=========
		20,710	3,420,338

Coverage: All respondents (DISAB=1)
Source: Participation and Activity Limitation Survey, 2001
Note: SEELIM is a derived variable. A value=1 in this field indicates that this person has a seeing disability.

Variable Name: **SPCHLIM** *Position:* 1461 *Length:* 2
Collection Name: SPCHLIM

Derived variable: Flag to indicate a speech disability

		FREQ	WTD
1	Yes	2,542	362,597
3	No	17,926	3,011,944
4	Undetermined	20	7,062
98	Not stated	222	38,734
		======	=========
		20,710	3,420,337

Coverage: All respondents (DISAB=1)
Source: Participation and Activity Limitation Survey, 2001
Note: SPCHLIM is a derived variable. A value =1 in this field indicates that this person has a speech disability

---

Variable Name: **SRC_HELP1** *Position:* 1463 *Length:* 2
Collection Name: SRC_HELP1

Derived variable: Source of help received - family living with the person

		FREQ	WTD
0	No	12,041	1,994,875
1	Yes	8,669	1,425,463
		======	=========
		20,710	3,420,338

Coverage: Respondents (DISAB = 1) who reported receiving help with preparing meals, everyday housework, heavy chores, appointments/errands, looking after personal finances, child care, personal care or moving about inside residence.
Source: Participation and Activity Limitation Survey, 2001
Note: SRC_HELP1 is derived from variables C3A, C7A, C11A, C15A, C19A, C24A, C28A, C36A.

Variable Name:	**SRC_HELP2**	*Position:*	1465	*Length:*	2
Collection Name:	SRC_HELP2				

Derived variable: Source of help received - family not living with the person

		FREQ	WTD
0	No	15,092	2,464,211
1	Yes	5,618	956,127
		======	=========
		20,710	3,420,338

Coverage: Respondents (DISAB = 1) who reported receiving help with preparing meals, everyday housework, heavy chores, appointments/errands, looking after personal finances, child care, personal care or moving about inside residence.
Source: Participation and Activity Limitation Survey, 2001
Note: SRC_HELP2 is derived from variables C3B, C7B, C11B, C15B, C19B, C24B, C28B, C36B.

Variable Name:	**SRC_HELP3**	*Position:*	1467	*Length:*	2
Collection Name:	SRC_HELP3				

Derived variable: Source of help received - friends or neighbours

		FREQ	WTD
0	No	17,319	2,872,445
1	Yes	3,391	547,893
		======	=========
		20,710	3,420,338

Coverage: Respondents (DISAB = 1) who reported receiving help with preparing meals, everyday housework, heavy chores, appointments/errands, looking after personal finances, child care, personal care or moving about inside residence.
Source: Participation and Activity Limitation Survey, 2001
Note: SRC_HELP3 is derived from variables C3C, C7C, C11C, C15C, C19C, C24C, C28C, C36C.

Variable Name:	**SRC_HELP4**	*Position:*	1469	*Length:*	2
Collection Name:	SRC_HELP4				

Derived variable: Source of help received - Organizations or Agencies

		FREQ	WTD
0	No	17,888	2,925,469
1	Yes	2,822	494,869
		======	=========
		20,710	3,420,338

Coverage: Respondents (DISAB = 1) who reported receiving help with preparing meals, everyday housework, heavy chores, appointments/errands, looking after personal finances, child care, personal care or moving about inside residence.
Source: Participation and Activity Limitation Survey, 2001
Note: SRC_HELP4 is derived from variables C3D, C7D, C11D, C15D, C19D, C24D, C28D, C36D.

Variable Name:	**SRC_HELP5**	*Position:*	1471	*Length:*	2
Collection Name:	SRC_HELP5				

Derived variable: Source of help received - other

		FREQ	WTD
0	No	18,893	3,104,190
1	Yes	1,817	316,148
		======	=========
		20,710	3,420,338

Coverage: Respondents (DISAB = 1) who reported receiving help with preparing meals, everyday housework, heavy chores, appointments/errands, looking after personal finances, child care, personal care or moving about inside residence.
Source: Participation and Activity Limitation Survey, 2001
Note: SRC_HELP5 is derived from variables C3E, C7E, C11E, C15E, C19E, C24E, C28E, C36E.

Variable Name:	**TENURP**	*Position:*	1473	*Length:*	1
Collection Name:	TENURP				

Tenure

		FREQ	WTD
1	Invalid data	252	18,255
3	Owned by member of household	14,413	2,324,516
4	Rented	6,045	1,077,567
		======	=========
		20,710	3,420,338

Coverage: All respondents
Source: 2001 Census of Population

Variable Name:	**TOTINCR**	*Position:*	1474	*Length:*	2
Collection Name:	TOTINCR				

Derived variable: Total income

		FREQ	WTD
00	No income	1,024	103,672
01	Less than $5,000	2,685	322,204
02	$5,000 - $9,999	3,050	473,130
03	$10,000 - $14,999	4,154	738,047
04	$15,000 - $19,999	2,752	467,565
05	$20,000 - $29,999	2,931	529,165
06	$30,000 - $39,999	1,745	321,425
07	$40,000 - $49,999	1,012	178,384
08	$50,000 - $59,999	536	113,575
09	$60,000 - $79,999	465	101,268
10	$80,000 or more	296	64,600
11	Invalid data	60	7,303
		======	=========
		20,710	3,420,338

Coverage: All respondents
Source: 2001 Census of Population

Note: This is a derived variable. It refers to the total money income received by individuals 15 years of age and over during the calendar year 2000 from the following sources: wages and salaries, self-employment income, Canada Child Tax Benefits, Old Age Security pension and Guaranteed Income Supplement, benefits from Canada/Quebec Pension Plans, benefits from Employment Insurance, other income from government sources, dividends, interest and other investment income, retirement pensions, superannuation and annuities, and other money income.

Variable Name:	**TRMODEP**	*Position:*	1476	*Length:*	1
Collection Name:	TRMODEP				

Derived variable: Mode of transportation to work

		FREQ	WTD
1	Bicycle, motorcycle, taxicab or other mode of transportation	248	27,090
2	Car, truck or van - driver	4,534	710,450
3	Invalid data	60	7,303
4	Not applicable	13,835	2,432,691
6	Car, truck or van - passenger	760	71,109
8	Public transit	679	99,286
9	Walked	594	72,408
		======	=========
		20,710	3,420,337

Coverage: All respondents aged 15-64, who worked at some time since January 1, 2000 at a usual workplace address, or had no fixed workplace address.
Source: 2001 Census of Population

Note: TRMODEP usually relates to the individual's job in the week prior to Census enumeration. However, if a person did not work during that week but had worked at some time since January 1, 2000, the information relates to the job held longest during that period.

Variable Name:	**UNITSP**	*Position:*	1477	*Length:*	1
Collection Name:	UNITSP				

Derived variable: Number of persons in household

		FREQ	WTD
1	One person	4,161	784,871
2	Two persons	7,775	1,412,697
3	Three persons	3,643	544,654
4	Four persons	2,858	376,562
5	Five persons or more	2,234	295,543
9	Invalid data	39	6,012
		======	=========
		20,710	3,420,339

Coverage: All respondents
Source: 2001 Census of Population

Variable Name:	**UPHWKP**	*Position:*	1478	*Length:*	1
Collection Name:	UPHWKP				

Unpaid housework

		FREQ	WTD
1	No hours of housework	3,382	529,341
2	Less than 5 hours of housework	4,450	664,018
3	5 to 14 hours of housework	5,627	947,955
4	15 to 29 hours of housework	3,807	681,901
5	30 to 59 hours of housework	2,439	440,774
6	60 or more hours of housework	945	149,046
9	Invalid data	60	7,303
		======	=========
		20,710	3,420,338

Coverage: All respondents 15 years of age and over
Source: 2001 Census of Population
Note: UPHWKP refers to the number of hours persons spent doing unpaid housework, yard work or home maintenance in the week (Sunday to Saturday) prior to Census Day. It includes hours spent doing unpaid housework for members of one's own household, for other family members outside the household, and for friends or neighbours. It does not include volunteer work for a non-profit organization, a religious organization, a charity or community group, or work without pay in the operation of a family farm, business or professional practice.

Variable Name:	**UPKIDP**	*Position:*	1479	*Length:*	1
Collection Name:	UPKIDP				

Unpaid child care

		FREQ	WTD
1	No hours of child care	15,100	2,546,988
2	Less than 5 hours of child care	1,666	289,792
3	5 to 14 hours of child care	1,323	214,900
4	15 to 29 hours of child care	850	140,246
5	30 to 59 hours of child care	670	94,399
6	60 or more hours of child care	1,041	126,710
9	Invalid data	60	7,303
		======	=========
		20,710	3,420,338

Coverage: All respondents 15 years of age and over
Source: 2001 Census of Population

Note: UPKIDP refers to the number of hours persons spent looking after children without pay. It includes hours spent providing child care for members of one's own household, for other family members outside the household, for friends or neighbours or for other family members outside the household in the week (Sunday to Saturday) prior to Census Day.

Variable Name:	**USEAID**	*Position:*	1480	*Length:*	2
Collection Name:	USEAID				

Derived variable: Use assistive aids and devices

		FREQ	WTD
1	Yes	9,779	1,683,725
3	No	9,229	1,446,282
4	Undetermined	545	88,683
93	Not applicable	0	0
98	Not stated	1,157	201,649
		======	=========
		20,710	3,420,339

Coverage: All respondents (DISAB=1)
Source: Participation and Activity Limitation Survey, 2001

Note: USEAID is derived from variables B9, B25, B36, B51, B69, B81 and B124. A value=1 in this field indicates that the person uses assistive aids and devices to enable them to carry out everyday activities. These include, for example, aids that make it easier for them to get around, or that help them hear, see, speak or learn.

Variable Name:	**USEOTH**	*Position:*	1482	*Length:*	2
Collection Name:	USEOTH				

Derived variable: Use other assistive aids and devices

		FREQ	WTD
1	Yes	2,817	470,771
3	No	17,087	2,815,032
93	Not applicable	0	0
98	Not stated	774	126,788
R	Refusal	1	106
X	Don't know	31	7,641
		======	=========
		20,710	3,420,338

Coverage: All respondents (DISAB=1)
Source: Participation and Activity Limitation Survey, 2001
Note: USEOTH is derived from variables B81 and B124.

Variable Name:	**WORKACTP**	*Position:*	1484	*Length:*	2
Collection Name:	WORKACTP				

Derived variable: Work activity

		FREQ	WTD
01	Not applicable	7,796	1,451,844
02	Did not work in 2000, worked before 2000/never	4,756	796,495
03	Did not work in 2000, only in 2001	221	25,618
04	Worked 1-13 weeks mostly full time	326	37,518
05	Worked 1-13 weeks mostly part time	439	42,135
06	Worked 14-26 weeks mostly full time	515	57,671
07	Worked 14-26 weeks mostly part time	386	42,278
08	Worked 27-39 weeks mostly full time	391	56,922
09	Worked 27-39 weeks mostly part time	236	33,527
10	Worked 40-48 weeks mostly full time	640	99,661
11	Worked 40-48 weeks mostly part time	258	40,345
12	Worked 49-52 weeks mostly full time	2,838	471,005
13	Worked 49-52 weeks mostly part time	640	97,033
91	Invalid data	1,268	168,284
		======	=========
		20,710	3,420,336

Coverage: All respondents aged 15-64
Source: 2001 Census of Population
Note: WORKACTP refers to the number of weeks in which a person worked for pay or in self-employment in 2000 at all jobs held, even if only for a few hours, and whether these weeks were mostly full time (30 hours or more per week) or mostly part time (1 to 29 hours per week). The term "full-year full-time workers" refers to persons aged 15-64 who worked 49-52 weeks (mostly full time) in 2000 for pay or in self-employment.

INDEX

A
AGEGRP5 2
AGILIM 302
ATTENDRP 302
B
B1 3
B10_OTH 7
B100 36
B101 37
B104 37
B105 38
B107 38
B109 39
B10A 4
B10B 4
B10C 4
B10D 5
B10E 5
B10F 5
B10G 6
B10H 6
B10I 6
B11 7
B110 39
B112 40
B113_R 40
B114_R 41
B115 41
B116_R 42
B117_R 42
B118 43
B12 8
B12_OTH 11
B121 43
B122 44
B123 44
B126 45
B12A 8
B12B 8
B12C 9
B12D 9
B12E 9
B12F 10
B12G 10
B12H 10
B12I 11
B12L 11
B131A 45
B131B 46
B131C 46
B131D 47
B131E 47
B131F 48
B14 12
B19 12
B25 13
B26_OTH 16
B26A 13
B26B 14
B26C 14
B26D 14
B26E 15
B26F 15
B26G 15
B26H 16
B27 17
B28 17
B28_OTH 20
B28A 17
B28B 18
B28C 18
B28D 18
B28E 19
B28F 19
B28G 19
B28H 20
B28I 20
B36 21
B38 21
B51 22
B52A 22
B52B 23
B52C 23
B52DE 26

B52F.. 23
B52G.. 24
B52H.. 24
B52I.. 24
B52J 25
B52K.. 25
B53.. 26
B54.. 27
B54A.. 27
B54B 27
B54C 28
B54DE.. 30
B54F.. 28
B54G.. 28
B54H.. 29
B54I.. 29
B54J 29
B54K.. 30
B69.. 30
B70A.. 31
B70B 31
B70C 31
B71.. 32
B72.. 32
B72A.. 32
B72B 33
B72C 33
B9.. 3
B94.. 33
B95.. 34
B96.. 34
B97.. 35
B98.. 35
B99.. 36
BUILT.. 303

C

C1.. 48
C10.. 62
C11A.. 62
C11AA.. 63
C11AB 64
C11AC 65
C11AD.. 66
C11AE.. 67
C11B 63
C11C 64
C11D.. 65
C11E 66
C12.. 67
C13.. 68
C14.. 68
C15A.. 69
C15AA.. 69
C15AB 70
C15AC 71
C15AD.. 72
C15AE.. 73
C15B 70
C15C 71
C15D.. 72
C15E 73
C16.. 74
C17.. 74
C18.. 75
C19A.. 75
C19AA.. 76
C19AB 77
C19AC 78
C19AD.. 79
C19AE.. 80
C19B 76
C19C 77
C19D.. 78
C19E 79
C2.. 49
C20.. 80
C22.. 81
C23.. 81
C24A.. 82
C24AA.. 82
C24AB 83
C24AC 84
C24AD.. 85
C24AE.. 86
C24B 83
C24C 84
C24D.. 85
C24E 86
C25.. 87
C26.. 87
C27.. 88
C28A.. 88

C28AA 88
C28AB 89
C28AC 90
C28AD 90
C28AE 91
C28B 89
C28C 89
C28D 90
C28E 91
C29 91
C30 92
C31 92
C32A 93
C32AA 93
C32AB 94
C32AC 95
C32AD 96
C32AE 97
C32B 94
C32C 95
C32D 96
C32E 97
C33 98
C34 98
C35 99
C36A 99
C36B 100
C36C 100
C36D 101
C36E 101
C37 102
C38 102
C39A 103
C39B 103
C39C 104
C39D 104
C39E 105
C3A 49
C3AA 50
C3AB 51
C3AC 52
C3AD 53
C3AE 54
C3B 50
C3C 51
C3D 52
C3E 53
C4 54
C40 105
C40A 105
C40B 106
C40C 106
C40D 106
C40E 107
C40F 107
C40G 107
C40H 108
C41 108
C42A 108
C42B 109
C42C 109
C42D 110
C45A 110
C45B 111
C45C 111
C45D 112
C45E 112
C45F 113
C45G 113
C45H 114
C46 114
C47A 115
C47B 115
C47C 116
C47D 116
C47E 117
C47F 117
C47G 118
C48 118
C5 55
C51 119
C52A 119
C52B 120
C52C 120
C52D 121
C52E 121
C52F 122
C6 55
C7A 56
C7AA 56
C7AB 57
C7AC 58

C7AD ... 59
C7AE ... 60
C7B ... 57
C7C ... 58
C7D ... 59
C7E ... 60
C8 ... 61
C9 ... 61
CFAMSTR ... 303
CFINCR ... 304
CHDNUMBER ... 304
COM_LAW ... 305
COSTAIDS ... 305
COSTHELP ... 306
COSTHLTH ... 306
COSTMED ... 307
COSTRMOD ... 307
COSTTRAN ... 308
COWD ... 308
D
D1 ... 122
D10A ... 127
D10AA ... 127
D10AB ... 128
D10AC ... 128
D10AD ... 129
D10AE ... 130
D10AF ... 130
D10B ... 127
D10C ... 128
D10D ... 129
D10E ... 129
D10F ... 130
D11 ... 131
D12A ... 131
D12AA ... 131
D12AB ... 132
D12AC ... 133
D12AD ... 133
D12AE ... 134
D12AF ... 135
D12AG ... 135
D12AH ... 136
D12AI ... 137
D12AJ ... 137
D12B ... 132
D12C ... 132
D12D ... 133
D12E ... 134
D12F ... 134
D12G ... 135
D12H ... 136
D12I ... 136
D12J ... 137
D13 ... 138
D14 ... 138
D15A ... 139
D15AA ... 139
D15AB ... 140
D15AC ... 140
D15AD ... 141
D15AE ... 142
D15AF ... 142
D15B ... 139
D15C ... 140
D15D ... 141
D15E ... 141
D15F ... 142
D16 ... 143
D17A ... 143
D17AA ... 143
D17AB ... 144
D17AC ... 145
D17AD ... 145
D17AE ... 146
D17AF ... 147
D17AG ... 147
D17AH ... 148
D17AI ... 149
D17AJ ... 149
D17B ... 144
D17C ... 144
D17D ... 145
D17E ... 146
D17F ... 146
D17G ... 147
D17H ... 148
D17I ... 148
D17J ... 149
D18 ... 150
D19 ... 150
D2 ... 123

D21A.......... 151
D21B.......... 151
D21C.......... 152
D21D.......... 152
D21E.......... 153
D21F.......... 153
D21G.......... 154
D3.......... 123
D4.......... 123
D5.......... 124
D6.......... 124
D8.......... 125
D8A.......... 125
D8B.......... 125
D8C.......... 126
D8I.......... 126
D9.......... 126
DEG_AGILP.......... 309
DEG_HEARP.......... 309
DEG_MOBP.......... 310
DEG_OTHEP.......... 310
DEG_PAINP.......... 311
DEG_SEEP.......... 311
DEG_SPCHP.......... 312
DEGREE.......... 312
DGMFSP.......... 313
DISAB.......... 1
DTYPE.......... 313
E
E100_108.......... 222
E13.......... 157
E14.......... 157
E15.......... 158
E16.......... 158
E17.......... 159
E18.......... 159
E1HRS.......... 155
E24.......... 160
E25.......... 160
E26.......... 161
E27.......... 161
E28.......... 162
E29A.......... 162
E29B.......... 163
E29C.......... 163
E30.......... 164
E31.......... 164
E32.......... 165
E33.......... 165
E34.......... 166
E35.......... 166
E36.......... 167
E37.......... 167
E38.......... 168
E39A.......... 168
E39AA.......... 169
E39AB.......... 170
E39AC.......... 171
E39AD.......... 172
E39AE.......... 173
E39AF.......... 174
E39AG.......... 175
E39B.......... 169
E39C.......... 170
E39D.......... 171
E39E.......... 172
E39F.......... 173
E39G.......... 174
E40A.......... 175
E40AA.......... 176
E40AB.......... 177
E40AC.......... 178
E40AD.......... 179
E40AE.......... 180
E40AF.......... 181
E40AG.......... 182
E40B.......... 176
E40C.......... 177
E40D.......... 178
E40E.......... 179
E40F.......... 180
E40G.......... 181
E41.......... 182
E42.......... 183
E43.......... 183
E44.......... 184
E45A.......... 184
E45B.......... 185
E45C.......... 185
E45D.......... 186
E45E.......... 186
E45F.......... 187

E45G ... 187
E46A ... 188
E46B ... 188
E46C ... 189
E46D ... 189
E47 ... 190
E48 ... 190
E49_LSTWK ... 191
E50HRS ... 191
E54 ... 192
E55 ... 192
E56 ... 193
E57 ... 193
E58 ... 194
E59 ... 194
E60 ... 195
E61 ... 195
E62A ... 196
E62B ... 196
E63A ... 197
E63B ... 197
E63C ... 198
E63D ... 198
E63E ... 199
E63F ... 199
E63G ... 200
E64A ... 200
E64B ... 201
E64C ... 201
E64D ... 202
E64E ... 202
E64F ... 203
E64G ... 203
E65 ... 204
E66 ... 204
E67 ... 205
E68 ... 205
E69A ... 206
E69B ... 206
E69C ... 207
E69D ... 207
E69E ... 208
E69F ... 208
E69G ... 208
E70A ... 209
E70B ... 209
E70C ... 210
E70D ... 210
E71 ... 211
E72 ... 211
E73_LSTWK ... 212
E7HRS ... 155
E8 ... 156
E82_102 ... 212
E91_104A ... 213
E91_104B ... 213
E91_104C ... 214
E91_104D ... 214
E91_104E ... 215
E91_104F ... 215
E91_104G ... 216
E92_105A ... 216
E92_105B ... 217
E92_105C ... 217
E92_105D ... 218
E92_105E ... 218
E92_105F ... 219
E92_105G ... 219
E98_106A ... 220
E98_106B ... 220
E98_106C ... 221
E98_106D ... 221
E99_107 ... 222
E9JSTART ... 156
EFAMSTR ... 314
EMPINR ... 314

F

F1 ... 223
F10 ... 237
F11 ... 238
F12A ... 238
F12B ... 239
F12C ... 239
F12D ... 240
F12E ... 240
F12F ... 241
F12G ... 241
F12H ... 242
F13 ... 242
F14 ... 243
F15A ... 243
F15B ... 244

F15C 244
F15D 245
F16 245
F17 246
F18A 246
F18B 247
F18C 247
F18D 247
F19 248
F2 223
F20 248
F21 249
F22A 249
F22B 249
F22C 250
F23 250
F24 251
F25 251
F26 252
F27A 252
F27B 253
F27C 253
F27D 254
F27E 254
F27F 255
F28 255
F29A 256
F29B 256
F29C 257
F29D 257
F29E 257
F29F 258
F3 224
F30 258
F31 259
F32 259
F33 260
F34A 260
F34B 261
F34C 261
F34D 262
F34E 262
F34F 262
F34G 263
F34H 263
F34I 263
F34J 264
F34K 264
F34L 265
F35A 265
F35B 266
F35C 266
F35D 267
F36 267
F37A 268
F37B 268
F37C 269
F37D 269
F37E 270
F37F 270
F37G 271
F37H 271
F37I 272
F37J 272
F37K 273
F37L 273
F38 274
F39 274
F40 275
F41 275
F44 276
F45_OTH 279
F45B 276
F45C 277
F45D 277
F45E 277
F45F 278
F45G 278
F46 279
F47 280
F48A 280
F48B 281
F48C 281
F48D 282
F48E 282
F48F 283
F48G 283
F48H 284
F49A 284
F49B 284
F49C 285
F49D 285

F49E.. 286
F4A 224
F4B.. 225
F4C.. 225
F4D 226
F4E.. 226
F50 286
F51 287
F52 287
F53 288
F5A 227
F5B.. 227
F5C.. 228
F6A 228
F6B.. 229
F6C.. 229
F6D 230
F6E.. 230
F6F 231
F6G 231
F6H 232
F7 232
F8A 233
F8B.. 233
F8C.. 234
F8D 234
F8E.. 235
F8F 235
F8G 236
F8H 236
F9 237
G
G1A.. 288
G1B.. 289
G1C.. 289
G2.. 290
G3.. 290
G4.. 291
G5.. 291
G6A.. 292
G6B.. 292
G6C.. 293
G6D.. 293
G7A.. 294
G7B.. 294
G7C.. 295
G7D.. 295
G7E 296
G8C.. 296
G8D.. 297
G8E 297
G8F 298
G8G.. 298
G8H.. 299
G8I 299
G8J 300
G8K.. 300
G8L 301
G8M.. 301
H
HEARLIM 315
HHINCR 315
HLNBP 316
HLSA 316
HMAIN... 316
HOURS... 317
I
ICD9_1... 317
ICD9_10... 320
ICD9_11... 321
ICD9_12... 321
ICD9_13... 321
ICD9_14... 322
ICD9_15... 322
ICD9_16... 322
ICD9_17... 323
ICD9_18... 323
ICD9_19... 323
ICD9_2... 318
ICD9_20... 324
ICD9_21... 324
ICD9_22... 324
ICD9_23... 325
ICD9_24... 325
ICD9_3... 318
ICD9_4... 318
ICD9_5... 319
ICD9_6... 319
ICD9_7... 319
ICD9_8... 320
ICD9_9... 320
IDNUM 1

L
LFSTAT ... 154
LIMDUR ... 326
LOINC ... 326
LSTWK ... 327
M
MARSTHP ... 327
MOBLIM ... 327
MTNDRP ... 328
N
NAICS ... 328
NAICSPALS ... 329
NEEDAID ... 330
NEEDHELP ... 330
NEEDOTH ... 331
NOC ... 331
NOCPALS ... 332
NSTIENP ... 332
NUM_COND ... 333
O
OTH_LIM ... 333
P
PAINLIM ... 334
R
RECHELP ... 334
ROOMSP ... 335
RPAIR ... 335
S
SEELIM ... 336
SEX ... 2
SPCHLIM ... 336
SRC_HELP1 ... 336
SRC_HELP2 ... 337
SRC_HELP3 ... 337
SRC_HELP4 ... 337
SRC_HELP5 ... 338
T
TENURP ... 338
TOTINCR ... 339
TRMODEP ... 339
U
UNITSP ... 340
UPHWKP ... 340
UPKIDP ... 341
USEAID ... 341
USEOTH ... 342
W
WEIGHT_P ... 1
WORKACTP ... 342

APPENDIX D

Public Use Microdata File Record Layout

Observations: 76260
Variables: 758
October 4, 2004

Field	Variable	Format	Length	Position
-----Variables Ordered by Position-----				
1	IDNUM	Num	8	1
2	DISAB	Char	1	9
3	WEIGHT_P	Num	8	10
4	SEX	Char	1	18
5	AGEGRP5	Char	2	19
6	AB1	Char	2	21
7	AB9	Char	2	23
8	AB10A	Char	2	25
9	AB10B	Char	2	27
10	AB10C	Char	2	29
11	AB10D	Char	2	31
12	AB10E	Char	2	33
13	AB10F	Char	2	35
14	AB10G	Char	2	37
15	AB10H	Char	2	39
16	AB10I	Char	2	41
17	AB10_OTH	Char	2	43
18	AB11	Char	2	45
19	AB12	Char	2	47
20	AB12A	Char	2	49
21	AB12B	Char	2	51
22	AB12C	Char	2	53
23	AB12D	Char	2	55
24	AB12E	Char	2	57
25	AB12F	Char	2	59
26	AB12G	Char	2	61
27	AB12H	Char	2	63
28	AB12I	Char	2	65
29	AB12_OTH	Char	2	67
30	AB12L	Char	2	69
31	AB14	Char	2	71
32	AB19	Char	2	73
33	AB25	Char	2	75
34	AB26A	Char	2	77
35	AB26B	Char	2	79
36	AB26C	Char	2	81
37	AB26D	Char	2	83
38	AB26E	Char	2	85
39	AB26F	Char	2	87
40	AB26G	Char	2	89
41	AB26H	Char	2	91
42	AB26_OTH	Char	2	93
43	AB27	Char	2	95
44	AB28	Char	2	97

Field	Variable	Format	Length	Position
45	AB28A	Char	2	99
46	AB28B	Char	2	101
47	AB28C	Char	2	103
48	AB28D	Char	2	105
49	AB28E	Char	2	107
50	AB28F	Char	2	109
51	AB28G	Char	2	111
52	AB28H	Char	2	113
53	AB28I	Char	2	115
54	AB28_OTH	Char	2	117
55	AB36	Char	2	119
56	AB38	Char	2	121
57	AB51	Char	2	123
58	AB52A	Char	2	125
59	AB52B	Char	2	127
60	AB52C	Char	2	129
61	AB52F	Char	2	131
62	AB52G	Char	2	133
63	AB52H	Char	2	135
64	AB52I	Char	2	137
65	AB52J	Char	2	139
66	AB52K	Char	2	141
67	AB52DE	Char	2	143
68	AB53	Char	2	145
69	AB54	Char	2	147
70	AB54A	Char	2	149
71	AB54B	Char	2	151
72	AB54C	Char	2	153
73	AB54F	Char	2	155
74	AB54G	Char	2	157
75	AB54H	Char	2	159
76	AB54I	Char	2	161
77	AB54J	Char	2	163
78	AB54K	Char	2	165
79	AB54DE	Char	2	167
80	AB69	Char	2	169
81	AB70A	Char	2	171
82	AB70B	Char	2	173
83	AB70C	Char	2	175
84	AB71	Char	2	177
85	AB72	Char	2	179
86	AB72A	Char	2	181
87	AB72B	Char	2	183
88	AB72C	Char	2	185
89	AB94	Char	2	187
90	AB95	Char	2	189
91	AB96	Char	2	191
92	AB97	Char	2	193
93	AB98	Char	2	195
94	AB99	Char	2	197
95	AB100	Char	2	199
96	AB101	Char	2	201
97	AB104	Char	2	203

Field	Variable	Format	Length	Position
98	AB105	Char	2	205
99	AB107	Char	2	207
100	AB109	Char	2	209
101	AB110	Char	2	211
102	AB112	Char	2	213
103	AB113_R	Char	1	215
104	AB114_R	Char	1	216
105	AB115	Char	2	217
106	AB116_R	Char	1	219
107	AB117_R	Char	1	220
108	AB118	Char	2	221
109	AB121	Char	2	223
110	AB122	Char	2	225
111	AB123	Char	2	227
112	AB126	Char	2	229
113	AB131A	Char	2	231
114	AB131B	Char	2	233
115	AB131C	Char	2	235
116	AB131D	Char	2	237
117	AB131E	Char	2	239
118	AB131F	Char	2	241
119	AC1	Char	2	243
120	AC2	Char	2	245
121	AC3A	Char	2	247
122	AC3AA	Char	2	249
123	AC3B	Char	2	251
124	AC3AB	Char	2	253
125	AC3C	Char	2	255
126	AC3AC	Char	2	257
127	AC3D	Char	2	259
128	AC3AD	Char	2	261
129	AC3E	Char	2	263
130	AC3AE	Char	2	265
131	AC4	Char	2	267
132	AC5	Char	2	269
133	AC6	Char	2	271
134	AC7A	Char	2	273
135	AC7AA	Char	2	275
136	AC7B	Char	2	277
137	AC7AB	Char	2	279
138	AC7C	Char	2	281
139	AC7AC	Char	2	283
140	AC7D	Char	2	285
141	AC7AD	Char	2	287
142	AC7E	Char	2	289
143	AC7AE	Char	2	291
144	AC8	Char	2	293
145	AC9	Char	2	295
146	AC10	Char	2	297
147	AC11A	Char	2	299
148	AC11AA	Char	2	301
149	AC11B	Char	2	303
150	AC11AB	Char	2	305

Field	Variable	Format	Length	Position
151	AC11C	Char	2	307
152	AC11AC	Char	2	309
153	AC11D	Char	2	311
154	AC11AD	Char	2	313
155	AC11E	Char	2	315
156	AC11AE	Char	2	317
157	AC12	Char	2	319
158	AC13	Char	2	321
159	AC14	Char	2	323
160	AC15A	Char	2	325
161	AC15AA	Char	2	327
162	AC15B	Char	2	329
163	AC15AB	Char	2	331
164	AC15C	Char	2	333
165	AC15AC	Char	2	335
166	AC15D	Char	2	337
167	AC15AD	Char	2	339
168	AC15E	Char	2	341
169	AC15AE	Char	2	343
170	AC16	Char	2	345
171	AC17	Char	2	347
172	AC18	Char	2	349
173	AC19A	Char	2	351
174	AC19AA	Char	2	353
175	AC19B	Char	2	355
176	AC19AB	Char	2	357
177	AC19C	Char	2	359
178	AC19AC	Char	2	361
179	AC19D	Char	2	363
180	AC19AD	Char	2	365
181	AC19E	Char	2	367
182	AC19AE	Char	2	369
183	AC20	Char	2	371
184	AC22	Char	2	373
185	AC23	Char	2	375
186	AC24A	Char	2	377
187	AC24AA	Char	2	379
188	AC24B	Char	2	381
189	AC24AB	Char	2	383
190	AC24C	Char	2	385
191	AC24AC	Char	2	387
192	AC24D	Char	2	389
193	AC24AD	Char	2	391
194	AC24E	Char	2	393
195	AC24AE	Char	2	395
196	AC25	Char	2	397
197	AC26	Char	2	399
198	AC27	Char	2	401
199	AC28A	Char	2	403
200	AC28AA	Num	3	405
201	AC28B	Char	2	408
202	AC28AB	Num	3	410
203	AC28C	Char	2	413

Field	Variable	Format	Length	Position
204	AC28AC	Num	3	415
205	AC28D	Char	2	418
206	AC28AD	Num	3	420
207	AC28E	Char	2	423
208	AC28AE	Num	3	425
209	AC29	Char	2	428
210	AC30	Char	2	430
211	AC31	Char	2	432
212	AC32A	Char	2	434
213	AC32AA	Char	2	436
214	AC32B	Char	2	438
215	AC32AB	Char	2	440
216	AC32C	Char	2	442
217	AC32AC	Char	2	444
218	AC32D	Char	2	446
219	AC32AD	Char	2	448
220	AC32E	Char	2	450
221	AC32AE	Char	2	452
222	AC33	Char	2	454
223	AC34	Char	2	456
224	AC35	Char	2	458
225	AC36A	Char	2	460
226	AC36B	Char	2	462
227	AC36C	Char	2	464
228	AC36D	Char	2	466
229	AC36E	Char	2	468
230	AC37	Char	2	470
231	AC38	Char	2	472
232	AC39A	Char	2	474
233	AC39B	Char	2	476
234	AC39C	Char	2	478
235	AC39D	Char	2	480
236	AC39E	Char	2	482
237	AC40	Char	2	484
238	AC40A	Char	2	486
239	AC40B	Char	2	488
240	AC40C	Char	2	490
241	AC40D	Char	2	492
242	AC40E	Char	2	494
243	AC40F	Char	2	496
244	AC40G	Char	2	498
245	AC40H	Char	2	500
246	AC41	Char	2	502
247	AC42A	Char	2	504
248	AC42B	Char	2	506
249	AC42C	Char	2	508
250	AC42D	Char	2	510
251	AC45A	Char	2	512
252	AC45B	Char	2	514
253	AC45C	Char	2	516
254	AC45D	Char	2	518
255	AC45E	Char	2	520
256	AC45F	Char	2	522

Field	Variable	Format	Length	Position
257	AC45G	Char	2	524
258	AC45H	Char	2	526
259	AC46	Char	2	528
260	AC47A	Char	2	530
261	AC47B	Char	2	532
262	AC47C	Char	2	534
263	AC47D	Char	2	536
264	AC47E	Char	2	538
265	AC47F	Char	2	540
266	AC47G	Char	2	542
267	AC48	Char	2	544
268	AC51	Char	2	546
269	AC52A	Char	2	548
270	AC52B	Char	2	550
271	AC52C	Char	2	552
272	AC52D	Char	2	554
273	AC52E	Char	2	556
274	AC52F	Char	2	558
275	AD1	Char	2	560
276	AD2	Char	2	562
277	AD3	Char	2	564
278	AD4	Char	2	566
279	AD5	Char	2	568
280	AD6	Char	2	570
281	AD8	Char	2	572
282	AD8A	Char	2	574
283	AD8B	Char	2	576
284	AD8C	Char	2	578
285	AD8I	Char	2	580
286	AD9	Char	2	582
287	AD10A	Char	2	584
288	AD10AA	Char	2	586
289	AD10B	Char	2	588
290	AD10AB	Char	2	590
291	AD10C	Char	2	592
292	AD10AC	Char	2	594
293	AD10D	Char	2	596
294	AD10AD	Char	2	598
295	AD10E	Char	2	600
296	AD10AE	Char	2	602
297	AD10F	Char	2	604
298	AD10AF	Char	2	606
299	AD11	Char	2	608
300	AD12A	Char	2	610
301	AD12AA	Char	2	612
302	AD12B	Char	2	614
303	AD12AB	Char	2	616
304	AD12C	Char	2	618
305	AD12AC	Char	2	620
306	AD12D	Char	2	622
307	AD12AD	Char	2	624
308	AD12E	Char	2	626
309	AD12AE	Char	2	628

Field	Variable	Format	Length	Position
310	AD12F	Char	2	630
311	AD12AF	Char	2	632
312	AD12G	Char	2	634
313	AD12AG	Char	2	636
314	AD12H	Char	2	638
315	AD12AH	Char	2	640
316	AD12I	Char	2	642
317	AD12AI	Char	2	644
318	AD12J	Char	2	646
319	AD12AJ	Char	2	648
320	AD13	Char	2	650
321	AD14	Char	2	652
322	AD15A	Char	2	654
323	AD15AA	Char	2	656
324	AD15B	Char	2	658
325	AD15AB	Char	2	660
326	AD15C	Char	2	662
327	AD15AC	Char	2	664
328	AD15D	Char	2	666
329	AD15AD	Char	2	668
330	AD15E	Char	2	670
331	AD15AE	Char	2	672
332	AD15F	Char	2	674
333	AD15AF	Char	2	676
334	AD16	Char	2	678
335	AD17A	Char	2	680
336	AD17AA	Char	2	682
337	AD17B	Char	2	684
338	AD17AB	Char	2	686
339	AD17C	Char	2	688
340	AD17AC	Char	2	690
341	AD17D	Char	2	692
342	AD17AD	Char	2	694
343	AD17E	Char	2	696
344	AD17AE	Char	2	698
345	AD17F	Char	2	700
346	AD17AF	Char	2	702
347	AD17G	Char	2	704
348	AD17AG	Char	2	706
349	AD17H	Char	2	708
350	AD17AH	Char	2	710
351	AD17I	Char	2	712
352	AD17AI	Char	2	714
353	AD17J	Char	2	716
354	AD17AJ	Char	2	718
355	AD18	Char	2	720
356	AD19	Char	2	722
357	AD21A	Char	2	724
358	AD21B	Char	2	726
359	AD21C	Char	2	728
360	AD21D	Char	2	730
361	AD21E	Char	2	732
362	AD21F	Char	2	734

Field	Variable	Format	Length	Position
363	AD21G	Char	2	736
364	LFSTAT	Char	1	738
365	E1HRS	Num	3	739
366	E7HRS	Num	3	742
367	AE8	Char	2	745
368	E9JSTART	Char	2	747
369	AE13	Char	2	749
370	AE14	Char	2	751
371	AE15	Char	2	753
372	AE16	Char	2	755
373	AE17	Char	2	757
374	AE18	Char	2	759
375	AE24	Char	2	761
376	AE25	Char	2	763
377	AE26	Char	2	765
378	AE27	Char	2	767
379	AE28	Char	2	769
380	AE29A	Char	2	771
381	AE29B	Char	2	773
382	AE29C	Char	2	775
383	AE30	Char	2	777
384	AE31	Char	2	779
385	AE32	Char	2	781
386	AE33	Char	2	783
387	AE34	Char	2	785
388	AE35	Char	2	787
389	AE36	Char	2	789
390	AE37	Char	2	791
391	AE38	Char	2	793
392	AE39A	Char	2	795
393	AE39AA	Char	2	797
394	AE39B	Char	2	799
395	AE39AB	Char	2	801
396	AE39C	Char	2	803
397	AE39AC	Char	2	805
398	AE39D	Char	2	807
399	AE39AD	Char	2	809
400	AE39E	Char	2	811
401	AE39AE	Char	2	813
402	AE39F	Char	2	815
403	AE39AF	Char	2	817
404	AE39G	Char	2	819
405	AE39AG	Char	2	821
406	AE40A	Char	2	823
407	AE40AA	Char	2	825
408	AE40B	Char	2	827
409	AE40AB	Char	2	829
410	AE40C	Char	2	831
411	AE40AC	Char	2	833
412	AE40D	Char	2	835
413	AE40AD	Char	2	837
414	AE40E	Char	2	839
415	AE40AE	Char	2	841

Field	Variable	Format	Length	Position
416	AE40F	Char	2	843
417	AE40AF	Char	2	845
418	AE40G	Char	2	847
419	AE40AG	Char	2	849
420	AE41	Char	2	851
421	AE42	Char	2	853
422	AE43	Char	2	855
423	AE44	Char	2	857
424	AE45A	Char	2	859
425	AE45B	Char	2	861
426	AE45C	Char	2	863
427	AE45D	Char	2	865
428	AE45E	Char	2	867
429	AE45F	Char	2	869
430	AE45G	Char	2	871
431	AE46A	Char	2	873
432	AE46B	Char	2	875
433	AE46C	Char	2	877
434	AE46D	Char	2	879
435	AE47	Char	2	881
436	AE48	Char	2	883
437	E49_LSTWK	Char	2	885
438	E50HRS	Num	3	887
439	AE54	Char	2	890
440	AE55	Char	2	892
441	AE56	Char	2	894
442	AE57	Char	2	896
443	AE58	Char	2	898
444	AE59	Char	2	900
445	AE60	Char	2	902
446	AE61	Char	2	904
447	AE62A	Char	2	906
448	AE62B	Char	2	908
449	AE63A	Char	2	910
450	AE63B	Char	2	912
451	AE63C	Char	2	914
452	AE63D	Char	2	916
453	AE63E	Char	2	918
454	AE63F	Char	2	920
455	AE63G	Char	2	922
456	AE64A	Char	2	924
457	AE64B	Char	2	926
458	AE64C	Char	2	928
459	AE64D	Char	2	930
460	AE64E	Char	2	932
461	AE64F	Char	2	934
462	AE64G	Char	2	936
463	AE65	Char	2	938
464	AE66	Char	2	940
465	AE67	Char	2	942
466	AE68	Char	2	944
467	AE69A	Char	2	946
468	AE69B	Char	2	948

Field	Variable	Format	Length	Position
469	AE69C	Char	2	950
470	AE69D	Char	2	952
471	AE69E	Char	2	954
472	AE69F	Char	2	956
473	AE69G	Char	2	958
474	AE70A	Char	2	960
475	AE70B	Char	2	962
476	AE70C	Char	2	964
477	AE70D	Char	2	966
478	AE71	Char	2	968
479	AE72	Char	2	970
480	E73_LSTWK	Char	2	972
481	E82_102	Char	2	974
482	E91_104A	Char	2	976
483	E91_104B	Char	2	978
484	E91_104C	Char	2	980
485	E91_104D	Char	2	982
486	E91_104E	Char	2	984
487	E91_104F	Char	2	986
488	E91_104G	Char	2	988
489	E92_105A	Char	2	990
490	E92_105B	Char	2	992
491	E92_105C	Char	2	994
492	E92_105D	Char	2	996
493	E92_105E	Char	2	998
494	E92_105F	Char	2	1000
495	E92_105G	Char	2	1002
496	E98_106A	Char	2	1004
497	E98_106B	Char	2	1006
498	E98_106C	Char	2	1008
499	E98_106D	Char	2	1010
500	E99_107	Char	2	1012
501	E100_108	Char	2	1014
502	AF1	Char	2	1016
503	AF2	Char	2	1018
504	AF3	Char	2	1020
505	AF4A	Char	2	1022
506	AF4B	Char	2	1024
507	AF4C	Char	2	1026
508	AF4D	Char	2	1028
509	AF4E	Char	2	1030
510	AF5A	Char	2	1032
511	AF5B	Char	2	1034
512	AF5C	Char	2	1036
513	AF6A	Char	2	1038
514	AF6B	Char	2	1040
515	AF6C	Char	2	1042
516	AF6D	Char	2	1044
517	AF6E	Char	2	1046
518	AF6F	Char	2	1048
519	AF6G	Char	2	1050
520	AF6H	Char	2	1052
521	AF7	Char	2	1054

Field	Variable	Format	Length	Position
522	AF8A	Char	2	1056
523	AF8B	Char	2	1058
524	AF8C	Char	2	1060
525	AF8D	Char	2	1062
526	AF8E	Char	2	1064
527	AF8F	Char	2	1066
528	AF8G	Char	2	1068
529	AF8H	Char	2	1070
530	AF9	Char	2	1072
531	AF10	Char	2	1074
532	AF11	Char	2	1076
533	AF12A	Char	2	1078
534	AF12B	Char	2	1080
535	AF12C	Char	2	1082
536	AF12D	Char	2	1084
537	AF12E	Char	2	1086
538	AF12F	Char	2	1088
539	AF12G	Char	2	1090
540	AF12H	Char	2	1092
541	AF13	Char	2	1094
542	AF14	Char	2	1096
543	AF15A	Char	2	1098
544	AF15B	Char	2	1100
545	AF15C	Char	2	1102
546	AF15D	Char	2	1104
547	AF16	Char	2	1106
548	AF17	Char	2	1108
549	AF18A	Char	2	1110
550	AF18B	Char	2	1112
551	AF18C	Char	2	1114
552	AF18D	Char	2	1116
553	AF19	Char	2	1118
554	AF20	Char	2	1120
555	AF21	Char	2	1122
556	AF22A	Char	2	1124
557	AF22B	Char	2	1126
558	AF22C	Char	2	1128
559	AF23	Char	2	1130
560	AF24	Char	2	1132
561	AF25	Char	2	1134
562	AF26	Char	2	1136
563	AF27A	Char	2	1138
564	AF27B	Char	2	1140
565	AF27C	Char	2	1142
566	AF27D	Char	2	1144
567	AF27E	Char	2	1146
568	AF27F	Char	2	1148
569	AF28	Char	2	1150
570	AF29A	Char	2	1152
571	AF29B	Char	2	1154
572	AF29C	Char	2	1156
573	AF29D	Char	2	1158
574	AF29E	Char	2	1160

Field	Variable	Format	Length	Position
575	AF29F	Char	2	1162
576	AF30	Char	2	1164
577	AF31	Char	2	1166
578	AF32	Char	2	1168
579	AF33	Char	2	1170
580	AF34A	Char	2	1172
581	AF34B	Char	2	1174
582	AF34C	Char	2	1176
583	AF34D	Char	2	1178
584	AF34E	Char	2	1180
585	AF34F	Char	2	1182
586	AF34G	Char	2	1184
587	AF34H	Char	2	1186
588	AF34I	Char	2	1188
589	AF34J	Char	2	1190
590	AF34K	Char	2	1192
591	AF34L	Char	2	1194
592	AF35A	Char	2	1196
593	AF35B	Char	2	1198
594	AF35C	Char	2	1200
595	AF35D	Char	2	1202
596	AF36	Char	2	1204
597	AF37A	Char	2	1206
598	AF37B	Char	2	1208
599	AF37C	Char	2	1210
600	AF37D	Char	2	1212
601	AF37E	Char	2	1214
602	AF37F	Char	2	1216
603	AF37G	Char	2	1218
604	AF37H	Char	2	1220
605	AF37I	Char	2	1222
606	AF37J	Char	2	1224
607	AF37K	Char	2	1226
608	AF37L	Char	2	1228
609	AF38	Char	2	1230
610	AF39	Char	2	1232
611	AF40	Char	2	1234
612	AF41	Char	2	1236
613	AF44	Char	2	1238
614	AF45B	Char	2	1240
615	AF45C	Char	2	1242
616	AF45D	Char	2	1244
617	AF45E	Char	2	1246
618	AF45F	Char	2	1248
619	AF45G	Char	2	1250
620	F45_OTH	Char	2	1252
621	AF46	Char	2	1254
622	AF47	Char	2	1256
623	AF48A	Char	2	1258
624	AF48B	Char	2	1260
625	AF48C	Char	2	1262
626	AF48D	Char	2	1264
627	AF48E	Char	2	1266

Field	Variable	Format	Length	Position
628	AF48F	Char	2	1268
629	AF48G	Char	2	1270
630	AF48H	Char	2	1272
631	AF49A	Char	2	1274
632	AF49B	Char	2	1276
633	AF49C	Char	2	1278
634	AF49D	Char	2	1280
635	AF49E	Char	2	1282
636	AF50	Char	2	1284
637	AF51	Char	2	1286
638	AF52	Char	2	1288
639	AF53	Char	2	1290
640	AG1A	Char	2	1292
641	AG1B	Char	2	1294
642	AG1C	Char	2	1296
643	AG2	Char	2	1298
644	AG3	Char	2	1300
645	AG4	Char	2	1302
646	AG5	Char	2	1304
647	AG6A	Char	2	1306
648	AG6B	Char	2	1308
649	AG6C	Char	2	1310
650	AG6D	Char	2	1312
651	AG7A	Char	2	1314
652	AG7B	Char	2	1316
653	AG7C	Char	2	1318
654	AG7D	Char	2	1320
655	AG7E	Char	2	1322
656	AG8C	Char	2	1324
657	AG8D	Char	2	1326
658	AG8E	Char	2	1328
659	AG8F	Char	2	1330
660	AG8G	Char	2	1332
661	AG8H	Char	2	1334
662	AG8I	Char	2	1336
663	AG8J	Char	2	1338
664	AG8K	Char	2	1340
665	AG8L	Char	2	1342
666	AG8M	Char	2	1344
667	AGILIM	Char	2	1346
668	ATTENDRP	Char	1	1348
669	BUILT	Char	2	1349
670	CFAMSTR	Char	1	1351
671	CFINCR	Char	2	1352
672	CHDNUMBER	Char	1	1354
673	COM_LAW	Char	1	1355
674	COSTAIDS	Char	2	1356
675	COSTHELP	Char	2	1358
676	COSTHLTH	Char	2	1360
677	COSTMED	Char	2	1362
678	COSTRMOD	Char	2	1364
679	COSTTRAN	Char	2	1366
680	COWD	Char	1	1368

Field	Variable	Format	Length	Position
681	DEG_AGILP	Char	2	1369
682	DEG_HEARP	Char	2	1371
683	DEG_MOBP	Char	2	1373
684	DEG_OTHEP	Char	2	1375
685	DEG_PAINP	Char	2	1377
686	DEG_SEEP	Char	2	1379
687	DEG_SPCHP	Char	2	1381
688	DEGREE	Char	2	1383
689	DGMFSP	Char	2	1385
690	DTYPE	Char	1	1387
691	EFAMSTR	Char	1	1388
692	EMPINR	Char	2	1389
693	HEARLIM	Char	2	1391
694	HHINCR	Char	2	1393
695	HLNBP	Char	1	1395
696	HLSA	Char	1	1396
697	HMAIN	Char	1	1397
698	HOURS	Num	3	1398
699	ICD9_1	Char	1	1401
700	ICD9_2	Char	1	1402
701	ICD9_3	Char	1	1403
702	ICD9_4	Char	1	1404
703	ICD9_5	Char	1	1405
704	ICD9_6	Char	1	1406
705	ICD9_7	Char	1	1407
706	ICD9_8	Char	1	1408
707	ICD9_9	Char	1	1409
708	ICD9_10	Char	1	1410
709	ICD9_11	Char	1	1411
710	ICD9_12	Char	1	1412
711	ICD9_13	Char	1	1413
712	ICD9_14	Char	1	1414
713	ICD9_15	Char	1	1415
714	ICD9_16	Char	1	1416
715	ICD9_17	Char	1	1417
716	ICD9_18	Char	1	1418
717	ICD9_19	Char	1	1419
718	ICD9_20	Char	1	1420
719	ICD9_21	Char	1	1421
720	ICD9_22	Char	1	1422
721	ICD9_23	Char	1	1423
722	ICD9_24	Char	1	1424
723	LIMDUR	Char	1	1425
724	LOINC	Char	1	1426
725	LSTWK	Char	1	1427
726	MARSTHP	Char	1	1428
727	MOBLIM	Char	2	1429
728	MTNDRP	Char	1	1431
729	NAICS	Char	2	1432
730	NAICSPALS	Char	2	1434
731	NEEDAID	Char	2	1436
732	NEEDHELP	Char	2	1438
733	NEEDOTH	Char	2	1440

Field	Variable	Format	Length	Position
734	NOC	Char	2	1442
735	NOCPALS	Char	2	1444
736	NSTIENP	Char	2	1446
737	NUM_COND	Char	2	1448
738	OTH_LIM	Char	2	1450
739	PAINLIM	Char	2	1452
740	RECHELP	Char	2	1454
741	ROOMSP	Char	2	1456
742	RPAIR	Char	1	1458
743	SEELIM	Char	2	1459
744	SPCHLIM	Char	2	1461
745	SRC_HELP1	Char	2	1463
746	SRC_HELP2	Char	2	1465
747	SRC_HELP3	Char	2	1467
748	SRC_HELP4	Char	2	1469
749	SRC_HELP5	Char	2	1471
750	TENURP	Char	1	1473
751	TOTINCR	Char	2	1474
752	TRMODEP	Char	1	1476
753	UNITSP	Char	1	1477
754	UPHWKP	Char	1	1478
755	UPKIDP	Char	1	1479
756	USEAID	Char	2	1480
757	USEOTH	Char	2	1482
758	WORKACTP	Char	2	1484

APPENDIX E

Document Discussing Changes between the 1991 HALS and the 2001 PALS

Statistics Canada

Housing, Family and
Social Statistics Division

Catalogue no. 89-578-XIE

A New Approach to Disability Data:

Changes between the 1991 Health and Activity Limitation Survey (HALS) and the 2001 Participation and Activity Limitation Survey (PALS)

December 2002

Statistics Canada   Statistique Canada

Canada

How to obtain more information

Specific inquiries about this product and related statistics or services should be directed to: Housing, Family and Social Statistics Division, Statistics Canada, Ottawa, Ontario, K1A 0T6 (telephone: (613) 951-5979).

For information on the wide range of data available from Statistics Canada, you can contact us by calling one of our toll-free numbers. You can also contact us by e-mail or by visiting our Web site.

**National inquiries line**	**1 800 263-1136**
**National telecommunications device for the hearing impaired**	**1 800 363-7629**
**Depository Services Program inquiries**	**1 800 700-1033**
**Fax line for Depository Services Program**	**1 800 889-9734**
**E-mail inquiries**	**infostats@statcan.ca**
**Web site**	**www.statcan.ca**

Standards of service to the public

Statistics Canada is committed to serving its clients in a prompt, reliable and courteous manner and in the official language of their choice. To this end, the Agency has developed standards of service which its employees observe in serving its clients. To obtain a copy of these service standards, please contact Statistics Canada toll free at 1-800-263-1136.

Product information

This product, Catalogue no. 89-578-XIE, is available on the Internet for free. Users can obtain single issues at http://www.statcan.ca/english/IPS/Data/89-578-XIE.htm.

Statistics Canada
Housing, Family and Social Statistics Division

A New Approach to Disability Data:

Changes between the 1991 Health and Activity Limitation Survey (HALS) and the 2001 Participation and Activity Limitation Survey (PALS)

Published by authority of the Minister responsible for Statistics Canada

© Minister of Industry, 2002

All rights reserved. No part of this publication may be reproduced, stored in a retrieval system or transmitted in any form or by any means, electronic, mechanical, photocopying, recording or otherwise, without prior written permission from Licence Services, Marketing Division, Statistics Canada, Ottawa, Ontario, Canada K1A 0T6.

December 2002

Catalogue no. 89-578-XIE

Frequency: Occasional

ISBN 0-662-33230-X

Ottawa

Cette publication est disponible en français (n° 89-578-XIF au catalogue)

Note of appreciation

Canada owes the success of its statistical system to a long-standing partnership between Statistics Canada, the citizens of Canada, its businesses, governments and other institutions. Accurate and timely statistical information could not be produced without their continued cooperation and goodwill.

Symbols

The following standard symbols are used in this Statistics Canada publication:

.	not available for any reference period
..	not available for a specific reference period
...	not applicable
p	preliminary
r	revised
x	suppressed to meet the confidentiality requirements of the Statistics Act
E	use with caution
F	too unreliable to be published

TABLE OF CONTENTS

Page
EXECUTIVE SUMMARY..2

1. INTRODUCTION 4

2. BACKGROUND 5

2.1 Use of census as a survey frame 5

2.2 Health and Activity Limitation Survey (HALS) 5

3. THE CENSUS AND HALS 7

3.1 Overview of the relationship between the census and HALS 7

3.2 Reasons for the existence of false positive and false negative respondents 9

4. PREPARING THE WAY FOR THE 2001 PALS 10

4.1 Research project: Developing “new” filter questions (1997 – 2000) 10

4.1.1 First phase of research: Developing the 2001 Census filter questions (1997) 11

4.1.2 Second phase of research: Evaluation of the new filter questions (1998 – 2000) ...12

4.1.3 Research project conclusions 14

5. OPERATIONAL DEFINITION OF THE 2001 PALS POPULATION 15

6. THE 2001 PALS SURVEY 17

7. CONCLUSIONS 19

EXECUTIVE SUMMARY

Statistics Canada has been involved in conducting surveys on persons with disabilities since the 1980s. In 1986, Statistics Canada conducted its first post-censal disability survey — the *Health and Activity Limitation Survey (HALS)* — after the 1986 Census. HALS is a post-censal survey because it uses the census as a sampling frame to identify its target population. The 1986 Census questionnaire included two general questions on activity limitations and long-term disabilities *(referred to as **"census disability filter questions"**)*. The 1986 HALS respondents were selected through the use of the census information on age, geography and the responses to the disability filter questions.

The HALS survey was repeated in the fall of 1991 immediately after the 1991 Census. The 1991 HALS again used the census filter questions to pre-identify its target population. This population consisted of respondents with the potential of reporting activity limitations to the post-censal survey. A decade later, the 2001 post-censal disability survey, renamed the *Participation and Activity Limitation Survey (PALS),* was carried out in the fall of 2001, about four months after the 2001 Census.

Both HALS and PALS provide detailed information about the demographic and socio-economic situation of persons with disabilities as well as the type and severity of their disabilities. However, the data from the two surveys cannot be compared because of significant differences in the 1991 and 2001 Census filter questions, the sampling plans that were used in HALS and PALS and the content of the questionnaires. The following summarizes the major differences between the 1991 HALS and 2001 PALS:

1) **New census disability filter questions:** The 2001 PALS uses new census disability filter questions to identify its population. The new filter questions are more inclusive than the ones used in 1991.

2) **New sampling plan:** The HALS sample included both respondents who answered YES to the disability filter questions on the census form and those who answered NO. But the 2001 PALS survey sampled only those individuals with positive answers to the 2001 Census filter questions. Respondents who answered NO to the census disability filter questions were excluded from the PALS.

3) **New questionnaire content:** The PALS questionnaire content, including new screening questions, is significantly different from that used in the 1991 HALS questionnaire, in particular the content related to the identification of the types and severity of activity limitations. For example, for the 2001 PALS survey new questions were designed to better identify non-physical disabilities including learning disabilities, developmental disabilities and psychological conditions. In the 1991 HALS, persons with learning disabilities, mental illness and developmental disabilities were grouped together under the category of "Other".

   Compared to the 1991 HALS, the 2001 PALS used a different approach in the identification of the severity of the activity limitations. For example, the HALS severity scale gave more weight to physical disabilities than to non-physical disabilities, but the 2001 PALS severity scale assigned an equal weight to all types of disabilities. In addition, the severity scale in the 1991 HALS was divided into three groups (mild, moderate and severe), while the PALS severity scale was divided into four groups (mild, moderate, severe and very severe).

1. INTRODUCTION

Statistics Canada has been involved in conducting surveys on persons with disabilities since the early 1980s when a special parliamentary committee was formed to study issues surrounding this population. Among its 130 recommendations to the Parliament in 1981 was a directive that Statistics Canada “give high priority to the development and implementation of a long-term strategy which will generate comprehensive data on disabled persons in Canada.”[1]

In 1986, Statistics Canada conducted its first post-censal disability survey — the *Health and Activity Limitation Survey (HALS)* — after the 1986 Census. HALS is a post-censal survey because it uses the census as a sampling frame to identify its target population. The census questionnaire included two general questions on activity limitations and long-term disabilities. The 1986 HALS respondents were selected through the use of the census information on age, geography and the responses to these two general questions.

The HALS survey was repeated in the fall of 1991 immediately after the 1991 Census, but was not conducted after the 1996 Census. The 2001 post-censal disability survey, renamed the Participation and Activity Limitation Survey (PALS), was carried out in the fall of 2001, about four months after the 2001 Census.

Both HALS and PALS provide detailed information about the demographic and socio-economic situation of persons with disabilities as well as the type and severity of their disabilities. However, there are significant differences between the two surveys as PALS has undergone major methodological and content changes compared to HALS. The efforts since 1991 to improve the criteria for identifying the disabled population resulted in changes in the census disability questions as well as changes in the methodology and the operational definition of the population used by the 2001 post-censal survey. This paper presents an overview of the changes that have taken place since the 1991 HALS and of the activities undertaken to improve the identification of disabled population.

[1] Canada. House of Commons. Special Committee on the Disabled and the Handicapped. 1981. *Obstacles - Report of the Special Parliamentary Committee on the Disabled and the Handicapped. p.131.*

2. BACKGROUND

2.1 Use of census as a survey frame

In the early 1980s, following early trials at finding a definition of disability, Statistics Canada chose to adopt the World Health Organization (WHO)'s 1980 model. It defined disability as a limitation in daily activities resulting from an impairment associated with physical or mental conditions or heath problems. This definition was then operationalized through an adaptation of the Activities of Daily Living (ADL) questions developed by the Organization for Economic Cooperation and Development (OECD). The detailed ADL questions were useful to determine the presence of disability and also to identify the types and severity of disabilities.

Given that the objectives of the national database were to provide in-depth information on persons with disabilities according to selected age groups (including children under 15) for each province and territory as well as for the various types and severity of disability, a large sample of disabled persons was required. But early research revealed that Canada's population with disabilities was a relatively small and dispersed sub-population. These database objectives and the characteristics of the disabled population created a methodological challenge. Statistics Canada determined that the census could provide a survey frame that had the potential to meet this challenge and therefore began the development of its first post-censal survey.

2.2 Health and Activity Limitation Survey (HALS)

The 1986 HALS was the first post-censal survey on disability. However, prior to the 1986 HALS, Statistics Canada had to come up with strategies for selecting a sample of potential respondents for this survey. The chosen approach was to add two general questions on activity limitations and long-term disabilities *(referred to as "**census disability filter questions**")* to the 1986 Census long-form questionnaire. The census disability filter questions were used as a "filtering" device to identify potentially disabled persons who could be asked more in-depth disability questions during the post-censal disability survey.

The second post-censal survey on disability was conducted in the fall of 1991 shortly after the 1991 Census. The 1991 HALS again used the census filter questions to pre-identify its target population. This population consisted of respondents with the potential of reporting activity limitations to the post-censal survey.

The census disability questions used on the 1991 Census questionnaire are below:

1. Is this person limited in the kind or amount of activity that he/she can do because of a long-term physical condition, mental condition or health problem:

(a) At home?
☐ No, not limited
☐ Yes, limited

(b) At school or at work?
☐ No, not limited
☐ Yes, limited
☐ Not applicable

(c) In other activities, e.g., transportation to or from work, leisure time activities?
☐ No, not limited
☐ Yes, limited

2. Does this person have any long-term disabilities or handicaps?
☐ No
☐ Yes

As for the 1986 HALS, the 1991 post-censal survey operationalized the WHO's definition of disability with a detailed series of questions on difficulty in performing daily activities in order to determine the presence of a disability, and the type and severity of disability. These detailed daily activity questions are referred to as ***"disability screening questions"***.

The 1991 HALS questionnaire for the population aged 15 and over used 32 questions in the disability screening section of the survey questionnaire. Any respondent having a positive answer to one of the screening questions was identified as disabled. In the 1991 HALS, the types of disabilities identified by these questions were hearing, seeing, speaking, mobility, agility and "other". This last category referred to individuals who were limited because of a learning disability, a mental health condition, an intellectual impairment or because of labeling by others.

3. THE CENSUS AND HALS

3.1 Overview of the relationship between the census and HALS

A post-censal survey uses census information to improve the efficiency of its sample design. That is, compared to a survey, which does not make use of any prior information, the post-censal survey approach will reduce the sample size required to reach a given number of individuals in the target population. There are many advantages to the post-censal approach. For example, because the census contains a large amount of information for each person, its information can be used in the sampling design of a survey on a relatively small and scattered sub-group of the population, such as the disabled population. Operationally, the post-censal approach also reduces the respondent burden by reducing the number of persons who will be asked in-depth disability questions during the survey.

While the use of the census filter questions to pre-identify disabled persons adds to the efficiency of the post-censal approach, it also causes some drawbacks. Since the number of disability questions on the census form is limited, the identification of disabled persons by the census is less precise compared to the post-censal survey which includes a large number of disability screening questions. Consequently, the responses to the census filter questions are not always consistent with the responses to the screening questions on the post-censal survey.

For example, respondents may answer YES to the census disability filter questions, but report no positive answer when responding to the post-censal disability screening questions *(these respondents are referred to as **"false positive respondents"**)*. On the other hand, a respondent could answer NO to the census filter questions, but answer YES to the post-censal screening questions *(referred to as **"false negative respondents"**).* Ideally all respondents reporting a limitation to the census filter questions would also report a limitation to the post-censal survey *(referred to as **"true positive respondents"**).* As well, in an ideal situation, respondents reporting no activity limitations on the census form would also report no limitations in the post-censal survey *(referred to as **"true negative respondents")***.

Table 1 summarizes the four scenarios that occurred in terms of the responses to the 1991 Census filter questions and 1991 HALS screening questions. As indicated in the table, in 1991, almost 80% of the adults classified as positive in the census were also classified as disabled in HALS *(true positives).* But 20% of the adults who answered YES to the census disability questions, later answered NO to the HALS screening questions *(false positives)*. Furthermore, the results indicated that 10% of adults who answered NO to the census disability questions reported YES to the HALS disability screening questions *(false negatives)*.

Table 1 – Relationship between the census filter questions and HALS disability screening questions (1991 HALS)

Response to 1991 Census filter questions	Response to 1991 HALS screening questions		
	**YES**	**NO**	**TOTAL**
**YES**	True positives (80%)	False positives (20%)	100%
**NO**	False negatives (10%)	True negatives (90%)	100%

A further analysis of the HALS data indicated that the false negative group represented almost 53% of the 1991 HALS adult population. In other words, more than half of the adults with disabilities as defined by HALS had originally been missed by the census filter questions. Consequently, sampling only from the group of individuals who reported YES on the census form would seriously bias the post-censal survey results, if the target population is defined by the HALS screening questions. In order to prevent such a bias, the HALS sampling strategy consisted of selecting a sample among individuals who answered YES to the census filter questions *(referred to as the **YES sample**)* and a sample from those who answered NO to the filter questions *(referred to as the **NO sample**)*.

The 1991 HALS data showed that although the NO sample contributed more than half of the HALS disabled population, most of the individuals in that group were mildly disabled; that is, they reported fewer limitations and required fewer accommodations. This means that those who perceived themselves to be mildly limited were more likely to answer NO to the census and YES to the post-censal survey compared to those with severe limitations. The following table compares the level of severity among the disabled population identified in the YES sample and the NO sample.

Table 2 – Severity of disability among YES sample and NO sample (1991 HALS)

	**YES Sample**	**NO Sample**
**Mild**	29%	67%
**Moderate**	40%	27%
**Severe**	31%	6%

Although the sampling of the NO population was necessary to eliminate any bias, it was, however, very costly and time-consuming to implement, given the relative sample size required for the NO population compared to the YES population. This is because the probability of someone in the NO sample answering YES in HALS is about 8 times smaller than the probability of someone in the YES sample to answer YES in HALS. As a result, in the

1991 HALS, approximately 35,000 adults were selected from the YES sample compared to 113,000 from the NO sample.

An additional drawback of this sampling strategy was that it eliminated the possibility of developing comparable datasets on disability using other surveys. To achieve this goal it would be necessary to develop new census filter questions that correspond as closely as possible with the HALS definition of disability. If such filter questions could be developed, it would then possible to use them as a substitute to the long series of HALS screening questions in other social surveys which are not focused on disability. This was not the case for the 1991 Census filter questions. These questions were too imprecise to be used without the follow-up post-censal survey.

3.2 Reasons for the existence of false positive and false negative respondents

A number of factors are responsible for the discrepancy between the proportion of the census disabled population and the HALS disabled population (discrepancy due to the existence of false positives and false negatives). Firstly, as mentioned earlier, the census disability filter questions consist of only two questions, while the post-censal survey includes an extensive number of questions to identify persons with disabilities. Therefore, compared to HALS, the census is less precise in identifying the disabled population, which results in a variation in the number of disabled persons provided by the census and the one available through the post-censal survey.

Secondly, the mode of data collection such as proxy versus non-proxy is likely to influence the difference between the census and post-censal results. A proxy method is one in which a member of the household other than the intended respondent answers on behalf of the respondent. During the census usually one household member completes the census form on behalf of all household members and therefore responds to the disability filter questions for everyone in the household.

However, during the post-censal survey, the information about disability is obtained as much as possible directly from the intended respondent (non-proxy). Proxy is allowed only in special circumstances. For example, the respondent's health condition could make it difficult for him/her to answer the questions. There could be a language barrier with the selected respondent or the respondent could be hospitalized or absent for the duration of the survey. Studies have found that surveys that allow proxy interviews are likely to result in lower disability rates since proxies tend to underestimate the degree of activity limitations experienced by household members.[2]

[2] Binder, D. A. and J. P. Morin. 1988. "Use of questions on activities of daily living to screen for disabled persons in a household survey." *The Canadian Journal of Statistics*. Volume 16, Supplement: 143-156.

Thirdly, the survey context within which the disability questions are put is likely to influence the variation in the proportion of disabled population.[3] The emphasis of the census is on general characteristics of the household, while the HALS is a disability survey that targets individuals who may experience activity limitations. Responses to the disability questions depend to a great extent on the respondent's perception of the situation and are therefore subjective and influenced by the context in which the questions appear.

Fourthly, the time gap between a census and a post-censal survey might have an impact on the differences in responses. It is possible that persons with mild disabilities report limitations at the time of the census, but change their answers after four or five months during the post-censal survey because of the reduction in the degree of their activity limitations. Conversely, activity limitations may appear during the time elapsed between the census and the post-censal survey.

4. PREPARING THE WAY FOR THE 2001 PALS

4.1 Research project: Developing "new" filter questions (1997 – 2000)

In looking ahead to the 2001 post-censal disability survey, Statistics Canada embarked on a research project to determine whether more efficient census filter questions could be developed to improve the identification of the target population in the 2001 survey. The goal was to develop a short set of questions that would cover more of the potentially disabled population, especially those respondents with more severe disabilities. The objective was to find questions that would substantially reduce the false negatives to the point where the NO sample could be completely eliminated from the 2001 post-censal survey.

At the same time, it was hoped that more efficient and inclusive filter questions could also be used as a "global" indicator of disability. That is, the new filter questions could be incorporated in other social survey instruments and allow the definition of disability to become standardized across Statistics Canada's social surveys.

In summary, the two major objectives of the research project were:

- to streamline the collection process of post-censal disability surveys by reducing the required sample size (that is, to eliminate the use of a NO sample);
- to harmonize the definition of disability in Statistics Canada's social surveys including surveys on labour, health, education and Aboriginal people, in order to ensure the development of a comprehensive database on persons with disabilities.

This research project was undertaken between 1997 and 2000. It began with qualitative research, including linguistic analysis of question wording. This was followed by a quantitative evaluation phase in order to assess the efficiency of the new filter questions.

[3] ibid

4.1.1 First phase of research: Developing the 2001 Census filter questions (1997)

The first phase of the research project began in the fall of 1997 with the purpose of developing new filter questions. Given that the disability filter questions were to be used at a broader level (that is, applicable to post-censal disability surveys as well as other social surveys), a certain set of criteria was desired.

First of all, the new filter questions had to be broad and inclusive in order to cover more of the potentially disabled population and to allow persons with all types and levels of disability to take part in the survey. Second, the questions had to be applicable to the whole population, that is, children, adults and the elderly. Third, the new filter questions had to be concise enough to be inserted in a number of survey instruments (whether social or general health surveys) and in the census long form. Fourth, the language used in the questions had to be understandable and clear enough to be used in self-administered surveys, without intervention from an interviewer, but also be applicable in telephone and face-to-face interviews.

A series of tests was conducted to compare the filter questions used in 1991 *(referred to as the **"old filter questions**")* against the **"new filter questions**." The testing results led to the replacement of the old filter questions with the new ones. The new filter questions to be used in the 2001 Census read as follows:

1. Does this person have any **difficulty** hearing, seeing, communicating, walking, climbing stairs, bending, learning or doing any similar activities?
   - ☐ Yes, sometimes
   - ☐ Yes, often
   - ☐ No

2. Does a physical condition **or** mental condition **or** health problem **reduce the amount or the kind of activity** this person can do:

(a) At home?
- ☐ Yes, sometimes
- ☐ Yes, often
- ☐ No

(b) At work or at school?
- ☐ Yes, sometimes
- ☐ Yes, often
- ☐ No
- ☐ Not applicable

(c) In other activities, for example, transportation or leisure?
- ☐ Yes, sometimes
- ☐ Yes, often
- ☐ No

Compared to the old filter questions, the new ones eliminated negative or severe sounding terms, and shifted the limitation from the person to the activity. The term “limit” has been replaced by “reduce” in the new filter questions. The response categories of “sometimes” and “often” have been added to the new filter questions to allow the possibility of reporting limitations that are recurring, but not constant. Finally, the old filter question “Does this person have any long-term disabilities or handicaps?” has been replaced by a shortened version of the ADL questions. This new question, which is worded as “Does this person have any difficulty hearing, seeing, communicating, walking, climbing stairs, bending, learning or doing any similar activities?”, is much more easily understood by respondents. In summary, the new filter questions are more similar to the post-censal disability screening questions.

4.1.2 Second phase of research: Evaluation of the new filter questions (1998 – 2000)

The next step in the research project was to evaluate the performance of the “new” filter questions in a simulated post-censal survey setting. This phase began in October 1998 with the National Census Test (NCT) which was the major field test for the 2001 Census. During the 1998 NCT, two versions of the long census form were tested. One version had the old filter questions and the other version had the new ones. Half of the respondents completed the old version, while the other half completed the new version.

The NCT results showed that the new filter questions with their changed wording did result in higher disability rates. In fact, the version with the old filter questions presented a disability rate of 12.2% among the adult population, while the version with the new filter questions indicated a disability rate of 18.8%. In other words, the new filter questions allowed a larger number of individuals to report a positive answer.

However, it was important to find out whether or not these individuals who answered YES to the filter questions during the NCT would correspond to the post-censal target population. A test (the 1999 HALS Test) was conducted with 12,500 respondents from the 1998 NCT to find out more about the impact of the new filter questions on the number of false positives and false negatives. Respondents who had answered YES or NO to the census questions were contacted by telephone in April 1999 and asked to respond to the 1991 HALS screening questions. Their answers to the screening questions were then correlated with their answers to the census filter questions. It was hoped that the new filter questions would capture more of the disabled population of all levels of severity, but particularly the mildly disabled individuals; a group that was previously coming mainly from the NO sample in 1991.

Table 3 shows the percentage of each severity level screened in by the census filter questions in the 1999 HALS Test. An analysis of the results indicated a stronger relationship between the new filter questions and the HALS screening questions compared to the old ones. That is, the new filter questions screened in more of the disabled population for all three levels of severity (mild, moderate and severe). This was particularly true for persons with milder activity limitations. For example, the old census filter questions screened in 12%

of persons with mild disabilities (88% of the mild disabled came from the NO sample), while the new filter questions screened in 23% of the mildly disabled persons. In other words, for each level of severity, the new filter questions screened in a higher proportion of the post-censal target population.

Table 3 – Severity of disability identified by OLD and NEW filter questions (1999 HALS Test)

	OLD filter questions	NEW filter questions
**Mild**	12%	23%
**Moderate**	45%	61%
**Severe**	76%	84%

The PALS Pilot Test was conducted between May and October 2000 to prepare for the 2001 post-censal survey, but also to gather further evidence on the performance of the new filter questions. This test was designed mainly to answer the question: *What population would be left out if the NO sample was excluded from the post-censal survey?* The results showed that the population that would be left out is the less severely disabled persons. For instance, only 2.4% of the pilot test's false negative respondents were severely disabled, compared to 20.6% of the true positive respondents (See Table 4).

Table 4 – Severity of disability among true positives and false negatives (PALS Pilot Test)

	True positives	False negatives
**Mild**	43.5%	84.6%
**Moderate**	35.9%	13.0%
**Severe**	20.6%	2.4%

Not only were there fewer severely disabled respondents among false negative respondents than in the true positives, but analysis of their characteristics indicated that the severely disabled false negative respondents reported fewer problems than the severely disabled true positive respondents. For example, a smaller proportion of severe false negative respondents reported being completely unable to work or receiving disability-related income compared to the severe true positive respondents. These results showed that the population that would not be included in the survey, if the NO sample were eliminated, was on average relatively mildly disabled.

4.1.3 Research project conclusions

The results of the testing projects conducted between 1997 and 2000 were consistent in showing that while the coverage of the new filter questions was not perfect, it was much improved compared to the situation in 1991. That is, the new filter questions covered a larger proportion of the target population than the 1991 filter questions, and most importantly, did not leave out a significant portion of the population with severe disabilities.

The new set of questions could also be used as a “global disability indicator” in other Statistics Canada’s surveys, thereby increasing the scope of available data on persons with disabilities. Although it was recognized that the resulting disability rates would vary depending on the survey context and methodology, this improved consistency in the measurement of disability would enhance the usefulness of data on persons with disabilities.

Therefore, it was decided that the new filter questions would be used in the 2001 Census form. Table 5 presents the disability rates obtained from the census and post-censal surveys in 1991 and 2001.

Table 5 – Disability rates by age group in census and post-censal disability surveys, 1991 and 2001 (population in households)

Age Groups	1991		2001	
	**Census**	**HALS**	**Census***	**PALS**
**Total population**	8.4	14.7	16.0	12.4
**Under 15**	2.6	7.0	5.0	3.3
**15 and over**	10.0	16.8	18.6	14.6

***** *Full results from the 2001 Census disability filter questions will be available from Statistics Canada in early 2003.*

5. OPERATIONAL DEFINITION OF THE 2001 PALS POPULATION

Figure 1 presents a graphical illustration of the sampling strategy used by the 2001 PALS. As shown in the graph, the PALS interviews begin with the same filter questions that appear on the 2001 Census form followed by a series of detailed disability screening questions. The filter and screening questions are to be administered to all respondents. Respondents who answer YES to at least one of the filter or screening questions are included in the disabled population. These respondents continue the interview to the end. The interview for the other respondents (that is, those respondents who answer NO to filter questions as well as screening questions) stops after the screening questions. These respondents are not part of the post-censal disabled population.

The target population of the 2001 PALS is therefore those respondents who indicate a limitation to the 2001 Census filter questions and who maintain some evidence of limitation in PALS through the PALS filter or screening questions. It therefore eliminates from its target population respondents who no longer report a limitation at the time of the PALS interview.

In summary, the following new strategies were implemented for the 2001 Census and 2001 post-censal survey:

- Use of the new filter questions on the census form and the 2001 post-censal disability survey as well as other Statistics Canada's social surveys;
- Exclude the NO sample (that is, those respondents who answered NO to the disability filter questions on the census form*)* from the 2001 post-censal sample;
- The 2001 PALS disabled population consists of those respondents who answer YES to the census filter questions and then during the PALS survey report a limitation through a positive answer to either filter questions or screening questions included in the PALS questionnaire.

Adopting the above new strategies allows the 2001 post-censal survey to efficiently identify a population with all levels of severity, including those persons with severe disabilities.

Figure 1 - PALS 2001: Operational definition of population with disabilities

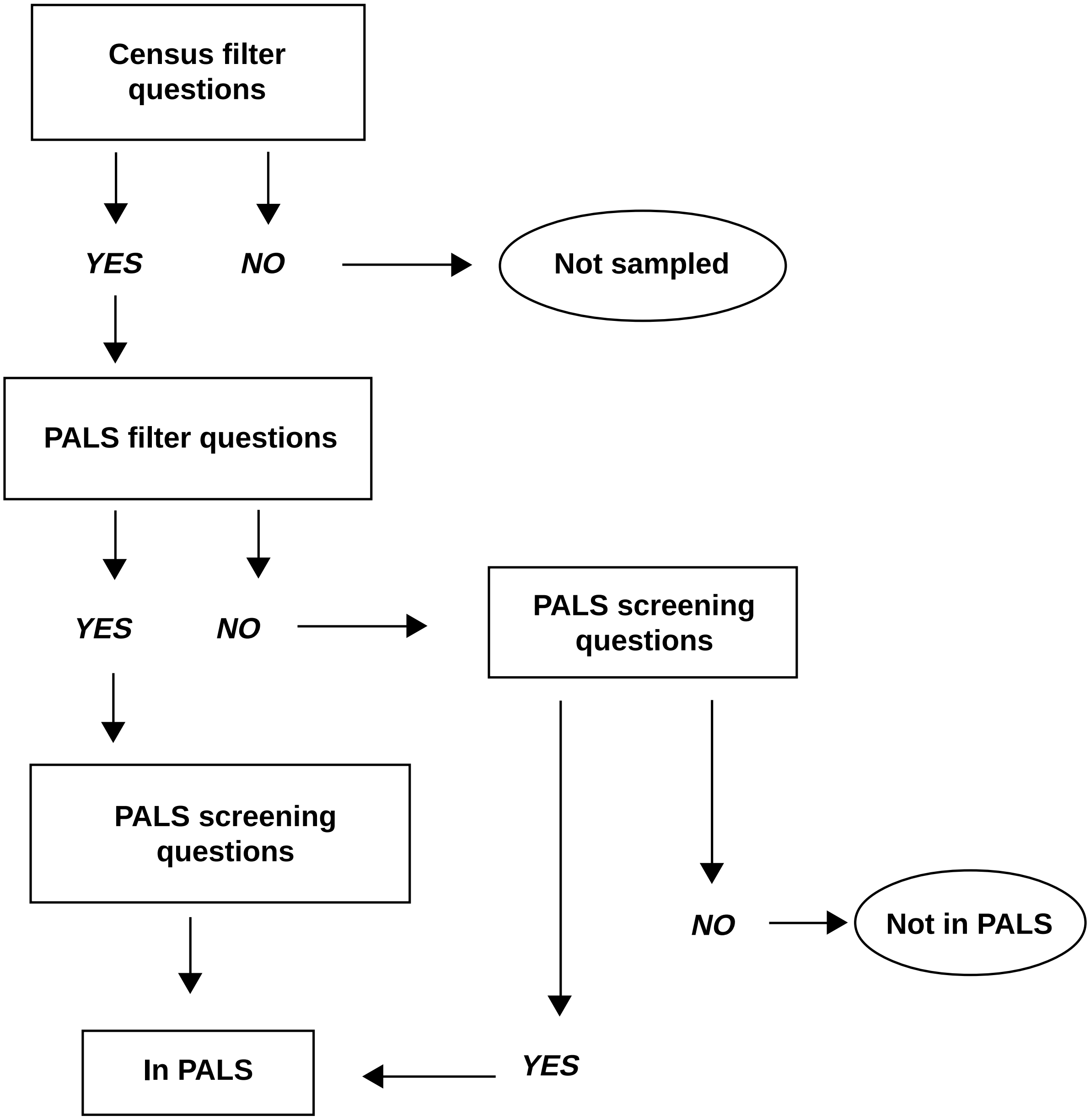

6. THE 2001 PALS SURVEY

In the decade between 1991 and 2001, the World Health Organization revised its model and classification of disability. The *International Classification of Functioning, Disability and Health (ICF)* was officially launched in 2001. Statistics Canada's 2001 post-censal disability survey uses the ICF as its framework, and views disability as the interrelationship between body functions, activities and social participation, while recognizing the role of the environment as providing barriers or facilitators. The name change, from *Health and Activity Limitation Survey* to *Participation and Activity Limitation Survey*, serves to underscore this updated view of disability and the significant changes implemented in the survey.

The Participation and Activity Limitation Survey was conducted between September 2001 and January 2002. The survey population for both children (under 15) and adults (15 and over) was selected through the two new census disability filter questions on the 2001 Census questionnaire. After the 2001 Census, a sample of individuals who reported an activity limitation to the (new) census filter questions was selected. Respondents who answered NO to the census disability questions were not included in the PALS sample.

The population covered by the survey was persons residing in private and some collective households in the ten provinces. Persons living in institutions and persons living in the three territories as well as people living on the First Nations reserves were excluded from the survey. The interviews with respondents were conducted by phone with the interviewers completing a paper and pencil questionnaire. A small number of face-to-face interviews was conducted as well.

The PALS interview began with the census disability filter questions followed by a series of detailed screening questions on activity limitations. If respondents answered NO to all of the filter questions and screening questions, the interview ended. But if respondents answered YES to any of the filter questions or screening questions, the interview continued to collect information on the impact of disability on their everyday activities and other aspects of their life, such as education, employment, leisure, transportation and accommodation. The PALS sample size was 43,000 consisting of approximately 35,000 adults and 8,000 children. The response rate was 82.5%.

The PALS survey collected data on:

- difficulties with certain daily activities, such as moving around, hearing, seeing, communicating and learning;
- type and severity of the activity limitation;
- specialized equipment and aids that are used and/or needed;
- help required to complete everyday activities;
- impact on employment, education, leisure, accommodation and transportation;
- information on out-of-pocket expenses related to specialized aids and services, medications, transportation, etc.;
- economic characteristics, such as insurance coverage and sources of income.

There are major differences between the HALS and PALS with respect to the questionnaire content. For example, compared to the 1991 HALS, considerable changes have been made to the disability screening questions of the 2001 PALS. In 1991, 32 screening questions were used to identify six types of activity limitation among adults: hearing, seeing, speech, mobility, agility and other (a grouping of non-physical disabilities related to psychological conditions, learning, memory, etc.). The 2001 PALS collected data on ten types of limitation: hearing, seeing, speech, mobility, agility, learning, developmental disability or disorder, psychological, memory limitation and chronic pain. Major changes were implemented in the PALS disability screening section of the questionnaire compared to the 1991 HALS:

- Most of the 1991 HALS disability screening questions had only two response options of “YES, HAS DIFFICULTY” and “NO DIFFICULTY”. However, the choice between a YES and NO response was not always easy or clear for many respondents, in particular for those with mild or cyclical disabilities whose limitations were not significant or constant. Therefore, where applicable, the 2001 PALS screening questions offered respondents a new scaled-response category. For example, for questions on mobility and agility, respondents were provided with the option of selecting response categories of “YES, SOMETIMES”, “YES, OFTEN OR ALWAYS” or “NO”. In some questions, the frequency and intensity of the limitations were probed to provide more detail on the severity of the disability.

- In the PALS, some screening questions have been more precisely defined to avoid ambiguity and some have been expanded. For example, in the 2001 survey new questions were designed to better identify non-physical disabilities including learning disabilities, developmental disabilities and psychological conditions. In the 1991 HALS, persons with learning disabilities, mental illness and developmental disabilities were grouped together under the category of “Other”. This significant content development in the 2001 PALS shifts the focus from almost exclusively physical disabilities and provides a better balance between physical and non-physical disabilities.

- Another major difference between HALS and PALS is related to the approach used in the identification of the severity of the activity limitations. In 1991, a severity scale had been developed using the responses to the screening questions. Each respondent received a severity score by adding together the individual’s responses to all activity limitation questions. One point was scored for each partial loss of function and two points were scored for each total loss of function. The total score was then divided into three severity levels: mild, moderate and severe. Since some types of disabilities were identified through the use of many questions, they had more weight in the severity scale. As a result, the 1991 HALS severity scale gave more weight to certain disabilities (such as mobility and agility) than to others (such as the “other” category).

For the 2001 PALS severity scale, an index measuring the severity of the disability was constructed based on the answers to the survey questions. Points were given according to the intensity and the frequency of the activity limitations reported by the respondent. A single score was computed for each type of disability. Each score was then standardized in order to have a value between 0 and 1. The final score was the average of the scores for each type of disability. Since the survey questions differed depending on the age of the respondent, a different scale was constructed for adults (15 years and over), for children under 5 and for children aged 5 to 14. Each scale was then divided into different severity levels. The scale for adults and for children aged 5 to 14 was divided into four groups (that is, mild, moderate, severe and very severe), while the scale for children under 5 was divided into two groups (that is, mild to moderate and severe to very severe). The PALS severity scale is therefore equally weighted for all types of disabilities and this results in a different severity profile than in the 1991 HALS.

7. CONCLUSIONS

Both HALS and PALS represent post-censal surveys of persons with disabilities in Canada. They collect information on the type and severity of disabilities and the impact of disability on everyday activities as well as information on the barriers related to education, employment, transportation, accommodation and leisure activities. However, the data from the two surveys cannot be compared because of significant differences in the 1991 and 2001 Census filter questions, the sampling plans that were used in HALS and PALS, the operational definition of the post-censal survey population and the content of the questionnaires.

The following summarizes the major differences between the 1991 HALS and 2001 PALS:

1) **New filter questions:** The 2001 PALS uses new census disability filter questions to identify its population. The new filter questions are more inclusive than the ones used in 1991;

2) **New sampling plan:** The HALS sample included both respondents who answered YES to the disability filter questions on the census form and those who answered NO. But the 2001 PALS survey sampled only those individuals with positive answers to the 2001 Census filter questions. Respondents who answered NO to the census disability filter questions were excluded from the PALS.

3) **New questionnaire content:** The PALS questionnaire content, including new screening questions, is significantly different from that used in the 1991 HALS questionnaire, in particular the content related to the identification of the types and severity of activity limitations. This expanded content provides more detailed information regarding the type of disabilities. In addition, the new severity scale takes into account the whole range of disabilities.

APPENDIX F

PALS 2001 (PUMF)

Development of Severity Scale

Global Severity Index

A global severity index was constructed using all questions for each type of disability in the PALS 2001 questionnaire. Points were assigned to each question based on severity, the maximum score being given for someone who is totally disabled in all areas. For each type of disability, there are essentially two types of questions: intensity and frequency. Some intensity questions are asked in different contexts. In this case, the mean score is used to measure intensity. The product of frequency and intensity was used to measure severity for each disability type.

For some disability types, more than one question is used to determine the limitation being measured (for example, there are three questions for hearing problems in the adult questionnaire). Since the number of questions varies depending on the disability, the indices were standardized for each type of disability. This will avoid over-representing disability types for which numerous functional limitations are being measured.

CONSTRUCTION OF INDEX

The overall score is calculated by taking the average of all standardized scores.

CREATION OF SEVERITY LEVELS

After discussion with some data users, it was decided that the disability scale should be cut into four severity levels. The severity levels were created by examining the distribution of the global severity scores. The distribution was divided into deciles. The first decile corresponds to the 10% of people with the lowest disability scores. Then, the second decile corresponds to the next 10% of people with the lowest disability scores, etc. The average score was calculated for each decile and a plot of this average score as a function of the decile was produced. No obvious cut-off in the global severity score distribution exists. Since a relatively simple method was desirable for data users, a rather intuitive approach was developed. Thus, the severity levels were essentially determined using a graphical method.

In a first step, an attempt was made to identify a "natural cut-off point" in the scale. The beginning of the distribution is fairly linear up to 70th percentile and then, the slope starts to increase more and more rapidly. This cut-off point in the distribution seems to correspond to a score around 1/9. This particular score corresponds to the score of someone with the maximum score for one disability type and nothing else. Many such cases were found in the sample. Of course, there are a number of ways to obtain a score of 1/9. Because of the particular interpretation of this point, the cut-off was chosen to be exactly 1/9.

These two groups were then subdivided into two parts. These two cut-off points are equivalent to respectively half and twice the maximum score obtained for one disability. Thus, respondents with a score equivalent to less than half the maximum score for one disability are included in Level 1, while those with an equivalent score between half and once

the maximum score for one disability are in Level 2. Those with an equivalent score between one and twice the maximum score for one disability are in Level 3, while those with a score equivalent to more than twice the maximum score for one disability are in Level 4.

The interpretation of these levels is as follows: persons in Level 1 are less severely disabled than persons in Level 2, who in turn are less severely disabled than persons in Level 3, and so on. However, for practical purposes, these levels were assigned names. The terms "mild," "moderate," "severe" and "very severe" to designate levels 1 to 4 were assigned. It should be noted that there is no judgment associated with the use of this terminology.

In order to enhance the PUMF's analytical strengths, a severity index was also developed for each of the seven disability types used in the PUMF, including hearing, seeing, speech, mobility, agility, pain and "other" disabilities. These indices were produced using the same approach that was used for the global index, but they contain only two severity levels: the less severe and the more severe. Here is the list of variables identifying each index :

DEG_HEARP
DEG_SEEP
DEG_SPCHP
DEG_MOBP
DEG_AGILP
DEG_PAINP
DEG_OTHEP

APPENDIX G

PALS 2001 (PUMF)

Type of disability among adults

The PALS survey questions allow the identification of the following types of disabilities among adults aged 15 and over :

Hearing: Difficulty hearing what is being said in a conversation with one other person, in conversation with three or more persons or in a telephone conversation.

Seeing: Difficulty seeing ordinary newsprint or clearly seeing the face of someone from 4 metres (12 feet).

Speech: Difficulty speaking and/or being understood.

Mobility: Difficulty walking half a kilometre or up and down a flight of stairs, about 12 steps without resting, moving from one room to another, carrying an object of 5 kg (10 pounds) for 10 metres (30 feet) or standing for long periods.

Agility: Difficulty bending, dressing or undressing oneself, getting into and out of bed, cutting own toenails, using fingers to grasp or handling objects, reaching in any direction (for example, above one's head) or cutting own food.

Pain: Limited in the amount or kind of activities that one can do because of a long-term pain that is constant or reoccurs from time to time, for example, recurrent back pain.

Other: For confidentiality reasons related to the PUMF, 5 types of disabilities were reclassified into the "Other" disabilities category. The disabilities included in this category are: "Learning", "Memory", "Developmental", "Psychological" and "Unknown".

APPENDIX H
Weighted and Unweighted Frequencies for the Population Without Disabilities

VARIABLE AND VALUE CODE	UNWEIGHTED	WEIGHTED
AGEGRP5		
1	5777	1917866
2	5399	1814800
3	4758	1756290
4	5185	1920840
5	6218	2275087
6	6237	2270257
7	5283	2010326
8	4561	1728281
9	3316	1248457
10	2515	947649.5
11	2261	805529.1
12	1878	628044.3
13	1240	422421.4
14	630	196908.2
15	292	82661.41

ATTENDRP		
1	38874	14286241
2	2800	1016779
3	7568	2586502
9	6308	2135895

CFAMSTR		
1	27028	9942316
2	5555	2026781
3	2701	969422.4
4	10329	3535632
5	8982	3203458
9	955	347807.6

CFINCR		
1	2022	568038.3
2	1632	533582.8
3	2535	822048
4	2496	819999.1
5	5737	1977517
6	6016	2118109
7	5607	1994795
8	5201	1877528
9	8971	3282542
10	15292	5670023
11	41	361233.5

VARIABLE AND VALUE CODE	UNWEIGHTED	WEIGHTED
CHDNUMBER		
1	32635	11575417
2	8482	3113710
3	9335	3461938
4	4136	1526214
9	962	348138.5
COM_LAW		
1	49963	17987016
2	5587	2038402
COWD		
1	138	51686.15
2	14207	4913839
3	665	245806.2
4	902	340662.6
5	36723	13387653
6	2060	771123.2
7	855	314646.8
DGMFSP		
1	26105	9315100
2	2336	868995.1
3	1281	471863.6
4	1494	558309.8
5	2370	882679.2
6	4987	1850628
7	1114	416117.5
9	4879	1778532
10	2493	932615.9
11	2095	787481.4
12	65	24717.47
91	7	330.9556
93	6324	2138047
EFAMSTR		
1	31912	11715044
2	2445	881713.6
3	955	347807.6
4	1796	638884
5	10506	3604348
6	7936	2837619

VARIABLE AND VALUE CODE	UNWEIGHTED	WEIGHTED
EMPINR		
0	9409	3314910
1	5613	1953404
2	3984	1418486
3	3354	1205610
4	3139	1128433
5	6383	2351545
6	5753	2133777
7	4028	1510465
8	2767	1044321
9	2831	1072398
10	1981	756173.8
11	6301	2135564
91	7	330.9556
HHINCR		
1	2046	733327.3
2	1165	407285.7
3	1880	644066.7
4	2053	697804.6
5	5113	1774854
6	5574	1974493
7	5542	1969266
8	5397	1948150
9	9480	3459014
10	17293	6416824
91	7	330.9556
HLNBP		
1	36277	12910236
2	12790	4748842
3	6483	2366339
HLSA		
1	12797	4510569
2	13325	4806225
3	5372	1954467
4	7892	2916534
5	9856	3701726
6	6301	2135564
9	7	330.9556

VARIABLE AND VALUE CODE	UNWEIGHTED	WEIGHTED
HMAIN		
1	29441	10525608
2	955	347807.6
3	25154	9152002

VARIABLE AND VALUE CODE	UNWEIGHTED	WEIGHTED
HOURS		
-3	6301	2135564
-1	7	330.9556
0	14568	5132122
1	22	7138.608
2	86	28950.86
3	77	26234.81
4	102	36736.38
5	221	80198.97
6	153	54528.24
7	74	24734.76
8	307	110676.6
9	64	23665.59
10	562	205086.2
11	43	14806.63
12	304	106716.9
13	59	21135.89
14	108	38742.06
15	563	201494.5
16	315	111552
17	65	22138.48
18	143	51602.69
19	35	12324.77
20	1099	394611.7
21	112	40135.66
22	100	35129.16
23	84	32306.94
24	371	134155.1
25	611	220244.2
26	80	27224.21
27	76	26570.19
28	186	68859.27
29	65	23385.82
30	1388	506397.5
31	38	13905.38
32	429	153659.8
33	89	31919.98
34	118	43878.92
35	2084	777331.3
36	479	178316.8
37	926	346484.7
38	1192	438026
39	150	54915.47

VARIABLE AND VALUE CODE	UNWEIGHTED	WEIGHTED
40	12060	4450370
41	99	36973.94
42	424	159486.4
43	167	60210.01
44	476	176049.2
45	1499	560649.3
46	151	56228.51
47	79	29806.06
48	572	214807.9
49	43	15918.37
50	2282	855049.1
51	33	12768.29
52	127	47852.79
53	55	18897.25
54	63	22046.03
55	442	167067.1
56	111	42101.56
57	27	9832.242
58	59	21133.44
59	14	4699.859
60	1532	567198
61	4	1552.915
62	20	6798.801
63	25	8563.695
64	19	6293.715
65	164	60581.74
66	1147	418540.7

LFSTAT		
1	35899	13189776
2	2856	997678.3
3	10487	3702068
9	6308	2135895

LOINC		
1	46545	16884494
2	8042	2790017
3	963	350905.9

LSTWK		
1	4674	1688018
2	2776	969698.6
3	38567	14141879
4	3225	1089926
5	6308	2135895

VARIABLE AND VALUE CODE	UNWEIGHTED	WEIGHTED
MARSTHP		
1	2562	950649.9
2	33122	12167041
3	1288	470136.5
4	16512	5733870
5	2066	703720.1
MTNDRP		
1	31322	11118372
2	13342	4947895
3	10886	3959150
NAICS		
1	14207	4913839
2	1590	575129.6
3	426	152288.4
4	326	122501.9
5	2209	799002.8
6	5645	2077875
7	1795	655150.1
8	4755	1708867
9	2026	749476.8
10	1178	435195.8
11	1645	613334.9
12	636	235984.9
13	2525	952847.9
14	37	13610.12
15	1597	574228.3
16	2678	1003231
17	3934	1446190
18	863	312628.9
19	3190	1121993
20	1985	719036.7
21	2303	843003.7
NOC		
0	7	330.9556
1	14200	4913508
2	3990	1500572
3	7230	2668989
4	2663	995544.2
5	2118	785600
6	3238	1209247
7	1188	433942.2
8	10225	3634263
9	5833	2130329

VARIABLE AND VALUE CODE	UNWEIGHTED	WEIGHTED
10	1912	682366.8
11	2946	1070725

NSTIENP		
1	30385	10896345
2	22699	8242083
3	1029	367803.8
4	482	171378
99	955	347807.6

ROOMSP		
1	3249	1164304
2	5674	2037597
3	8682	3104001
4	9011	3233075
5	8119	2945074
6	7818	2835510
7	4836	1751995
8	3684	1330912
9	3522	1275143
99	955	347807.6

RPAIR		
1	35405	12914126
2	955	347807.6
3	4168	1432391
4	15022	5331092

SEX		
1	27420	9868731
2	28130	10156686

TENURP		
1	975	353055.9
3	40005	14454135
4	14570	5218227

TOTINCR		
0	3238	1116567
1	7107	2473862
2	5662	1999602
3	5630	1969428
4	4934	1734643
5	8269	3008118

VARIABLE AND VALUE CODE	UNWEIGHTED	WEIGHTED
6	7066	2606421
7	4802	1786371
8	3185	1193130
9	3239	1224066
10	2411	912878.1
11	7	330.9556
TRMODEP		
1	962	342510.9
2	27194	9994962
3	7	330.9556
4	17578	6163897
6	2968	1038715
8	4177	1534691
9	2664	950310.5
UNITSP		
1	5787	2073690
2	15910	5725689
3	11003	3983258
4	13012	4714833
5	9838	3527947
UPHWKP		
1	5510	1927033
2	13668	4891411
3	17491	6359834
4	10823	3936696
5	5679	2056428
6	2372	853682.9
9	7	330.9556
UPKIDP		
1	33648	12045815
2	5401	1937515
3	5675	2084454
4	4034	1482484
5	3035	1110094
6	3750	1364724
9	7	330.9556

VARIABLE AND VALUE CODE	UNWEIGHTED	WEIGHTED
WORKACTP		
1	6308	2135895
2	7899	2777944
3	1262	443571.8
4	1243	435289.8
5	1644	568678.4
6	2210	787263.6
7	1744	614409.9
8	1882	682562.2
9	1006	350977.4
10	4593	1689902
11	1265	456371.8
12	21373	7948470
13	3121	1134080
