# Supplementary material for: Smoking and alcohol consumption patterns among elderly Canadians with mobility disabilities
Source: BMC Res Notes. 2013 Jun 4;6:218. doi: 10.1186/1756-0500-6-218 (PMC3680044; doi:10.1186/1756-0500-6-218)
Supplement: Additional file 2 — Provides a summary of Participation and Activity Limitation Survey (PALS) 2001 disability scale. [file 1756-0500-6-218-S2.pdf]

Summary of Participation and Activity Limitation Survey (PALS) 2001 Disability Scale

SCORES

A disability index was constructed using all questions for each type of disability in the PALS 2001 questionnaire. Points were assigned to each question based on severity, the maximum score being given for someone who is totally disabled in all area. For each type of disability, there are essentially two types of questions: intensity and frequency. Some intensity questions are asked in different contexts. In this case, the mean score is used to measure intensity. The product of frequency and intensity was used to measure severity for each disability type.

For some disability types, more than one functional limitation is being measured (for example, there are three for hearing problems). Since the number of functional limitations varies depending on the disability, the indices were standardized for each type of disability. This will avoid over-representing disability types for which numerous functional limitations are being measured.
Partial non-response was treated by imputing the average score of respondents sharing some of the characteristics of the partial non-respondent.

CONSTRUCTION OF INDEX

The overall score is calculated taking the average of all standardized scores. But, since there is a strong relationship between learning difficulties and developmental disability, points are given only for developmental disability to respondents reporting both types of disability.

In order to avoid over-representing individuals with correlated disability types, an unequally weighted scale was considered. Instead of having a weight of 1, disability types that are strongly correlated would have a smaller weight in the global score. Since it is difficult to justify the use of unequal weights, this option was rejected.

For children, since questions differ according to their age, two different scales were created, one for children between 0 and 4 and one for children between 5 and 14.

CREATION OF CLASSES

After discussion with some data users, it was decided that the disability scale should be cut into four severity classes. The severity classes were created in examining the distribution of the global severity score. The distribution has been

divided into deciles. The first decile corresponds to the 10% of people with the lowest disability scores. Then, the second decile corresponds to the next 10% of people with the lowest disability scores, etc. The average score was calculated for each decile and a plot of this average score as a function of the decile was produced. No obvious cut-off in the global severity score distribution exists. Since a relatively simple method was desirable for data users, a rather intuitive approach was developed. Thus, the severity classes were essentially determined using a graphical method.

In what follows, only the approach for creating the adult severity classes is described, the approach for children being similar. In a first step, an attempt was made to identify a “natural cut-off point” in the scale. The beginning of the distribution is fairly linear up to 70th percentile and then, the slope starts to increase more and more rapidly. This cut-off point in the distribution seems to correspond to a score around 1/9. This particular score corresponds to the score of someone with the maximum score for one disability type and nothing else. Many such cases were found in the sample. Of course, there are a number of ways to obtain a score of 1/9. Because of the particular interpretation of this point, the cut-off was chosen to be exactly 1/9.

These two groups were then subdivided into two parts. These two cut-off points are equivalent to respectively half and twice the maximum score obtained for one disability. Thus, respondents with a score equivalent to less than half the maximum score for one disability are included in Class 1, while those with an equivalent score between half and once the maximum score for one disability are in Class 2. Those with an equivalent score between one and twice the maximum score for one disability are in Class 3, while those with a score equivalent to more than twice the maximum score for one disability are in Class 4.

This classification system has the advantage of being easy for all users to understand and interpret. In light of the relatively subjective nature of this classification and in order to avoid any misinterpretation, it is preferable not to use specific terms to characterize the classes. The interpretation of these classes is as follows: persons in Class 4 are more severely disabled than persons in Class 3, who in turn are more severely disabled than persons in Class 2, and so on. However, for practical purposes, these classes were assigned names. The terms “mild,” “moderate,” “severe” and “very severe” to designate classes 1 to 4 were assigned. It should be noted that there is no judgment associated with the use of this terminology.

Further information is provided by Statistics Canada:

Statistics Canada. Participation and Activity Limitation Survey 2006: Technical and Methodological Report. Ottawa, Ontario: Statistics Canada; 2007. (Catalogue no. 89-628-XIE).
